# Unprecedented nucleophile-promoted 1,7-S or Se shift reactions under Pummerer reaction conditions of 4-alkenyl-3-sulfinylmethylpyrroles

**DOI:** 10.3762/bjoc.14.250

**Published:** 2018-10-29

**Authors:** Takashi Go, Akane Morimatsu, Hiroaki Wasada, Genzoh Tanabe, Osamu Muraoka, Yoshiharu Sawada, Mitsuhiro Yoshimatsu

**Affiliations:** 1 Department of Chemistry, Faculty of Education, Gifu University, Yanagido 1-1, Gifu 501-1193, Japan; 2 Department of Chemistry, Faculty of Regional Study, Gifu University, Yanagido 1-1, Gifu 501-1193, Japan; 3 School of Pharmacy, Kindai University, 3-4-1 Kowakae, Higashi-osaka, Osaka 577-8502, Japan; 4 Life Science Research Center, Gifu University, Yanagido 1-1, Gifu 501-1193, Japan

**Keywords:** hydroamination, Pummerer reaction, pyrrole, pyrroloazepine, 1,7-sulfur shift

## Abstract

A unique 1,7-S- and Se-shift reaction under Pummerer reaction conditions of 4-alkenyl-3-sulfinyl- and seleninylpyrroles was described. The usual Pummerer reaction of 4-(alkenylaminomethyl)-3-phenylsulfinylpyrroles and a successive reaction with tetrabutylammonium hydroxide (TBAH) yielded either pyrrolo[3,2-*c*]azepines or *N*-pyrrol-3-ylmethyl-*N*-(4-hydroxy-3-sulfanylpropyl)-*p*-toluenesulfonamides (diols). *Seleno*-Pummerer reactions of 3-selanylmethylpyrroles also proceeded via in situ generation of selenoxides, followed by a treatment with TBAH.

## Introduction

Pyrrolo- and indoloazepine skeletons are chemical frameworks present in marine natural products [1–2] such as hymenialdisines [3–4], latonduines [5], paullones, kenpaullones and alsterpaullones [6–7]. These compounds can be used for various pharmaceutical applications [8] such as for the treatment of neurodegenerative and proliferative disorders. These compounds operate by inhibiting proteins or enzymes that regulate cell cycles, including cyclin-dependent kinases [9], tyrosine kinase [10], glycogen-synthase kinase and mitochondrial malate dehydrogenase [11]. These interesting biological activities led us to develop one-pot sequential or cascade protocols for the synthesis of [2,3-*b*]-, [2,3-*c*]- and [3,4-*b*]pyrrolo- and indoloazepines [12–20]. However, the yields of the pyrrolo- and indolo[3,2-*c*]azepines were relatively low. One of the most efficient procedures for preparing pyrrolo[3,2-*c*]azepin-4-ones involves the Beckmann rearrangement of 6,7-dihydroindol-4(5*H*)-ones. However, after implementing this approach, an undesirable isomer was obtained [21]. Although a number of acid- [22–23] and metal-catalysed cascade processes [24–25] have been developed (for example, Pictet–Spengler [26] and Ugi-type reactions [27], the acyl radical cyclisations [28] and the 1,7-electrocyclisation of azomethine ylides or ring expansion sequences [29]), most of these approaches are ineffective for synthesising pyrrolo[3,2-*c*]azepines. Recently, Echavarren and Beller reported a novel Au- or Pt-catalysed cycloisomerisation reaction of pyrrole-2-carboxamides that produces pyrrolo[2,3-*c*]- and [2,3-*d*]azepinones [30–31]. However, this reaction is not suitable for the synthesis of pyrrolo[3,2-*c*]azepines in viewpoint of the difficulties in preparing the starting materials.

**Scheme 1:**
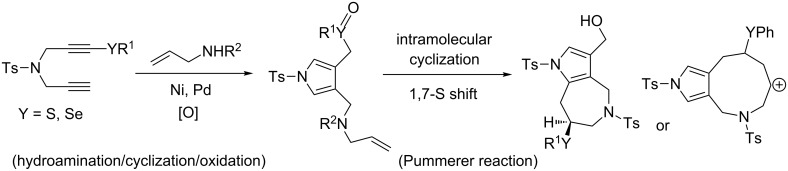
Our synthetic plan for pyrrolo[3,2-*c*]azepines.

We previously reported that the hydroamination/cyclisation reaction of sulfanyl-1,6-diynes with secondary amines resulted in the formation of 3-sulfanylmethyl-4-aminomethylpyrroles (Scheme 1) [32–33]. The sulfanyl group of pyrroles is easily oxidised to produce their corresponding sulfoxides. When we performed the Pummerer reaction and cyclization of pyrrolesulfoxides to obtain their corresponding pyrroloazepines, we observed unprecedented events, i.e., 1,7-sulfanyl group (1,7-S) shift reactions, on the cation intermediates. Herein, we report the unique 1,7-S and 1,7-Se shift reactions through Pummerer reactions of alkenyl pyrrolomethyl sulfoxides and the corresponding successive cyclisation reactions.

## Results and Discussion

**Scheme 2:**
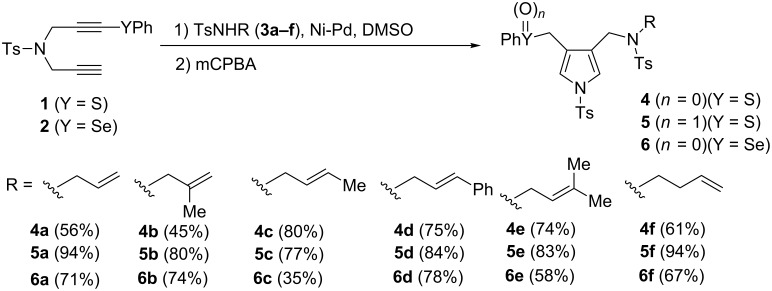
Preparation of precursors for the Pummerer reactions.

We prepared sulfoxides as substrates for the Pummerer reactions according to the aforementioned method [32–33]; subsequently, oxidation with mCPBA was performed (Scheme 2). Both the allylic sulfides **4a**–**f** and their sulfoxides **5a–f** were obtained in good to high yields. Corresponding pyrroloselenides **6a–f** were prepared by applying a similar synthetic sequence; however, the selenoxides could not be obtained by implementing the usual reaction conditions. The structure of the products derived from the amination–cyclisation reaction of sulfanyl 1,6-diyne **1a** was determined by analysing the single-crystal X-ray diffraction spectrum of the corresponding sulfone of **4a**; the structure was a pyrrole bearing both 3-sulfanylmethyl and 4-allylaminomethyl groups (see Supporting Information File 1).

To develop a useful Pummerer reaction procedure, we selected allylamine derivative **5a** as representative substrate and performed a screening to find suitable conditions for its reaction with anhydrides (Table 1). Since the reaction of **5a** with acetic anhydride/acetic acid completely stagnated at room temperature, we performed the same reaction at 120 °C. However, the reaction conditions did not yield the desired cyclic products and ester **7a** was instead produced in good yield (entry 1 in Table 1). We next examined the reaction of **5a** with 1.5 equiv of trifluoroacetic anhydride (TFAA) in dichloromethane at 0 °C; however, the reaction yielded a complex mixture (Table 1, entry 2). Since the reaction with trifluoromethanesulfonic anhydride resulted in the recovery of **5a** (Table 1, entry 3), we reinvestigated the reaction of **5a** with TFAA in detail. The use of an excess of TFAA, followed by the treatment with triethylamine yielded aldehyde **8a** instead of alcohol **7a** (Table 1, entry 4). These results show that the reaction conducted in the presence of an excess of TFAA was indeed successful and the Pummerer reaction products such as α-trifluoroacetoxysulfide underwent hydrolysis to yield aldehyde **8a**. In entries 5 and 6, we present the results from performing the reactions at lower temperatures. Fortunately, we found the formation of unprecedented product **9a**, which was contaminated with unknown products at both −20 °C and −40 °C. The Pummerer rearrangement and successive cyclisation with either the intramolecular C–C double bond or the pyrrole were unsuccessful; however, *N*-(3-hydroxy-2-(phenylthio)propyl)-*N*-((4-(hydroxymethyl)-1-tosyl-1*H*-pyrrol-3-yl)methyl)-4-methylbenzenesulfonamide (**9a**) was apparently obtained via the sulfanyl group migration (S-shift reaction) (refer the NMR studies of **9a** in Supporting Information File 1). To clarify the details of the S-shift reactions, we performed the usual Pummerer reaction, followed by treatment with bases at −20 °C (Table 1, entries 8–10). We determined that the *S*-shift reaction was strongly affected by the base. Tetrabutylammonium hydroxide was found to be the best base for the formation of **9a** (Table 1, entry 10). Neither trimethylsilyl trifluoromethanesulfonate nor oxallyl chloride/triethylamine proved effective for the formation of **9a** (Table 1, entries 11 and 12).

**Table 1:** Screening the reaction conditions for the Pummerer reactions.

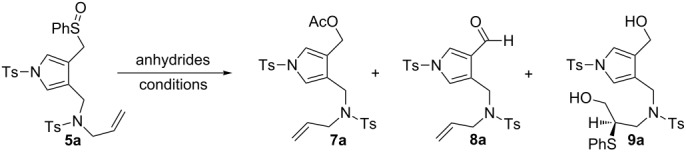								

entry	anhydride [equiv]	solvent	*T* [°C]	time [h]	**5a**	yields [%] **7a**	**8a**	**9a**

1	Ac_2_O (5)	AcOH	120	6		86	–	–
2	TFAA (1.5)	DCM	0	2	–	–	–	–
3	Tf_2_O (1.5)	MeCN	0	1		–	–	–
4	TFAA (8), Et_3_N (3)	DCM	0	1.3		–	36	–
5	TFAA (5)	DCM	−20	0.5		–	–	^–a^
6	TFAA (5)	DCE	−40	0.3		–	–	^–a^
7	TFAA (5)	THF	−20	0.5		–	86	–
8	TFAA (5), Bu_4_NOH aq	DCM	−20	2		–	–	67
9	TFAA (5), K_2_CO_3_ aq	DCM	−20	1.5		–	–	46
10	TFAA (5), Bu_4_NOH aq	DCM	−20	0.5		–	–	93
11	TMSOTf (3)	DCE	−20	0.7		–	–	–
12	(COCl)_2_ (5), Et_3_N (5)	DCE	0	0.3		–	74	18

^a^**9a** was obtained contaminated with traces of other unknown compounds.

**Scheme 3:**
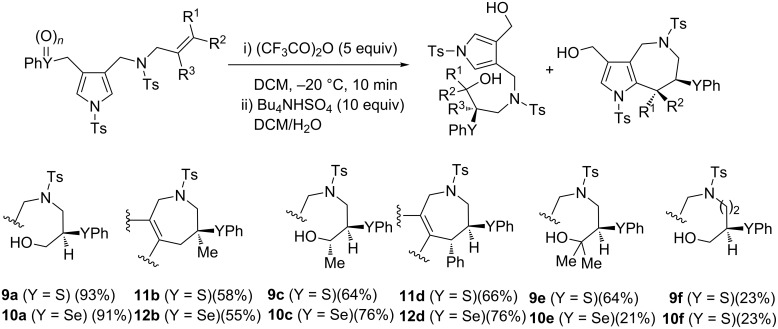
Substrate scope of 1,7-S and 1,7-Se shift reactions.

Although the synthesis of the desired pyrrolo[3,2-*c*]azepines was unsuccessful, we discovered the unique 1,7-S shift reactions. We next examined the scope of 1,7-S- and 1,7-Se-shift reactions under Pummerer reaction conditions of sulfoxides and the results are shown in Scheme 3. We first examined the following one-pot sequential reactions of the selenium analog: the mCPBA oxidation of *N*-allylpyrrolomethylselenide **6a**, the following Pummerer reaction, and final treatment with TBAH. The stepwise procedure succeeded to give 7-phenylselenodiol **10a** in 91% yield. Next, we performed the Pummerer reaction of *N*-β-methallyl sulfoxide **5b** in order to clarify the substituent effects on the *N*-alkenyl groups. Surprisingly, the reaction of **5b** afforded the intramolecular cyclised pyrroloazepine **11b**. The *N*-methallylselenopyrrole **6b** also gave the pyrroloazepine **10b**, which was formed via the 1,7-Se shift reaction. While *N*-3-methylbut-2-enyl derivatives **4c** and **6c** yielded 1,7-S- and Se-shifted diols **9c** and **10c**, respectively. Interestingly, the reaction of *N*-cinnamylpyrroles **4d** and **6d** exclusively afforded *trans*-azepines **11d** and **12d** via intramolecular cyclization. The same procedure applied to the bulky *N*-(3-methylbut-2-enyl) derivatives **4e** and **6e** yielded the corresponding diols **9e** and **10e**. Whether the Pummerer reactions yielded cyclised products or not was strongly affected by the substituent patterns. If the azepinium cation intermediates were stabilised by either α-methyl or β-phenyl substituents, the intramolecular cyclisation with the pyrrole ring proceeded to afford azepinoindoles. If both substituents stabilised the intermediates, *N*-allyl (*n* = 1; R^1^ = R^2^ = H), *N*-2-butenyl (*n* = 1; R^1^ = H, R^2^ = Me) and *N*-3-methyl-2-butenyl derivatives (*n* = 1; R^1^ = R^2^ = Me) exclusively gave the diols **9a**, **9c**, **9e**, **10a**, **10c** and **10e**. In particular, all products were obtained through 1,7-S- or 1,7-Se shift reactions. The yields of the Pummerer reactions of both *N*-3-butenyl derivatives **9f** and **10f** were very low. These products were confirmed by X-ray spectra and/or single-crystal X-ray diffraction patterns.

**Scheme 4:**
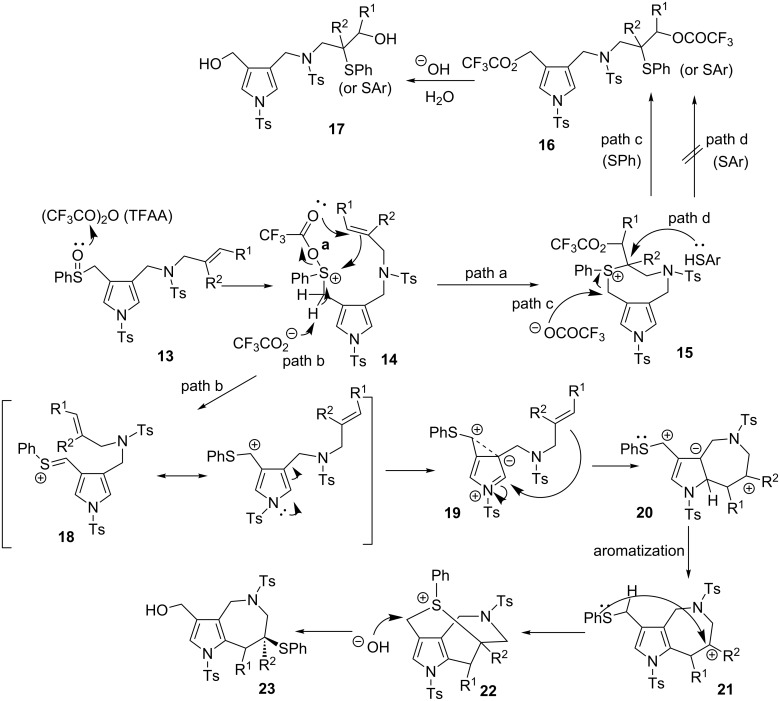
Proposed mechanism.

**Scheme 5:**
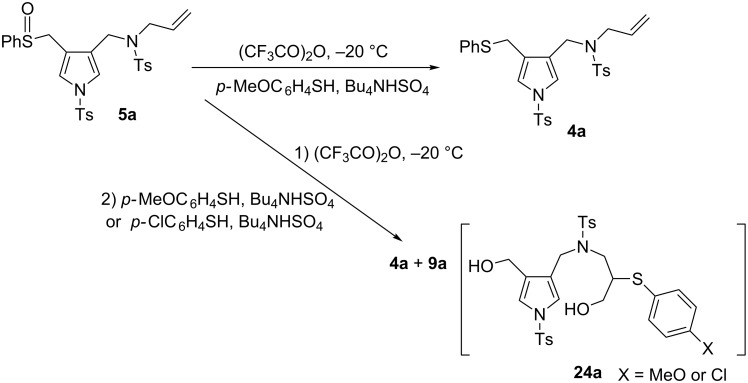
Crossover experiment.

The unique S-shift reactions in the formation of either pyrrolo[3,2-*c*]azepines or diols proceeded according to the mechanism we proposed, which is depicted in Scheme 4. Sulfoxide **13** is activated by TFAA to form sulfonium salt **14**, which is characterised by an intramolecular double bond [34–35]. A less substituted double bond easily approaches the sulfonium ion and undergoes an addition reaction to produce intermediate **15**. The trifluoroacetate anion attacks the posterior side of the α-carbon of the sulfur atom. Bis(trifluoro)acetate **16** resulted from the opening of the ring is hydrolysed by TBAH to yield diol **17**. Conversely, sterically hindered double bonds of **14** are not easily accessible to the sulfonium ion; therefore, the Pummerer intermediate α-thio carbenium ion **18** is generated through detrifluoroacetoxylation of **14** (path b: normal Pummerer reactions) and undergoes intramolecular cyclisation on the pyrrole ring of **19**. Aromatisation of dihydropyrrole intermediate **20** leads to the formation of pyrroloazepinic cation **21**, which is stabilised by the phenylsulfanyl group at its 3-position. The S-shift reaction involves the transannular sulfonium intermediate **22**, which is attacked by a hydroxide anion from the *anti*-direction. Both products, diols and azepinopyrroles, are obtained in a stereoselective manner. In order to confirm the reaction mechanism, we performed crossover experiments with arylthiol (Scheme 5). First, we examined the Pummerer reactions in the presence of *p*-methoxybenzenethiol or *p*-chlorobenzenethiol; however, the reactions yielded the reductive sulfide **4a**. We next examined the stepwise process: i) the Pummerer reaction of **5a** with TFAA, followed by ii) the usual work-up and successive treatment with *p*-methoxybenzenethiol/TBAH, which exclusively produced **9a** (the diol **24a** was not detected by ESI mass spectroscopy). A similar result was obtained from the reaction of **5a** with *p*-chlorobenzenethiol/TBAH. These results support the hypothesis that the reaction proceeds via the initial formation of the key intermediate **15**, of which the sulfanyl group would intramolecularly migrate to produce **16** stereoselectively (path c, not d). The hydrolysis of **16** using TBAH yields diol **17**. We further confirmed the mechanism of the 1,7-S shift reaction by performing density functional theory (DFT)-based calculations (Table 2) [36]. Whether the reactions lead to the formation of pyrroloazepines or diols clearly depends on the substituents in the alkenyl group. We performed DFT calculations for the reaction pathways starting from the key intermediate **14** bearing methyl and phenyl groups. The DFT-calculated Δ*G* values of **15a** is by 24 kcal/mol lower than that of **21a** (R^1^ = R^2^ = H), which is in accordance with the experimental result that the reaction of **4a** leads to the formation of diol **9a**. The calculated results related to **15b**,**c** were also found to match the experimental data. The S-shift process of pyrrolo[3,2-*c*]azepinium cation **21x** enables the reaction to proceed via an intramolecular 1,7-S shift, followed by a hydroxide attack on **22** (relevant details are reported in Supporting Information File 1).

**Table 2:** DFT Calculation of the two pathways: from **14** to **15x** or from **14** to **21x**.

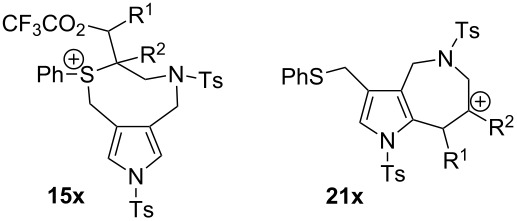			

R^1^	R^2^	15x (Δ*G* kcal/mol)	**21x** (Δ*G*)

H	H	**15a** (−27.16)	**21a** (−3.17)
Me	H	**15b** (−28.25)	**21b** (−16.55)
H	Me	**15c** (−12.82)	**21c** (−26.02)
Ph	H	**15d** (−18.59)	**21d** (−24.51)

**Scheme 6:**
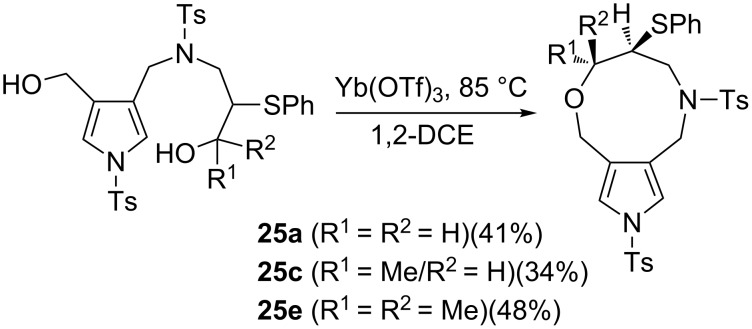
Lewis acid-catalysed cyclization of diols.

**Scheme 7:**
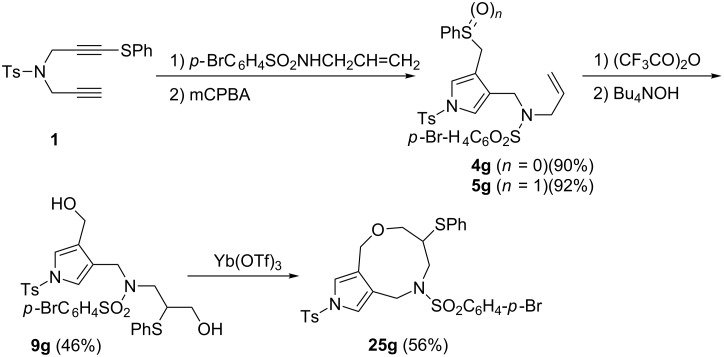
Sequential process of sulfanyl-1,6-diyne **1** to 4*H*-pyrrolo[3,4-*g*]oxazine **25g**.

We further investigated the cyclisation of diols to synthesise pyrrolo[3,2-c]azepines (Scheme 6). However, the products obtained were not the desired pyrrolo[3,2-*c*]azepines but were 2,6,7,8,9,10-hexahydro-4*H*-pyrrolo[3,4-*g*][1,5]oxazonines **25a**–**e**, which were formed via a reaction route that involved the intramolecular dehydration of diols. To confirm the structure of 4*H*-pyrrolo[3,4-*g*]oxazines, we prepared an *N*-bromobenzenesulfonyl derivative, which was expected to act as a good substrate for the isolation of crystals, and performed the same sequential process of sulfanyl-1,6-diynes to the oxazine derivatives (Scheme 7). Hydroamination–cyclisation of diyne **1** with *N*-allyl-*p*-bromobenzenesulfonamide **3g** and the successive oxidation proceeded to give the corresponding sulfoxide **5g** in high yield. The sequential process, Pummerer reaction of **5g** with TFAA, followed by treatment with TBAH to afford the 1,7-sulfur-shifted product **9g** as crystals; however, the yield was relatively low. The final ytterbium-catalysed intramolecular cyclisation afforded **25g** with a 56% yield. We obtained the 4*H*-pyrrolo[3,4-*g*]oxazine derivative as crystals. The structure of these products was determined through NMR spectroscopy experiments (COSY and HMBC data are reported and discussed in Supporting Information File 1).

## Conclusion

We found unique 1,7-S and 1,7-Se shift reactions by investigating the synthesis of pyrroloazepines through amination–cyclisation of 1,6-diynes, followed by the Pummerer reaction and TBAH hydrolysis. We are now investigating the syntheses of pyrroloazepines using 4-heteroarylmethyl-3-sulfanylpyrroles, which could be easily obtained by hydroamination–cyclization of sulfanyl-1,6-diynes/the successive cyclization. The results of this investigation will be reported elsewhere.

## Supporting Information

For crystallographic data see also CCDC 1824587 and 1824588.

File 1 Schemes S1 and S2 on the syntheses of compounds **1**, **2**, and **3a–g**; the NMR study for the structure determinations, further DFT calculations, the ORTEP drawing of both sulfone of **5a**, **11d** and the ^1^H and ^13^C NMR charts.

File 2 Crystallographic information of compound **11d**.

File 3 Crystallographic information of the sulfone of compound **5a**.
