# Supplementary material for: Unprecedented nucleophile-promoted 1,7-S or Se shift reactions under Pummerer reaction conditions of 4-alkenyl-3-sulfinylmethylpyrroles
Source: Beilstein J Org Chem. 2018 Oct 29;14:2722–9. doi: 10.3762/bjoc.14.250 (PMC6244179; doi:10.3762/bjoc.14.250)
Supplement: File 1 — Schemes S1 and S2 on the syntheses of compounds 1, 2, and 3a–g; the NMR study for the structure determinations, further DFT calculations, the ORTEP drawing of both sulfone of 5a, 11d and the 1H and 13C NMR charts. [file Beilstein_J_Org_Chem-14-2722-s001.pdf]

Supporting Information

for

Unprecedented nucleophile-promoted 1,7-S- or Se-shift reactions under Pummerer reactions of 4-alkenyl-3-sulfinylmethylpyrroles

Takashi Go[1], Akane Morimatsu[1], Hiroaki Wasada[2], Genzoh Tanabe[3], Osamu Muraoka[3], Yoshiharu Sawada[4] and Mitsuhiro Yoshimatsu[*1]

Address: [1]Department of Chemistry, Faculty of Education, Gifu University, Yanagido 1-1, Gifu 501-1193, Japan, [2]Department of Chemistry, Faculty of Regional Study, Gifu University, Yanagido 1-1, Gifu 501-1193, Japan, [3]School of Pharmacy, Kindai University, 3-4-1 Kowakae, Higashi-osaka, Osaka 577-8502, Japan and [4]Life Science Research Center, Gifu University, Yanagido 1-1, Gifu 501-1193, Japan

Email: Mitsuhiro Yoshimatsu - yoshimae@gifu-u.ac.jp

*Corresponding author

Schemes S1 and S2 on the syntheses of compounds 1, 2, and 3a–g; the NMR study for the structure determinations, further DFT calculations, the ORTEP drawing of both sulfone of 5a, 11d and the ^{1}H and ^{13}C NMR charts

CONTENTS

1. Scheme S1 and S2 for the preparations of allylsulfones 3 S3

2. NMR study for the structure determinations S2

3. Sample preparation and crystal structure determination of compound 12d and sulfone of 5a S8

4. DFT calculations S13

5. Experimental S16

6. X-ray crystallographic analysis S63

7. References S65

8. DFT computational results, Cartesian coordinates, computed total energies of optimized structures S66

9. ^{1}H and ^{13}C NMR harts S177

1. Preparations of *N*-allyl sulfonamides 3a–f.

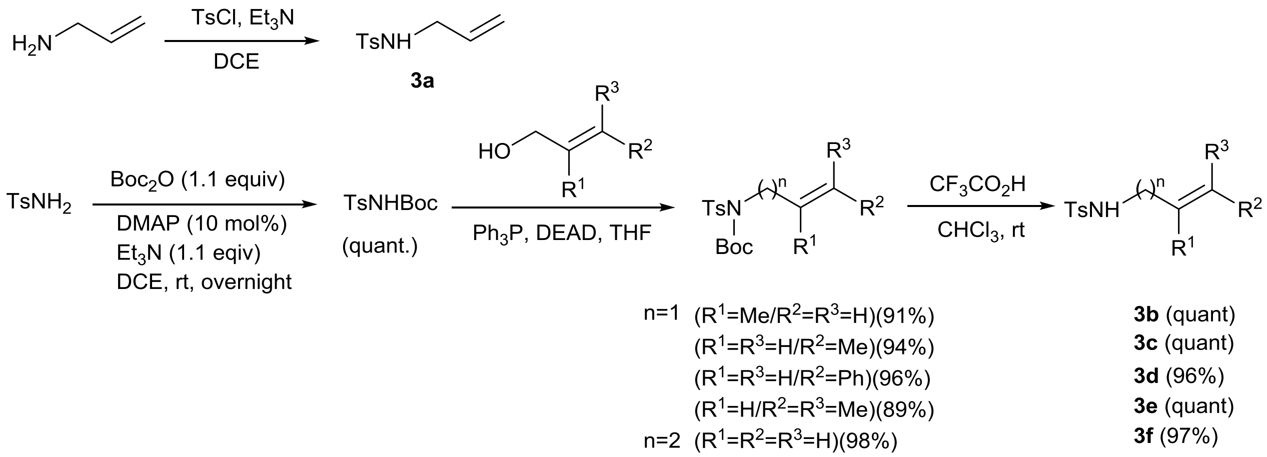

Scheme S1: Preparations of sulfonamides 3.

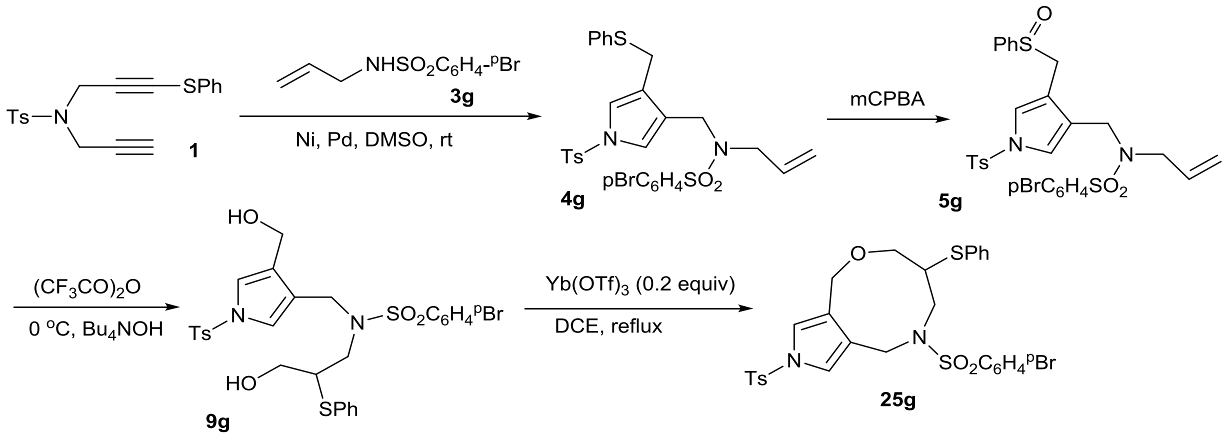

Scheme S2: Synthesis and ytterbium-catalyzed reaction of 9g.

2. NMR study for the structure determinations.

N-(3-Hydroxy-2-(phenylselanyl)propyl)-*N*-((4-(hydroxymethyl)-1-tosyl-1*H*-pyrrol-3-yl)methyl)-4-methylbenzenesulfonamide (10a).

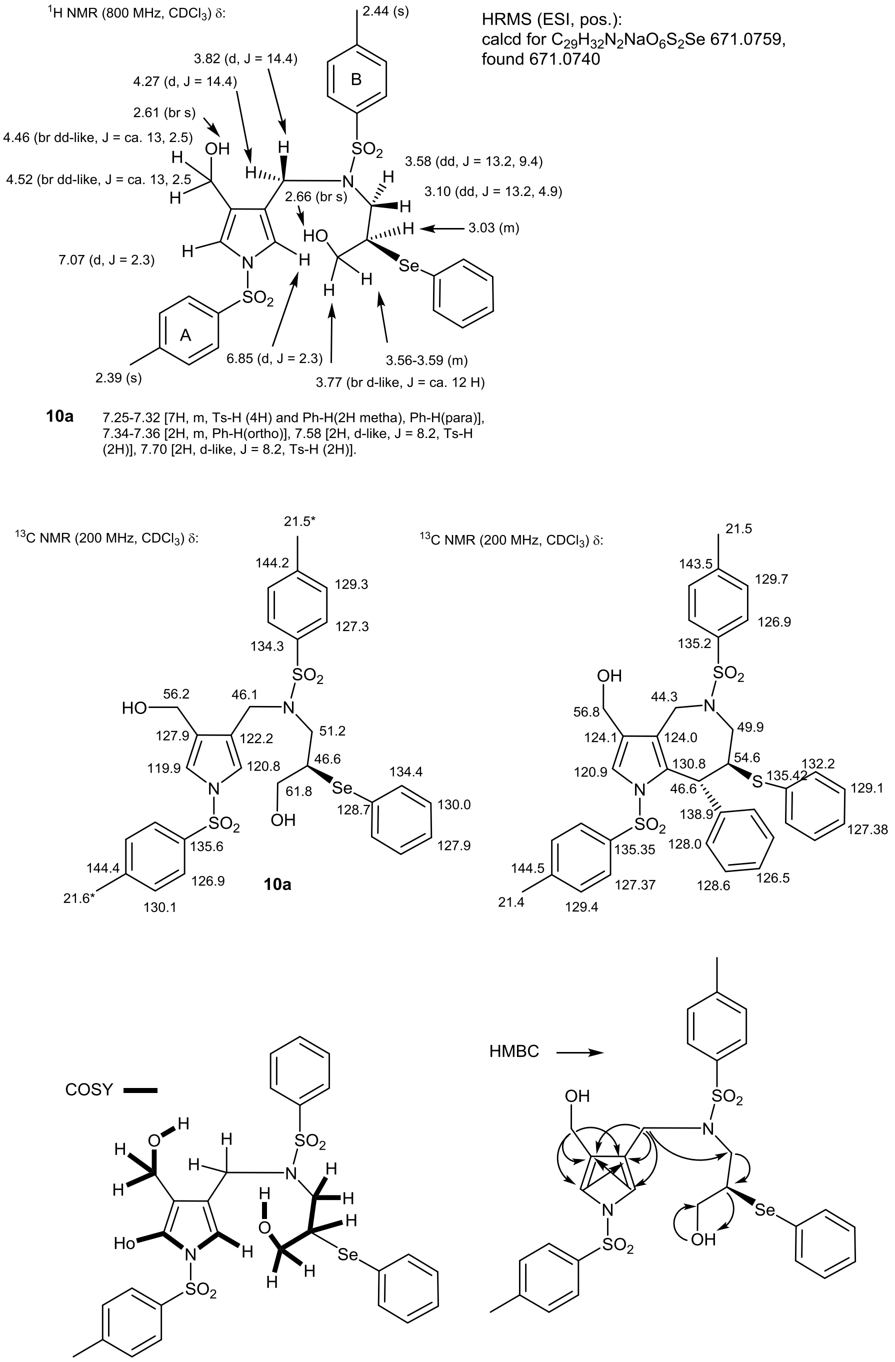

Fig S1: NMR study of 10a in $CDCl_3$.

1,5-Bis(*p*-toluenesulfonyl)-1,4,5,6,7,8-hexahydro-8-phenyl-7-(phenylthio)pyrrolo[3,2-c]-azepin-3-methanol (**11d**).

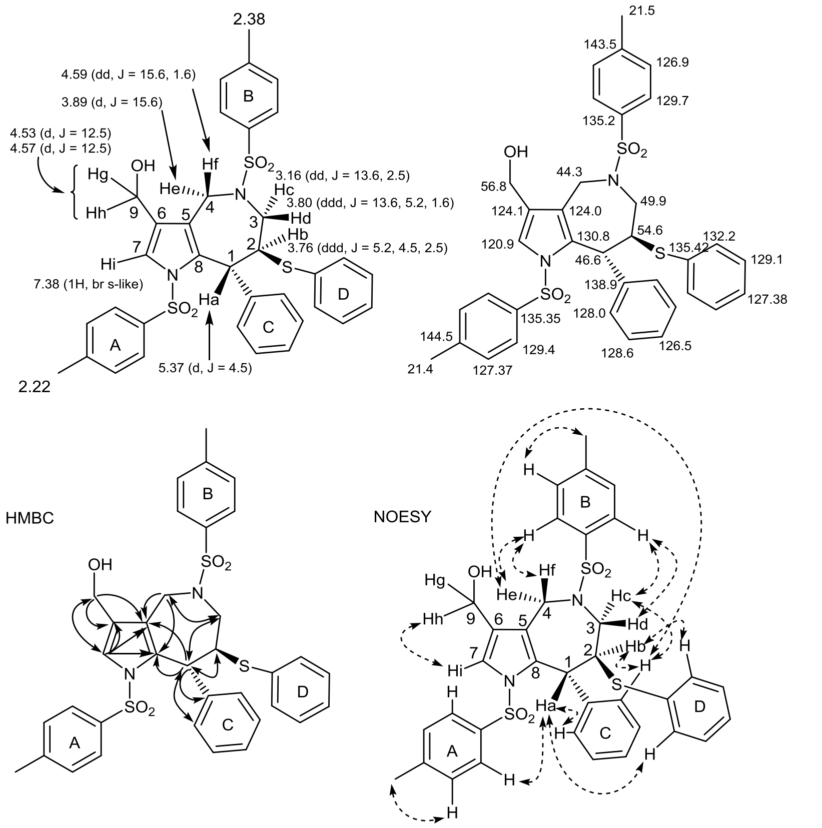

Fig S2: NMR study of 11d in $CDCl_3$.

^{1}H NMR (800 MHz, $CDCl_3$) δ 2.22 (3H, s, Ts(A)CH_3), 2.38 (3H, s, Ts(B)CH_3), 3.16 (1H, dd, *J* = 13.6, 2.5 Hz, Hc [quasi-ax]), 3.76 (1H, ddd, J = 5.2, 4.5, 2.5 Hz, Hb), 3.80 (1H, ddd, *J* = 13.6, 5.2, 1.6 Hz, Hd [quasi-eq]), 3.89 (1H, d, *J* = 15.6, He [quasi-ax]), 4.53/4.57 (each 1H, d, *J* = 12.5, Hg and Hh), 4.59 (1H, dd, *J* = 15.6, 1.6, Hf [quasi-eq]), 5.37 (1H, d, *J* = 4.5, Ha), 6.74 (2H, dm, *J* = ca. 7.4 Hz, Ph(C)-ortho), 6.86 (2H, dm, *J* = ca. 8.4 Hz, Ts(A)-meta), 7.07 (2H, tm, *J* = ca. 7.4 Hz, Ph(C)-meta), 7.10 (1H, tm-like, *J* = ca. 7.4, Ph(C)-para), 7.24 (2H, m, Ts(A)-ortho and Ts(B)-metha), 7.30 (1H, tm, *J* = ca. 7.4, Ph(D)-para), 7.35 (2H, dm, *J* = ca. 7.4 Hz, Ph(C)-meta), 7.38 (1H, br s-like, Hi), 7.46 (2H, dm, *J* = ca. 7.4 Hz, Ph(D)-ortho), 7.63 (2H, dm, *J* = ca. 8.4 Hz, Ts(S)-ortho). ^{13}C NMR (200 MHz, $CDCl_3$) δ: 21.4/21.5 (Ts(B)CH_3/Ts(A)CH_3), 44.3 (C-4), 46.6 (C-1), 49.9 (C-3), 54.6 (C-2), 56.8 (C-9), 120.9 (C-7), 124.0 (C-5), 124.1 (C-6), 126.5 (Ph(C)-para), 126.9

(Ts(A)-meta), 127.37 (Ts(B)-meta), 127.38 (Ph(D)-para), 128.0 (Ph(C)-ortho), 128.6 (Ph(C)-meta), 129.1 (Ph(D)-meta), 129.4 (Ts(A)-ortho), 129.7 (Ts(B)-ortho), 130.8 (C-8), 132.2 (Ph(D)-ortho), 135.2 (Ts(A)-ipso), 135.35 (Ts(B)-ipso), 135.42 (Ph(D)-ipso), 138.9 (Ph(C)-ipso), 143.5 (Ts(B)-ipso), 144.5 (Ts(A)-ipso).

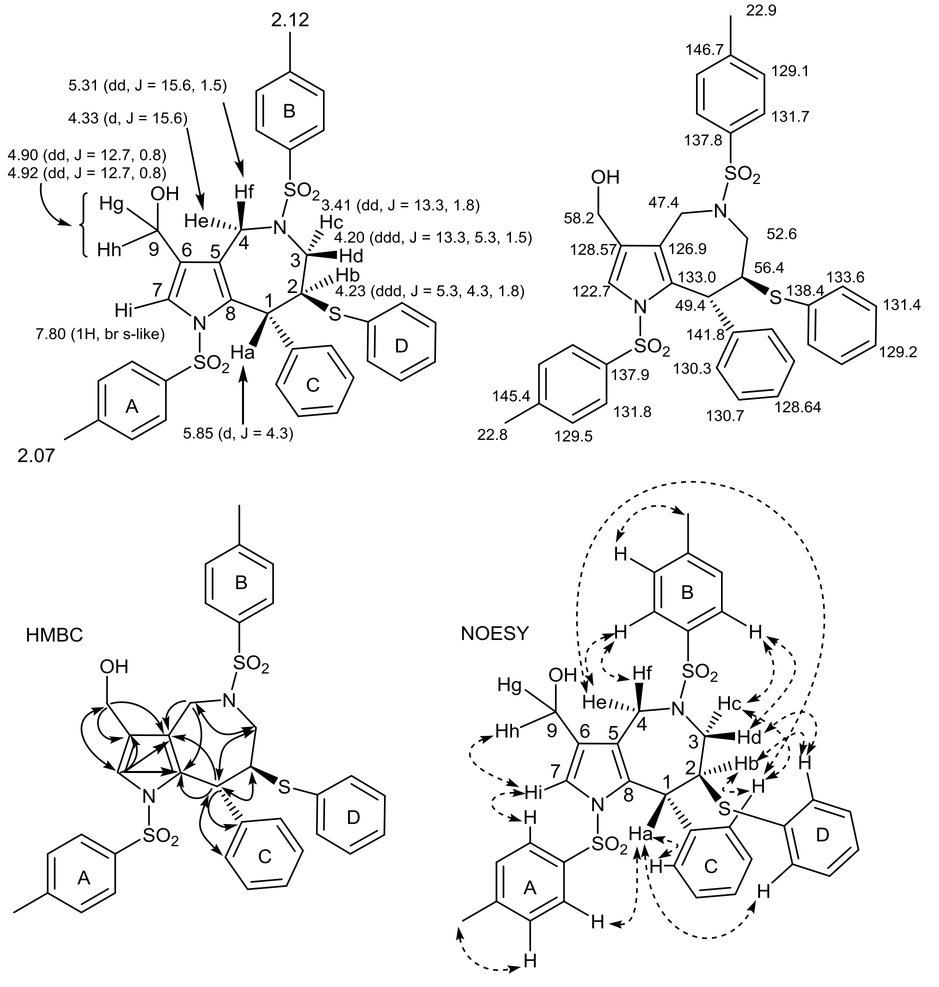

Fig S3: NMR study of 11d in Pyridine D_5.

^{1}H NMR (800 MHz, pyridine-d_5) δ 2.07 (3H, s, Ts(A)CH_3), 2.12 (3H, s, Ts(B)CH_3), 3.41 (1H, dd, J = 13.3, 1.8 Hz, Hc [quasi-ax]), 4.20 (1H, ddd, J = 13.3, 5.3, 1.5 Hz, Hd [quasi-eq]), 4.23 (1H, ddd, J = 5.3, 4.3, 1.8 Hz, Hb), 4.33 (1H, d, J = 15.6, He [quasi-ax]), 4.90/4.92 (each 1H, dd, J = 12.7, 0.8, Hg and Hh), 5.31 (1H, dd, J = 15.6, 1.5, Hf [quasi-eq]), 5.85 (1H, d, J = 4.3, Ha), 6.84 (2H, dm, J = ca. 8.4 Hz, Ts(A)-meta), 7.04 (2H, dm, J = ca. 7.4 Hz, Ph(C)-ortho), 7.06 (2H, tm, J = ca. 7.4 Hz, Ph(C)-meta), 7.08 (1H,

tm-like, J = ca. 7.4, Ph(C)-para), 7.10 (1H, dm, J = 8.4, Ts(B)-metha), 7.30 (1H, tm, J = ca. 7.4, Ph(D)-para), 7.40 (2H, dm, J = ca. 7.4 Hz, Ph(C)-meta), 7.49 (2H, dm, J = ca. 8.4 Hz, Ts(A)-ortho), 7.69 (2H, dm, J = ca. 7.4 Hz, Ph(D)-ortho), 7.80 (1H, br s-like, Hi), 7.84 (2H, dm, J = ca. 8.4 Hz, Ts(S)-ortho); ^{13}C NMR (200 MHz, pyridine-d_5) δ: 22.8/22.9 (Ts(B)CH_3/Ts(A)CH_3), 47.4 (C-4), 49.4 (C-1), 52.6 (C-3), 56.4 (C-2), 58.2 (C-9), 122.7 (C-7), 126.9 (C-5), 128.57 (C-6), 128.64 (Ph(C)-para), 129.1 (Ts(A)-meta), 129.2 (Ph(D)-para), 129.5 (Ts(B)-meta), 130.3 (Ph(C)-ortho), 130.7 (Ph(C)-meta), 131.4 (Ph(D)-meta), 131.7 (Ts(A)-ortho), 131.8 (Ts(B)-ortho), 133.0 (C-8), 133.6 (Ph(D)-ortho), 137.8 (Ts(A)-ipso), 137.9 (Ts(B)-ipso), 138.4 (Ph(D)-ipso), 141.8 (Ph(C)-ipso), 145.4 (Ts(B)-ipso), 146.7 (Ts(A)-ipso).

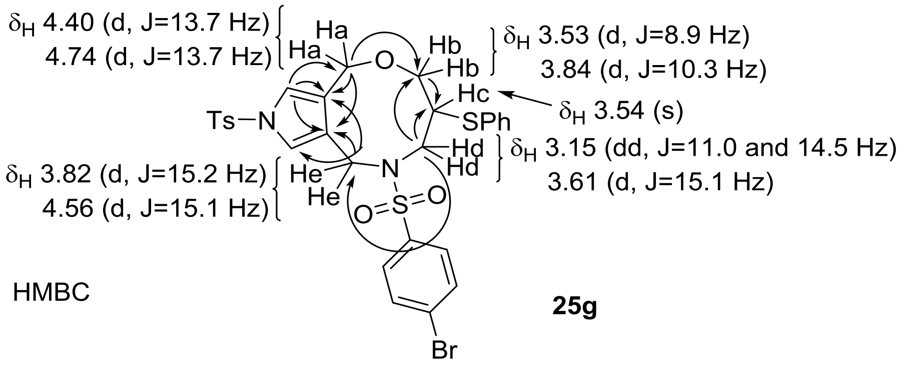

25g

Fig S4: NMR study of 25g in $CDCl_3$.

3. Sample preparation and crystal structure determination of compound 11d

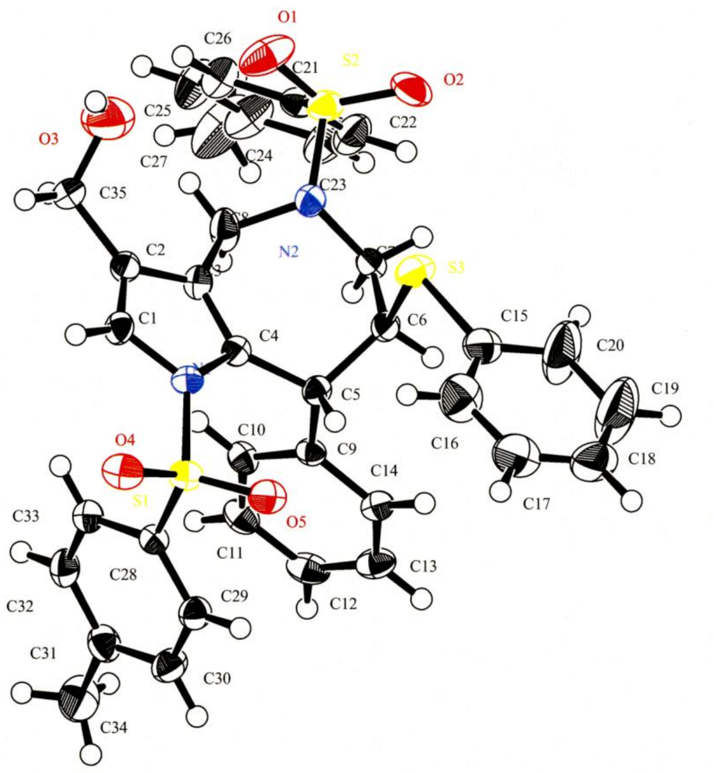

Perspective drawing of compound **11d**, showing the atom-numbering scheme. Displacement ellipsoids are drawn at 50% probability level.

EXPERIMENTAL DETAILS

Sample preparation of compound 11d: Colorless prism-shaped single crystals were obtained by slow evaporation of a solution of **11d** in *n*-hexane/acetone.

Crystal data and structure refinement for compound 11d (CCDC 1824588)

Crystal data

Empirical Formula	$C_{35}H_{34}N_2O_5S_3$
Formula Weight	658.84
Crystal Dimensions	0.30 X 0.20 X 0.10 mm
Temperature	123 K

Crystal System	tetragonal
Space Group	$P4_1$
Lattice Parameters	a = 14.8202(6) Å
	c = 14.7217(6) Å
Volume	3233.4(2) $Å^3$
Z value	4
Dcalc	1.353 g/cm^3
F000	1384.00
μ(MoKα)	2.746 cm^{-1}

Intensity Measurements

Diffractometer	Rigaku RAXIS-RAPID
Radiation	MoKα (λ = 0.71075 Å)
	graphite monochromated
Detector Aperture	280 mm x 256 mm
Data Images	44 exposures
ω oscillation Range (χ=45.0, ϕ=90.0)	130.0 - 190.0°
Exposure Rate	60.0 sec./°
ω oscillation Range (χ=45.0, ϕ=270.0)	0.0 - 160.0°
Exposure Rate	60.0 sec./°
2θmax	54.9°
No. of Reflections Measured	Total: 30943
	Unique: 7244 (Rint = 0.024)

Structure Solution and Refinement

Structure Solution	Direct Methods (SIR97)
Refinement	Full-matrix least-squares on F
Function Minimized	Σ w (|Fo| - |Fc|)^2
Least Squares Weights	$1/[0.0010Fo^2+3.0000\sigma(Fo2)+0.5000]$
2θmax cutoff	54.9°
Anomalous Dispersion	All non-hydrogen atoms

No. Observations (All reflections)	30945
No. Variables	440
Reflection/Parameter Ratio	70.33
Residuals: R (I>2.00σ(I))	0.0454
Residuals: R (All reflections)	0.0504
Residuals: Rw (All reflections)	0.0564
Goodness of Fit Indicator	0.919
Max Shift/Error in Final Cycle	0.000
Maximum peak in Final Diff. Map	13.10 $e^{-}/Å^3$
Minimum peak in Final Diff. Map	–3.66 $e^{-}/Å^3$

Sample preparation and crystal structure determination of sulfone of 5a.

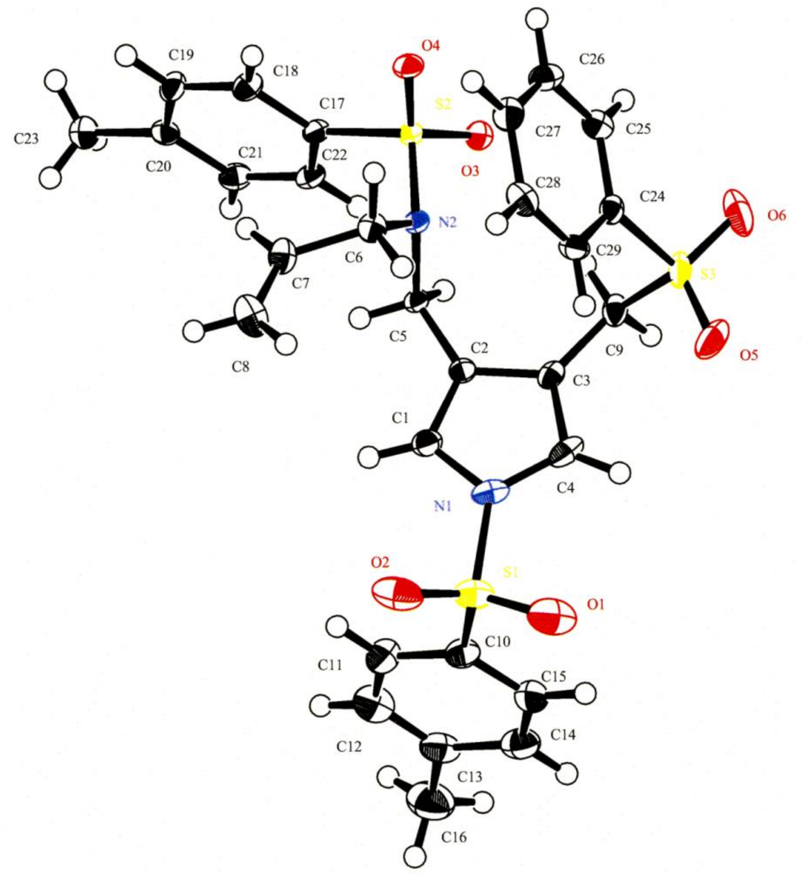

Perspective drawing of sulfone of **5a**, showing the atom-numbering scheme. Displacement ellipsoids are drawn at 50% probability level.

EXPERIMENTAL DETAILS

Sample preparation of sulfone of 5a: Colorless prism-shaped single crystals were obtained by slow evaporation of a solution of sulfone of **5a** in $CHCl_3$/MeOH**.**

Crystal data and structure refinement for sulfone of 5a (CCDC 1824587)

Crystal data

Empirical Formula	$C_{29}H_{30}N_2O_6S_3$
Formula Weight	598.75
Crystal Dimensions	0.25 X 0.20 X 0.20 mm
Temperature	123 K
Crystal System	monoclinic
Lattice Parameters	a = 8.8310(3) Å
	b = 13.1020(5) Å
	c = 25.1669(11) Å
	β = 91.4836(13)°
Volume	2910.9(2) Å^3
Z value	4
D_{calc}	1.366 g/cm^3
F_{000}	1256.00
μ(MoKα)	2.997 cm^{-1}

Intensity Measurements

Diffractometer	Rigaku RAXIS-RAPID
Radiation	MoKα (λ = 0.71075 Å)
	graphite monochromated
Detector Aperture	280 mm x 256 mm

Data Images	44 exposures
ω oscillation Range (χ=45.0, ϕ=0.0)	130.0 - 190.0°
Exposure Rate	60.0 sec./ °
ω oscillation Range (χ=45.0, ϕ=180.0)	0.0 - 160.0°
Exposure Rate	60.0 sec./°
$2\theta_{max}$	55.0°
No. of Reflections Measured	Total: 27881
	Unique: 6657 (R_{int} = 0.018)

Structure Solution and Refinement

Structure Solution	Direct Methods (SIR92)
Refinement	Full-matrix least-squares on F^2
Function Minimized	Σ w $(Fo^2 - Fc^2)^2$
Least Squares Weights	$1/[0.0010Fo^2+3.0000\sigma(Fo^2)+0.5000]/(4Fo^2)$
$2\theta_{max}$ cutoff	55.0°
Anomalous Dispersion	All non-hydrogen atoms
No. Observations (All reflections)	27881
No. Variables	391
Reflection/Parameter Ratio	71.31
Residuals: R1 ($I>2.00\sigma(I)$)	0.0748
Residuals: R (All reflections)	0.0855
Residuals: wR2 (All reflections)	0.1797
Goodness of Fit Indicator	1.474
Max Shift/Error in Final Cycle	0.000
Maximum peak in Final Diff. Map	17.30 $e^-/Å^3$
Minimum peak in Final Diff. Map	–4.66 $e^-/Å^3$

4. DFT calculations

We performed geometry optimization calculations for all compounds (**14a–d, 15a,d, and 21a,d**) at the B3LYP/6-31G(d) level. Frequency calculations were also preformed to confirm whether the optimized structures were energy minimum structures on the potential energy surface. All DFT calculations were performed with the Gaussian16,[S1] Revision A.03 suite program installed on the Fujitsu PRIMERGY CX400/2550 computer system at the Information Technology Center of Nagoya University.

The energy for each process of both from the sulfonium intermediate **14x** to the thioacetylated intermediate **15x** and from **14x** to azepinium cation **21x** were exhibited in Fig. S6. The lower energy of the intermediate **15a** of the reaction of *N*-allylsulfonium intermediate **14a** would lead to the formation of diols by treatment with tetrabutylammonium hydroxide (TBAH). On the other hand, the energy of intermediate **21b** is lower than that of **15b** in the reaction of *N*-methallyl sulfonium salt **14b** because of the stabilizing effect of methyl group on the azepinium cation. The azepinium cation **21b** exclusively undergo intra- or intermolecular 1,7-S shift reaction to give the 7-(phenylsulfanyl)pyrrolo[3,2-*c*]azepin-3-methanol. These calculation data are in good agreement with the experimental results.

The similar tendency was observed in the calculation of the Pummerer reaction of *N*-2-butenyl **14c** and *N*-cinnamyl derivatives **14d**.

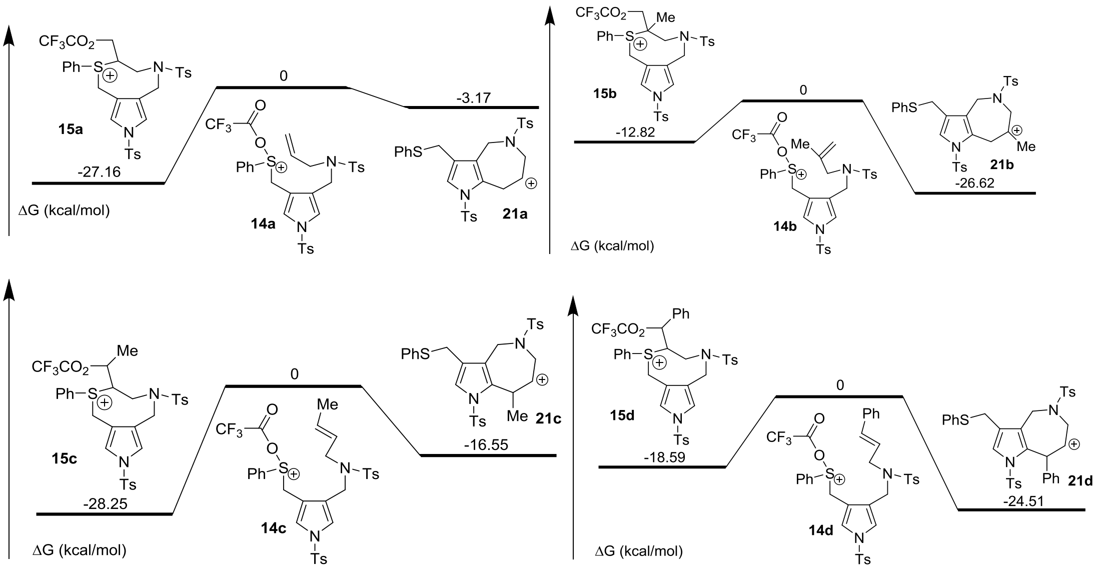

Fig S6: Comparing some pathways derived from the Pummerer intermediate 14.

We further investigated the 1,7-S shift reaction from the azepinium cation **21b** to the corresponding alcohol. We first predict that the intramolecular 1,7-S shift could occur via the transannular sulfonium intermediate **22b** to form the S-shifted pyrroloazepine **23b** because the 1,7-S shift reaction proceeded with high diastereoselectivity (**11b, 12b, 11d, 12d**). The calculation data also supported the intramolecular 1,7-S shift reaction as shown in Fig. S7. The speculation that the diol **17a** could be formed by the base-promoted hydrolysis of bis(trifluoroacetate) **16a** was ruled out by the DFT-calculation as shown in Fig S8.

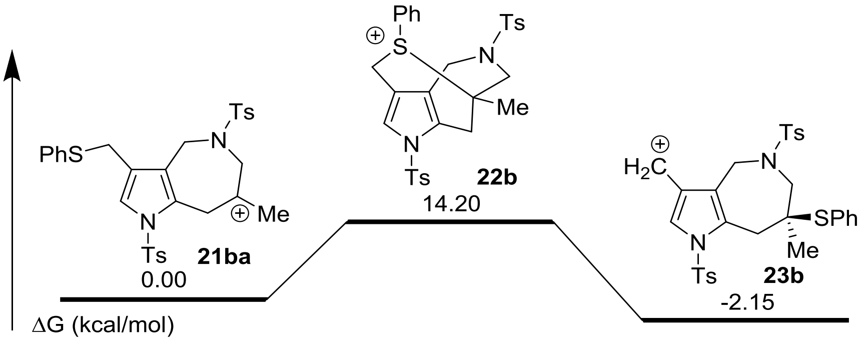

Fig S7: 1,7- Sulfur shift on the pyrrolo[2,3-c]azepinium cations 21b.

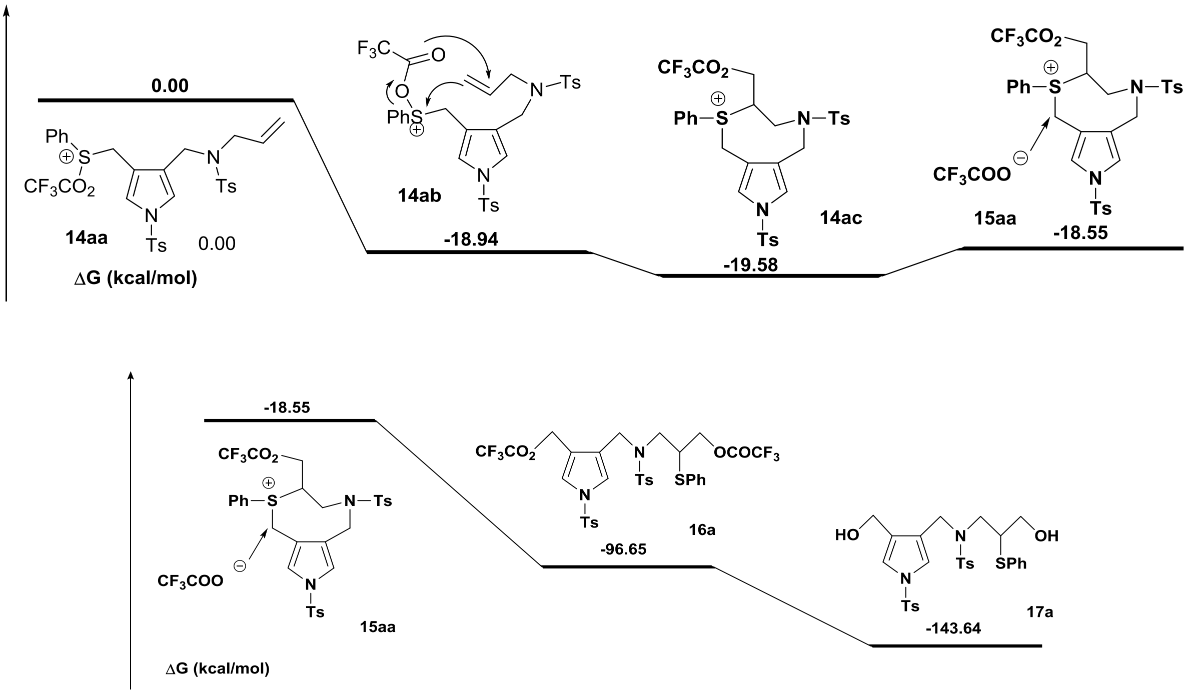

Fig S8: DFT calculation of 14a to the diol 17a.

Finally, we selected both *N*-allyl and *N*-methalyl derivatives and performed the DFT calculations in the processes from the key intermediates **14x** to the final products. The results are shown in Fig S6 and S8, respectively. The energy of *N*-allylsulfonium trifluoroacetate **14aa** is lowered at −18.94 kcal/mol by moving both the double bond of the *N*-allyl group and the trifluoroacetoxy group to give the cyclic intermediate **14ab**. The attack of the trifluoroacetoxy anion to the cyclic intermediate **15a** would occur from the back side of the α-carbon of the sulfur atom. Treatment of labile bis(trifluoroacetoxy)pyrrole **16a** with TBAH would provide the diol **17a**. We also calculated the azepine formation from the sulfonium intermediate **14b** (Fig. S9). The thionium ion (α-sulfur-substituted carbenium ion) **18ba**, which was generated from the normal Pummerer reaction, could be stabilized by the bridged intermediate **19b** (estimated at −5.05 kcal/mol by the DFT calculations), easily undergo intramolecular cyclization to give **20b**. The 1,5-hydride shift reaction of **20b** forms the azepinium cation **21b**. The final 1,7-S shift reaction have been already described above.

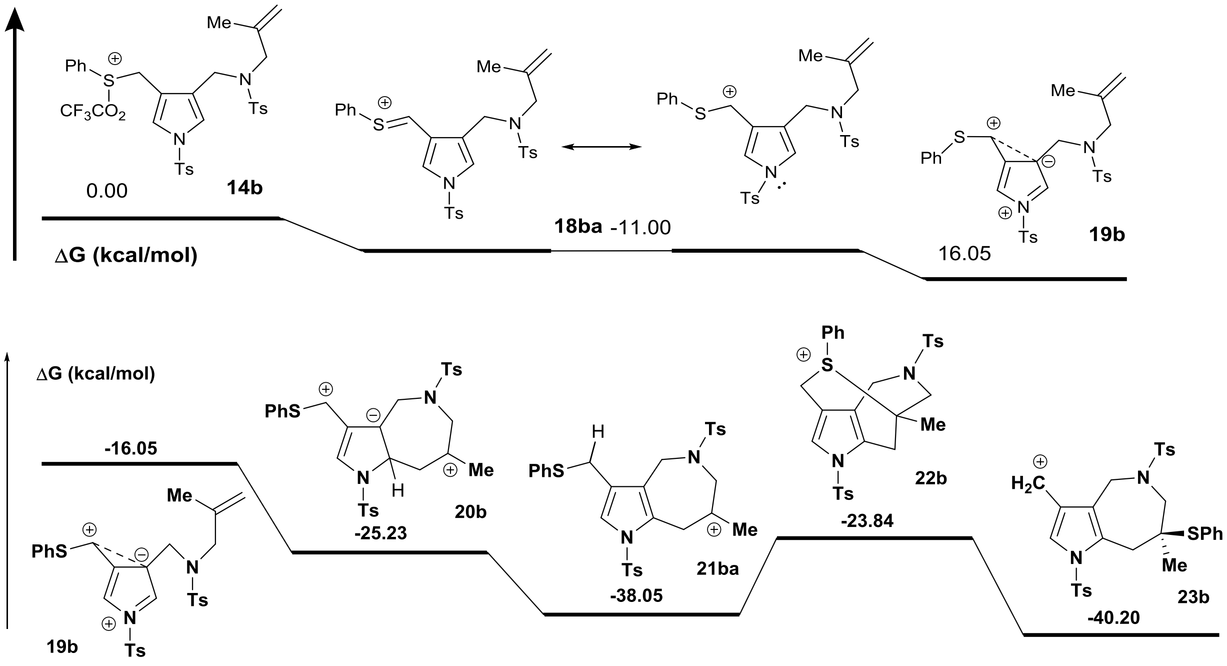

Fig S9: DFT calculation of 14b to the azepinopyrrole intermediate 23b.

5. Experimental

General experimental methods.

Analytical thin layer chromatography (TLC) was performed using silica gel precorted glass plates and visualized by ultraviolet radiation (254 nm). Flash column chromatography on silica gel was performed using silica gel (particle size 0.063–0.200 mm) under air pressure. Melting points were determined and uncorrected. ^{1}H and ^{13}C NMR spectra were determined with 600 MHz spectrometer. Chemical shifts are expressed in parts per million (ppm) with respect to tetramethylsilane as an internal standard. Splitting patterns are designated as follows: s, singlet; d, doublet; t, triplet; q, quartet. IR spectra were determined on a FT-IR infrared spectrometer and are expressed in reciprocal centimeters. EI mass spectra (MS) were obtained with direct-insertion probe at 70 eV. ESI measurements and their high resolution mass were performed using Quadrupole and TOF system.

Preparations of sulfanyl-**1** and selanyl diynes **2** and *N*-alkenylsulfonamides **3a–f.**

N-(Phenylsulfanylprop-2-ynyl)-*N*-prop-2-ynyl-*p*-toluenesulfonamide (**1**) and *N*-(phenylselanylprop-2-ynyl)-*N*-prop-2-ynyl-*p*-toluenesulfonamide (**2**) were prepared according to our previous reports.[S2,S3] The substrates in the hydroamination of 1,6-diynes were prepared by the usual methods. *N*-Allyl-*p*-toluenesulfonamide (**3a**),[S4] *N*-methallyl-*p*-toluenesulfonamide (**3b**), *N*-(3-methyl-2-butenyl)-*p*-toluenesulfonamide (**3c**), *N*-cinnamyl-*p*-toluenesulfonamide (**3d**) were prepared from TsNHBoc and the corresponding alcohols by the Mitsunobu reaction[S5] and the following deprotection as shown in the SI (Scheme S1).[S6] Most of allylic sulfonamides were determined by comparing the spectral data of authentic samples.[S7]

N-Boc-*p*-toluenesulfonamide.

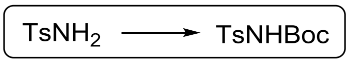

To a 1,2-dichloroethane (50 mL) solution of *p*-toluenesulfonamide (7.39 g, 43.2 mmol) were added Boc_2O (10.8 g, 50.0 mmol), triethylamine (6.60 mL, 47.5 mmol), and dimethylaminopyridines (DMAP) (0.53 g, 4.32 mmol) at room temperature. The reaction mixture was stirred overnight and poured into water (50 mL). The organic layer was separated and the aqueous layer was extracted with $CHCl_3$. The combined organic layer was washed with 1 M HCl (50 mL), water (50

mL) and then dried over $MgSO_4$. The solvent was removed under reduced pressure. The residue was precipitated from *n*-hexane and filtered off to give the titled compound (11.9 g, quant) as white powders.

^{1}H NMR (400 MHz, $CDCl_3$) δ 1.39 (9H, s, Me×3), 2.45 (3H, s, Me), 7.34 (2H, d, *J* = 7.8 Hz, ArH), 7.89 (2H, d, *J* = 7.8 Hz, ArH).

N-Boc-*N*-methallyl-*p*-toluenesulfonamide.

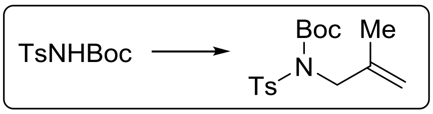

To a THF (2.0 mL) solution of *N*-Boc-*N*-*p*-toluenesulfonamide (0.500 g, 1.84 mmol), triphenylphosphine (0.483 g, 1.84 mmol), methallyl alcohol (0.133 g, 1.84 mmol) was added dropwise 2.2 M DEAD (0.80 mL, 1.84 mmol) in THF ate room temperature. The reaction mixture was stirred for 10 min and evaporated under reduced pressure. The residue was purified by column chromatography on silica gel eluting with AcOEt-*n*-hexane (1:10) to give the titled compound (0.547 g, 91%) as white powders.

^{1}H NMR (400 MHz, $CDCl_3$) δ 1.35 (9H, s, Me×3), 1.75 (3H, s, Me), 2.44 (3H, s, Me), 4.40 (2H, brs, CH_2), 4.90 (1H, brs, olefinic H), 4.93 (1H, brs, olefinic H), 7.29 (2H, d, *J* = 8.2 Hz, ArH), 7.80 (2H, d, *J* = 8.2 Hz, ArH).

N-Methallyl-*p*-toluenesulfonamide (**3b**).[S7,S8]

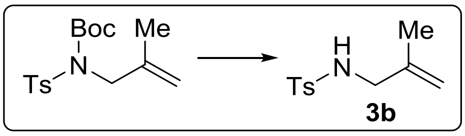

To a chloroform (5.0 mL) solution of *N*-Boc-*N*-methallyl-*p*-toluenesulfonamide (0.507 g, 1.56 mmol) was added dropwise trifluoroacetic acid (5.0 mL) at room temperature. The reaction mixture was stirred for 0.5 h and evaporated under reduced pressure. The residue was purified by column chromatography on silica gel eluting with AcOEt-*n*-hexane (1:5) to give the titled compound **3b** (0.446 g, quant) as colorless needles.

mp 36-38 ^{o}C, ^{1}H NMR (400 MHz, $CDCl_3$) δ 1.68 (3H, s, Me), 2.43 (3H, s, Me), 3.48 (2H, brs, CH_2), 4.80 (1H, brs, OH), 4.82 (1H, brs, olefinic H), 4.86 (1H, brs, olefinic H), 7.31 (2H, d, *J* = 8.3 Hz, ArH), 7.75 (2H, d, *J* = 8.3 Hz, ArH).

N-Boc-*N*-but-2-enyl-*p*-toluenesulfonamide.[27]

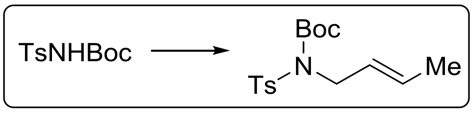

To a THF (8.0 mL) solution of *N*-Boc-*N*-*p*-toluenesulfonamide (2.00 g, 7.37 mmol), triphenylphosphine (1.90 g, 7.37 mmol), *(E)*-2-buten-1-ol (0.53 g, 7.37 mmol) was added dropwise 2.2 M DEAD (3.40 mL, 7.37 mmol) at room temperature. The reaction mixture was stirred for 10 min and then evaporated under reduced pressure. The residue was purified by column chromatography on silica gel eluting with AcOEt-*n*-hexane (1:10) to give the titled compound (2.24 g, 94%) as a white solid.

^{1}H NMR (400 MHz, $CDCl_3$) δ 1.34 (9H, s, Me×3), 1.73 (3H, d, *J* = 6.9 Hz, Me), 2.43 (3H, s, Me), 4.37 (2H, d, *J* = 6.2 Hz, CH_2), 5.56-5.61 (1H, m, olefinic H), 5.77-5.82 (1H, m, olefinic H), 7.29 (2H, d, *J* = 6.8 Hz, ArH), 7.79 (2H, d, *J* = 6.8 Hz, ArH).

(E)-*N*-But-2-enyl-*p*-toluenesulfonamide (**3c**).[S7]

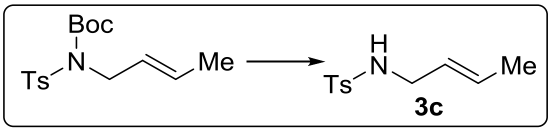

To a chloroform (11 mL) solution of *(E)*-*N*-Boc-*N*-2-butenyl-*p*-toluenesulfonamide (1.08 g, 3.32 mmol) was added dropwise trifluoroacetic acid (6.0 mL) at room temperature. The reaction mixture was stirred for 0.5 h and evaporated under reduced pressure. The residue was crystallized from *n*-hexane and filtered off to give the titled compound **3c** (0.742 g, 99%) as white powder.

mp 45-47 ^{o}C. ^{1}H NMR (400 MHz, $CDCl_3$) δ 1.59 (3H, dd, *J* = 1.4 and 6.4 Hz, Me), 2.43 (3H, s, Me), 3.50 (2H, dd, *J* = 1.4 and 6.4 Hz, CH_2), 5.28-5.35 (1H, m, olefinic H), 5.52-5.59 (1H, m, olefinic H), 5.68 (1H, brs, NH), 7.31 (2H, d, *J* = 8.2 Hz, ArH), 7.74 (2H, d, *J* = 8.2 Hz, ArH).

N-Boc-*N*-cinnamyl-*p*-toluenesulfonamide.[S9]

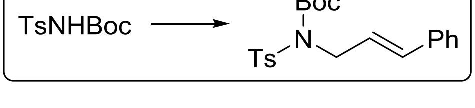

To a DMF (7.0 mL) solution of *N*-Boc-*p*-toluenesulfonamide (1.00 g, 3.69 mmol) was added 60% sodium hydride (0.22 g, 5.53 mmol) at 0 °C. The reaction mixture was stirred for 15 min at room temperature. To the mixture at 0 °C were added cinnamyl chloride (0.675 g, 4.42 mmol) and 15-crown-5-ether (0.22 g, 3.69 mmol) at 0 °C. The mixture was stirred for 12 h and poured into water (50 mL). The

organic layer was separated and the aqueous layer was extracted with AcOEt. The combined organic layer was washed with H_2O (50 mL × 2) and dried over $MgSO_4$. The solvent was removed under reduced pressure. The residue was precipitated from *n*-hexane and filtered off to give the titled compound (1.35 g, 95%) as white powders.

^{1}H NMR (400 MHz, $CDCl_3$) δ 1.35 (9H, s, Me×3), 2.41 (3H, s, Me), 4.60 (2H, dd, *J* = 1.4 and 6.9 Hz, CH2), 6.26-6.29 (1H, m, olefinic H), 6.66 (1H, brd, *J* = 15.8 Hz, olefinic H), 7.26-7.27 (3H, m, ArH), 7.31-7.34 (2H, m, ArH), 7.38 (2H, d, *J* = 7.8 Hz, ArH), 7.79 (2H, d, *J* = 8.3 Hz, ArH).

N-Cinnamyl-*p*-toluenesulfonamide (**3d**).

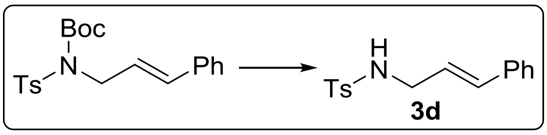

To a chloroform (20 mL) solution of *N*-Boc-*N*-cinnamyl-*p*-toluenesulfonamide (0.82 g, 2.17 mmol) was added dropwise trifluoroacetic acid (10 mL) at room temperature. The reaction mixture was stirred for 0.5 h and evaporated to give the titled compound **3d** (0.583 g, 96%) as light brown powders.

mp 82-84 ^{o}C. ^{1}H NMR (400 MHz, $CDCl_3$) δ 2.42 (3H, s, Me), 3.75 (2H, d, *J* = 6.2 Hz, CH_2), 4.62 (1H, brs, NH), 5.99-6.03 (1H, m, olefinic H), 6.43 (1H, d, *J* = 15.8 Hz, olefinic H), 7.22 (2H, brd, *J* = 7.6 Hz, ArH), 7.26 (2H, brd, *J* = 7.6 Hz, ArH), 7.27-7.29 (1H, m, ArH), 7.30 (2H, d, *J* = 8.3 Hz, ArH), 7.78 (2H, d, *J* = 8.3 Hz, ArH).

N-Boc-*N*-(3-methylbut-2-enyl)-*p*-toluenesulfonamide.[S7]

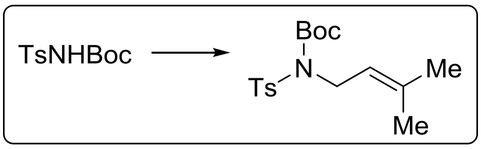

To a THF (8.0 mL) solution of *N*-Boc-*p*-toluenesulfonamide (2.00 g, 7.37 mmol), triphenylphosphine (1.90 g, 7.37 mmol), 3-methylbut-2-en-1-ol (635 mg, 7.37 mmol) was added 2.2 M DEAD (3.40 mL, 7.37 mmol) at room temperature. The reaction mixture was stirred for 0.5 h and evaporated under reduced pressure. The residue was purified by column chromatography on silica gel eluting with AcOEt-*n*-hexane (1:20 then 1:10) to give the titled compound (2.23 g, 89%) as a white solid.

^{1}H NMR (400 MHz, $CDCl_3$) δ 1.33 (9H, s, Me×3), 1.76 (3H, brs, Me), 1.77 (3H, brs, Me), 2.43 (3H, s, Me), 4.45 (2H, d, *J* = 6.9 Hz, CH_2), 5.30 (1H, brt, *J* = 6.9 Hz, olefinic H), 7.28 (2H, d, *J* = 8.3 Hz, ArH), 7.76 (2H, d, *J* = 8.3 Hz, ArH).

N-3-Methylbut-2-enyl-*p*-toluenesulfonamide (**3e**).[S7]

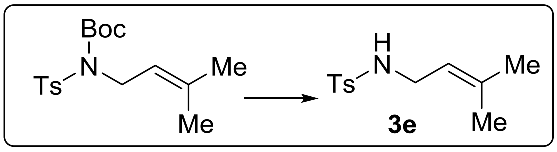

To a chloroform (5.0 mL) solution of *N*-Boc-*N*-(3-methylbut-2-enyl)-*p*-toluenesulfonamide (0.500 g, 1.47 mmol) was added dropwise trifluoroacetic acid (4.0 mL) at room temperature. The reaction mixture was stirred for 0.5 h and evaporated under reduced pressure. The residue was purified by column chromatography on silica gel eluting with AcOEt-*n*-hexane (1:5) to give the titled compound **3e** (0.335 g, quant) as white powders. mp 28-30 ^{o}C. ^{1}H NMR (400 MHz, $CDCl_3$) δ 1.53 (3H, s, Me), 1.62 (3H, s, Me), 2.43 (3H, s, Me), 3.53 (2H, brt, J = 6.4 Hz, CH_2), 4.59 (1H, brt, J = 5.5 Hz, NH), 5.03-5.07 (1H, m, olefinic H), 7.30 (2H, d, J = 8.3 Hz, ArH), 7.75 (2H, d, J = 8.3 Hz, ArH).

N-Boc-*N*-But-3-enyl-*p*-toluenesulfonamide.[S10]

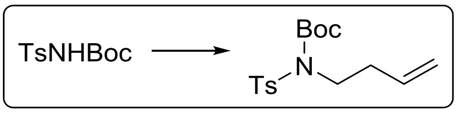

To a THF (8.0 mL) solution of *N*-Boc-*p*-toluenesulfonamide (2.00 g, 7.37 mmol), 3-buten-1-ol (0.532 g, 7.37 mmol), triphenylphosphine (1.90 g, 7.37 mmol) was added dropwise 2.2 M DEAD (3.40 mL, 7.37 mmol) at room temperature. The reaction mixture was stirred for 0.5 h and evaporated under reduced pressure. The residue was purified by column chromatography on silica gel eluting with AcOEt-*n*-hexane (1:20 then 1:10) to give the tilted compound (2.34 g, 98%) as a yellow oil.

^{1}H NMR (400 MHz, $CDCl_3$) δ 1.34 (9H, s, Me×3), 2.44 (3H, s, Me), 2.50-2.53 (2H, m, CH_2), 3.87-3.90 (2H, m, CH_2), 5.07 (1H, d, J = 10.3 Hz, olefinic H), 5.12-5.15 (1H, m, olefinic H), 5.78-5.84 (1H, m, olefinic H), 7.30 (2H, d, J = 8.3 Hz, ArH), 7.79 (2H, d, J = 8.3 Hz, ArH).

N-But-3-enyl-*p*-toluenesulfonamide (**3f**).[S7]

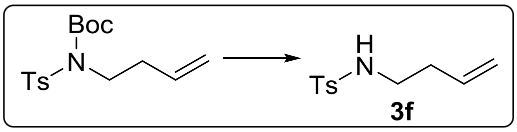

To a chloroform (24 mL) solution of *N*-Boc-*N*-but-3-enyl-*p*-toluenesulfonamide (2.31 g, 7.10 mmol) was added dropwise trifluoroacetic acid (10 mL) at room temperature. The reaction mixture stirred for 0.5 h and evaporated under reduced pressure. The residue was purified by column chromatography on silica gel eluting with

AcOEt-*n*-hexane (1:10 then 1:5) to give the titled compound **3f** (1.55 g, 97%) as colorless oil.

^{1}H NMR (400 MHz, $CDCl_3$) δ 2.20 (2H, q, *J* = 6.9 Hz, CH_2), 2.43 (3H, s, Me), 3.01 (2H, t, *J* = 6.9 Hz, CH_2), 4.48 (1H, brs, NH), 5.02-5.08 (2H, m, olefinic H), 5.60-5.66 (1H, m, olefinic H), 7.31 (2H, d, *J* = 8.2 Hz, ArH), 7.75 (2H, d, *J* = 8.3 Hz, ArH).

N-Allyl-*N*-*p*-bromobenzenesulfonamide (**3g**).[S11]

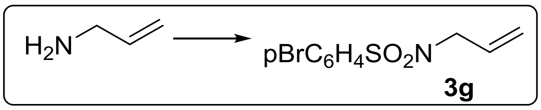

To a dichloroethane (15 mL) solution of allylamine (0.224 g, 3.91 mmol), triethyamine (0.792 g, 7.83 mmol) and DMAP (47.8 mg, 0.39 mmol) was added *p*-bromobenzenesulfonyl bromide (1.00 g, 3.91 mmol) at 0 °C. The reaction mixture was stirred for 1 h at room temperature and then poured into water (100 mL). The organic layer was separated and the aqueous layer was extracted with chloroform. The combined organic layer was washed with 1 M hydrochloric acid (50 mL) and water (50 mL) and dried over $MgSO_4$. The solvent was removed under reduced pressure. The residue was precipitated from *n*-hexane and filtrated to give the titled compound (1.04 g, 96%).

mp 50-53 ^{o}C, ^{1}H NMR (400 MHz, $CDCl_3$) δ 3.61 (2H, t, *J* = 5.9 Hz, CH_2), 4.58 (1H, brs, NH), 5.11-5.14 (1H, m, olefinic H), 5.15-5.20 (1H, m, olefinic H), 5.67-5.77 (1H, m, olefinic H), 7.66 (2H, d, *J* = 8.7 Hz, ArH), 7.74 (2H, d, *J* = 9.1 Hz, ArH).

Typical procedure for hydroamination of diynes **1** and **2** with *N*-alkenylsulfonamides **3**.

Preparation of 4-aminomethyl-3-phenylsulfanylpyrrole.

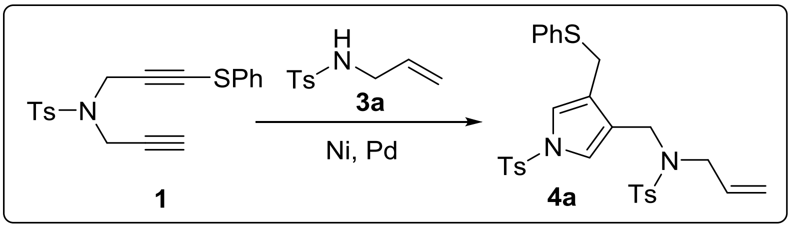

To a DMSO (1.0 mL) solution of *N*-(phenylsulfanylprop-2-yn-yl)-*N*-prop-2-ynyl-p-toluene-sulfonamide (**1**) (100 mg, 0.28 mmol) were added *N*-allyl-*p*-toluenesulfonamide (**3a**) (178 mg, 0.84 mmol), bis(hexafluoroacetylacetonato)nickel(II) hydrate (13 mg, 0.03 mmol), bis(triphenylphosphine)palladium(II) dichloride (20 mg, 0.03 mmol), and 1,8-diazabicyclo[5.4.0]undec-7-ene (43 mg, 0.28 mmol). The reaction mixture was stirred at room temperature for 6.5 h and then poured into water (50 mL). The organic

layer was separated and the aqueous layer was extracted with AcOEt. The combined organic layer was dried over $MgSO_4$. The solvent was removed under reduced pressure. The residue was purified by column chromatography on silica gel eluting with $CHCl_3$-*n*-hexane (5:1) to give *N*-allyl-*N*-[(3-(phenylsulfanylmethyl)-1-tosylpyrrole-4-yl)methyl]-*p*-toluenesulfonamide (**4a**) (89 mg, 56%) as white powders.

mp 97–101 ^{o}C, IR ν 1371, 1344, 1172 (SO_2); ^{1}H NMR (600 MHz, $CDCl_3$) δ 2.39 (3H, s, Me), 2.41 (3H, s, Me), 3.67 (2H, d, *J* = 6.2 Hz, CH_2), 3.87 (2H, s, CH_2), 4.16 (2H, s, CH_2), 4.87 (1H, dd, *J* = 1.4 and 17.2 Hz, olefinic H), 4.94 (1H, d, *J* = 11.0 Hz, olefinic H), 5.34–5.41 (1H, m, olefinic H), 6.86 (1H, d, *J* = 2.1 Hz, ArH), 6.87 (1H, d, *J* = 2.1 Hz, ArH), 7.17–7.19 (3H, m, ArH), 7.21 (2H, dd, *J* = 2.1 and 8.3 Hz), 7.24 (2H, d, *J* = 8.3 Hz, ArH), 7.27 (2H, d, *J* = 8.3 Hz, ArH), 7.58 (2H, d, *J* = 8.2 Hz, ArH), 7.68 (2H, d, *J* = 8.3 Hz, ArH); ^{13}C NMR (150 MHz, $CDCl_3$) δ 21.4 (q), 21.5 (q), 29.1 (t), 42.0 (t), 49.5 (t), 118.9 (t), 120.1 (d), 120.7 (d), 122.3 (s), 124.1 (s), 126.5 (d), 126.6 (d × 2), 127.1 (d × 2), 128.6 (d × 2), 129.7 (d × 2), 129.9 (d × 2), 130.5 (d × 2), 132.3 (d), 135.4 (s), 135.7 (s), 136.7 (s), 143.4 (s), 145.0 (s); MS (ESI-TOF) m/z 567 (M^++H). Anal. Calcd for $C_{29}H_{30}N_2O_4S_3$: C, 61.46; H, 5.34; N, 4.94. Found: C, 61.17; H, 5.37; N, 4.91.

N-Allyl-*N*-[(3-(phenylselanylmethyl)-1-tosylpyrrole-4-yl)methyl]-*p*-toluenesulfonamide (**6a**).

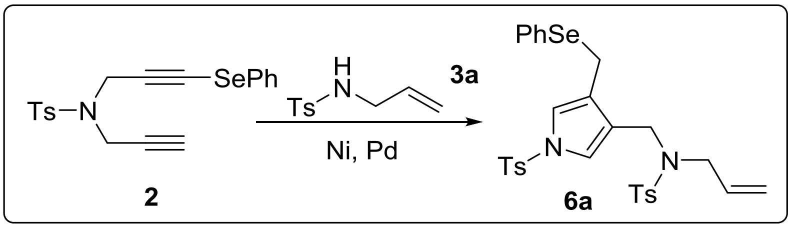

To a DMSO (3.0 mL) solution of *N*-(phenylselanylprop-2-ynyl) -*N*-prop-2-ynyl-p-toluenesulfonamide (**2**) (300 mg, 0.75 mmol) were added *N*-allyl-*p*-toluenesulfonamide (**3a**) (315 mg, 1.49 mmol), bis(hexafluoroacetylacetonato)nickel (II) hydrate (35 mg, 0.07 mmol), bis(triphenylphosphine)palladium (II) dichloride (52 mg0.07 mmol), and 1,8-diazabicyclo[5.4.0]undec-7-ene (0.11 g, 0.75 mmol). The reaction mixture was stirred at room temperature for 3.5 h and then poured into water (50 mL). The organic layer was separated and the aqueous layer was extracted with AcOEt. The combined

organic layer was dried over $MgSO_4$. The solvent was removed under reduced pressure. The residue was purified by column chromatography on silica gel eluting with $CHCl_3$-*n*-hexane (4:1) to give *N*-Allyl-*N*-[(3-(phenylselanylmethyl)-1-tosylpyrrole-4-yl)methyl]-*p*-toluenesulfonamide (**6a**) (0.325 mg, 71%) as white powders.

mp 74–76 ^oC, IR ν 1371, 1344, 1172 (SO_2); ^1H NMR (600 MHz, $CDCl_3$) δ 2.41 (3H, s, Me), 2.43 (3H, s, Me), 3.68 (2H, d, *J* = 6.2 Hz, CH_2), 3.83 (2H, s, CH_2), 4.15 (2H, s, CH_2), 4.89 (1H, d, *J* = 17.2 Hz, olefinic H), 4.95 (1H, d, *J* = 10.3 Hz, olefinic H), 5.36-5.42 (1H, m, olefinic H), 6.71 (1H, d, *J* = 2.1 Hz, ArH), 6.84 (1H, d, *J* = 2.1 Hz, ArH), 7.13-7.19 (2H, m, ArH), 7.25-7.33 (7H, m, ArH), 7.58 (2H, d, *J* = 8.2 Hz, ArH), 7.69 (2H, d, *J* = 7.6 Hz, ArH); ^{13}C NMR (150 MHz, $CDCl_3$) δ 21.3 (t), 21.5 (q), 21.6 (q), 41.9 (t), 49.5 (t), 119.0 (t), 119.7 (d), 120.6 (d), 122.1 (s), 124.9 (s), 126.7 (d × 2), 127.2 (d × 2), 127.3 (d), 128.8 (d × 2), 129.6 (s), 129.7 (d × 2), 129.9 (d × 2), 132.3 (d), 134.0 (d × 2), 135.7 (s), 136.7 (s), 143.4 (s), 145.0 (s); MS (EI) m/z 614 (M^+), 457 (M^+-SePh). HRMS (ESI-TOF) m/z: $[M+Na]^+$ Calcd for $C_{29}H_{30}N_2O_4S_2SeNa$ 637.0710, found 637.0727. Anal. Calcd for $C_{29}H_{30}N_2O_4S_2Se$: 56.76; H, 4.93; N, 4.56. Found: C, 56.48; H, 4.75; N, 4.36.

Oxidation of 3-(phenylsulfanylmethyl)-4-(*N*-alkenyl-*N*-tosylaminomethyl)pyrroles.

N-Allyl-*N*-[(3-(phenylsulfinylmethyl)-1-tosylpyrrole-4-yl)methyl]-*p*-toluenesulfonamide (**5a**).

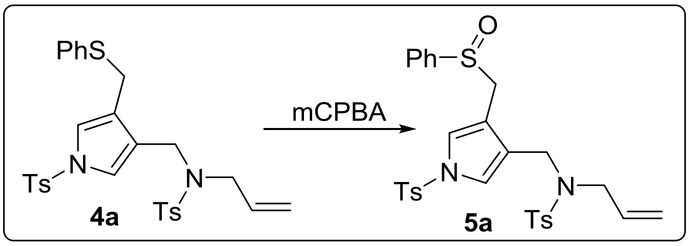

To a 1,2-dichloroethane (5.0 mL) solution of *N*-allyl-*N*-[3-(phenylsulfanylmethyl) -1-tosylpyrrole-4-methyl]-*p*-toluene sulfonamide (**4a**) (144 mg, 0.25 mmol) was added portionwise *m*-chloroperbenzoic acid (44 mg, 0.25 mmol) over 1 h at 0 ^oC. The reaction mixture was stirred for 5 min and poured into a sat. $NaHCO_3$ (50 mL). The whole was vigorously stirred for 15 min. The organic layer was separated and the aqueous layer was extracted with chloroform. The combined organic layer was dried over $MgSO_4$. The solvent was removed under reduced pressure. The residue was purified by preparative TLC on silica gel eluting with AcOEt-*n*-hexane (1:2) to give

N-allyl-*N*-[(3-(phenylsulfinylmethyl)-1-tosylpyrrole-4-yl)methyl]-*p*-toluenesulfonamide (**5a**) (139 mg, 94%) as white powders.

mp 119–122 °C, IR ν 1372, 1334, 1171 (SO_2); ^{1}H NMR (600 MHz, $CDCl_3$) δ 2.42 (3H, s, Me), 2.43 (3H, s, Me), 3.61 (2H, dt, *J* = 6.2 and 15.8 Hz, CH × 2), 3.79 (1H, d, *J* = 14.5 Hz, CH), 3.83 (1H, d, *J* = 14.4 Hz, CH), 3.85 (1H, d, *J* = 13.7 Hz, CH), 4.12 (1H, d, *J* = 13.7 Hz, CH), 4.84 (1H, d, *J* = 17.2 Hz, olefinic H), 4.93 (1H, d, *J* = 10.3 Hz, olefinic H), 5.21-5.28 (1H, m, olefinic H), 6.87 (1H, d, *J* = 2.0 Hz, ArH), 7.07 (1H, d, *J* = 2.1 Hz, ArH), 7.28–7.35 (6H, m, ArH), 7.41-7.44 (3H, m, ArH), 7.64 (2H, d, *J* = 8.3 Hz, ArH), 7.73 (2H, d, *J* = 8.2 Hz, ArH); ^{13}C NMR (150 MHz, $CDCl_3$) δ 21.5 (q), 21.6 (q), 41.7 (t), 49.3 (t), 52.7 (t), 116.1 (s), 119.2 (t), 120.5 (d), 122.37 (d), 122.40 (s), 124.3 (d × 2), 127.0 (d × 2), 127.1 (d × 2), 128.7 (d × 2), 129.8 (d × 2), 130.0 (d × 2), 131.8 (d), 135.6 (s), 136.5 (s), 143.0 (s), 143.6 (s), 145.3 (s); MS (EI) m/z 457 (M^+–SOPh). Anal. Calcd for $C_{29}H_{30}N_2O_5S_3$+1/2H_2O: C, 58.86; H, 5.28; N, 4.73. Found: C, 58.98; H, 5.08; N, 4.73.

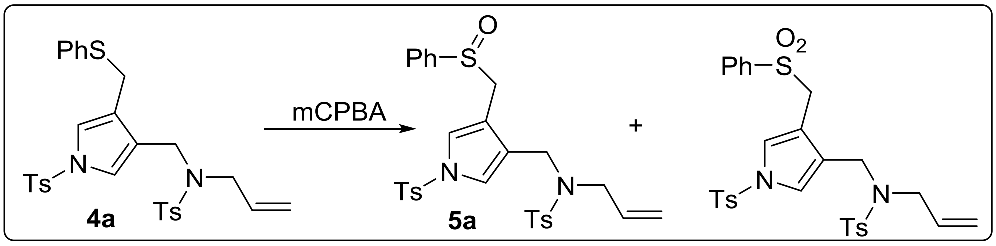

N-Allyl-*N*-[(3-(phenylsulfonylmethyl)-1-tosylpyrrole-4-yl)methyl]-*p*-toluenesulfonamide.

m-Chloroperbenzoic acid (0.44 g, 2.58 mmol) was added portion wise to the dichloroethane (66 mL) solution of **4a** (1.46 g, 2.58 mmol) at 0 °C. The reaction mixture was stirred for 0.5 h and poured into sat. $NaHCO_3$ (50 mL). The mixture was vigorously stirred for 0.5 h. The organic layer was separated and the aqueous layer was extracted with chloroform. The combined organic layer was dried over $MgSO_4$. The solvent was removed under reduced pressure. The residue was purified by column chromatography on silica gel eluting with AcOEt-*n*-hexane (1:2 then 1:1) to give **5a** (0.464 g, 31%) and the corresponding sulfone (0.398 g, 26%).

mp 166-169 °C, IR (KBr, cm^{-1}) 1374, 1334, 1158 (SO_2), 1091 (SO); ^{1}H NMR (600 MHz, $CDCl_3$) δ 2.44 (3H, s, Me), 2.45 (3H, s, Me), 3.59 (2H, d, *J* = 6.9 Hz, NCH_2), 3.97 (2H, s, CH_2), 4.32 (2H, s, CH_2), 4.88 (2H, brd, *J* = 16.5 Hz, olefinic H), 4.95 (2H, d, *J* = 9.0 Hz,

olefinic H), 5.24-5.30 (1H, m, olefinic H), 6.91 (1H, d, J = 2.0 Hz, ArH), 7.10 (1H, d, J = 2.1 Hz, ArH), 7.30-7.34 (4H, m, ArH), 7.43 (2H, t, J = 8.3 Hz, ArH), 7.60 (1H, t, J = 7.6 Hz, ArH), 7.66 (2H, d, J = 8.2 Hz, ArH), 7.71 (2H, d, J = 8.2 Hz, ArH), 7.75 (2H, d, J = 8.5 Hz, ArH); ^{13}C NMR (150 MHz, $CDCl_3$) δ 21.5 (q), 21.6 (q), 41.7 (t), 49.6 (t), 52.5 (t), 114.7 (s), 119.2 (t), 120.8 (d), 122.8 (s), 122.9 (d), 127.0 (dx2), 127.2 (dx2), 128.6 (dx2), 128.9 (dx2), 129.8 (dx2), 130.1 (dx2), 132.0 (d), 133.7 (d), 135.5 (s), 136.4 (s), 138.2 (s), 143.6 (s), 145.5 (s); MS (ESI-TOF) m/z 621 $[M + Na]^+$. HRMS (ESI-TOF) m/z: $[M+Na]^+$ Calcd for $C_{29}H_{30}N_2O_6S_3BrNa$ 621.1164; found 621.1149.

Single X-ray analysisi of sulfone was described in the CIF format.

N-[(3-(Phenylsulfanylmethyl)-1-tosylpyrrole-4-yl)methyl]-*N*-methallyl-*p*-toluenesulfonamide (**4b**).

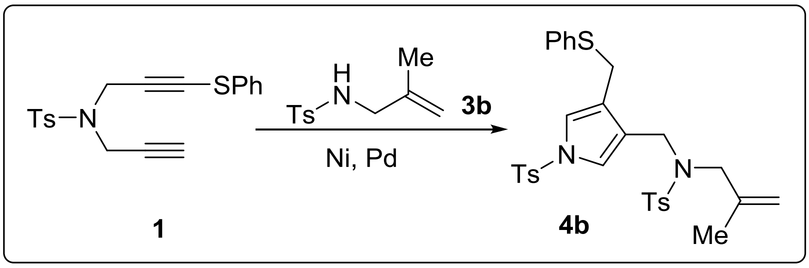

To a DMSO (0.5 mL) solution of *N*-(phenylsulfanylprop-2-ynyl)-*N*-prop-2-ynyl-p-toluenesulfonamide (**1**) (50 mg, 0.14 mmol) were added *N*-methallyl-*p*-toluenesulfonamide (**3b**)[S6,S7] (63 mg, 0.28 mmol), bis(triphenylphosphine)nickel (II) dichloride (9.2 mg, 0.014 mmol) and 1,8-diazabicyclo[5.4.0]undec-7-ene (21 mg, 0.14 mmol). The reaction mixture was stirred at room temperature for 2 h and then poured into water (50 mL). The organic layer was separated and the aqueous layer was extracted with AcOEt. The combined organic layer was washed with water (50 mL) and dried over $MgSO_4$. The solvent was removed under reduced pressure. The residue was purified by column chromatography on silica gel eluting with chloroform-*n*-hexane (5:1) to give *N*-[(3-(phenylsulfanylmethyl)-1-tosylpyrrole-4-yl)methyl]-*N*-methallyl-*p*-toluenesulfonamide (**4b**) (37 mg, 45%) as white powders.

mp 96–99 ^{o}C, IR ν 1371, 1336, 1172 (SO_2); ^{1}H NMR (600 MHz, $CDCl_3$) δ 1.46 (3H, s, Me), 2.40 (3H, s, Me), 2.41 (3H, s, Me), 3.61 (2H, s, CH_2), 3.82 (2H, s, CH_2), 4.15 (2H, s, CH_2), 4.61 (1H, s, olefinic H), 4.69 (1H, s, olefinic H), 6.73 (1H, d, J = 2.8 Hz, ArH), 6.82 (1H, d, J = 2.0 Hz, ArH), 7.16–7.21 (5H, m, ArH), 7.24 (2H, brd, J =8.9 Hz, ArH), 7.26 (2H,

d, J = 8.2 Hz, ArH), 7.56 (2H, d, J = 8.9 Hz, ArH), 7.66 (2H, d, J = 8.2 Hz, ArH); ^{13}C NMR (150 MHz, $CDCl_3$) δ 19.7 (q), 21.4 (q), 21.5 (q), 29.2 (t), 42.3 (t), 53.6 (t), 114.0 (t), 119.6 (d), 120.7 (d), 122.4 (s), 123.9 (s), 126.5 (d), 126.7 (d × 2), 127.1 (d × 2), 128.7 (d × 2), 129.6 (d × 2), 129.9 (d × 2), 130.5 (d × 2), 135.4 (s), 135.7 (s), 136.6 (s), 140.2 (s), 143.4 (s), 144.9 (s); MS (EI) 580 (small M^+), 471, (M^+–SPh), 425 (M^+–Ts). Anal. Calcd for $C_{30}H_{32}N_2O_4S_3$+1/4H_2O: C, 61.57; H, 5.60; N, 4.79. Found: C, 61.76; H, 5.63; N, 4.51.

N-[(3-(Phenylsulfanylmethyl)-1-tosylpyrrole-4-yl)methyl]-*N*-(but-2-enyl)-*p*-toluenesulfonamide (**4c**).

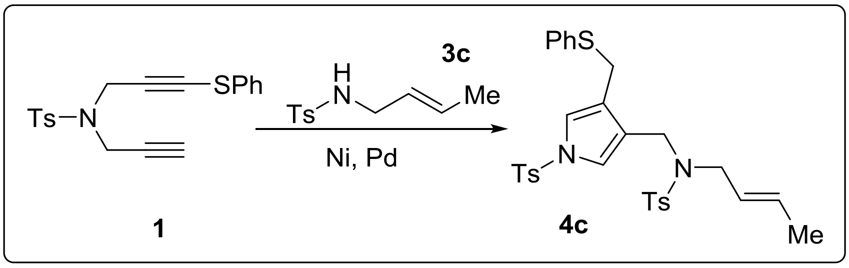

To a DMSO (1.0 mL) solution of *N*-(phenylsulfanylprop-2-ynyl)-*N*-prop-2-ynyl-p-toluenesulfonamide (**1**) (50 mg, 0.14 mmol) were added *N*-but-2-enyl-*p*-toluenesulfonamide (**3c**) (63.4 mg, 0.28 mmol), bis(hexafluoroacetylacetonato)nickel(II) hydrate (6.7 mg, 0.014 mmol), bis(triphenylphosphine)palladium(II) dichloride (9.9 mg, 0.014 mmol), and 1,8-diazabicyclo[5.4.0]undec-7-ene (21 mg, 0.14 mmol). The reaction mixture was stirred at room temperature for 10 h and then poured into water (50 mL). The organic layer was separated and the aqueous layer was extracted with AcOEt. The combined organic layer was washed with water (50 mL) and dried over $MgSO_4$. The solvent was removed under reduced pressure. The residue was purified by column chromatography on silica gel eluting with $CHCl_3$-*n*-hexane (5:1) to give *N*-[(3-(phenylsulfanylmethyl)-1-tosylpyrrole-4-yl)methyl]-*N*-(but-2-enyl)-*p*-toluenesulfonamide (**4c**) (66 mg, 80%) as white powder.

Procedure for large scale preparation of **4c**: To a DMSO (5.0 mL) solution of *N*-(phenylsulfanylprop-2-ynyl)-*N*-prop-2-ynyl-*p*-toluenesulfonamide (**1**) (481 mg, 1.35 mmol) were added *N*-but-2-enyl-*p*-toluenesulfonamide (**3c**) (610 mg, 2.71 mmol), bis(hexafluoroacetylacetonato)-nickel(II) hydrate (64 mg, 0.14 mmol), bis(triphenylphosphine)palladium(II) dichloride (95 mg, 0.14 mmol), and

1,8-diazabicyclo[5.4.0]undec-7-ene (0.21 g, 1.35 mmol). The workup procedure gave **4c** (341 mg, 43%).

mp 63–65 °C, IR ν 1371, 1337, 1172 (SO_2); ^{1}H NMR (600 MHz, $CDCl_3$) δ 1.50 (3H, d, *J* = 6.9 Hz, Me), 2.41 (3H, s, Me), 2.42 (3H, s, Me), 3.62 (2H, d, *J* = 6.9 Hz, CH_2), 3.86 (2H, s, CH_2), 4.15 (2H, s, CH_2), 5.01–5.07 (1H, m, olefinic H), 5.26–5.32 (1H, m, olefinic H), 6.85 (1H, brs, ArH), 6.86 (1H, brs, ArH), 7.16–7.22 (5H, m, ArH), 7.25 (2H, d, *J* = 9.6 Hz, ArH), 7.27 (2H, d, *J* = 7.6 Hz, ArH), 7.59 (2H, d, *J* = 8.3 Hz, ArH), 7.67 (2H, d, *J* = 8.2 Hz, ArH); ^{13}C NMR (150 MHz, $CDCl_3$) δ 17.6 (q), 21.5 (q), 21.6 (q), 29.2 (t), 41.7 (t), 48.9 (t), 120.0 (d), 120.5 (d), 122.6 (s), 124.1 (s), 124.8 (d), 126.5 (d), 126.7 (d × 2), 127.2 (d × 2), 128.7 (d × 2), 129.6 (d × 2), 129.9 (d × 2), 130.5 (d × 2), 130.7 (d), 135.5 (s), 135.8 (s), 136.9 (s), 143.3 (s), 145.0 (s); MS (EI) m/z 580 (small M^+), 471 (M^+–SPh), 425 (M^+–Ts). Anal. Calcd for $C_{30}H_{32}N_2O_4S_3$+1/4H_2O: C, 61.57; H, 5.60; N, 4.79. Found: C, 61.56; H, 5.57; N, 4.84.

N-[(3-(Phenylsulfanylmethyl)-1-tosylpyrrole-4-methyl]-*N*-cinnamyl-*p*-toluenesulfonamide (**4d**).

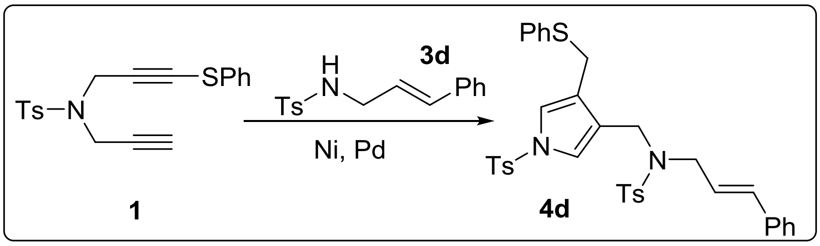

To a DMSO (1.0 mL) solution of *N*-(phenylsulfanylprop-2-ynyl)-*N*-prop-2-ynyl-p-toluenesulfonamide (**1**) (50 mg, 0.14 mmol) were added *N*-cinnamyl-*p*-toluenesulfonamide (**3d**)[28] (81 mg, 0.28 mmol), bis(hexafluoroacetylacetonato)-nickel(II) hydrate (6.7 mg, 0.014 mmol), bis(triphenylphosphine)palladium(II) dichloride (9.9 mg, 0.014 mmol), and 1,8-diazabicyclo[5.4.0]undec-7-ene (21 mg, 0.14 mmol). The reaction mixture was stirred at room temperature for 1.5 h and then poured into water (50 mL). The organic layer was separated and the aqueous layer was extracted with AcOEt. The combined organic layer was dried over $MgSO_4$. The solvent was removed under reduced pressure. The residue was purified by column chromatography on silica gel eluting with $CHCl_3$-*n*-hexane (5:1) to give *N*-[(3-(phenylsulfanylmethyl)-1-tosyl-

pyrrole-4-yl)methyl]-*N*-cinnamyl-*p*-toluenesulfonamide (**4d**) (68 mg, 75%) as white powder.

Procedure for large scale preparation of **4d**: To a DMSO (2.8 mL) solution of *N*-(phenylsulfanylprop-2-ynyl)-*N*-prop-2-ynyl-*p*-toluenesulfonamide (**1**) (278.3 mg, 0.78 mmol) were added *N*-but-2-enyl-*p*-toluenesulfonamide (**3d**) (450 mg, 1.57 mmol), bis(hexafluoroacetylacetonato)-nickel(II) hydrate (37 mg, 0.08 mmol), bis(triphenylphosphine)palladium(II) dichloride (55 mg, 0.08 mmol), and 1,8-diazabicyclo[5.4.0]undec-7-ene (0.12 g, 0.78 mmol). The workup procedure gave **4d** (351 mg, 70%).

mp 79–83 °C, IR ν 1372, 1340, 1172 (SO_2); ^{1}H NMR (600 MHz, $CDCl_3$) δ 2.37 (3H, s, Me), 2.41 (3H, s, Me), 3.83 (2H, d, *J* = 6.8 Hz, CH_2), 3.84 (2H, s, CH_2), 4.21 (2H, s, CH_2), 5.70-5.75 (1H, m, olefinic H), 6.19 (1H, d, *J* = 15.9 Hz, olefinic H), 6.87 (1H, d, *J* = 2.0 Hz, ArH), 6.91 (1H, d, *J* = 2.1 Hz, ArH), 7.10 (2H, d, *J* = 6.8 Hz, ArH), 7.13-7.17 (4H, m, ArH), 7.22 (3H, brt, *J* = 8.2 Hz, ArH), 7.24 (2H, s, ArH), 7.27 (3H, t, *J* = 8.3 Hz, ArH), 7.58 (2H, d, *J* = 8.9 Hz, ArH), 7.71 (2H, d, *J* = 8.2 Hz, ArH); ^{13}C NMR (150 MHz, $CDCl_3$) δ 21.5 (q), 21.6 (q), 29.2 (t), 42.0 (t), 48.9 (t), 120.1 (d), 120.6 (d), 122.4 (s), 123.2 (d), 124.0 (s), 126.3 (d × 2), 126.5 (d), 126.7 (d × 2), 127.3 (d × 2), 127. 9 (d), 128.5 (d × 2), 128.7 (d × 2), 129.8 (d × 2), 130.0 (d × 2), 130.5 (d × 2), 134.2 (d), 135.4 (s), 135.7 (s), 136.0 (s), 136.8 (s), 143.5 (s), 145.0 (s); MS (EI) m/z 487 (M^+-Ts). HRMS (ESI-TOF) m/z: $[M+Na]^+$ Calcd for $C_{35}H_{34}N_2O_4S_3Na$ 665.1578; found m/z 665.1577. Anal. Calcd for $C_{35}H_{34}N_2O_4S_3$+1/3H_2O: C, 64.79; H, 5.39; N, 4.32. Found: C, 64.67; H, 5.46; N, 4.31.

N-[(3-(Phenylsulfanylmethyl)-1-tosylpyrrole-4-yl)methyl]-*N*-(3-methyl-2-butenyl)-*p*-toluene sulfonamide (**4e**).

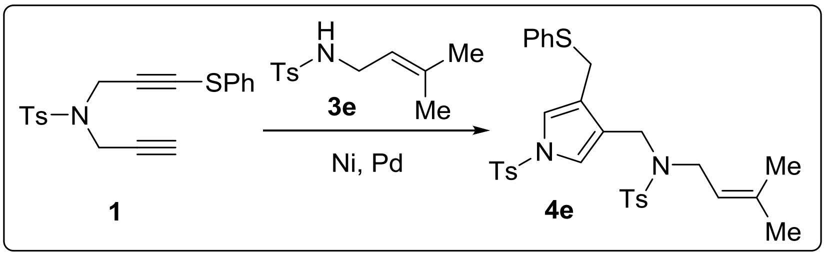

To a DMSO (0.5 mL) solution of *N*-(phenylsulfanylprop-2-ynyl)-*N*-prop-2-ynyl-p-toluenesulfonamide (**1**) (50 mg, 0.14 mmol) were added *N*-(3-methyl-2-butenyl)-*p*-toluenesulfonamide (**3e**) (67 mg, 0.28 mmol), bis(hexafluoroacetylacetonato)nickel(II) hydrate (7 mg, 0.01 mmol),

bis(triphenylphosphine)palladium(II) dichloride (10 mg, 0.01 mmol), and 1,8-diazabicyclo[5.4.0]undec-7-ene (21 mg, 0.14 mmol). The reaction mixture was stirred at room temperature for 17 h and then poured into water (50 mL). The organic layer was separated and the aqueous layer was extracted with AcOEt. The combined organic layer was dried over $MgSO_4$. The solvent was removed under reduced pressure. The residue was purified by column chromatography on silica gel eluting with chloroform-*n*-hexane (4:1) to give *N*-[(3-(phenylsulfanylmethyl)-1-tosylpyrrole-4-ly)methyl]-*N*-(3-methyl-2-butenyl)-*p*-toluene sulfonamide (**4e**) (62 mg, 74%) as white powders.

mp 42–45 ^{o}C, IR ν 1371, 1339, 1173 (SO_2); ^{1}H NMR (600 MHz, $CDCl_3$) δ 1.34 (3H, s, Me), 1.53 (3H, s, Me), 2.41 (3H, s, Me), 2.43 (3H, s, Me), 3.70 (2H, d, *J* = 6.9 Hz, CH_2), 3.88 (2H, s, CH_2), 4.15 (2H, s, CH_2), 4.75-4.77 (1H, m, olefinic H), 6.84 (1H, d, *J* = 2.1 Hz, ArH), 6.87 (1H, d, *J* = 2.1 Hz, ArH), 7.17–7.20 (3H, m, ArH), 7.21–7.25 (4H, m, ArH), 7.28 (2H, d, *J* = 7.5 Hz, ArH), 7.59 (2H, d, *J* = 8.2 Hz, ArH), 7.67 (2H, d, *J* = 8.3 Hz, ArH); ^{13}C NMR (150 MHz, $CDCl_3$) δ 17.5 (q), 21.5 (q), 21.6 (q), 25.6 (q), 29.2 (t), 41.9 (t), 44.6 (t), 118.2 (d), 120.0 (d), 120.3 (d), 122.6 (s), 124.0 (s), 126.5 (d), 126.7 (d × 2), 127.2 (d × 2), 128.7 (d × 2), 129.6 (d × 2), 129.9 (d × 2), 130.5 (d × 2), 135.5 (s), 135.7 (s), 136.7 (s), 136.8 (s), 143.3 (s), 145.0 (s); MS (ESI-TOF) m/z 617 $[M + Na]^+$; HRMS (ESI-TOF) m/z: $[M+Na]^+$ Calcd for $C_{31}H_{34}N_2O_4S_3Na$ 617.1578; found 617.1552. Anal. Calcd for $C_{31}H_{34}N_2O_4S_3$+1/6H_2O: C, 62.28; H, 5.79; N, 4.69. Found: C, 62.53; H, 5.85; N, 4.39.

N-[(3-(Phenylsulfanylmethyl)-1-tosylpyrrole-4-yl)methyl]-*N*-(3-butenyl)-*p*-toluenesulfon-amide (**4f**).

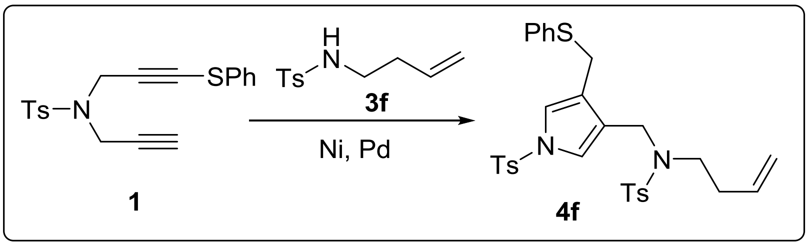

To a DMSO (0.5 mL) solution of *N*-(phenylsulfanylprop-2-ynyl)-*N*-prop-2-ynyl-p-toluenesulfonamide (**1**) (50 mg, 0.14 mmol) were added *N*-(3-butenyl)-*p*-toluenesulfonamide (**3f**)[29] (67 mg, 0.28 mmol), bis(hexafluoroacetylacetonato)nickel(II) hydrate (7 mg, 0.01 mmol), bis(triphenylphosphine)palladium(II) dichloride (10 mg, 0.01 mmol), and

1,8-diazabicyclo[5.4.0]undec-7-ene (21 mg, 0.14 mmol). The reaction mixture was stirred at room temperature for 17 h and then poured into water (50 mL). The organic layer was separated and the aqueous layer was extracted with AcOEt. The combined organic layer was dried over $MgSO_4$. The solvent was removed under reduced pressure. The residue was purified by column chromatography on silica gel eluting with chloroform-*n*-hexane (5:1) to give *N*-[(3-(phenylsulfanylmethyl)-1-tosylpyrrole-4-yl)methyl]-*N*-(3-butenyl)-*p*-toluenesulfon-amide (**4f**) (50 mg, 61%) as white powders.

mp 78–80 ^{o}C, IR ν 1372, 1338, 1173 (SO_2); ^{1}H NMR (600 MHz, $CDCl_3$) δ 1.99 (2H, dd, *J*=6.9 and 15.1 Hz, CH_2), 2.41 (3H, s, Me), 2.43 (3H, s, Me), 3.04 (2H, br t, *J* = 8.3 Hz, CH_2), 3.89 (2H, s, CH_2), 4.14 (2H, s, CH_2), 4.81 (1H, dd, *J* = 1.4 amd 17.2 Hz, olefinic H), 4.88 (1H, dd, *J* = 2.1 amd 10.3 Hz, olefinic H), 5.41-5.48 (1H, m, olefinic H), 6.88 (1H, d, *J* = 2.0 Hz, ArH), 6.89 (1H, d, *J* = 2.7 Hz, ArH), 7.18-7.25 (7H, m, ArH), 7.29 (2H, d, *J* = 8.3 Hz, ArH), 7.58 (2H, d, *J* = 8.2 Hz, ArH), 7.68 (2H, d, *J* = 8.2 Hz, ArH); ^{13}C NMR (150 MHz, $CDCl_3$) δ 21.5 (q), 21.6 (q), 29.2 (t), 33.0 (t), 43.7 (t), 47.4 (t), 116.8 (t), 120.2 (d), 120.4 (d), 122.8 (s), 124.0 (s), 126.6 (d), 126.7 (d × 2), 127.2 (d × 2), 128.7 (d × 2), 129.7 (d × 2), 129.9 (d × 2), 130.6 (d × 2), 134.5 (d), 135.4 (s), 135.7 (s), 136.2 (s), 143.4 (s), 145.0 (s); MS (EI) m/z 580 (small M^+), 471 (M^+–SPh), 425 (M^+–Ts). Anal. Calcd for $C_{30}H_{32}N_2O_4S_3$: C, 62.04; H, 5.55; N, 4.82. Found: C, 61.75; H, 5.62; N, 4.74.

N-[(3-(Phenylselanylmethyl)-1-tosylpyrrole-4-yl)methyl]-*N*-methallyl-*p*-toluenesulfonamide (**6b**).

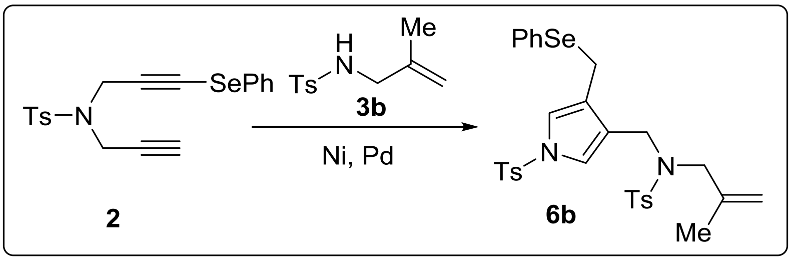

To a DMSO (3.0 mL) solution of *N*-(phenylselanylprop-2-ynyl)-*N*-prop-2-ynyl-*p*-toluenesulfonamide (**2**) (300 mg, 0.75 mmol) were added *N*-methallyl-*p*-toluenesulfonamide (**3b**) (336 mg, 1.49 mmol), bis(hexafluoroacetylacetonato)nickel(II) hydrate (35 mg, 0.07 mmol), bis(triphenylphosphine)palladium(II) dichloride (52 mg, 0.07 mmol), and 1,8-diazabicyclo[5.4.0]undec-7-ene (0.11 g, 0.75 mmol). The reaction mixture was

stirred at room temperature for 9 h and then poured into water (50 mL). The organic layer was separated and the aqueous layer was extracted with AcOEt. The combined organic layer was washed with water (50 mL) and then dried over $MgSO_4$. The solvent was removed under reduced pressure. The residue was purified by column chromatography on silica gel eluting with chloroform-*n*-hexane (4:1) to give *N*-[(3-(phenylselanylmethyl)-1-tosylpyrrole-4-yl)methyl]-*N*-methallyl-*p*-toluenesulfonamide (**6b**) (0.347 mg, 74%) as white needle crystals.

mp 98–102 ^{o}C, IR ν 1370, 1335, 1172 (SO_2); ^{1}H NMR (600 MHz, $CDCl_3$) δ 1.46 (3H, s, Me), 2.42 (3H, s, Me), 2.43 (3H, s, Me), 3.61 (2H, s, CH_2), 3.79 (2H, s, CH_2), 4.13 (2H, s, CH_2), 4.61 (1H, s, olefinic H), 4.69 (1H, s, olefinic H), 6.68 (1H, d, *J* = 2.0 Hz, ArH), 6.71 (1H, d, *J* = 2.1 Hz, ArH), 7.15 (2H, t, *J* = 7.6 Hz, ArH), 7.22-7.24 (1H, m, ArH), 7.26-7.28 (4H, m, ArH), 7.31 (2H, br d, *J* = 6.9 Hz, ArH), 7.58 (2H, d, *J* = 8.2 Hz, ArH), 7.67 (2H, d, *J* = 8.2 Hz, ArH); ^{13}C NMR (150 MHz, $CDCl_3$) δ 19.7 (q), 21.3 (t), 21.5 (q), 21.6 (q), 42.3 (t), 53.6 (t), 113.9 (t), 119.2 (d), 120.7 (d), 122.3 (s), 124.7 (s), 126.7 (d × 2), 127.1 (d × 2), 127.4 (d), 128.8 (d × 2), 129.58 (s), 129.60 (d × 2), 129.8 (d × 2), 133.9 (d × 2), 135.7 (s), 136.6 (s), 140.2 (s), 143.4 (s), 144.9 (s); MS (EI) m/z 471 (M^+-SePh), 316 (M^+-SePh-Ts). MS (ESI-TOF) m/z 651 $[M + Na]^+$. HRMS (ESI-TOF) m/z: $[M+Na]^+$ Calcd for $C_{30}H_{32}N_2O_4S_2SeNa$ 651.0866; found 651.0871. Anal. Calcd for $C_{30}H_{32}N_2O_4S_2Se$+1/4H_2O: C, 57.00; H, 5.18; N, 4.43. Found: C, 57.02; H, 4.98; N, 4.19.

N-[(3-(Phenylselanylmethyl)-1-tosylpyrrole-4-yl)methyl]-*N*-but-2-enyl-*p*-toluenesulfon-amide (**6c**).

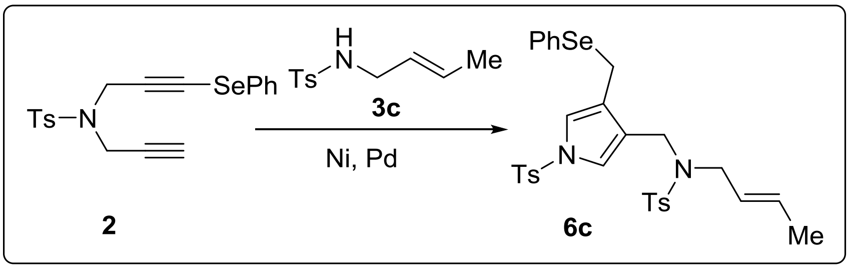

To a DMSO (3.5 mL) solution of *N*-(phenylselanylprop-2-ynyl)-*N*-prop-2-ynyl-*p*-toluenesulfonamide (**2**) (356 mg, 1.00 mmol) were added *N*-but-2-enyl-*p*-toluenesulfonamide (**3c**) (339 mg, 1.50 mmol), bis(hexafluoroacetylacetonato)nickel(II) hydrate (47 mg, 0.10 mmol), bis(triphenylphosphine)palladium(II) dichloride (70 mg, 0.10 mmol), and 1,8-diazabicyclo[5.4.0]undec-7-ene (0.15 g, 1.00 mmol). The reaction mixture was

stirred at room temperature for 5.5 h and then poured into water (50 mL). The organic layer was separated and the aqueous layer was extracted with AcOEt. The combined organic layer was washed with water (50 mL) and dried over $MgSO_4$. The solvent was removed under reduced pressure. The residue was purified by column chromatography on silica gel eluting with chloroform-*n*-hexane (5:1) gave *N*-[(3-(phenylselanylmethyl)-1-tosylpyrrole-4-yl)methyl]-*N*-but-2-enyl-*p*-toluenesulfonamide (**6c**) (0.226 mg, 35%) as white powders.

mp 69–72 ^{o}C, IR ν 1371, 1334, 1161 (SO_2); ^{1}H NMR (600 MHz, $CDCl_3$) δ 1.51 (3H, dd, *J*=6.9 and 1.4 Hz, Me), 2.42 (3H, s, Me), 2.43 (3H, s, Me), 3.62 (2H, d, *J*=6.9 Hz, CH_2), 3.82 (2H, s, CH_2), 4.14 (2H, s, CH_2), 5.02-5.07 (1H, m, olefinic H), 5.28-5.34 (1H, m, olefinic H), 6.70 (1H, d, *J*=2.1 Hz, ArH), 6.84 (1H, d, *J*=2.1 Hz, ArH), 7.14-7.17 (2H, m, ArH), 7.22-7.24 (1H, m, ArH), 7.26-7.29 (4H, m, ArH), 7.32-7.33 (2H, m, ArH), 7.59 (2H, d, *J*=8.3 Hz, ArH), 7.68 (2H, d, *J*=8.2 Hz, ArH); ^{13}C NMR (150 MHz, $CDCl_3$) δ 17.6 (q), 21.4 (t), 21.5 (q), 21.6 (q), 41.6 (t), 48.9 (t), 119.6 (d), 120.5 (d), 122.5 (s), 124.8 (d), 124.9 (s), 126.7 (d × 2), 127.2 (d × 2), 127.4 (d), 128.8 (d × 2), 129.6 (d × 2), 129.7 (s), 129.9 (d × 2), 130.7 (d), 133.9 (d × 2), 135.8 (s), 136.9 (s), 143.3 (s), 145.0 (s); MS (EI) m/z 628 (small M^+), 471 (M^+-SePh). Anal. Calcd for $C_{30}H_{32}N_2O_4S_2Se+1/2H_2O$: C, 56.59; H, 5.22; N, 4.40. Found: C, 56.32; H, 5.38; N, 4.45.

N-[(3-(Phenylselanylmethyl)-1-tosylpyrrole-4-ly)methyl]-*N*-cinnamyl-*p*-toluenesulfonamide (**6d**).

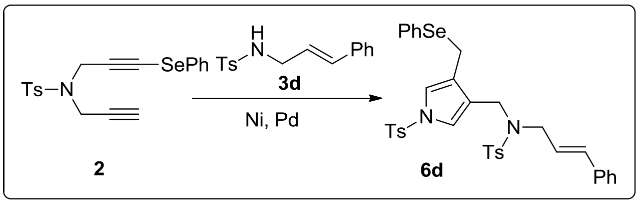

To a DMSO (3.0 mL) solution of *N*-(phenylselanylprop-2-ynyl)-*N*-prop-2-ynyl-p-toluenesulfonamide (**2**) (300 mg, 0.75 mmol) were added *N*-cinnamyl-*p*-toluenesulfonamide (**3d**) (429 mg, 1.49 mmol), bis(hexafluoroacetylacetonato)nickel(II) hydrate (35 mg, 0.07 mmol), bis(triphenylphosphine)palladium(II) dichloride (52 mg, 0.07 mmol), and 1,8-diazabicyclo[5.4.0]undec-7-ene (0.11 g, 0.75 mmol). The reaction mixture was stirred at room temperature for 2.5 h and then poured into water (50 mL). The organic layer was separated and the aqueous layer was extracted with AcOEt. The combined organic layer was washed with water (50 mL) and then dried over $MgSO_4$. The solvent

was removed under reduced pressure. The residue was purified by column chromatography on silica gel eluting with chloroform-*n*-hexane (5:1) to give *N*-[(3-(phenylselanylmethyl)-1-tosylpyrrole-4-yl)methyl]-*N*-cinnamyl-*p*-toluenesulfonamide (**6d**) (0.314 mg, 78%) as white powders.

mp 91–93 ^{o}C, IR ν 1371, 1341, 1161 (SO_2); ^{1}H NMR (600 MHz, $CDCl_3$) δ 2.39 (3H, s, Me), 2.42 (3H, s, Me), 3.81 (2H, s, CH_2), 3.85 (2H, d, *J* = 6.8 Hz, CH_2), 4.19 (2H, s, CH_2), 5.72 (1H, dt, *J* = 15.8 and 6.9 Hz, olefinic H), 6.20 (1H, d, *J* = 15.8 Hz, olefinic H), 6.71 (1H, d, *J* = 2.1 Hz, ArH), 6.89 (1H, d, *J* = 2.1 Hz, ArH), 7.10-7.12 (4H, m, ArH), 7.20 (1H, t, *J* = 6.8 Hz, ArH), 7.22-7.25 (4H, m, ArH), 7.26-7.30 (5H, m, ArH), 7.58 (2H, d, *J* = 8.2 Hz, ArH), 7.72 (2H, d, *J* = 8.2 Hz, ArH); ^{13}C NMR (150 MHz, $CDCl_3$) δ 21.3 (q), 21.5 (q), 21.6 (q), 41.9 (t), 48.9 (t), 119.7 (d), 120.5 (d), 122.2 (s), 123.2 (d), 124.8 (s), 126.3 (d × 2), 126.7 (d × 2), 127.2 (d × 2), 127.3 (d), 127.8 (d), 128.5 (d × 2), 128.8 (d × 2), 129.6 (s), 129.7 (d × 2), 129.9 (d × 2), 133.9 (d × 2), 134.2 (d), 135.7 (s), 136.0 (s), 136.7 (s), 143.5 (s), 145.0 (s); MS (EI) m/z 535 (M^{+}-Ts). Anal. Calcd for $C_{35}H_{34}N_2O_4S_2Se$+1/2H_2O: C, 60.16; H, 5.05; N, 4.01. Found: C, 59.86; H, 5.07; N, 3.80.

N-[(3-(Phenylselanylmethyl)-1-tosylpyrrole-4-yl)methyl]-*N*-(3-methyl-2-butenyl)-*p*-toluene sulfonamide (**6e**).

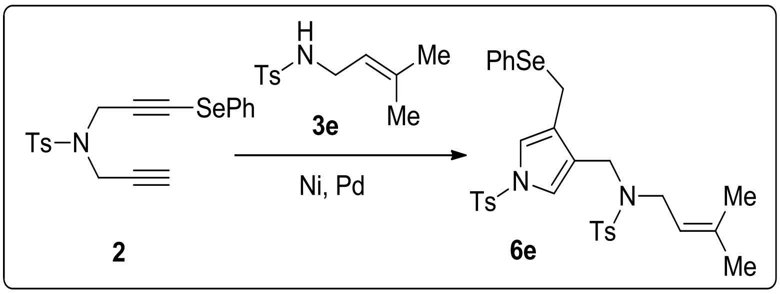

To a DMSO (0.5 mL) solution of *N*-(phenylselanylprop-2-ynyl) -*N*-prop-2-ynyl-p-toluenesulfonamide (**2**) (50 mg, 0.12 mmol) were added *N*-(3-methyl-2-butenyl)-*p*-toluenesulfonamide (**3e**) (53 mg, 0.25 mmol), bis(hexafluoroacetylacetonato)nickel(II) hydrate (6 mg, 0.01 mmol), bis(triphenylphosphine)palladium(II) dichloride (9 mg, 0.01 mmol), and 1,8-diazabicyclo[5.4.0]undec-7-ene (19 mg, 0.12 mmol). The reaction mixture was stirred at room temperature for 14 h and then poured into water (50 mL). The organic layer was separated and the aqueous layer was extracted with AcOEt. The combined organic layer was washed with water (50 mL) and then dried over $MgSO_4$. The solvent

was removed under reduced pressure. The residue was purified by column chromatography on silica gel eluting with $CHCl_3$-*n*-hexane (5:1) to give *N*-[(3-(phenylselanylmethyl)-1-tosylpyrrole-4-yl)methyl]-*N*-(3-methyl-2-butenyl)-*p*-toluene-sulfonamide (**6e**) (46 mg, 58%) as yellow oil.

IR ν 1371, 1339, 1172 (SO_2); ^{1}H NMR (600 MHz, $CDCl_3$) δ 1.35 (3H, s, Me), 1.53 (3H, s, Me), 2.41 (3H, s, Me), 2.43 (3H, s, Me), 3.70 (2H, d, *J* = 6.9 Hz, CH_2), 3.84 (2H, s, CH_2), 4.14 (2H, s, CH_2), 4.77 (1H, t, *J* = 6.9 Hz, olefinic H), 6.71 (1H, d, *J* = 2.0 Hz, ArH), 6.83 (1H, d, *J* = 2.1 Hz, ArH), 7.15 (2H, t, *J* = 7.6 Hz, ArH), 7.22 (1H, t, *J* = 7.6 Hz, ArH), 7.25 (2H, d, *J* = 6.2 Hz, ArH), 7.28 (2H, d, *J* = 8.3 Hz, ArH), 7.33 (2H, d, *J* = 6.9 Hz, ArH), 7.59 (2H, d, *J* = 8.3 Hz, ArH), 7.68 (2H, d, *J* = 8.2 Hz, ArH); ^{13}C NMR (150 MHz, $CDCl_3$) δ 17.5 (q), 21.4 (t), 21.5 (q), 21.6 (q), 25.7 (q), 41.9 (t), 44.6 (t), 118.3 (d), 119.6 (d), 120.3 (d), 122.5 (s), 124.9 (s), 126.8 (d × 2), 127.2 (d × 2), 127.4 (d), 128.8 (d × 2), 129.6 (d × 2), 129.8 (s), 129.9 (d × 2), 134.0 (d × 2), 135.8 (s), 136.7 (s), 136.9 (s), 143.2 (s), 145.0 (s); MS (ESI-TOF) m/z 665 $[M + Na]^+$. HRMS (ESI-TOF) m/z: $[M+Na]^+$ Calcd for $C_{31}H_{34}N_2O_4S_2SeNa$ 665.1023; found 665.1025.

N-[(3-(Phenylselanylmethyl)-1-tosylpyrrole-4-yl)methyl]-*N*-(3-butenyl)-*p*-toluenesulfonamide (**6f**).

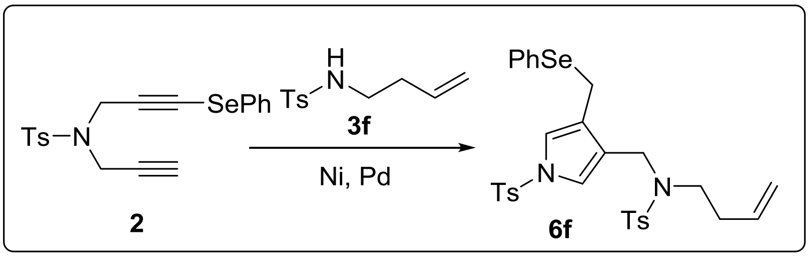

To a DMSO (0.5 mL) solution of *N*-(phenylselanylprop-2-ynyl)-*N*-prop-2-ynyl-p-toluenesulfonamide (**2**) (50 mg, 0.12 mmol) were added *N*-(3-butenyl)-*p*-toluenesulfonamide (**3f**) (56 mg, 0.25 mmol), bis(hexafluoroacetylacetonato)nickel(II) hydrate (6 mg, 0.01 mmol), bis(triphenylphosphine)palladium(II) dichloride (9 mg, 0.01 mmol), and 1,8-diazabicyclo[5.4.0]undec-7-ene (19 mg, 0.12 mmol). The reaction mixture was stirred at room temperature for 15 h and then poured into water (50 mL). The organic layer was separated and the aqueous layer was extracted with AcOEt. The combined organic layer was washed with water (50 mL) and then dried over $MgSO_4$. The solvent was removed under reduced pressure. The residue was purified by column

chromatography on silica gel eluting with chloroform-*n*-hexane (5:1) to give *N*-[(3-(phenylselanylmethyl)-1-tosylpyrrole-4-yl)methyl]-*N*-(3-butenyl)-*p*-toluenesulfon-amide (**6f**) (52 mg, 67%) as white powder.

mp 76–78 °C, IR ν 1371, 1339, 1172 (SO_2); ^{1}H NMR (600 MHz, $CDCl_3$) δ 1.99 (2H, dd, *J* = 6.8 and 15.1 Hz, CH_2), 2.40 (3H, s, Me), 2.43 (3H, s, Me), 3.04 (2H, brt, *J* = 8.2 Hz, CH_2), 3.85 (2H, s, CH_2), 4.13 (2H, s, CH_2), 4.81 (1H, dd, *J* = 1.3 and 17.1 Hz, olefinic H), 4.87 (1H, brd, *J* =10.3 Hz, olefinic H), 5.41-5.48 (1H, m, olefinic H), 6.73 (1H, d, *J* = 2.8 Hz, ArH), 6.88 (1H, d, *J* = 2.7 Hz, ArH), 7.14-7.17 (2H, m, ArH), 7.21-7.26 (3H, m, ArH), 7.29 (2H, d, *J* = 8.3 Hz, ArH), 7.33 (2H, brd, *J* = 6.8 Hz, ArH), 7.58 (2H, d, *J* = 8.3 Hz, ArH), 7.68 (2H, d, *J* = 8.2 Hz, ArH); ^{13}C NMR (150 MHz, $CDCl_3$) δ 21.4 (t), 21.5 (q), 21.6 (q), 33.0 (t), 43.6 (t), 47.4 (t), 116.7 (t), 119.8 (d), 120.3 (d), 122.7 (s), 124.8 (s), 126.7 (d × 2), 127.2 (d × 2), 127.4 (d), 128.8 (d × 2), 129.6 (s), 129.7 (d × 2), 129.9 (d × 2), 134.0 (d × 2), 134.5 (d), 135.7 (s), 136.2 (s), 143.4 (s), 145.0 (s); MS (ESI-TOF) m/z 651 $[M + Na]^+$. HRMS (ESI-TOF) m/z: $[M+Na]^+$ Calcd for $C_{30}H_{32}N_2O_4S_2SeNa$ 651.0866; found 651.0874. Anal. Calcd for $C_{30}H_{32}N_2O_4S_2Se$: C, 57.41; H, 5.14; N, 4.46. Found: C, 57.17; H, 5.00; N, 4.27.

N-[(3-(Phenylsulfinylmethyl)-1-tosylpyrrole-4-yl)methyl]-*N*-methallyl-*p*-toluenesulfonamide (**5b**).

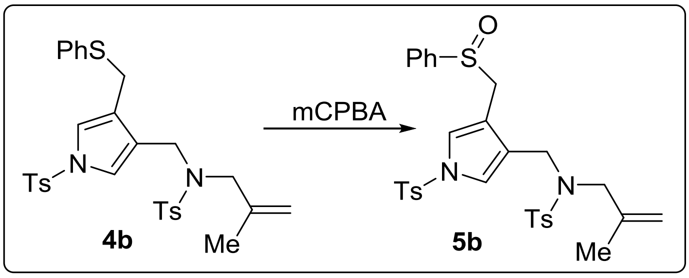

m-Chloroperbenzoic acid (71 mg, 0.41 mmol) was added over 0.5 h to a 1,2-dichloroethane (10.0 mL) solution of *N*-[(3-(phenylsulfanylmethyl)-1-tosylpyrrole-4-yl)methyl]-*N*-methallyl-*p*-toluenesulfonamide (**4b**) (238 mg, 0.41 mmol) at 0 °C. The reaction mixture was further stirred for 10 min and poured into a sat. $NaHCO_3$ (50 mL). The whole was vigorously stirred for 15 min. The organic layer was separated and the aqueous layer was extracted with chloroform. The combined organic layer was washed with a sat. $NaHCO_3$ (50 mL) and then dried over $MgSO_4$. The solvent was removed under reduced pressure. The residue was purified by preparative TLC on silica gel eluting with AcOEt-*n*-hexane (1:2) to give

N-[(3-(phenylsulfinylmethyl)-1-tosylpyrrole-4-yl)methyl]-*N*-methallyl-*p*-toluenesulfonamide (**5b**) (196 mg, 80%) as white powder.

mp 154–158 °C, IR ν 1372, 1335, 1173 (SO_2), 1092 (SO); ^{1}H NMR (600 MHz, $CDCl_3$) δ 1.36 (3H, s, Me), 2.43 (6H, s, Me × 2), 3.51 (2H, s, CH_2), 3.73 (1H, d, *J* = 15.1 Hz, CH), 3.80 (1H, d, *J* = 15.1 Hz, CH), 3.83 (1H, d, *J* = 13.8 Hz, CH), 4.08 (1H, d, *J* = 13.8 Hz, CH), 4.54 (1H, s, olefinic H), 4.62 (1H, s, olefinic H), 6.78 (1H, s, ArH), 6.94 (1H, d, *J*=2.1 Hz, ArH), 7.28-7.37 (8H, m, ArH), 7.42 (1H, t, *J* = 6.9 Hz, ArH), 7.61 (2H, d, *J* = 7.6 Hz, ArH), 7.70 (2H, d, *J* = 7.5 Hz, ArH); ^{13}C NMR (150 MHz, $CDCl_3$) δ 19.8 (q), 21.5 (q), 21.6 (q), 42.6 (t), 52.6 (t), 53.8 (t), 113.9 (t), 115.8 (s), 120.7 (d), 121.7 (d), 122.9 (s), 124.3 (d × 2), 127.0 (d × 2), 127.1 (d × 2), 128.7 (d × 2), 129.7 (d × 2), 130.0 (d × 2), 130.9 (d), 135.6 (s), 136.2 (s), 140.3 (s), 142.8 (s), 143.6 (s), 145.3 (s); MS (EI) m/z 471 (M^+-PhSO), 316 (M^+-PhSO-Ts); MS (ESI-TOF) m/z 619 $[M + Na]^+$. HRMS (ESI-TOF) m/z: $[M+Na]^+$ Calcd for $C_{30}H_{32}N_2O_5S_2S_3Na$ 619.1371; found 619.1344. Anal. Calcd for $C_{30}H_{32}N_2O_5S_3$+1/2H_2O: C, 59.48; H, 5.49; N, 4.62. Found: C, 59.33; H, 5.55; N, 4.57.

(*E*)-*N*-(But-2-enyl)-*N*-[(3-(phenylsulfinylmethyl)-1-tosylpyrrol-4-yl)methyl]-*N*-*p*-toluene-sulfonamide (**5c**).

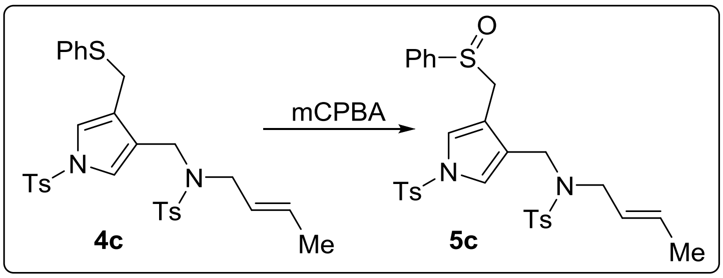

m-Chloroperbenzoic acid (86 mg, 0.50 mmol) was portionwise added over 1 h to a 1,2-dichloroethane (15.0 mL) solution of *N*-[(3-(phenylsulfanylmethyl)-1-tosylpyrrole-4-yl)methyl]-*N*-(but-2-enyl)-*p*-toluenesulfonamide (**4c**) (290 mg, 0.50 mmol) at 0 °C. The reaction mixture was further stirred for 5 min and poured into a sat. $NaHCO_3$ (50 mL). The whole was vigorously stirred for 15 min. The organic layer was separated and the aqueous layer was extracted with chloroform. The combined organic layer was washed with a sat. $NaHCO_3$ (50 mL) and then dried over $MgSO_4$. The solvent was removed under reduced pressure. The residue was purified by preparative TLC on silica gel eluting with AcOEt-*n*-hexane (1:2) to give (*E*)-*N*-(but-2-enyl)-*N*-[(3-(phenylsulfinylmethyl)-1-tosylpyrrol-4-yl)methyl]-*N*-*p*-toluene-sulfonamide (**5c**) (230 mg, 77%) as white powder.

mp 143–146 °C. IR ν 1372, 1335 (SO_2), 1091 (SO); ^{1}H NMR (600 MHz, $CDCl_3$) δ 1.48 (3H, d, *J* = 6.9 Hz, Me), 2.43 (3H, s, Me), 2.44 (3H, s, Me), 3.51 (1H, dd, *J* = 6.9 and 15.8 Hz, CH), 3.57 (1H, dd, *J* = 6.9 and 15.1 Hz, CH), 3.78 (1H, d, *J* = 15.1 Hz, CH), 3.80 (1H, d, *J* = 13.8 Hz, CH), 3.84 (1H, d, *J* = 13.8 Hz, CH), 4.11 (1H, d, *J* = 13.7 Hz, CH), 4.88-4.93 (1H, m, olefinic H), 5.23-5.29 (1H, m, olefinic H), 6.86 (1H, d, *J* = 2.1 Hz, ArH), 7.06 (1H, d, *J* = 2.1 Hz, ArH), 7.30 (2H, d, *J* = 8.3 Hz, ArH), 7.33-7.36 (4H, m, ArH), 7.42-7.44 (3H, m, ArH), 7.62 (2H, d, *J* = 8.2 Hz, ArH), 7.74 (2H, d, *J* = 8.2 Hz, ArH); ^{13}C NMR (150 MHz, $CDCl_3$) δ 17.6 (q), 21.5 (q), 21.7 (q), 41.5 (t), 48.7 (t), 52.8 (t), 116.2 (s), 120.4 (d), 122.3 (d), 122.6 (s), 124.2 (d), 124.4 (d × 2), 127.0 (d × 2), 127.2 (d × 2), 128.7 (d × 2), 129.7 (d × 2), 130.1 (d × 2), 130.8 (d), 130.9 (d), 135.6 (s), 136.6 (s), 143.0 (s), 143.5 (s), 145.4 (s); MS (EI) m/z 471 (M^+-PhSO). MS (ESI-TOF) m/z 619 $[M + Na]^+$. HRMS (ESI-TOF) m/z: $[M+Na]^+$ Calcd for $C_{30}H_{32}N_2O_5S_3Na$ 619.1371; found 619.1384. Anal. Calcd for $C_{30}H_{32}N_2O_5S_3$+1/4H_2O: C, 59.93; H, 5.45; N, 4.66. Found: C, 59.85; H, 5.21; N, 4.61.

4-(*N*-Cinnamyl-*N*-tosylaminomethyl)-3-(phenylsulfinylmethyl)-*N*-tosylpyrrole (**5d**).

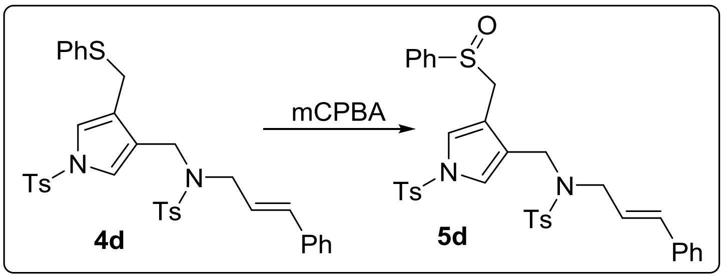

m-Chloroperbenzoic acid (94 mg, 0.55 mmol) was portionwise added over 1 h to a 1,2-dichloroethane (15.0 mL) solution of 4-(*N*-cinnamyl-*N*-tosylaminomethyl)-3-(phenylsulfanylmethyl)-*N*-tosylpyrrole (**4d**) (351 mg, 0.55 mmol) at 0 °C. The reaction mixture was further stirred for 5 min and poured into a sat. $NaHCO_3$ (50 mL). The whole was vigorously stirred for 15 min. The organic layer was separated and the aqueous layer was extracted with chloroform. The combined organic layer was washed with a sat. $NaHCO_3$ (50 mL) and then dried over $MgSO_4$. The solvent was removed under reduced pressure. The residue was purified by preparative TLC on silica gel eluting with AcOEt-*n*-hexane (1:2) to give 4-(*N*-cinnamyl-*N*-tosylaminomethyl)-3-(phenylsulfinylmethyl)-*N*-tosylpyrrole (**5d**) (301 mg, 84%) as white powder.

mp 50–53 °C, IR ν 1372, 1337, 1171 (SO_2), 1067 (SO); ^{1}H NMR (600 MHz, $CDCl_3$) δ 2.42 (3H, s, Me), 2.45 (3H, s, Me), 3.72 (1H, dd, *J* = 6.9 and 15.8 Hz, CH), 3.79–3.87 (4H, m,

CH × 4), 4.09 (1H, d, J = 13.8 Hz, CH), 5.56 (1H, dt, J = 6.8 and 15.8 Hz, olefinic H), 6.16 (1H, d, J = 15.8 Hz, olefinic H), 6.91 (1H, d, J = 2.1 Hz, ArH), 7.06 (1H, d, J = 2.1 Hz, ArH), 7.07–7.08 (2H, m, ArH), 7.23–7.28 (4H, m, ArH), 7.30–7.33 (5H, m, ArH), 7.39-7.43 (3H, m, ArH), 7.67 (2H, d, J=8.3 Hz, ArH), 7.73 (2H, d, J=8.9 Hz, ArH); ^{13}C NMR (150 MHz, $CDCl_3$) δ 21.5 (q), 21.6 (q), 41.8 (t), 48.8 (t), 52.7 (t), 116.0 (s), 120.4 (d), 122.4 (d), 122.5 (s), 122.6 (d), 124.4 (d × 2), 126.3 (d × 2), 127.0 (d × 2), 127.2 (d × 2), 128.0 (d), 128.6 (d × 2), 128.7 (d × 2), 129.8 (d × 2), 130.1 (d × 2), 130.8 (d), 134.4 (d), 135.5 (s), 135.9 (s), 136.5 (s), 142.9 (s), 143.7 (s), 145.4 (s); MS (EI) m/z 533 (M^+-SOPh); MS (ESI-TOF) m/z 681 $[M + Na]^+$. HRMS (ESI-TOF) m/z: $[M+Na]^+$ Calcd for $C_{35}H_{34}N_2O_5S_3Na$ 681.1528; found 681.1537.

N-[(3-(Phenylsulfinylmethyl)-1-tosylpyrrole-4-yl)methyl]-*N*-(3-methyl-2-butenyl)-*p*-toluenesulfonamide (**5e**).

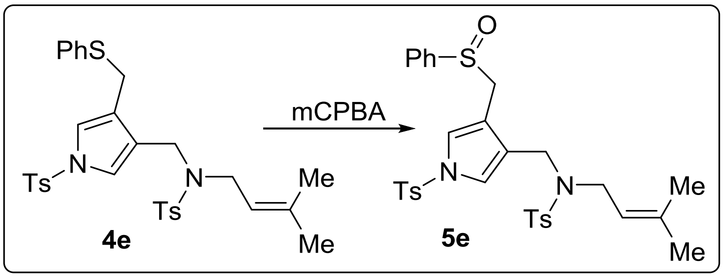

m-Chloroperbenzoic acid (14 mg, 0.08 mmol) was added over 15 min to a 1,2-dichloroethane (3.0 mL) solution of *N*-[3-(phenylsulfanylmethyl)-1-tosylpyrrole-4-methyl]-*N*-(3-methyl-2-butenyl)-*p*-toluenesulfonamide (**4e**) (47 mg, 0.08 mmol) at 0 °C. The reaction mixture was further stirred for 15 min and poured into a sat. $NaHCO_3$ (50 mL). The whole was vigorously stirred for 15 min. The organic layer was separated and the aqueous layer was extracted with chloroform. The combined organic layer was washed with a sat. $NaHCO_3$ (50 mL) and then dried over $MgSO_4$. The solvent was removed under reduced pressure. The residue was purified by preparative TLC on silica gel eluting with AcOEt-*n*-hexane (1:2) to give *N*-[(3-(phenylsulfinylmethyl)-1-tosylpyrrole-4-yl)-methyl]-*N*-(3-methyl-2-butenyl)-*p*-toluenesulfonamide (**5e**) (40 mg, 83%) as white powder. mp 62 – 64 ^{o}C, IR ν 1373, 1338, 1159 (SO_2), 1068 (SO); ^{1}H NMR (600 MHz, $CDCl_3$) δ 1.36 (3H, s, Me), 1.50 (3H, s, Me), 2.43 (3H, s, Me), 2.45 (3H, s, Me), 3.55-3.65 (2H, m, CH_2), 3.76 (1H, d, J = 14.5 Hz, CH), 3.82 (1H, d, J = 14.5 Hz, CH), 3.84 (1H, d, J = 13.7 Hz, CH), 4.13 (1H, d, J = 13.8 Hz, CH), 4.59-4.61 (1H, m, olefinic H), 6.84 (1H, d, J = 2.0 Hz, ArH), 7.08 (1H, d, J = 2.0 Hz, ArH), 7.30 (2H, d, J = 8.3 Hz, ArH), 7.33 (2H, d, J = 8.2

Hz, ArH), 7.36 (2H, d, *J* = 7.6 Hz, ArH), 7.42–7.45 (3H, m, ArH), 7.62 (2H, d, *J* = 8.2 Hz, ArH), 7.74 (2H, d, *J* = 8.3 Hz, ArH); ^{13}C NMR (150 MHz, $CDCl_3$) δ 17.5 (q), 21.5 (q), 21.6 (q), 25.6 (q), 41.7 (t), 44.4 (t), 52.7 (t), 116.1 (s), 117.7 (d), 120.2 (d), 122.2 (d), 122.7 (s), 124.3 (d × 2), 127.0 (d × 2), 127.1 (d × 2), 128.7 (d × 2), 129.6 (d × 2), 130.0 (d × 2), 130.8 (d), 135.5 (s), 136.6 (s × 2), 143.0 (s), 143.4 (s), 145.3 (s); MS (ESI-TOF) m/z 633 $[M + Na]^+$; HRMS (ESI-TOF) m/z: $[M+Na]^+$ Calcd for $C_{31}H_{34}N_2O_5S_3Na$ 633.1528; found 633.1518. Anal. Calcd for $C_{31}H_{34}N_2O_5S_3$+1/3H_2O: C, 60.39; H, 5.67; N, 4.54. Found: C, 60.48; H, 5.66; N, 4.54.

N-[(3-(Phenylsulfinylmethyl)-1-tosylpyrrole-4-yl)methyl]-*N*-(3-butenyl)-*p*-toluenesulfonamide (**5f**).

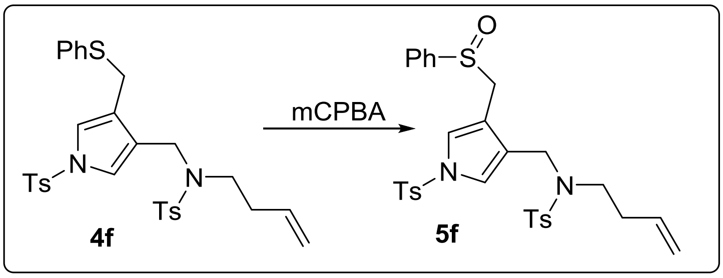

m-Chloroperbenzoic acid (54 mg, 0.31 mmol) was added over 0.5 h to a 1,2-dichloroethane (11.0 mL) solution of *N*-[(3-(phenylsulfanylmethyl)-1-tosylpyrrole-4-yl)methyl]-*N*-(3-butenyl)-*p*-toluenesulfonamide (**4f**) (181 mg, 0.31 mmol) at 0 °C. The reaction mixture was further stirred for 15 min and poured into a sat. $NaHCO_3$ (50 mL). The whole was vigorously stirred for 15 min. The organic layer was separated and the aqueous layer was extracted with chloroform. The combined organic layer was washed with a sat. sodium hydrogen carbonate (50 mL) and then dried over $MgSO_4$. The solvent was removed under reduced pressure. The residue was purified by preparative TLC on silica gel eluting with AcOEt-*n*-hexane (1:2) to give *N*-[(3-(phenylsulfinylmethyl)-1-tosylpyrrole-4-yl)methyl]-*N*-(3-butenyl)-*p*-toluenesulfonamide (**5f**) (174 mg, 94%) as white powders.

mp 126–129 ^{o}C, IR ν 1373, 1334, 1161 (SO_2), 1066 (SO); ^{1}HNMR (600 MHz, $CDCl_3$) δ 1.75–1.95 (2H, m, CH_2), 2.42 (3H, s, Me), 2.44 (3H, s, Me), 2.92-2.98 (2H, m, CH_2), 3.73 (1H, d, *J* = 14.5 Hz, CH), 3.79 (1H, d, *J* = 14.4 Hz, CH), 3.84 (1H, d, *J* = 13.8 Hz, CH), 4.14 (1H, d, *J* = 13.7 Hz, CH), 4.76 (1H, dd, *J* = 1.4 and 17.2 Hz, olefinic H), 4.85 (1H, d, *J* = 10.3 Hz, olefinic H), 5.35–5.42 (1H, m, olefinic H), 6.92 (1H, d, *J* = 2.7 Hz, ArH), 7.08 (1H, d, *J* = 2.0 Hz, ArH), 7.31 (4H, t, *J* = 6.9 Hz, ArH), 7.35 (2H, br t, *J* = 8.3 Hz, ArH), 7.43

(3H, t, J = 8.2 Hz, ArH), 7.63 (2H, d, J = 8.3 Hz, ArH), 7.73 (2H, d, J = 8.2 Hz, ArH); ^{13}C NMR (150 MHz, $CDCl_3$) δ 21.5 (q), 21.6 (q), 32.9 (t), 43.8 (t), 47.4 (t), 52.6 (t), 116.1 (s), 116.8 (t), 120.0 (d), 122.5 (d), 123.1 (s), 124.3 (d × 2), 126.9 (d × 2), 127.1 (d × 2), 128.7 (d × 2), 129.8 (d × 2), 130.1 (d × 2), 130.8 (d × 2), 134.3 (d), 135.5 (s), 135.7 (s), 142.9 (s), 143.6 (s), 145.4 (s); MS (ESI) m/z (ESI-TOF) 619 $[M + Na]^+$; HRMS (ESI-TOF) m/z: $[M+Na]^+$ Calcd for $C_{30}H_{32}N_2O_5S_3Na$ 619.1371; found 617.1377. Anal. Calcd for $C_{30}H_{32}N_2O_5S_3$+1/3H_2O: C, 59.78; H, 5.46; N, 4.65. Found: C, 59.90; H, 5.33; N, 4.71.

Typical procedure for Pummerer reaction of **5a** with TFAA and successive treatment with TBAH, synthesis of diol **9a**.

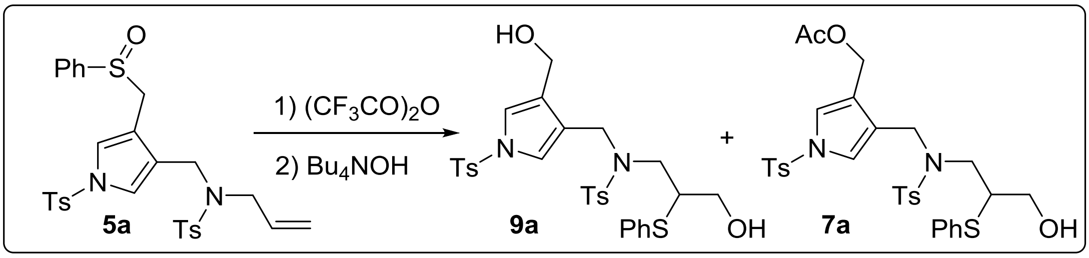

Trifluoroacetic anhydride (180 mg, 0.85 mmol) was added dropwise to a dichloromethane (2.0 mL) solution of *N*-allyl-*N*-[(3-(phenylsulfinylmethyl)-1-tosylpyrrole-4-yl)-methyl]-*p*-toluenesulfonamide (**5a**) (50 mg, 0.09 mmol) at −20 °C. The reaction mixture was stirred for 0.5 h and poured into a sat. sodium hydrogen carbonate (50 mL). The organic layer was separated and the aqueous layer was extracted with chloroform. The combined organic layer was dried over $MgSO_4$. The solvent was removed under reduced pressure. To the residue in dichloromethane (2.5 mL) were added tetrabutylammonium hydroxide (0.22 g, 0.86 mmol) in H_2O (2 mL) and tetrabutylammonium hydrogensulfate (5.8 mg, 1.7 × 10^{-2} mmol) at room temperature. The mixture was stirred for 10 h and poured into water (50 mL). The organic layer was separated and the aqueous layer was extracted with chloroform. The combined organic layer was dried over $MgSO_4$. The solvent was removed under reduced pressure. The residue was purified by preparative TLC on silica gel eluting with AcOEt-*n*-hexane (1:2) to give *N*-(3-hydroxy-2-(phenylthio)propyl)-*N*-((4-(hydroxymethyl)-1-tosyl-1*H*-pyrrol-3-yl)methyl)-4-methylbenzenesulfonamide (**9a**) (48 mg, 93 %) as white powders (entry 10, Table 1).

mp 57–61 ^{o}C, IR ν 3432 (OH), 1369, 1338, 1172 (SO_2), 1067 (SO); ^{1}H NMR (600 MHz, $CDCl_3$) δ 2.37 (3H, s, Me), 2.43 (3H, s, Me), 2.96 (1H, dd, *J* = 4.8 and 14.4 Hz, CH), 3.05 (1H, dd, *J* = 4.8 and 9.6 Hz, CH), 3.47 (1H, dd, *J* = 9.6 and 14.4 Hz, CH), 3.54 (1H, dd, *J* = 3.4 and 12.4 Hz, CH), 3.66 (1H, dd, *J* = 3.4 and 12.4 Hz, CH), 3.87 (1H, d, *J* = 14.4 Hz, CH), 4.28 (1H, d, *J* = 14.4 Hz, CH), 4.48 (1H, d, *J* = 13.1 Hz, CH), 4.53 (1H, d, *J* = 13.0 Hz, CH), 6.88 (1H, d, *J* = 2.8 Hz, ArH), 7.09 (1H, d, *J* = 2.1 Hz, ArH), 7.21 (1H, brd, *J* = 6.8 Hz, ArH), 7.25-7.27 (3H, m, ArH), 7.29 (4H, brd, *J* = 7.6 Hz, ArH), 7.60 (2H, d, *J* = 8.2 Hz, ArH), 7.70 (2H, d, *J* = 8.3 Hz, ArH); ^{13}C NMR (150 MHz, $CDCl_3$) δ 21.5 (q), 21.6 (q), 46.3 (t), 50.2 (t), 50.3 (d), 56.2 (t), 61.4 (t), 119.9 (d), 120.9 (d), 122.1 (s), 126.9 (d × 2), 127.3 (d × 2), 127.4 (d), 128.0 (s), 129.2 (d × 2), 130.0 (d × 2), 130.1 (d × 2), 131.9 (d × 2), 133.3 (s), 134.4 (s), 135.6 (s), 144.2 (s), 145.4 (s); MS (EI) m/z 600 (small M^+), 445 (M^+-Ts). MS (ESI-TOF) m/z 623 $[M + Na]^+$; HRMS (ESI-TOF) m/z: $[M+Na]^+$ Calcd for $C_{29}H_{32}N_2O_6S_3Na$ 623.13202; found 623.12954. Anal. Calcd for $C_{29}H_{32}N_2O_6S_3$+1/2H_2O: C, 57.12; H, 5.46; N, 4.59. Found: C, 57.01; H, 5.25; N, 4.41.

3-Acetoxymethyl-4-(*N*-allyl-*N*-tosylaminomethyl)-1-(tosyl)pyrrole (**7a**), entry 1, Table 1.

To an acetic acid (0.50 mL) solution of **5a** (20 mg, 0.03 mmol) solution was added acetic anhydride (17.5 mg, 0.17 mmol) at room temperature. The reaction mixture was heated at 120 ^{o}C for 6 h and then cooled mixture was poured into water (50 mL). The organic layer was separated and the aqueous layer was extracted with AcOEt. The combined organic layer was washed with water (50 mL) and then saturated sodium hydrogen carbonate (50 mL) and dried over $MgSO_4$. The solvent was removed under reduced pressure. The residue was purified by preparative TLC on silica gel eluting with EtOAc-*n*-hexane (1:3) to give the tilted compound **7a** (14 mg, 86%) as a yellow oil.

IR ν 1739 (CO), 1374, 1172, 1068 (SO_2); ^{1}H NMR (600 MHz, $CDCl_3$) δ 2.03 (3H, s, Me), 2.41 (3H, s, Me), 2.44 (3H, s, Me), 3.68 (2H, d, *J* = 6.8 Hz, CH_2), 4.16 (2H, s, CH_2), 4.86 (1H, dd, *J* = 1.4 and 17.2 Hz, olefinic H), 4.89 (1H, s, CH_2), 4.96-4.98 (1H, m, olefinic H), 5.35-5.42 (1H, m, olefinic H), 6.91 (1H, d, *J* = 2.3 Hz, ArH), 7.12 (1H, d, *J* = 2.3 Hz, ArH), 7.30 (2H, d, *J* = 7.4 Hz, ArH), 7.30 (2H, d, *J* = 8.0 Hz, ArH), 7.68 (2H, d, *J* = 8.3 Hz, ArH), 7.73 (2H, d, *J* = 8.2 Hz, ArH); ^{13}C NMR (150 MHz, $CDCl_3$) δ 20.9 (q), 21.5 (q), 21.6 (q), 41.9 (t), 49.6 (t), 57.7 (t), 119.0 (t), 120.6 (d), 121.0 (d), 122.4 (s), 122.7 (s), 127.0 (d × 2), 127.2 (d × 2), 129.7 (d × 2), 130.1 (d × 2), 132.3 (d), 135.7 (s), 136.8 (s), 143.5 (s), 145.3

(s), 170.6 (s); MS (EI) m/z 516 (small M^+). MS (ESI-TOF) m/z 539 $[M + Na]^+$; HRMS (ESI-TOF) m/z: $[M+Na]^+$ Calcd for $C_{25}H_{28}N_2O_6S_2Na$ 539.1287; Found 539.1278.

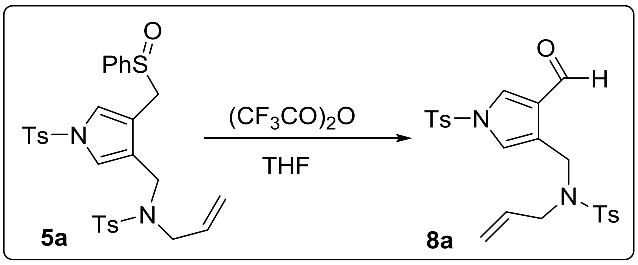

4-(*N*-Allyl-*N*-tosylaminomethyl)-1-(tosyl)pyrrol-3-carboxaldehyde (**8a**), entry 7, Table 1.

To a THF (0.50 mL) solution of **5a** (20 mg, 0.03 mmol) was added trifluoroacetic anhydride (36 mg, 0.10 mL, 0.17 mmol) at −20 °C. The reaction mixture was stirred for 0.5 h then poured into a saturated sodium hydrogen carbonate (50 mL). The organic layer was separated and the aqueous layer was extracted with AcOEt. The combined organic layer was dried over $MgSO_4$. The solvent was removed under reduced pressure. The residue was purified by preparative TLC on silica gel eluting with EtOAc-*n*-hexane (1:3) to give the titled compound (14 mg, 86%) as colorless prisms (mp 109-110 ^oC).

IR ν 1683 (CO), 1381, 1344, 1175, 1063 (SO_2); ^1H NMR (600 MHz, $CDCl_3$) δ 2.43 (3H, s, Me), 2.44 (3H, s, Me), 3.81 (2H, d, J = 6.2 Hz, CH_2), 4.42 (2H, s, CH_2), 4.94-4.99 (2H, m, olefinic H), 5.48-5.52 (1H, m, olefinic H), 7.18 (1H, s, ArH), 7.28 (2H, d, J = 8.2 Hz, ArH), 7.36 (2H, d, J = 8.2 Hz, ArH), 7.67 (2H, d, J = 8.3 Hz, ArH), 7.69 (1H, d, J = 4.0 Hz, ArH), 7.81 (2H, d, J = 8.3 Hz, ArH), 9.76 (1H, s, CHO); ^{13}C NMR (150 MHz, $CDCl_3$) δ 21.5 (q), 21.7 (q), 43.1 (t), 51.4 (t), 119.1 (t), 121.5 (d), 124.6 (s), 126.8 (s), 127.2 (d × 2), 127.4 (d × 2), 129.7 (d × 2), 129.8 (d), 130.4 (d × 2), 132.3 (d), 134.7 (s), 136.6 (s), 143.5 (s), 146.3 (s), 185.8 (d); MS (ESI-TOF) m/z 495 $[M + Na]^+$; HRMS (ESI-TOF) m/z: $[M+Na]^+$ Calcd for $C_{23}H_{24}N_2O_5S_2Na$ 495.1024; Found 495.1019.

Pummerer reaction of pyrrole **5b** with TFAA and successive treatment with TBAH.

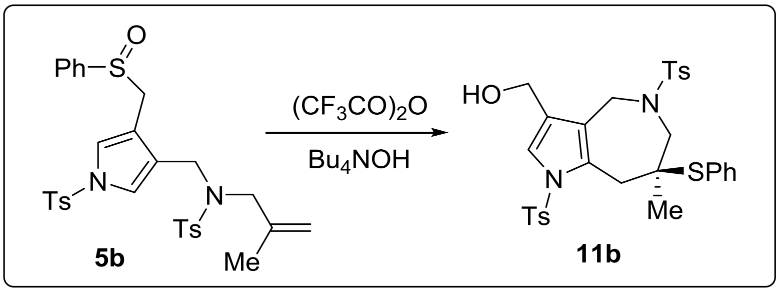

Trifluoroacetic anhydride (88 mg (0.10 mL), 0.42 mmol) was added dropwise to a dichloromethane (2.0 mL) solution of *N*-methallyl-*N*-[(3-(phenylsulfinylmethyl)-1-tosylpyrrole-4-yl)methyl]-*p*-toluenesulfonamide (**5b**) (50 mg, 0.08 mmol) at −20 °C. The reaction mixture was stirred for 0.5 h and poured into a sat. $NaHCO_3$ (50 mL). The organic layer was separated and the aqueous layer was extracted with chloroform. The combined organic layer was dried over $MgSO_4$. The solvent was removed under reduced pressure to give the residue. To the residue in dichloromethane (2.5 mL) were added tetrabutylammonium hydroxide (0.22 g, 0.80 mmol) in H_2O (2.0 mL) and tetrabutylammonium hydrogensulfate (5.7 mg, 1.6×10^{-2} mmol) at room temperature. The mixture was stirred for 10 h and poured into water (50 mL). The organic layer was separated and the aqueous layer was extracted with $CHCl_3$. The combined organic layer was dried over $MgSO_4$. The solvent was removed under reduced pressure. The residue was purified by preparative TLC on silica gel eluting with AcOEt-*n*-hexane (1:2) to give 1,5-bis(*p*-toluenesulfonyl)-1,4,5,6,8-hexahydro-7-methyl-7-(phenylsulfanyl)pyrrolo-[3,2-c]azepin-3-methanol (**11b**) (29 mg, 58%) as white powder.

mp 61–65 ^{o}C, IR ν 3456 (OH), 1363, 1169, 1046 (SO_2), 1091 (SO); ^{1}H NMR (600 MHz, $CDCl_3$) δ 1.33 (3H, s, Me), 1.65 (1H, s, OH), 2.36 (3H, s, Me), 2.40 (3H, s, Me), 2.81 (1H, d, *J* = 11.7 Hz, CH), 3.40 (1H, d, *J* = 12.4 Hz, CH), 3.45 (1H, d, *J* = 11.7 Hz, CH), 3.68 (1H, d, *J* = 13.0 Hz, CH), 3.95 (1H, d, *J* = 14.5 Hz, CH), 4.01 (1H, d, *J* = 14.5 Hz, CH), 4.46 (2H, s, CH_2), 7.17 (1H, t, *J* = 7.5 Hz, ArH), 7.19 (2H, d, *J* = 8.3 Hz, ArH),7.23-7.26 (3H, m, ArH), 7.29 (2H, d, *J* = 8.2 Hz, ArH), 7.34 (2H, d, *J* = 6.8 Hz, ArH), 7.46 (2H, d, *J* = 9.0 Hz, ArH), 7.67 (2H, d, *J* = 8.2 Hz, ArH); ^{13}C NMR (150 MHz, $CDCl_3$) δ 21.5 (q), 21.6 (q), 22.5 (q), 40.2 (s), 40.9 (t), 43.7 (t), 54.9 (t), 56.8 (t), 121.4 (s), 123.0 (s), 123.5 (d), 125.8 (d), 126.3 (d × 2), 127.7 (d × 2), 128.7 (d × 2), 129.3 (d × 2), 129.9 (d × 2), 132.9 (s), 134.5 (s), 136.8 (s), 137.1 (s), 143.7 (s), 144.9 (s); MS (EI) m/z 596 (M^+), 441 (M^+–Ts).

MS (ESI-TOF) m/z 619 [M + Na]$^+$; HRMS (ESI-TOF) m/z: [M+Na]$^+$ Calcd for $C_{30}H_{32}N_2O_5S_3Na$ 619.1371; found 619.1351.

Pummerer reaction of pyrrole **5c** with TFAA and successive treatment with TBAH.

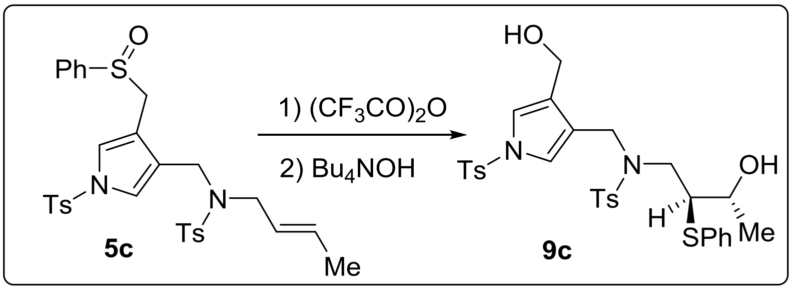

Trifluoroacetic anhydride (88 mg, 0.42 mmol) was added dropwise to a dichloromethane (2.0 mL) solution of *N*-[(3-(phenylsulfinylmethyl)-1-tosylpyrrole-4-yl)methyl]-*N*-(but-2-enyl)-*p*-toluenesulfon- amide (**5c**) (50 mg, 0.08 mmol) at −20 °C. The reaction mixture was stirred for 0.5 h and poured into a sat. $NaHCO_3$ (50 mL). The organic layer was separated and the aqueous layer was extracted with $CHCl_3$. The combined organic layer was dried over $MgSO_4$. The solvent was removed under reduced pressure. To the residue in dichloromethane (2.5 mL) were added tetrabutylammonium hydroxide (0.22 g, 0.80 mmol) in H_2O (2 mL) and tetrabutylammonium hydrogensulfate (5.7 mg, 1.6 × 10^{-2} mmol) at room temperature. The mixture was stirred for 10 h and poured into water (50 mL). The organic layer was separated and the aqueous layer was extracted with $CHCl_3$. The combined organic layer was dried over $MgSO_4$. The solvent was removed under reduced pressure. The residue was purified by preparative TLC on silica gel eluting with AcOEt-*n*-hexane (1:2) to give *N*-(3-hydroxy-2-(phenylthio)butyl)-*N*-((4-(hydroxymethyl)-1-tosyl-1*H*-pyrrol-3-yl)methyl)-4-methylbenzenesulfonamide (**9c**) (33 mg, 64%) as white powder.

mp 53–57 ^oC, IR ν 3431 (OH), 1366, 1342, 1172 (SO_2), 1067 (SO); ^1H NMR (600 MHz, CD_3OD) δ 0.90 (3H, d, *J* = 6.2 Hz, Me), 2.40 (3H, s, Me), 2.44 (3H, s, Me), 3.03 (1H, dd, *J* = 6.8 and 14.5 Hz, CH), 3.30 (1H, dd, *J* = 8.3 and 14.5 Hz, CH), 3.45-3.48 (1H, m, CH), 3.87-3.90 (1H, m, CH), 4.00 (1H, d, *J* = 15.2 Hz, CH), 4.24 (1H, d, *J* = 14.4 Hz, CH), 4.45 (1H, d, *J* = 13.1 Hz, CH), 4.48 (1H, d, *J* = 13.1 Hz, CH), 6.82 (1H, d, *J* = 2.1 Hz, ArH), 7.12 (1H, br s, ArH), 7.27–7.33 (8H, m, ArH), 7.51 (2H, d, *J* = 8.2 Hz, ArH), 7.64 (1H, s, ArH), 7.73 (2H, d, *J* = 8.2 Hz, ArH); ^{13}C NMR (150 MHz, $CDCl_3$) δ 18.6 (q), 21.47 (q), 21.53 (q), 46.1 (t), 49.4 (t), 56.1 (d), 56.2 (t), 66.9 (d), 119.8 (d), 120.6 (d), 121.8 (s), 126.9 (d × 2),

127.3 (d × 3), 128.3 (s), 129.1 (d × 2), 129.8 (d × 2), 130.0 (d × 2), 131.9 (d × 2), 133.7 (s), 134.2 (s), 135.6 (s), 144.0 (s), 145.2 (s); MS (EI) m/z 459 (M^+–Ts). MS (EI) m/z 637 $[M + Na]^+$. HRMS (ESI-TOF) m/z: $[M+Na]^+$ Calcd for $C_{30}H_{34}N_2O_6S_3Na$ 637.1477; found 637.1466.

Pummerer reaction of pyrrole **5d** with TFAA and successive treatment with TBAH.

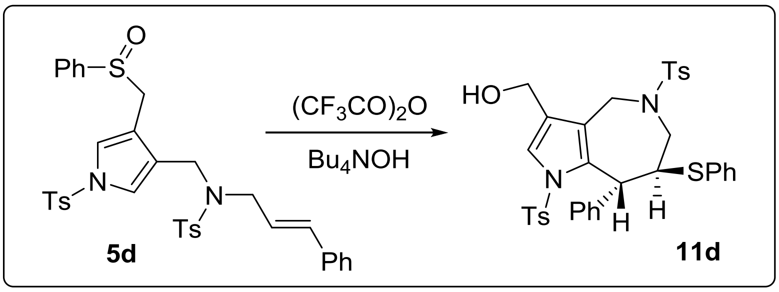

Trifluoroacetic anhydride (0.24 g, 1.14 mmol) was added dropwise to a dichloromethane (2.0 mL) solution of *N*-cinnamyl-*N*-[3-(phenylsulfinylmethyl)-1-tosylpyrrole-4-methyl]-*p*-toluenesulfonamide (**5d**) (50 mg, 0.08 mmol) at −20 °C. The reaction mixture was stirred for 0.5 h and poured into a sat. sodium hydrogen carbonate (50 mL). The organic layer was separated and the aqueous layer was extracted with AcOEt. The combined organic layer was dried over $MgSO_4$. The solvent was removed under reduced pressure. To the residue in dichloromethane (2.5 mL) were added tetrabutylammonium hydroxide (0.22 g, 0.80 mmol) in H_2O (2.0 mL) and tetrabutylammonium hydrogensulfate (5.2 mg, 1.6×10^{-2} mmol) at room temperature. The whole was stirred for 2 h and poured into water (50 mL). The organic layer was separated and the aqueous layer was extracted with chloroform. The combined organic layer was washed with water (50 mL) and dried over MgSO4. The solvent was removed under reduced pressure. The residue was purified by preparative TLC on silica gel eluting with AcOEt-*n*-hexane (1:2) gave 1,5-bis(*p*-toluenesulfonyl)-1,4,5,6,7,8-hexahydro-8-phenyl-7-(phenylsulfanyl)pyrrolo-[3,2-*c*]azepin-3-methanol (**11d**) (33 mg, 66%) as white powders.

mp 90–94 °C, IR ν 3535 (OH), 1167 (SO_2), 1093 (SO); ^{1}H NMR (800 MHz, $CDCl_3$) δ 2.22 (3H, s, Me), 2.38 (3H, s, Me), 3.16 (1H, dd, *J* = 2.5 and 13.6 Hz, CH), 3.76 (1H, ddd, *J* = 2.5 and 4.5 and 5.2 Hz, CH), 3.80 (1H, ddd, *J* = 1.6 and 5.2 and 13.6 Hz, CH), 3.89 (1H, d, *J* = 15.6 Hz, CH), 4.53/4.57 (each 1H, d, *J* = 12.5 Hz, CH × 2), 4.59 (1H, dd, *J* = 1.6 and 15.6 Hz, CH), 5.37 (1H, d, *J* = 4.5 Hz, CH), 6.74 (2H, dm, *J* = ca. 7.4 Hz, ArH), 6.86 (2H, dm, *J* = ca. 8.4 Hz, ArH), 7.07 (2H, tm, *J* = ca. 7.4 Hz, ArH), 7.10 (1H, tm-like, *J* = ca. 7.4

Hz, ArH), 7.24 (2H, m, ArH), 7.30 (1H, tm, *J* = ca. 7.4 Hz, ArH), 7.35 (2H, dm, *J* = ca. 7.4 Hz, ArH), 7.38 (1H, br s-like, ArH), 7.46 (2H, dm, *J* = ca. 7.4 Hz, ArH), 7.63 (2H, dm, *J* = ca. 8.4 Hz, ArH); ^{13}C NMR (200 MHz, $CDCl_3$) δ 21.4 (q), 21.5 (q), 44.3 (t), 46.6 (d), 49.9 (t), 54.6 (d), 56.8 (t), 120.9 (d), 124.0 (s), 124.1 (s), 126.5 (d), 126.9 (d × 2), 127.37 (d × 2), 127.38 (d), 128.0 (d × 2), 128.6 (d × 2), 129.1 (d × 2), 129.4 (d × 2), 129.7 (d × 2), 130.8 (s), 132.2 (d × 2), 135.2 (s), 135.35 (s), 135.42 (s), 138.9 (s), 143.5 (s), 144.5 (s); MS (EI) m/z 658 (M^+), 549 (M^+-SPh), 503 (M^+-Ts). Anal. Calcd for $C_{35}H_{34}N_2O_5S_3$+3/2H_2O: C, 61.29; H, 5.44; N, 4.08. Found: C, 61.05; H, 5.26; N, 4.04.

Pummerer reaction of pyrrole **5e** with TFAA and successive treatment with TBAH.

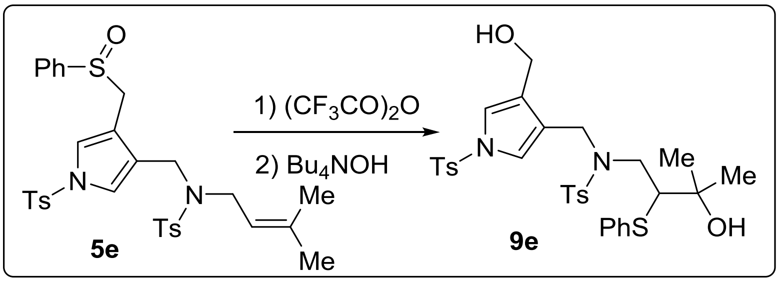

Trifluoroacetic anhydride (60 mg, 0.29 mmol) was added dropwise to a dichloromethane (2.0 mL) solution of *N*-(3-methyl-2-butenyl)-*N*-[(3-(phenylsulfinylmethyl)-1-tosylpyrrole-4-yl)methyl]-*p*-toluenesulfonamide (**5e**) (35 mg, 0.06 mmol) at −20 °C. The reaction mixture was stirred for 1 h and poured into a sat. sodium hydrogen carbonate (50 mL). The organic layer was separated and the aqueous layer was extracted with chloroform. The combined organic layer was dried over $MgSO_4$. The solvent was removed under reduced pressure to give the residue. To the residue in dichloromethane (2 mL) were added tetrabutylammonium hydroxide (0.15 g, 0.57 mmol) in H_2O (1.5 mL) and tetrabutylammonium hydrogensulfate (3.9 mg, 1.1 x 10^{-2} mmol) at room temperature. The mixture was stirred for 0.5 h and poured into water (50 mL). The organic layer was separated and the aqueous layer was extracted with $CHCl_3$. The combined organic layer was dried over $MgSO_4$. The solvent was removed under reduced pressure. The residue was purified by preparative TLC on silica gel eluting with AcOEt-*n*-hexane (1:2) to give *N*-(3-hydroxy-3-methyl-2-(phenylthio)butyl)-*N*-((4-(hydroxymethyl)-1-tosyl-1*H*-pyrrol-3-yl)-methyl)-4-methylbenzenesulfonamide (**9e**) (33 mg, 64%) as white powders.

mp 53–55 °C, IR ν 3421 (OH), 1371, 1337 (SO_2), 1067 (SO); ^{1}H NMR (600 MHz, $CDCl_3$) δ 1.14 (3H, s, Me), 1.15 (3H, s, Me), 2.39 (3H, s, Me), 2.43 (3H, s, Me), 2.61 (1H, brs, OH),

3.00 (1H, dd, J = 9.6 and 15.1 Hz, CH), 3.42 (1H, dd, J = 4.1 and 9.6 Hz, CH), 3.61 (1H, dd, J = 4.1 and 15.1 Hz, CH), 3.98 (1H, d, J = 15.1 Hz, CH), 4.37 (1H, d, J = 15.1 Hz, CH), 4.38 (2H, s, CH_2), 6.80 (1H, d, J = 2.1 Hz, ArH), 7.03 (1H, d, J = 2.1 Hz, ArH), 7.17-7.20 (1H, m, ArH), 7.21-7.23 (4H, m, ArH), 7.26 (2H, d, J = 8.2 Hz, ArH), 7.27 (2H, d, J = 9.0 Hz, ArH), 7.61 (2H, d, J = 8.2 Hz, ArH), 7.70 (2H, d, J = 8.3 Hz, ArH); ^{13}C NMR (150 MHz, $CDCl_3$) δ 21.50 (q), 21.53 (q), 25.9 (q), 27.5 (q), 45.4 (t), 49.3 (t), 56.0 (t), 60.6 (d), 73.0 (s), 119.7 (d), 120.8 (d), 121.6 (s), 126.6 (d), 126.9 (d × 2), 127.4 (d × 2), 128.4 (s), 129.0 (d × 2), 129.8 (d × 2), 130.0 (d × 2), 130.1 (d × 2), 135.0 (s), 135.8 (s), 136.1 (s), 143.9 (s), 145.2 (s); MS (ESI-TOF) m/z 651 $[M + Na]^+$; HRMS (ESI-TOF) m/z: $[M+Na]^+$ Calcd for $C_{31}H_{36}N_2O_6S_3Na$ 651.1633; found 651.1631.

Pummerer reaction of pyrrole **5f** with TFAA and successive treatment with TBAH.

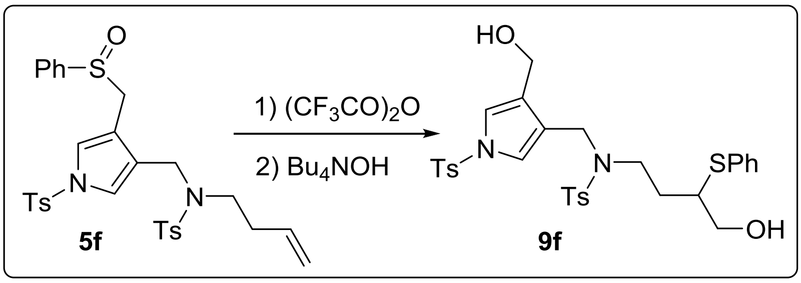

Trifluoroacetic anhydride (88 mg, 0.42 mmol) was added dropwise to a dichloromethane (3.5 mL) solution of *N*-(3-butenyl)-*N*-[3-(phenylsulfinylmethyl)-1-tosylpyrrole-4-methyl]-*p*-toluenesulfonamide (**5f**) (50 mg, 0.08 mmol) at -20 ^{o}C. The reaction mixture was stirred for 0.5 h and poured into a sat. sodium hydrogen carbonate (50 mL). The organic layer was separated and the aqueous layer was extracted with chloroform. The combined organic layer was dried over $MgSO_4$. The solvent was removed under reduced pressure to give the residue. To the residue in dichloromethane (2.5 mL) were added tetrabutylammonium hydroxide (0.22 g, 0.84 mmol) in H_2O (2.0 mL) and tetrabutylammonium hydrogensulfate (5.7 mg, 1.7×10^{-2} mmol) at room temperature. The mixture was stirred for 2 h and poured into water (50 mL). The organic layer was separated and the aqueous layer was extracted with chloroform. The combined organic layer was dried over $MgSO_4$. The solvent was removed under reduced pressure. The residue was purified by preparative TLC on silica gel eluting with AcOEt-*n*-hexane (2:3) to give *N*-(4-hydroxy-3-phenylthiobutyl)-*N*-((4-(hydroxymethyl)-1-tosyl-1*H*-pyrrol-3-yl)methyl)-4-methylbenzenesulfonamide (**9f**) (12 mg, 23 %) as white powders.

mp 42–45 °C, IR ν 3510, 2923, 1707, 1597, 1370, 1304, 1221, 1172, 1092, 1066, 815, 749, 674, 588, 540; ^{1}H NMR (600 MHz, $CDCl_3$) δ 1.24-1.30 (1H, m, CH), 1.45-1.50 (1H, m, CH), 2.37 (3H, s, Me), 2.43 (3H, s, Me), 2.66 (1H, dd, *J* = 8.3 and 13.8 Hz, CH), 2.80 (1H, dd, *J* = 4.2 and 13.8 Hz, CH), 3.01-3.05 (1H, m, CH), 3.27-3.32 (1H, m, CH), 3.53 (1H, brs, CH), 3.95 (1H, d, *J* = 14.5 Hz, CH), 4.23 (1H, d, *J* = 13.8 Hz, CH), 4.42 (1H, d, *J* = 13.1 Hz, CH), 4.47 (1H, d, *J* =13.1 Hz, CH), 6.95 (1H, brs, ArH), 7.03 (1H, d, *J* =1.4 Hz, ArH), 7.22-7.23 (1H, m, ArH), 7.26 (2H, d, *J* =7.6 Hz, ArH), 7.30 (6H, brs, ArH), 7.66 (2H, d, *J* =7.6 Hz, ArH), 7.71 (2H, d, *J* =8.3 Hz, ArH); ^{13}C NMR (150 MHz, $CDCl_3$) δ 21.5 (q), 21.6 (q), 35.2 (t), 41.3 (t), 45.0 (t), 45.7 (t), 56.2 (t), 66.6 (d), 119.7 (d), 120.7 (d), 122.3 (s), 126.5 (d), 126.9 (d × 2), 127.1 (d × 2), 128.1 (s), 129.1 (d × 2), 129.6 (d × 2), 129.9 (d × 2), 130.0 (d × 2), 135.2 (s × 2), 135.7 (s), 143.8 (s), 145.3 (s); MS (ESI-TOF) m/z 637 [M + Na]$^+$; HRMS (ESI-TOF) m/z: [M+Na]$^+$ Calcd for $C_{30}H_{34}N_2O_6S_3Na$ 637.14767; found 637.14502.

Oxidation-Pummerer reaction of **6a** and successive treatment with TBAH.

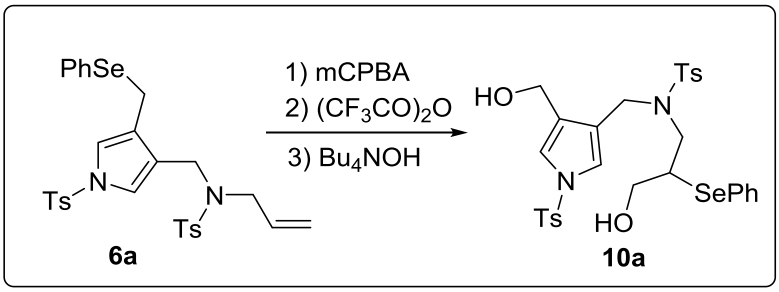

To a 1,2-dichloroethane (2.0 mL) solution of *N*-[3-(phenylselanylmethyl)-1-tosylpyrrole-4-methyl]-*N*-allyl-*p*-toluenesulfonamide (**6a**) (50 mg, 0.08 mmol) was added *m*-chloroperbenzoic acid (14 mg, 0.08 mmol) over 20 min to at 0 °C. The reaction mixture was stirred for 5 min. Then trifluoroacetic anhydride (85.6 mg, 0.41 mmol) was added dropwise to the reaction mixture. The whole was stirred for 15 min and poured into a sat. sodium hydrogen carbonate (50 mL). The mixture was vigorously stirred for 15 min. The organic layer was separated and the aqueous layer was extracted with chloroform. The combined organic layer was dried over $MgSO_4$. The solvent was removed under reduced pressure. To the residue in dichloromethane (2.5 mL) was added tetrabutylammonium hydroxide (0.21 g, 0.81 mmol) in H_2O (2.0 mL) and tetrabutylammonium hydrogensulfate (5.5 mg, 0.02 mmol). The mixture was stirred for 2 h and poured into water (50 mL). The organic layer was separated and the aqueous

layer was extracted with chloroform. The combined organic layer was washed with water (50 mL) and dired over $MgSO_4$. The solvent was removed under reduced pressure. The residue was purified by the preparative TLC on silica gel eluting with AcOEt-*n*-hexane to give *N*-(3-hydroxy-2-(phenylselanyl)propyl)-*N*-((4-(hydroxymethyl)-1-tosyl-1*H*-pyrrol-3-yl)methyl)-4-methylbenzenesulfonamide (**10a**) (48 mg, 91%) as white powders.

mp 37–41 ^{o}C, IR ν 3435, 2924, 2871, 1707, 1597, 1521, 1438, 1372, 1335, 1305, 1172, 1092, 1066, 1018, 915, 814, 745, 673, 588, 550; ^{1}H NMR (800 MHz, $CDCl_3$) 2.39 (3H, s, Me), 2.44 (3H, s, Me), 2.61 (1H, br s, OH), 2.66 (1H, br s, OH), 3.03(1H, m, CH), 3.10 (1H, dd, *J* = 4.9 and 13.2 Hz, CH), 3.56-3.59 (1H, m, CH), 3.58 (1H, dd, *J* = 9.4 and 13.2 Hz, CH), 3.77 (1H, br d-like, *J* = ca. 12 H Hz, CH), 3.82 (1H, d, *J* = 14.4 Hz, CH), 4.27 (1H, d, *J* = 14.4 Hz, CH), 4.46 (1H, br dd-like, *J* = 2.5 and 13 Hz, CH), 4.52 (1H, br dd-like, *J* = 2.5 and 13 Hz, CH), 6.85 (1H, d, *J* = 2.3 Hz, ArH), 7.07 (1H, d, *J* = 2.3 Hz, ArH), 7.25-7.32 (7H, m, ArH), 7.34-7.36 (2H, m, ArH), 7.58 (2H, d-like, *J* = 8.2 Hz, ArH), 7.70 (2H, d-like, *J* = 8.2 Hz, ArH); ^{13}C NMR (200 MHz, $CDCl_3$) δ 21.5 (q), 21.6 (q), 46.1 (t), 46.6 (d), 51.2 (t), 56.2 (t), 61.8 (t), 119.9 (d), 120.8 (d), 122.2 (s), 126.9 (d × 2), 127.3 (d × 2), 127.9 (s), 127.9 (d), 128.7 (s), 129.3 (d × 2), 130.0 (d × 2), 130.1 (d × 2), 134.3 (s), 134.4 (d × 2), 135.6 (s), 144.2 (s), 144.4 (s); MS (EI) m/z 648 (short M^+), HRMS (ESI-TOF) m/z: $[M+Na]^+$ Calcd for $C_{29}H_{32}N_2NaO_6S_2Se$ 671.0759; found 671.0740. Anal. Calcd for $C_{29}H_{32}N_2O_6S_2Se+H_2O$: C, 52.33; H, 5.19; N, 4.21. Found: C, 52.39; H, 5.00; N, 4.19.

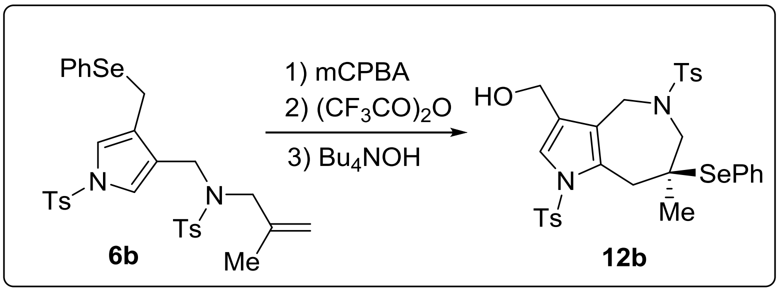

Oxidation-Pummerer reaction of **6b** and successive treatment with TBAH.

m-Chloroperbenzoic acid (14 mg, 0.08 mmol) was added over 20 min to a 1,2-dichloroethane (2.0 mL) solution of *N*-[3-(phenylselanylmethyl)-1-tosylpyrrole-4-methyl]-*N*-allyl-*p*-toluenesulfonamide (**6b**) (50 mg, 0.08 mmol) at 0 ^{o}C. The reaction mixture was stirred for 15 min. To the mixture was added dropwise trifluoroacetic anhydride (85.6 mg, 0.41 mmol). The whole

was stirred for further 25 min and poured into a sat. $NaHCO_3$ (50 mL). Then the mixture was vigorously stirred for 15 min. The organic layer was separated and the aqueous layer was extracted with chloroform. The combined organic layer was dried over $MgSO_4$. The solvent was removed under reduced pressure. To the residue in dichloromethane (2.5 mL) was added tetrabutylammonium hydroxide (0.22 g, 0.81 mmol) in H_2O (2.0 mL) and tetrabutylammonium hydrogensulfate (5.7 mg, 0.02 mmol). The whole was stirred for 1 h and then poured into a saturated sodium hydrogen carbonate (50 mL). The whole was vigorously stirred for 15 min. The organic layer was separated and the aqueous layer was extracted with chloroform. The combined organic layer was dried over MgSO4. The solvent was removed under reduced pressure. The residue was purified by preparative TLC on silica gel eluting with AcOEt-n-hexane (1:2) to give 1,5-bis(*p*-toluenesulfonyl)-1,4,5,6,7,8-hexahydro-7-methyl-7-(phenylselanyl)pyrrolo[3,2-c]azepin-3-methanol (**12b**) (28 mg, 55%) as white powders.

mp 69–73 ^{o}C, IR ν 3444(OH), 1360, 1169 (SO_2), 1091 (SO); ^{1}H NMR (600 MHz, $CDCl_3$) δ 1.30 (3H, s, Me), 2.37 (3H, s, Me), 2.41 (3H, s, Me), 3.39 (1H, d, J = 12.4 Hz, CH), 3.40 (1H, d, J = 11.7 Hz, CH), 3.71 (1H, d, J = 11.7 Hz, CH), 3.92 (1H, d, J = 14.4 Hz, CH), 4.02 (1H, d, J = 14.4 Hz, CH), 4.45 (2H, s, CH_2), 7.20 (2H, d, J = 8.2 Hz, ArH), 7.22 (2H, d, J = 10.3 Hz, ArH), 7.25 (2H, d, J = 15.0 Hz, ArH), 7.29 (2H, d, J = 8.3 Hz, ArH), 7.45 (2H, d, J = 8.2 Hz, ArH), 7.50 (2H, dd, J = 2.1 and 7.6 Hz, ArH), 7.65 (2H, d, J = 8.2 Hz, ArH); ^{13}C NMR (150 MHz, $CDCl_3$) δ 21.5 (q), 21.6 (q), 23.0 (q), 36.3 (t), 40.1 (s), 43.8 (t), 55.9 (t), 56.8 (t), 121.2 (s), 123.1 (s), 123.5 (d), 126.3 (d × 2), 126.6 (d), 127.7 (d × 2), 128.9 (d × 2), 129.8 (d × 2), 129.9 (d × 2), 131.2 (s), 132.5 (d × 2), 133.0 (s), 134.7 (s), 136.9 (s), 143.7 (s), 144.9 (s); MS (EI) m/z 644 (M^+), 489 (M^+-Ts). Anal. Calcd for $C_{30}H_{32}N_2O_5S_2Se$+1/2H_2O: C, 54.46; H, 5.18; N, 4.23. Found: C, 54.71; H, 5.20; N, 3.93.

Oxidation-Pummerer reaction of **6c** and successive treatment with TBAH.

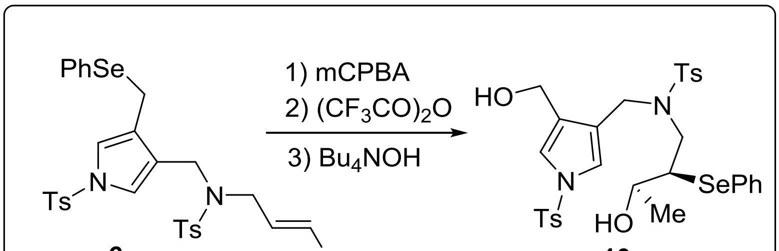

6c **10c**

m-Chloroperbenzoic acid (14 mg, 0.08 mmol) was added over 20 min to a 1,2-dichloroethane (2.0 mL) solution of *N*-[3-(phenylselanylmethyl)-1-t

osylpyrrole-4-methyl]-*N*-but-2-enyl-*p*-toluenesulfonamide (**6c**) (50 mg, 0.08 mmol) at 0 ^{o}C. After stirring for 10 min, trifluoroacetic anhydride (83.7 mg, 0.39 mmol) was added dropwise to the reaction mixture. The whole was stirred for further 15 min and poured into a sat. sodium hydrogen carbonate (50 mL). Then the mixture was vigorously stirred for 15 min. The organic layer was separated and the aqueous layer was extracted with chloroform. The combined organic layer was dried over $MgSO_4$. The solvent was removed under reduced pressure. To the residue in dichloromethane (2.5 mL) was added tetrabutylammonium hydroxide (0.21 g, 0.81 mmol) in H_2O (2 mL) and tetrabutylammonium hydrogensulfate (5.4 mg, 0.02 mmol). The whole was stirred for 1 h and then poured into a saturated sodium hydrogen carbonate (50 mL). The mixture was vigorously stirred for 15 min. The organic layer was separarted and the aqueous layer was extracted with chloroform. The combined organic layer was dried over $MgSO_4$. The solvent was removed under reduced pressure. The residue was purified by the preparative TLC on silica gel eluting with AcOEt-*n*-hexane (1:2) to give *N*-(3-hydroxy-2-(phenylselanyl)butyl)-*N*-((4-(hydroxymethyl)-1-tosyl-1*H*-pyrrol-3-yl)methyl)-4-methylbenzenesulfonamide (**10c**) (40 mg, 76%) as white powders.

mp 44–47 ^{o}C, IR ν 3434 (OH), 1370, 1304, 1172, 1092 (SO_2), 1067 (SO); ^{1}H NMR (800 MHz, $CDCl_3$) δ 0.97 (3H, d, *J* = 6.4 Hz, Me), 2.39 (3H, s, Me), 2.44 (3H, s, Me), 2.44 (1H, br s, OH), 2.83 (1H, brs, OH), 3.15 (1H, dd, *J* = 5.5 and 13.2 Hz, CH), 3.36 (1H, m, CH), 3.38 (1H, dd, *J* = 8.2 and 13.2 Hz, CH), 3.77 (1H, m, CH), 4.02 (1H, d, *J* = 14.4 Hz, CH), 4.06 (1H, dd, *J* = 14.4 Hz, CH), 4.54 (1H, d, *J* = 13.2 Hz, CH), 4.60 (1H, d, *J* = 13.2 Hz, CH), 6.85 (1H, d, *J* = 2.3 Hz, ArH), 7.11 (1H, d, *J* = 2.3 Hz, ArH), 7.25–7.29 (6H, m, ArH), 7.30–7.33 (1H, m, ArH), 7.38–7.40 (2H, m, ArH), 7.55 (2H, d-like, *J* = 8.2 Hz, ArH), 7.71 (2H, d-like, *J* = 8.2 Hz, ArH); ^{13}C NMR (200 MHz, $CDCl_3$) δ 19.5 (q), 21.5 (q), 21.6 (q), 46.2 (t), 50.6 (t), 53.7 (d), 56.3 (t), 67.2 (d), 120.0 (d), 120.6 (d), 121.9 (s), 127.0 (d × 2), 127.96 (s) 127.98 (d × 2), 128.3 (s), 129.3 (d × 2), 129.9 (d × 2), 130.1 (d × 2), 134.0 (s), 134.6 (d × 2), 135.7 (s), 144.1 (s), 145.3 (s); MS (EI) m/z 662 (M^+), 507 (M^+–Ts). HRMS (ESI-TOF) m/z: $[M+Na]^+$ Calcd for $C_{30}H_{34}N_2NaO_6S_2Se$ 685.0921; found 685.0908.

Oxidation-Pummerer reaction of **6d** and successive treatment with TBAH.

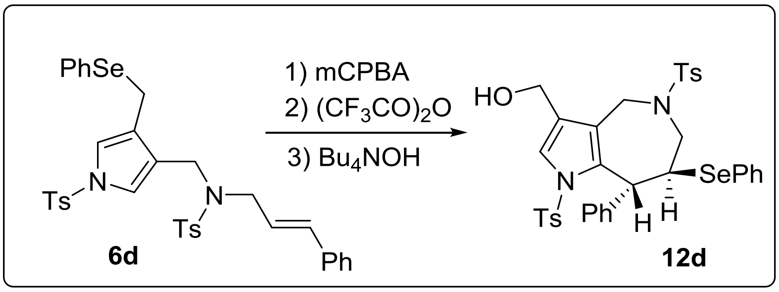

m-Chloroperbenzoic acid (16.3 mg, 0.09 mmol) was added over 15 min to a 1,2-dichloroethane (2.0 mL) solution of *N*-[3-(phenylselanylmethyl)-1-tosylpyrrole-4-methyl]-*N*-cinnamyl-*p*-toluenesulfonamide (**6d**) (50 mg, 0.07 mmol) at 0 °C. After stirring for 10 min, trifluoroacetic anhydride (76.1 mg, 0.36 mmol) was added dropwise to the reaction mixture. The whole was stirred for further 15 min and poured into a sat. sodium hydrogen carbonate (50 mL). Then the mixture was vigorously stirred for 15 min. The organic layer was separated and the aqueous layer was extracted with chloroform. The combined organic layer was dried over $MgSO_4$. The solvent was removed under reduced pressure. To the residue in dichloromethane (2.5 mL) was added tetrabutylammonium hydroxide (0.19 g, 0.72 mmol) in H_2O (2.0 mL) and tetrabutylammonium hydrogensulfate (4.9 mg, 0.01 mmol). The whole was stirred for 1 h and poured into a saturated sodium hydrogen carbonate (50 mL) solution. The mixture was vigorously stirred for 15 min. The organic layer was separated and the aqueous layer was extracted with chloroform. The combined organic layer was washed with a sodium hydrogenecarbonate (50 mL) and dried over $MgSO_4$. The solvent was removed under reduced pressure. The residue was purified by preparative TLC on silica gel eluting with AcOEt-*n*-hexane 1:2 to give 1,5-bis(p-toluenesulfonyl)-1,4,5,6,7,8-hexahydro-8-phenyl-7-(phenylselanyl)pyrrolo[3,2-c]azepin-3-methanol (**12d**) (40 mg, 76%) as white powders.

mp 88–91 °C, IR ν 3445 (OH), 1362, 1270, 1167 (SO_2), 1101 (SO); ^{1}H NMR (600 MHz, $CDCl_3$) δ 2.21 (3H, s, Me), 2.38 (3H, s, Me), 3.12 (1H, dd, *J* = 2.0 and 13.7 Hz, CH), 3.74 (1H, d, *J* = 15.1 Hz, CH), 3.76-3.78 (1H, m, CH), 3.98-4.01 (1H, m, CH), 4.52 (1H, d, *J* = 13.1 Hz, CH), 4.57 (1H, d, *J* = 12.3 Hz, CH_2), 4.67 (1H, dd, *J* = 2.0 and 15.1 Hz, CH), 5.44 (1H, d, *J* = 4.1 Hz, CH), 6.67 (2H, d, *J* = 7.6 Hz, ArH), 6.84 (2H, d, *J* = 8.2 Hz, ArH), 7.02-7.09 (3H, m, ArH), 7.22-7.27 (4H, m, ArH), 7.33-7.35 (3H, m, ArH), 7.38 (1H, s, ArH),

7.63-7.65 (4H, m, ArH); ^{13}C NMR (150 MHz, $CDCl_3$) δ 21.4(q), 21.5 (q), 44.6 (t), 47.4 (d), 50.2 (d), 50.8 (t), 56.7 (t), 120.7 (d), 124.0 (s), 124.1 (s), 126.4 (d), 126.9 (d × 2), 127.4 (d × 2), 127.7 (d), 127.9 (d × 2), 128.6 (d × 2), 129.2 (d × 2), 129.4 (d × 2), 129.7 (d × 2), 130.4 (s), 131.4 (s), 134.5 (d × 2), 135.06 (s), 135.15 (s), 139.2 (s), 143.6 (s), 144.5 (s); MS (EI) m/z 706 (M^+), 549 (M^+-SePh). MS (ESI-TOF) m/z 729 $[M + Na]^+$; HRMS (ESI-TOF) m/z: $[M+Na]^+$ Calcd for $C_{35}H_{34}N_2O_5S_2SeNa$ 729.0972; found 729.0946. Single X-ray analysisi of **12d** was described in the CIF format.

Oxidation-Pummerer reaction of **6e** and successive treatment with TBAH.

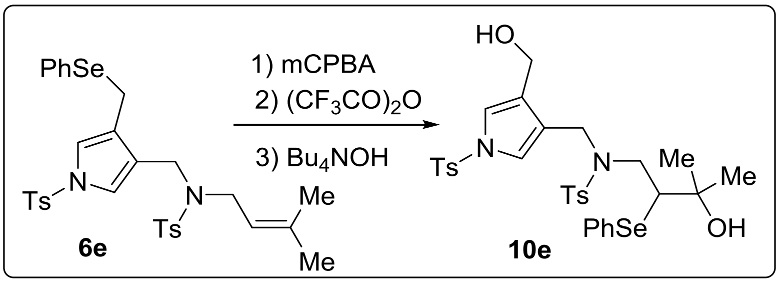

m-Chloroperbenzoic acid (60 mg, 0.35 mmol) was added over 1 h to a 1,2-dichloroethane (8.0 mL) solution of *N*-(3-methyl-2-butenyl)-*N*-[(3-(phenylselanylmethyl)-1-tosylpyrrole-4-yl)methyl]-*p*-toluene- sulfonamide (**6e**) (186 mg, 0.29 mmol) at 0 °C. After stirring for 20 min, trifluoroacetic anhydride (304.4 mg, 1.45 mmol) was added dropwise to the reaction mixture. The whole was stirred for further 45 min and poured into a sat. sodium hydrogen carbonate (50 mL). Then the mixture was vigorously stirred for 15 min. The organic layer was separated and the aqueous layer was extracted with chloroform. The combined organic layer was dried over $MgSO_4$. The solvent was removed under reduced pressure. To the residue in dichloroethane (10.0 mL) was added tetrabutylammonium hydroxide (0.75 g, 2.90 mmol) in H_2O (7.0 mL) and tetrabutylammonium hydrogensulfate (19.7 mg, 0.06 mmol). The whole was stirred for 1 h and then poured into a sodium hydrogen carbonate (50 mL) solution. The mixture was vigorously stirred for 15 min. The organic layer was separated and the aqueous layer was extracted with chloroform. The combined organic layer was dried over $MgSO_4$. The solvent was removed under reduced pressure. The residue was purified by preparative TLC on silica gel eluting with AcOEt-*n*-hexane (1:2) to give *N*-(3-hydroxy-3-methyl-2-(phenylselanyl)butyl)-*N*-((4-(hydroxymethyl)-1-tosyl-1*H*-pyrrol-3-yl)methyl)-4-methylbenzenesulfonamide (**10e**) (42 mg, 21%) as white powder.

mp 54–56 °C, IR ν 3421 (OH), 1371, 1337, 1172 (SO_2), 1067 (SO); ^{1}HNMR (600 MHz, $CDCl_3$) δ 1.14 (3H, s, Me), 1.18 (3H, s, Me), 2.40 (3H, s, Me), 2.43 (3H, s, Me), 2.61 (1H, brs, OH), 2.74 (1H, brs, OH), 3.11 (1H, dd, *J* = 9.0 and 15.1 Hz, CH), 3.44 (1H, dd, *J* = 5.5 and 8.9 Hz, CH), 3.65 (1H, *J* = 5.5 and 15.1 Hz, CH), 3.94 (1H, d, *J* = 15.1 Hz, CH), 4.33 (1H, d, *J* = 14.4 Hz, CH), 4.42 (2H, s, CH × 2), 6.77 (1H, s, ArH), 7.04 (1H, d, *J* = 2.1 Hz, ArH), 7.22-7.28 (7H, m, ArH), 7.39 (2H, d, *J* = 6.1 Hz, ArH), 7.59 (2H, d, *J* = 8.3 Hz, ArH), 7.70 (2H, d, *J* = 7.6 Hz, ArH); ^{13}C NMR (150 MHz, $CDCl_3$) δ 21.5 (q), 21.6 (q), 26.2 (q), 28.3 (q), 45.4 (t), 50.2 (t), 56.1 (t), 58.7 (d), 72.9 (s), 119.8 (d), 120.7 (d), 121.7 (s), 126.9 (d × 2), 127.35 (d), 127.42 (d × 2), 128.4 (s), 129.2 (d × 2), 129.8 (d × 2), 130.0 (d × 2), 130.4 (s), 133.1 (d × 2), 134.7 (s), 135.8 (s), 143.9 (s), 145.2 (s); MS (ESI-TOF) m/z 699 $[M + Na]^+$; HRMS (ESI-TOF) m/z: $[M+Na]^+$ Calcd for $C_{31}H_{36}N_2O_6S_2SeNa$ 699.1078; found 699.1074. Anal. Calcd for $C_{31}H_{36}N_2O_6S_2Se{+}H_2O$: C, 53.67; H, 5.52; N, 4.04. Found: C, 53.75; H, 5.35; N, 4.11.

Oxidation-Pummerer reaction of **6f** and successive treatment with TBAH.

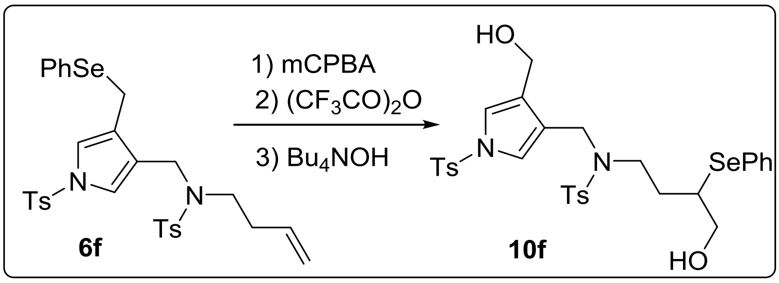

m-Chloroperbenzoic acid (45 mg, 0.26 mmol) was added over 0.5 h to a 1,2-dichloroethane (8.0 mL) solution of *N*-[(3-(phenylselanylmethyl)-1-tosylpyrrole-4-yl)methyl]-*N*-(3-butenyl)-*p*-toluenesulfonamide (**6f**) (135 mg, 0.22 mmol) at 0 °C. After stirring for 10 min, trifluoroacetic anhydride (225.9 mg, 1.08 mmol) was added dropwise to the reaction mixture. The whole was stirred for further 15 min and poured into a sat. sodium hydrogen carbonate (50 mL). Then the mixture was vigorously stirred for 15 min. The organic layer was separated and the aqueous layer was extracted with chloroform. The combined organic layer was dried over $MgSO_4$. The solvent was removed under reduced pressure. To the residue in dichloromethane (6.5 mL) was added tetrabutylammonium hydroxide (0.56 g, 2.15 mmol) in H_2O (5 mL) and tetrabutylammonium hydrogensulfate (14.6 mg, 0.04 mmol). The whole was stirred for 1 h and poured into a saturated sodium hydrogencarbonate (50 mL) solution. The mixture was vigorously stirred for 15 min. The organic layer was separated and the aqueous

layer was extracted with chloroform. The combined organic layer was dried over $MgSO_4$. The solvent was removed under reduced pressure to give *N*-(4-hydroxy-3-phenylselanylbutyl)-*N*-(4-(hydroxymethyl)-1-tosyl-1*H*-pyrrol-3-yl)methyl)-4-methylbenzenesulfonamide (**10f**) (32 mg, 23%) as white powders.

mp 45–47 °C, IR ν 3502 (OH), 1370, 1333, 1172 (SO_2), 1066 (SO); ^{1}HNMR (600 MHz, $CDCl_3$) δ 1.25-1.31 (1H, m, CH), 1.44-1.50 (1H, m, CH), 2.38 (3H, s, Me), 2.44 (3H, s, Me), 2.66 (1H, brs, OH), 2.67 (1H, dd, *J* = 8.3 and 13.1 Hz, CH), 2.76 (1H, brs, OH), 2.80 (1H, dd, *J* = 4.9 and 13.1 Hz, CH), 3.00-3.05 (1H, m, CH), 3.27-3.32 (1H, m, CH), 3.55 (1H, brs, CH), 3.95 (1H, d, *J* = 13.8 Hz, CH), 4.23 (1H, d, *J* = 14.5 Hz, CH), 4.43 (1H, d, *J* = 13.0 Hz, CH), 4.49 (1H, d, *J* = 13.7 Hz, CH), 6.94 (1H, d, *J* = 2.1 Hz, ArH), 7.04 (1H, d, *J* = 2.0 Hz, ArH), 7.25-7.29 (5H, m, ArH), 7.30 (2H, d, *J* = 8.2 Hz, ArH), 7.45-7.47 (2H, m, ArH), 7.66 (2H, d, *J* = 8.2 Hz, ArH), 7.71 (2H, d, *J* = 9.0 Hz, ArH); ^{13}C NMR (150 MHz, $CDCl_3$) δ 21.5 (q), 21.6 (q), 35.7 (t), 36.2 (t), 45.0 (t), 45.8 (t), 56.2 (t), 67.1 (d), 119.7 (d), 120.7 (d), 122.3 (s), 126.9 (d × 2), 127.2 (d × 2), 127.3 (d), 128.1 (s), 129.26 (d × 2), 129.32 (s), 129.9 (d × 2), 130.1 (d × 2), 132.8 (d × 2), 135.3 (s), 135.8 (s), 143.9 (s), 145.3 (s); MS (ESI-TOF) m/z 685 $[M + Na]^+$; HRMS (ESI-TOF) m/z: $[M+Na]^+$ Calcd for $C_{30}H_{34}N_2O_6S_2SeNa$ 685.0921; found 685.0912. Anal. Calcd for $C_{30}H_{34}N_2O_6S_2Se{+}1/4H_2O$: C, 54.09; H, 5.22; N, 4.20. Found: C, 54.13; H, 5.35; N, 4.16.

Pummerer reaction of **5a** in the presence of *p*-methoxybenzenethiol.

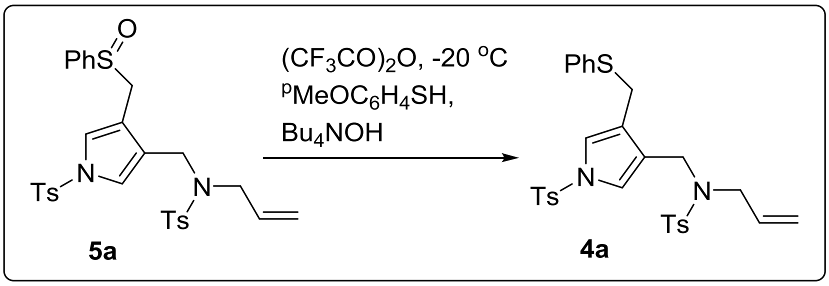

To a dichloroethane (0.50 mL) solution of **5a** (20 mg, 0.03 mmol) and *p*-methoxybenzenethiol (14.1 mg, 0.10 mmol) was added trifluoroacetic anhydride (36 mg, 0.10 mL, 0.17 mmol) at −20 °C. The reaction mixture was stirred for 0.5 h and poured into a saturated sodium hydrogen carbonate (50 mL). The organic layer was separated and the aqueous layer was extracted with AcOEt. The combined organic layer was dried over $MgSO_4$. The solvent was removed under reduced pressure. To the residue in dichloroethane (2.0 mL) was added tetrabutylammonium hydroxide (87 mg, 0.34 mmol) in H_2O (1 mL) and tetrabutylammonium hydrogensulfate

(2.3 mg, 0.01 mmol) at 0 °C. The whole was stirred for 1 h and then poured into a saturated sodium hydrogenecarbonate (50 mL). The mixture was vigorously stirred for 15 min. The organic layer was separated and the aqueous layer was extracted with chloroform. The combined organic layer was dried over $MgSO_4$. The solvent was removed under reduced pressure. The residue was purified by the preparative TLC on silica gel eluting with AcOEt-*n*-hexane (1:2) to give **4a** (13 mg, 77%).

Pummerer reaction of **5a** and successive treatment with TBAH in the presence of *p*-methoxybenzenethiol.

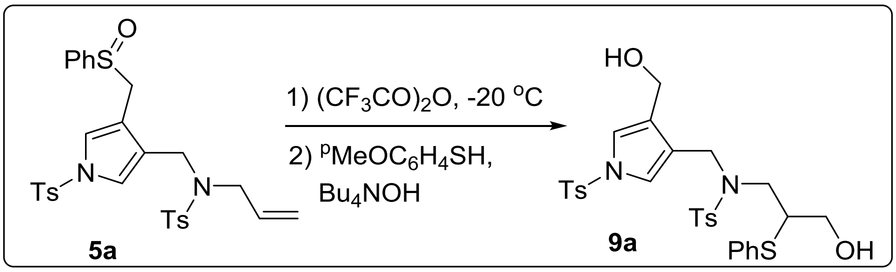

To a THF (0.50 mL) solution of **5a** (20 mg, 0.03 mmol) was added trifluoroacetic anhydride (36 mg, 0.10 mL, 0.17 mmol) at −20 °C. The reaction mixture was stirred for 0.5 h and poured into a saturated sodium hydrogen carbonate (50 mL). The organic layer was separated and the aqueous layer was extracted with AcOEt. The combined organic layer was dried over $MgSO_4$. The solvent was removed under reduced pressure. To the residue in dichloromethane (2.0 mL) was added tetrabutylammonium hydroxide (87 mg, 0.34 mmol) in H_2O (1 mL), *p*-methoxybenzenethiol (14.1 mg, 0.10 mmol) and tetrabutylammonium hydrogensulfate (2.3 mg, 0.01 mmol). The whole was stirred for 1 h and then poured into a saturated sodium carbonate (50 mL) solution. The organic layer was separated and the aqueous layer was extracted with AcOEt. The combined organic layer was dried over $MgSO_4$. The solvent was removed under reduced pressure. The residue was purified by the preparative TLC on silica gel eluting with AcOEt-*n*-hexane (1:2) to give **9a** (13 mg, 77%).

Pummerer reaction of **5a** and successive treatment with TBAH in the presence of *p*-chlorobenzenethiol.

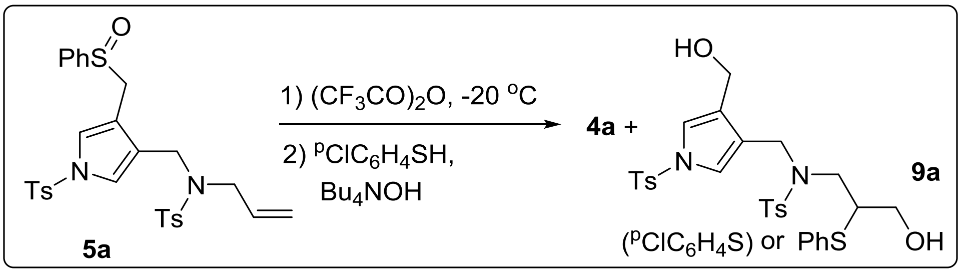

To a dichloroethane (0.50 mL) solution of **5a** (20 mg, 0.03 mmol) was added TFAA (36 mg, 0.10 mL, 0.17 mmol) at −20 °C. The reaction mixture was stirred for 0.5 h and poured into a saturated sodium hydrogen carbonate (50 mL). The organic layer was separated and the aqueous layer was extracted with AcOEt. The combined organic layer was dried over $MgSO_4$. The solvent was removed under reduced pressure. To the residue in dichloromethane (2.0 mL) was added tetrabutylammonium hydroxide (87 mg, 0.34 mmol) in H_2O (1 mL), *p*-chlorobenzenethiol (15.0 mg, 0.10 mmol) and tetrabutylammonium hydrogensulfate (2.3 mg, 0.01 mmol). The whole was stirred for 1 h and then poured into a saturated sodium carbonate (50 mL) solution. The organic layer was separated and the aqueous layer was extracted with chloroform. The combined organic layer was dried over $MgSO_4$. The solvent was removed under reduced pressure. The residue was purified by the preparative TLC on silica gel eluting with AcOEt-*n*-hexane (1:2) to give **4a** (10 mg, 60%) and **9a** (3.0 mg, 17%).

Ytterbium triflate-catalyzed intramolecular cyclization of diol **9a**.

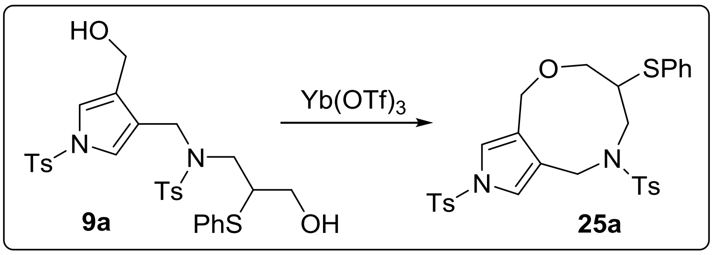

To a dichloroethane (1.5 mL) solution of *N*-(3-hydroxy-2-(phenylthio)propyl)-*N*-((4-(hydroxymethyl)-1-tosyl-1*H*-pyrrol-3-yl)methyl)-4-methylbenzenesulfonamide (**9a**) (30 mg, 0.05 mmol) was added ytterbium(III) trifluoromethanesulfonate hydrate (6.2 mg, 0.01 mmol) at room temperature. The reaction mixture was stirred at reflux for 3.5 h and then poured into a sodium hydrogen carbonate (50 mL). The organic layer was separated and the aqueous layer was extracted with chloroform. The combined organic layer was dried over $MgSO_4$. The solvent was removed under reduced pressure. The residue was purified by column chromatography on silica gel eluting with AcOEt-*n*-hexane (1:3) to

give 7-(phenylthio)-2,9-ditosyl-2,6,7,8,9,10-hexahydro-4H-pyrrolo[3,4-g][1,5]oxazonine (**25a**) (12 mg, 41%) as white powder.

mp 158–160 °C, IR ν 2924, 1374, 1341, 1173 (SO_2), 1067 (SO); ^{1}H NMR (600 MHz, $CDCl_3$) δ 2.41 (6H, s, Me × 2), 3.10 (1H, dd, *J* = 11.0 and 15.1 Hz, CH), 3.54-3.58 (2H, m, CH × 2), 3.67 (1H, dd, *J* = 5.5 and 15.2 Hz, CH), 3.78 (1H, d, *J* = 15.1 Hz, CH), 3.89 (1H, dd, *J* = 3.5 and 10.4 Hz, CH), 4.45 (1H, d, *J* = 13.8 Hz, CH), 4.56 (1H, d, *J* = 15.8 Hz, CH), 4.75 (1H, d, *J* = 13.7 Hz, CH), 6.94 (1H, s, ArH), 7.23 (2H, d, *J* = 8.3 Hz, ArH), 7.25-7.27 (2H, m, ArH), 7.30 (4H, m, ArH), 7.38 (2H, br d, *J* = 7.6 Hz, ArH), 7.55 (2H, d, *J* = 8.3 Hz, ArH), 7.76 (2H, d, *J* = 8.3 Hz, ArH); ^{13}C NMR (150 MHz, $CDCl_3$) δ 21.5 (q), 21.6 (q), 47.5 (d), 48.5 (t), 53.5 (t), 65.0 (t), 66.6 (t), 118.4 (d), 122.9 (d), 123.5 (s), 124.7 (s), 126.9 (d), 127.0 (d × 2), 127.1 (d × 2), 129.1 (d × 2), 129.7 (d × 2), 130.1 (d × 2), 130.7 (d × 2), 134.3 (s), 135.8 (s), 136.0 (s), 143.5 (s), 145.1 (s); MS (ESI-TOF) m/z 605 $[M + Na]^+$; HRMS (ESI-TOF) m/z: $[M+Na]^+$ Calcd for $C_{29}H_{30}N_2O_5S_3Na$ 605.1215; found 605.1187. Anal. Calcd for. Anal. Calcd for $C_{29}H_{30}N_2O_5S_3$+1/3H_2O: C, 59.16; H, 5.25; N, 4.76. Found: C, 58.88; H, 5.02; N, 4.61.

Ytterbium triflate-catalyzed intramolecular cyclization of diol **9c**.

To a 1,2-dichloroethane (1.5 mL) solution of *N*-(3-hydroxy-2-(phenylthio)butyl)-*N*-((4-(hydroxymethyl)-1-tosyl-1*H*-pyrrol-3-yl)methyl)-4-methylbenzene sulfonamide (**9c**) (30 mg, 0.05 mmol) was added ytterbium(III) trifluoromethanesulfonate hydrate (6.1 mg, 0.01 mmol). The reaction mixture was stirred at reflux for 4.5 h and then poured into a sodium hydrogen carbonate (50 mL). The organic layer was separated and the aqueous layer was extracted with chloroform. The combined organic layer was dried over $MgSO_4$. The solvent was removed under reduced pressure. The residue was purified by column chromatography on silica gel eluting with AcOEt-*n*-hexane (1:3) to give 6-methyl-7-(phenylthio)-2,9-ditosyl-2,6,7,8,9,10-hexahydro-4*H*-pyrrolo[3,4-*g*][1,5]oxazonine (**25c**) (10 mg, 34%) as white powder.

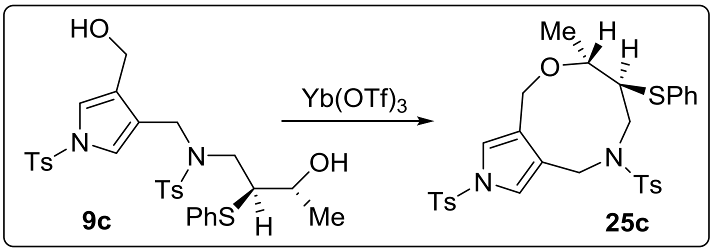

mp 185–187 °C, IR ν 2924, 1370, 1300 (SO_2), 1066 (SO); ^{1}H NMR (600 MHz, $CDCl_3$) δ 1.44 (3H, d, *J* = 6.2 Hz, Me), 2.40 (3H, s, Me), 2.41 (3H, s, Me), 2.97 (1H, dd, *J* = 8.2 and 15.1 Hz, CH), 3.50-3.61 (3H, m, CH × 3), 4.00 (1H, d, *J* = 14.4 Hz, CH), 4.31 (1H, d, *J* = 15.2 Hz, CH), 4.33 (1H, d, *J* = 12.3 Hz, CH), 4.59 (1H, d, *J* = 11.7 Hz, CH), 6.94 (1H, d, *J* = 2.0 Hz, ArH), 6.98 (1H, brs, ArH), 7.18 (2H, d, *J* = 8.2 Hz, ArH), 7.28 (2H, d, *J* = 8.2 Hz, ArH), 7.30-7.38 (5H, m, ArH), 7.51 (2H, brd, *J* = 7.5 Hz, ArH), 7.69 (2H, d, *J* = 8.2 Hz, ArH); ^{13}C NMR (150 MHz, $CDCl_3$) δ 18.4 (q), 21.5 (q), 21.6 (q), 48.6 (t), 53.3 (d), 54.9 (t), 60.1 (t), 75.6 (d), 118.8 (d), 120.2 (d), 124.4 (s), 125.5 (s), 126.7 (d × 2), 127.3 (d × 2), 127.4 (d), 129.0 (d × 2), 129.5 (d × 2), 130.0 (d × 2), 132.7 (d × 2), 133.5 (s), 134.9 (s), 135.8 (s), 143.3 (s), 145.1 (s); MS (ESI-TOF) m/z 619 [M + Na]$^+$; HRMS (ESI-TOF) m/z: [M+Na]$^+$ Calcd for $C_{30}H_{32}N_2O_5S_3Na$ 619.1371; found 619.1393. Anal. Calcd for $C_{30}H_{32}N_2O_5S_3$+1/3H_2O: C, 59.78; H, 5.46; N, 4.65. Found: C, 59.50; H, 5.41; N, 4.60.

Ytterbium triflate-catalyzed intramolecular cyclization of diol **9e**.

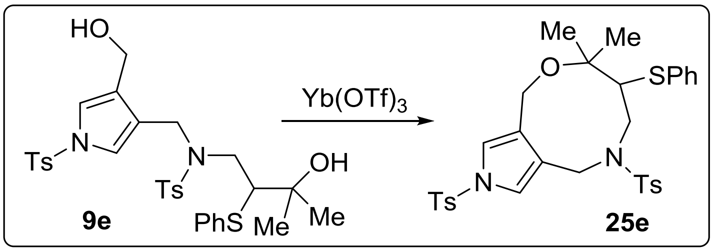

To a 1,2-dichloroethane (1.5 mL) solution of *N*-(3-hydroxy-3-methyl-2-(phenylthio)butyl)-*N*-((4-(hydroxymethyl)-1-tosyl-1*H*-pyrrol-3-yl)-methyl)-4-methylbenzenesulfonamide (**9e**) (30 mg, 0.05 mmol) was added ytterbium(III) trifluoromethanesulfonate hydrate (5.9 mg, 0.01 mmol). The reaction mixture was stirred at reflux for 3.5 h and then poured into a saturated sodium hydrogen carbonate (50 mL). The organic layer was separated and the aqueous layer was extracted with chloroform. The combined organic layer was dried over $MgSO_4$. The residue was purified by column chromatography on silica gel eluting with AcOEt-*n*-hexane (1:8) to give 6,6-dimethyl-7-(phenylthio)-2,9-ditosyl-2,6,7,8,9,10-hexahydro-4*H*-pyrrolo-[3,4-g][1,5]oxazonine (**25e**) (14 mg, 48%) as white powder.

mp 86–89 °C, IR ν 2925, 1364, 1173 (SO_2), 1064 (SO); ^{1}H NMR (600 MHz, $CDCl_3$) δ 1.20 (3H, s, Me), 1.57 (3H, s, Me), 2.37 (3H, s, Me), 2.38 (3H, s, Me), 3.11 (1H, dd, *J* = 10.3 and 15.8 Hz, CH), 3.73 (1H, dd, *J* = 1.4 and 15.8 Hz, CH), 3.90 (1H, d, *J* = 8.9 Hz, CH), 3.98 (1H, dd, *J* = 1.4 and 15.8 Hz, CH), 4.28 (1H, d, *J* = 10.3 Hz, CH), 4.45 (1H, d, *J* =

15.1 Hz, CH), 4.49 (1H, d, J = 11.0 Hz, CH), 6.93 (1H, br s, ArH), 7.00 (1H, br s, ArH), 7.09 (3H, s, ArH), 7.26 (2H, d, J = 8.2 Hz, ArH), 7.38 (1H, brd, J = 7.5 Hz, ArH), 7.42 (2H, t, J = 6.9 Hz, ArH), 7.62 (2H, d, J = 6.9 Hz, ArH), 7.69 (2H, d, J = 8.3 Hz, ArH); ^{13}C NMR (150 MHz, $CDCl_3$) δ 21.5 (q), 21.6 (q), 23.5 (q), 24.8 (q), 51.0 (t), 55.6 (t), 56.2 (d), 57.3 (t), 77.7 (s), 117.9 (d), 118.1 (d), 125.0 (s), 126.2 (s), 126.7 (d × 2), 127.2 (d × 2), 127.9 (d), 129.0 (d × 2), 129.4 (d × 2), 129.9 (d × 2), 133.4 (d × 2), 134.1 (s), 135.0 (s), 136.2 (s), 143.2 (s), 144.8 (s); MS (ESI-TOF) m/z 633 [M + Na]$^+$. HRMS (ESI-TOF) m/z: [M+Na]$^+$ Calcd for $C_{31}H_{34}N_2O_5S_3Na$ 633.1528; found 633.1506. Anal. Calcd for $C_{31}H_{34}N_2O_5S_3$+1/2H_2O: C, 60.07; H, 5.69; N, 4.52. Found: C, 59.91; H, 5.71; N, 4.44.

Synthesis of 1,5-Bis(*p*-toluenesulfonyl)-1,4,5,6,7,8-hexahydro-7-(phenylsulfanyl)pyrrolo[3,2-c]azepin-3-methanol (**25g**).

Reaction of 1,6-diyne **1** with *N*-allyl *p*-bromobenzenesulfonamide (**3g**).

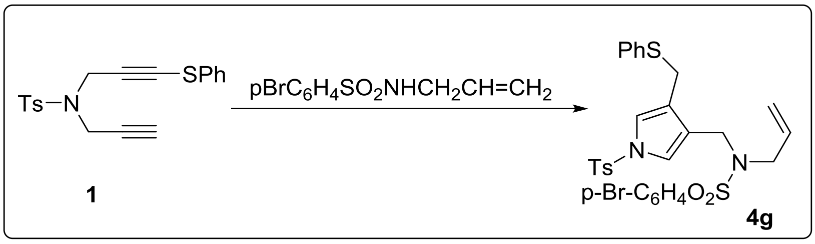

To a DMSO (2.0 mL) solution of *N*-(phenylsulfanylprop-2-ynyl)-*N*-prop-2-ynyl-*p*-toluenesulfonamide (**1**) (200 mg, 0.56 mmol) were added *N*-allyl-*p*-bromobenzenesulfonamide (**3g**) (311 mg, 1.13 mmol), bis(hexafluoroacetylacetonato)nickel(II) hydrate (27 mg, 0.06 mmol), bis(triphenylphosphine)palladium(II) dichloride (40 mg, 0.06 mmol), and 1,8-diazabicyclo[5.4.0]undec-7-ene (86 mg, 0.56 mmol). The reaction mixture was stirred at room temperature for overnight and then poured into water (50 mL). The organic layer was separated and the aqueous layer was extracted with AcOEt. The combined organic layer was dried over $MgSO_4$. The solvent was removed under reduced pressure. The residue was purified by column chromatography on silica gel eluting with $CHCl_3$-*n*-hexane (4:1) to give *N*-[(3-(phenylsulfanylmethyl)-1-tosylpyrrole-4-yl)methyl]-*N*-allyl-4-bromobenzenesulfon-amide (**4g**) (319 mg, 90%) as white powder.

mp 79–81 °C, IR ν 3291, 2926, 1712, 1597, 1439, 1348, 1160, 1092, 1067, 1025, 887, 815, 731, 673, 586, 550; ^{1}H NMR (600 MHz, $CDCl_3$) δ 2.42 (3H, s, Me), 3.70 (2H, d, *J* = 6.2 Hz, CH_2), 3.87 (2H, s, CH_2), 4.18 (2H, s, CH_2), 4.92 (1H, d, *J* = 17.8 Hz, olefinic H), 5.00 (1H, d, *J* = 10.3 Hz, olefinic H), 5.36-5.43 (1H, m, olefinic H), 6.87 (1H, d, *J* = 2.0 Hz, ArH), 6.89 (1H, d, *J* = 2.0 Hz, ArH), 7.18-7.22 (5H, m, ArH), 7.26 (2H, d, *J* = 8.3 Hz, ArH), 7.59 (2H, d, *J* = 8.3 Hz, ArH), 7.60 (2H, d, *J* = 8.2 Hz, ArH), 7.65 (2H, d, *J* = 8.9 Hz, ArH); ^{13}C NMR (150 MHz, $CDCl_3$) δ 21.6 (q), 29.2 (t), 41.9 (t), 49.6 (t), 119.5 (t), 120.2 (d), 120.7 (d), 121.9 (s), 123.8 (s), 126.6 (d), 126.7 (d × 2), 127.6 (s), 128.6 (d × 2), 128.7 (d × 2), 130.0 (d × 2), 130.6 (d × 2), 131.8 (d), 132.4 (d × 2), 135.3 (s), 135.6 (s), 138.8 (s), 145.1 (s); MS (ESI-TOF) m/z 653 $[M + Na]^+$. HRMS (ESI-TOF) m/z: $[M+Na]^+$ Calcd for $C_{28}H_{27}N_2O_4S_3BrNa$ 653.0214; found 653.0232. Anal. Calcd for $C_{28}H_{27}N_2O_4S_3Br$: C, 53.25; H, 4.31; N, 4.44. Found: C, 53.13; H, 4.19; N, 4.45.

Oxidation of *N*-Allyl-*N*-[3-(phenylsulfinylmethyl)-1-tosylpyrrole-4-methyl]-4-bromobenzenesulfonamide **4g** with mCPBA.

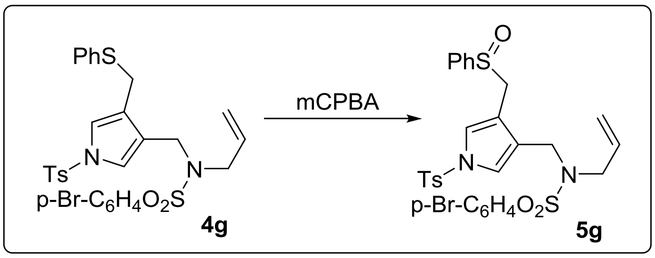

To 1,2-dichloroethane (15 mL) solution of *N*-allyl-*N*-[3-(phenylsulfanylmethyl)-1-tosylpyrrole-4-methyl]-4-brormobenzene sulfonamide (**4g**) (264 mg, 0.42 mmol) was added *m*-chloroperbenzoic acid (72 mg, 0.42 mmol) over 1 h at 0 °C. The reaction mixture was further stirred for 30 min and poured into a sat. $NaHCO_3$ (50 mL). The whole was vigorously stirred for 15 min. The organic layer was separated and the aqueous layer was extracted with chloroform. The combined organic layer was dried over $MgSO_4$. The solvent was removed under reduced pressure. The residue was purified by the preparative TLC on silica gel eluting with AcOEt-*n*-hexane (1:2) to give *N*-allyl-*N*-[3-(phenylsulfinylmethyl)-1-tosylpyrrole-4-methyl]-4-bromobenzenesulfonamide (**5g**) (250 mg, 92%) as white powders.

mp 116–117 °C, IR ν 3445, 2923, 1711, 1575, 1444, 1373, 1347, 1306, 1172, 1091, 1067, 903, 813, 749, 704, 672, 588, 540; ^{1}H NMR (600 MHz, $CDCl_3$) δ 2.44 (3H, s, Me),

3.59 (1H, dd, J = 6.8 and 15.8 Hz, CH), 3.66 (1H , dd, J = 6.2 and 15.8 Hz, CH) 3.82 (1H, d, J = 13.8 Hz, CH), 3.83 (1H, d, J = 4.4 Hz, CH), 3.87 (1H, d, J = 4.4 Hz, CH), 4.11 (1H, d, J = 13.7 Hz, CH), 4.89 (1H, dd, J = 1.4 and 17.2 Hz, olefinic H), 4.98 (1H, d, J = 8.9 Hz, olefinic H), 5.23-5.29 (1H, m, olefinic H), 6.90 (1H, d, J = 2.1 Hz, ArH), 7.04 (1H, d, J=2.0 Hz, ArH), 7.35 (4H, t, J=6.9 Hz, ArH), 7.41-7.45 (3H, m, ArH), 7.61 (2H, d, J = 8.9 Hz, ArH), 7.65 (2H, d, J = 8.9 Hz, ArH), (4H, dd, J=8.9 and 19.9 Hz, ArH), 7.74 (2H, d, J=8.2 Hz, ArH); ^{13}C NMR (150 MHz, $CDCl_3$) δ 21.6 (q), 41.7 (t), 49.2 (t), 52.5 (t), 115.9 (s), 119.7 (t), 120.5 (d), 122.1 (s), 122.4 (d), 124.3 (d × 2), 127.0 (d × 2), 127.7 (s), 128.5 (d × 2), 128.7 (d × 2), 130.0 (d × 2), 130.9 (d), 131.3 (d), 132.4 (d × 2), 135.4 (s), 138.5 (s), 142.9 (s), 145.4 (s); MS (ESI-TOF) m/z 669 $[M + Na]^+$. HRMS (ESI-TOF) m/z: $[M+Na]^+$ Calcd for $C_{28}H_{27}N_2O_5S_3BrNa$ 669.0163; found 669.0179. Anal. Calcd for $C_{28}H_{27}N_2O_5S_3Br$: C, 51.93; H, 4.20; N, 4.33. Found: C, 51.88; H, 4.19; N, 4.25.

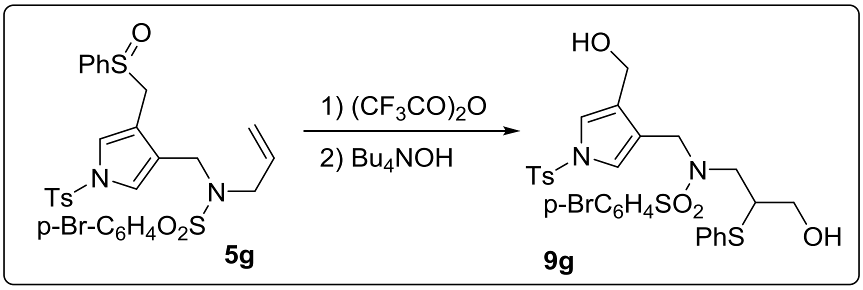

Pummerer reaction of sulfoxide **5g** with TFAA/TBAH.

Trifluoroacetic anhydride (340.5 mg, 1.62 mmol) was added dropwise to a dichloromethane (8 mL) solution of *N*-allyl-*N*-[(3-(phenylsulfinylmethyl)-1-tosylpyrrole-4-yl)methyl]-4-bromobenzenesulfon-amide (**5g**) (210 mg, 0.32 mmol) at −20 °C. The reaction mixture was stirred for 1.5 h and poured into a sat. sodium hydrogen carbonate (50 mL). The organic layer was separated and the aqueous layer was extracted with chloroform. The combined organic layer was dried over $MgSO_4$. The solvent was removed under reduced pressure. To the residue in dichloromethane (10 mL) were added tetrabutylammonium hydroxide (0.84 g, 3.24 mmol) in H_2O (8 mL) and tetrabutylammonium hydrogensulfate (22.0 mg, 0.06 mmol) at room temperature. The mixture was stirred for 0.5 h and poured into water (50 mL). The organic layer was separated and the aqueous layer was extracted with chloroform. The combined organic layer was dried over $MgSO_4$. The solvent was removed under reduced pressure. The residue was purified by preparative TLC on silica

gel eluting with AcOEt-*n*-hexane (1.5:1) to give *N*-(3-hydroxy-2-(phenylthio)propyl)-*N*-((4-(hydroxymethyl)-1-tosyl-1*H*-pyrrol-3-yl)methyl)-4-bromobenzenesulfonamide (**9g**) (100 mg, 46 %) as white powder.

mp 49–51 ^{o}C, IR ν 3529, 3409, 2925, 1574, 1520, 1471, 1440, 1371, 1301, 1171, 1092, 1068, 1010, 917, 814, 753, 703, 673, 588, 540, 422; ^{1}H NMR (600 MHz, $CDCl_3$) δ 2.37 (3H, s, Me), 2.52 (2H, brs, OH × 2), 2.98 (1H, dd, *J* = 4.8 and 14.4 Hz, CH), 3.07 (1H, dd, *J* = 4.8 and 9.0 Hz, CH), 3.44 (1H, dd, *J* = 10.3 and 15.1 Hz, CH), 3.52 (1H, dd, *J* = 3.4 and 12.3 Hz, CH), 3.60 (1H, dd, *J* = 3.4 and 12.4 Hz, CH), 3.94 (1H, d, *J* = 14.4 Hz, CH), 4.24 (1H, d, *J* = 14.5 Hz, CH), 4.45 (1H, d, *J* = 13.1 Hz, CH), 4.49 (1H, d, *J* = 13.1 Hz, CH), 6.91 (1H, d, *J* = 2.1 Hz, CH), 7.08 (1H, d, *J* = 2.1 Hz, ArH), 7.25-7.31 (5H, m, ArH), 7.52 (2H, d, *J* = 8.3 Hz, ArH), 7.60 (2H, d, *J* = 9.0 Hz, ArH), 7.70 (2H, d, *J* = 8.2 Hz, ArH); ^{13}C NMR (150 MHz, $CDCl_3$) δ 21.6 (q), 46.1 (t), 50.1 (t), 50.3 (d), 56.2 (t), 61.3 (t), 119.9 (d), 120.9 (d), 121.8 (s), 126.9 (d × 2), 127.6 (d), 127.7 (s), 128.3 (s), 128.7 (d × 2), 129.2 (d × 2), 130.1 (d × 2), 132.1 (d × 2), 132.6 (d × 2), 133.1 (s), 135.5 (s), 136.4 (s), 145.5 (s); MS (ESI-TOF) m/z 687 $[M + Na]^+$. HRMS (ESI-TOF) m/z: $[M+Na]^+$ Calcd for $C_{28}H_{29}N_2O_6S_3BrNa$ 687.0269; found 687.0244.

Ytterbium-catalyzed cyclization of diol **9g**.

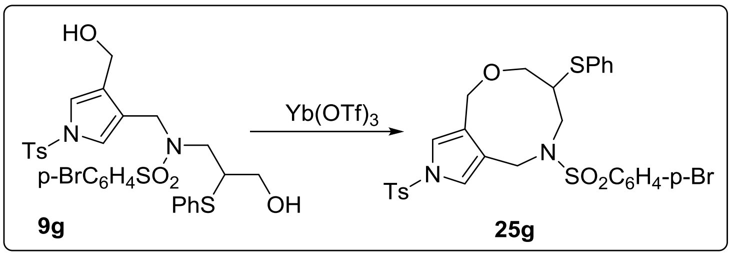

Ytterbium(III) trifluoromethanesulfonate hydrate (18.6 mg, 0.03 mmol) was added dropwise to a dichloroethane (5.0 mL) solution of *N*-(3-hydroxy-2-(phenylthio)propyl)-*N*-((4-(hydroxymethyl)-1-tosyl-1*H*-pyrrol-3-yl)methyl)-4-bromobenzenesulfonamide (**9g**) (100 mg, 0.15 mmol). The reaction mixture was stirred at reflux for 4.5 h and then poured into sodium hydrogen carbonate (50 mL). The organic layer was separated and the aqueous layer was extracted with chloroform. The combined organic layer was dried over $MgSO_4$. The solvent was removed under reduced pressure. The residue was purified by column chromatography on silica gel eluting with AcOEt-*n*-hexane (1:2) to give

7-(phenylthio)-2-tosyl-9-*p*-bromobenzenesulfonamide(2,6,7,8,9,10-hexahydro-4*H*-pyrrolo [3,4-*g*][1,5]oxazonine (**25g**) (54 mg, 56%) as white powders.

mp 188–190 °C, IR ν 3421, 2925, 2856, 1711, 1574, 1515, 1470, 1371, 1305, 1173, 1093, 1068, 1009, 963, 813, 766, 674, 595, 539, 421; ^{1}H NMR (600 MHz, $CDCl_3$) δ 2.41 (3H, s, Me), 3.15 (1H, dd, *J* = 11.0 and 14.5 Hz, CH), 3.53 (2H, d, *J* = 8.9 Hz, CH × 2), 3.62 (1H, d, *J* = 4.8 and 14.4 Hz, CH), 3.81-3.86 (2H, m, CH × 2), 4.44 (1H, d, *J* = 13.7 Hz, CH), 4.56 (1H, d, *J* = 15.2 Hz, CH), 4.74 (1H, d, *J* = 13.7 Hz, CH), 6.95 (1H, s, ArH), 7.24-7.26 (1H, m, ArH), 7.30-7.32 (5H, m, ArH), 7.38 (2H, d, *J* = 6.9 Hz, ArH), 7.48 (2H, d, *J* = 9.0 Hz, ArH), 7.53 (2H, d, *J* = 8.9 Hz, ArH), 7.77 (2H, d, *J* = 9.3 Hz, ArH); ^{13}C NMR (150 MHz, $CDCl_3$) δ 21.6 (q), 47.4 (d), 48.6 (t), 53.6 (t), 64.9 (t), 66.4 (t), 118.4 (d), 122.9 (d), 123.2 (s), 124.6 (s), 126.9 (d × 2), 127.0 (d), 127.6 (s), 128.5 (d × 2), 129.1 (d × 2), 130.1 (d × 2), 130.9 (d × 2), 132.3 (d × 2), 134.0 (s), 135.7 (s), 137.9 (s), 145.2 (s); MS (ESI-TOF) m/z 669 $[M + Na]^+$. HRMS (ESI-TOF) m/z: $[M+Na]^+$ Calcd for $C_{28}H_{27}N_2O_5S_3BrNa$ 669.0163; found 669.0189. Anal. Calcd for $C_{28}H_{27}N_2O_5S_3Br$+1/4H_2O: C, 51.57; H, 4.25; N, 4.30. Found: C, 51.52; H, 4.10; N, 4.23.

6. X-ray crystallographic analysis

Data of sulfone derivative of **5a** and pyrroloazepine **12d** were taken on a Rigaku AFC5R diffractometer with graphite-monochromated Mo-Kα radiation (λ = 0.71069 Å). The structures of sulfone derivative of **5a** and **12d** were solved by direct methods with SIR97. Full-matrix least-squares refinement was employed with anisotropic thermal parameters for all non-hydrogen atoms. All calculations were performed using the Crystal Structure (Ver. 3.8) crystallographic software package. ORTEP drawings of sulfone derivative of **5a** and **12d** are shown in Fig. 2 and Fig. 1, respectively. The data of sulfone derivative of **5a** and **12d** have been deposited with the Cambridge Crystallographic Data Centre as supplementary publication number CCDC 1824587 and CCDC 1824588, respectively.

Crystal data for sulfone derivative of **5a**.

Monoclinic, space group $P2_1/n$, *a* = 8.8310(3), *b* = 3.1020(5), *c* = 25.1669(11) Å, β = 91.4836(13)°, *V* = 2910.9(2) Å^3, *Z* = 4, μ(Mo-Kα) = 2.997 cm^{-1}, *F*(000) = 1256, *D*c = 1.366 g/cm^3, crystal dimensions: 0.25 x 0.20 x 0.20 mm. A total of 27881 reflections (6657 unique) were collected using the ω-2θ scan technique to a maximum 2θ value of 55°, and

all reflections were used in the structure determination. Final *R* and *R*w values were 0.086 and 0.180, respectively. The maximum and minimum peaks in the difference map were 17.3 e^- Å^{-3} and –4.66 e^- Å^{-3}, respectively.

Crystal data for **12d**.

Tetragonal, space group *P*41, *a* = *b* = 14.8202(6) Å, *c* = 14.7217(6) Å, *V* = 3233.4(2) Å^3, *Z* = 4, μ(Mo-Kα) = 2.746 cm^{-1}, *F*(000) = 1384, *D*c = 1.353 g/cm^3, crystal dimensions: 0.30 x 0.20 x 0.10 mm. A total of 30495 reflections (7244 unique) were collected using the ω-2θ scan technique to a maximum 2θ value of 55°, and all reflections with $I > 2\sigma(I)$ were used in the structure determination. Final *R* and *R*w values were 0.057 and 0.091, respectively. The maximum and minimum peaks in the difference map were 13.1 e^- Å^{-3} and –3.66 e^- Å^{-3}, respectively.

7. References

S1) M. J. Frisch, G. W. Trucks, H. B. Schlegel, G. E. Scuseria, M. A. Robb, J. R. Cheeseman, G. Scalmani, V. Barone, G. A. Petersson, H. Nakatsuji, X. Li, M. Caricato, A. V. Marenich, J. Bloino, B. G. Janesko, R. Gomperts, B. Mennucci, H. P. Hratchian, J. V. Ortiz, A. F. Izmaylov, J. L. Sonnenberg, D. Williams-Young, F. Ding, F. Lipparini, F. Egidi, J. Goings, B. Peng, A. Petrone, T. Henderson, D. Ranasinghe, V. G. Zakrzewski, J. Gao, N. Rega, G. Zheng, W. Liang, M. Hada, M. Ehara, K. Toyota, R. Fukuda, J. Hasegawa, M. Ishida, T. Nakajima, Y. Honda, O. Kitao, H. Nakai, T. Vreven, K. Throssell, J. A. Montgomery, Jr., J. E. Peralta, F. Ogliaro, M. J. Bearpark, J. J. Heyd, E. N. Brothers, K. N. Kudin, V. N. Staroverov, T. A. Keith, R. Kobayashi, J. Normand, K. Raghavachari, A. P. Rendell, J. C. Burant, S. S. Iyengar, J. Tomasi, M. Cossi, J. M. Millam, M. Klene, C. Adamo, R. Cammi, J. W. Ochterski, R. L. Martin, K. Morokuma, O. Farkas, J. B. Foresman, and D. J. Fox, Gaussian, Inc., Wallingford CT, 2016.

S2) Kobayashi, Y.; Tanahashi, R.; Yamaguchi, Y.; Hatae, N.; Kobayashi, M.; Ueno, Y.; Yoshimatsu, M. *J. Org. Chem.* **2017**, *82*, 2436–2449.

S3) Ito, Y.; Yoshimatsu, M. *Chem. Lett.*, **2014**, *43*, 1758–1760.

S4) Cabrera-Lobera, N.; Rodríguez-Salamanca, P.; Nieto-Carmona, J. C.; Buñuel, E.; Cárdenas, D. J. *Chem. Eur. J.* **2018**, *24*, 784–788.

S5) Dandapani, S.; Curran, D. P. *Tetrahedron* **2002**, *58*, 3855–3864.

S6) Jones, A. D.; Knight, D. W.; Hibbs, D. E. *J. Chem. Soc. Perkin Trans. 1* 2001, 1182–1203.

S7) Kataoka, T.; Yoshimatsu, M.; Noda, Y.; Sato, T.; Shimizu, H.; Hori, M. *J. Chem. Soc. Perkin Trans. 1* 1993, 121–129.

S8) Liu, B.; Song, R.-J.; Ouyang, X.-H.; Li, Y.; Hu, M.; Li, J.-H. *Chem. Commun*. **2015**, *51*, 12819-12822.

S9) Oppolzer, W.; Stammen, B. *Tetrahedron* **1997**, *53*, 3577-3586.

S10) Qi, X.; Yu, F.; Chen, P.; Liu, G. *Angew. Chem. Int. Ed.* **2017**, *56*, 12692-12696.

S11) Wilden, J. D.; Geldeard, L.; Lee, C. C; Judd, D. B.; Caddick, S. *Chem. Commun.* **2007**, 1074-1076.

8. DFT Computational results, Cartesian coordinates, computed total energies of optimized structures

----

molecule No.14a

----

optimized geometry

	x	y	z
C	-2.085560	-1.053060	-1.156055
C	-0.747163	-0.907943	-1.019121
C	-0.172526	-2.262515	-1.143344
C	-1.296247	-3.159407	-1.352424
N	-2.386501	-2.431771	-1.343441
S	-4.071723	-3.109423	-1.681604
O	-3.790854	-4.533763	-1.778823
O	-4.527180	-2.286444	-2.785776
C	-4.931446	-2.715809	-0.194118
C	-4.977498	-3.663881	0.835338
C	-5.677060	-3.347210	1.994058
C	-6.325909	-2.109054	2.139624
C	-6.256690	-1.182755	1.085465
C	-5.569460	-1.472859	-0.088075
C	-7.109762	-1.794739	3.387468

C	1.110639	-2.685361	-1.074038
S	5.580326	-2.546231	0.658256
C	6.864270	-1.781486	-0.284604
C	8.177624	-1.770795	0.215713
C	9.201898	-1.218439	-0.550270
C	8.919192	-0.677297	-1.807389
C	7.613210	-0.686593	-2.307550
C	6.583089	-1.239415	-1.551271
C	0.039539	0.379107	-0.872336
N	-0.651465	1.503700	-0.247686
C	-0.690693	1.583470	1.228038
S	-1.867437	2.279685	-1.134559
O	-3.191350	1.900657	-0.600422
O	-1.547401	1.997444	-2.538191
C	-1.612819	4.008331	-0.773860
C	-0.470596	4.646787	-1.269379
C	-0.299155	6.003770	-1.024182
C	-1.251991	6.739730	-0.298064
C	-2.387242	6.073295	0.179832
C	-2.579784	4.711813	-0.055666
C	-1.059945	8.217810	-0.064287

O	5.009688	-1.154480	1.616813
C	3.928929	-0.509484	1.200924
O	3.249913	-0.709620	0.220056
C	3.551782	0.595852	2.224019
F	2.798874	0.046931	3.199314
F	2.819122	1.540102	1.610373
F	4.619095	1.170150	2.774423
C	-1.424991	0.466725	1.924684
C	-0.845165	-0.352946	2.802843
H	-2.881458	-0.322514	-1.134511
H	-1.317856	-4.232488	-1.495043
H	-4.494339	-4.628140	0.720147
H	-5.728670	-4.076148	2.797789
H	-6.756304	-0.223096	1.180692
H	-5.540940	-0.759674	-0.905170
H	-8.155177	-2.108893	3.270308
H	-6.707304	-2.319715	4.258797
H	-7.114218	-0.721470	3.599450
H	1.940414	-2.010083	-0.881370
H	1.349702	-3.739043	-1.194365
H	8.382796	-2.195642	1.193042

H	10.218449	-1.210027	-0.168864
H	9.720776	-0.249077	-2.402420
H	7.402216	-0.267399	-3.286869
H	5.564985	-1.255578	-1.926923
H	0.361933	0.691459	-1.871613
H	0.946550	0.199794	-0.285696
H	-1.143362	2.549278	1.479719
H	0.347852	1.625059	1.572532
H	0.264775	4.091005	-1.841888
H	0.585879	6.505909	-1.406257
H	-3.136429	6.626225	0.739885
H	-3.466740	4.200778	0.302219
H	-1.249653	8.785178	-0.984313
H	-0.034006	8.442653	0.247034
H	-1.740627	8.595716	0.703813
H	-2.484911	0.374427	1.693173
H	-1.405118	-1.126835	3.321231
H	0.210732	-0.268478	3.051789

----

total energy   -3253.86256444   a.u.

----

number of imaginary frequencies 0

----

molecule No.14b

----

optimized geometry

	x	y	z
C	1.987391	-0.162176	1.294311
C	0.837537	-0.089891	0.550999
C	0.914047	1.133239	-0.213471
C	2.112330	1.747193	0.101951
N	2.754520	0.958738	1.019997
S	4.309957	1.336584	1.752616
O	4.223976	0.742275	3.079595
O	4.457390	2.766408	1.508509
C	5.490554	0.436062	0.774352
C	5.864786	-0.850400	1.171742

C	6.802560	-1.539870	0.408059
C	7.374765	-0.964340	-0.736981
C	6.980163	0.331927	-1.104257
C	6.044532	1.041065	-0.357188
C	8.419407	-1.705797	-1.533136
C	-0.094634	1.648183	-1.165640
S	-1.151759	2.998039	-0.363700
C	-2.415722	3.424587	-1.515791
C	-2.635072	4.806027	-1.663913
C	-3.578545	5.233683	-2.594244
C	-4.295032	4.294312	-3.340644
C	-4.078445	2.921808	-3.165877
C	-3.132719	2.465879	-2.252631
C	-0.272241	-1.104896	0.549158
N	-0.413800	-1.787977	-0.754900
C	0.545060	-2.869842	-1.044944
C	1.693749	-2.502808	-1.970109
S	-1.962447	-1.898691	-1.417948
O	-1.792260	-2.479511	-2.747994

O	-2.535206	-0.545388	-1.246757
C	-2.948504	-3.019202	-0.432717
C	-3.639298	-2.550111	0.688844
C	-4.366167	-3.453431	1.460636
C	-4.421611	-4.816079	1.131600
C	-3.726208	-5.255289	-0.004941
C	-2.993147	-4.370267	-0.791231
C	-5.239841	-5.779069	1.955942
O	-1.910440	1.937945	0.742001
C	-2.532710	2.551638	1.802314
O	-2.623204	3.729302	1.970646
C	-3.136474	1.454351	2.716258
F	-2.208097	0.529217	3.004979
F	-4.150342	0.855780	2.068942
F	-3.583993	2.002307	3.835380
C	1.648157	-1.473929	-2.816544
C	2.864887	-3.449433	-1.891177
H	2.321628	-0.894704	2.013657
H	2.551244	2.678464	-0.225623

H	5.448210	-1.289279	2.071966
H	7.105240	-2.537798	0.713456
H	7.420744	0.796574	-1.982181
H	5.764316	2.052251	-0.632032
H	8.289038	-2.789532	-1.457031
H	8.390372	-1.428125	-2.591282
H	9.425449	-1.470909	-1.161989
H	-0.804055	0.890831	-1.507871
H	0.326250	2.191920	-2.017833
H	-2.084177	5.523746	-1.063425
H	-3.759897	6.295213	-2.727643
H	-5.034342	4.632773	-4.060418
H	-4.650136	2.202128	-3.743157
H	-2.978971	1.401632	-2.099649
H	-1.220186	-0.617793	0.779395
H	-0.089024	-1.845319	1.339382
H	0.009804	-3.720959	-1.482442
H	0.946157	-3.227829	-0.086294
H	-3.632810	-1.495266	0.940005

H	-4.909022	-3.091799	2.330036
H	-3.766904	-6.304644	-0.284876
H	-2.485256	-4.713636	-1.686033
H	-6.252333	-5.879761	1.543885
H	-5.340792	-5.437704	2.990652
H	-4.793034	-6.778159	1.966028
H	2.471857	-1.263133	-3.492705
H	0.779267	-0.827982	-2.879103
H	3.331894	-3.423901	-0.896523
H	3.629162	-3.202049	-2.632748
H	2.551773	-4.488197	-2.064853

----

total energy -3293.18847015 a.u.

----

number of imaginary frequencies 0

----

molecule No.14c

----

optimized geometry

	x	y	z
C	-2.045835	-1.009078	-1.268847
C	-0.708214	-0.878275	-1.112185
C	-0.143187	-2.234790	-1.258011
C	-1.271877	-3.118253	-1.497045
N	-2.355766	-2.381073	-1.486547
S	-4.044137	-3.038649	-1.841865
O	-3.782159	-4.467590	-1.923536
O	-4.478135	-2.220620	-2.958273
C	-4.912416	-2.617718	-0.366417
C	-4.964759	-3.546165	0.680283
C	-5.665756	-3.204994	1.831553
C	-6.310628	-1.961802	1.951979
C	-6.234863	-1.055654	0.880825
C	-5.545201	-1.370281	-0.284946
C	-7.098334	-1.621338	3.190578

C	1.135937	-2.668891	-1.184729
S	5.660888	-2.535196	0.623007
C	6.927746	-1.745117	-0.322209
C	8.239228	-1.697962	0.180880
C	9.251253	-1.125809	-0.586861
C	8.958189	-0.601161	-1.848595
C	7.654061	-0.646763	-2.351561
C	6.636218	-1.219537	-1.593477
C	0.085386	0.399015	-0.925183
N	-0.610945	1.516857	-0.295406
C	-0.690554	1.565317	1.182558
S	-1.797463	2.318859	-1.196946
O	-3.138774	1.941545	-0.706165
O	-1.444297	2.063146	-2.597996
C	-1.539551	4.038308	-0.795129
C	-0.376239	4.675409	-1.240555
C	-0.201223	6.026021	-0.964331
C	-1.171153	6.757175	-0.256354
C	-2.327251	6.092435	0.171238

C	-2.523508	4.737374	-0.096085
C	-0.974616	8.228967	0.011215
O	5.052884	-1.152070	1.571214
C	3.963113	-0.531083	1.142422
O	3.298809	-0.747655	0.155150
C	3.551465	0.568803	2.158085
F	2.789148	0.008940	3.119995
F	2.815531	1.501593	1.530754
F	4.599451	1.159676	2.727706
C	-1.438782	0.435518	1.838920
C	-0.879866	-0.405788	2.714524
C	-1.597927	-1.508465	3.437083
H	-2.835423	-0.271831	-1.240319
H	-1.301302	-4.188528	-1.658088
H	-4.485403	-4.514452	0.584363
H	-5.723374	-3.919036	2.648141
H	-6.730541	-0.092253	0.956711
H	-5.509608	-0.671939	-1.114447
H	-8.148635	-1.916190	3.067264

H	-6.713295	-2.145680	4.070039
H	-7.084073	-0.546161	3.392551
H	1.968603	-2.003848	-0.970395
H	1.368533	-3.721595	-1.324776
H	8.452612	-2.110530	1.161708
H	10.266371	-1.089281	-0.203285
H	9.750289	-0.157489	-2.445012
H	7.435043	-0.240237	-3.334458
H	5.619707	-1.263884	-1.971111
H	0.432197	0.726735	-1.911293
H	0.978102	0.201644	-0.322653
H	-1.149057	2.527469	1.437638
H	0.339460	1.603126	1.552464
H	0.373208	4.123478	-1.798349
H	0.700432	6.526874	-1.307257
H	-3.089687	6.641721	0.716859
H	-3.425756	4.227638	0.223298
H	-1.139424	8.815833	-0.901386
H	0.045268	8.440428	0.350442

H	-1.670453	8.596460	0.770791
H	-2.495972	0.348782	1.589165
H	0.179659	-0.288889	2.949977
H	-1.113393	-2.479230	3.265581
H	-2.644453	-1.585581	3.123134
H	-1.580238	-1.342696	4.522274

----

total energy -3293.18300808 a.u.

----

number of imaginary frequencies 0

----

molecule No.14d

----

optimized geometry

	x	y	z
C	1.727902	-0.297164	-1.708880

C	0.376257	-0.321027	-1.662191
C	-0.089485	0.774724	-2.535312
C	1.107521	1.391802	-3.077553
N	2.139303	0.765871	-2.567934
S	3.898792	1.183300	-2.973299
O	3.752971	1.846776	-4.259147
O	4.561637	-0.089606	-2.766036
C	4.296632	2.346151	-1.708699
C	4.274120	3.711015	-2.017417
C	4.586020	4.620956	-1.012514
C	4.906667	4.191248	0.285098
C	4.918256	2.812386	0.557963
C	4.623388	1.880347	-0.428985
C	5.255505	5.181849	1.365477
C	-1.342122	1.202234	-2.811888
S	-4.980870	3.225897	-0.707265
C	-6.551412	2.418672	-0.641528
C	-7.636155	3.066893	-0.026180
C	-8.890028	2.459626	-0.023641

C	-9.062155	1.211642	-0.628581
C	-7.984328	0.564286	-1.241060
C	-6.728002	1.164398	-1.252369
C	-0.539034	-1.314676	-0.982860
N	0.090379	-2.358673	-0.187707
C	0.266166	-2.187658	1.269449
S	1.007630	-3.531823	-0.991103
O	2.425344	-3.351173	-0.634841
O	0.574943	-3.448477	-2.390695
C	0.466067	-5.078656	-0.280807
C	-0.794515	-5.581579	-0.620072
C	-1.198114	-6.801296	-0.089617
C	-0.362225	-7.534645	0.770218
C	0.896555	-7.008029	1.086289
C	1.321589	-5.786307	0.564171
C	-0.805380	-8.870885	1.313103
O	-4.232467	2.625066	0.795329
C	-3.442393	1.561805	0.718443
O	-3.157644	0.888947	-0.244674

C	-2.819593	1.277289	2.112675
F	-3.670421	1.506345	3.110303
F	-1.733327	2.061120	2.274463
F	-2.419359	-0.005038	2.171040
C	1.407875	-1.317578	1.725451
C	1.226767	-0.218035	2.473341
C	2.263678	0.622833	3.091755
C	3.592787	0.191945	3.264668
C	4.528994	1.004880	3.900305
C	4.159963	2.267884	4.377479
C	2.845302	2.709466	4.211875
C	1.908230	1.892558	3.580149
H	2.467389	-0.962446	-1.286620
H	1.210286	2.217168	-3.770543
H	4.038128	4.045428	-3.021733
H	4.582702	5.683099	-1.240207
H	5.152663	2.464004	1.558934
H	4.653884	0.817877	-0.212217
H	6.344835	5.264871	1.472875

H	4.870246	6.179998	1.138804
H	4.856448	4.863882	2.333772
H	-2.218595	0.763242	-2.345981
H	-1.509188	2.030135	-3.496038
H	-7.488273	4.036394	0.438545
H	-9.731492	2.957820	0.448214
H	-10.041651	0.742031	-0.626049
H	-8.127013	-0.403769	-1.712019
H	-5.882397	0.674689	-1.724355
H	-1.143943	-1.787334	-1.765312
H	-1.235412	-0.782155	-0.324952
H	0.382690	-3.195379	1.687441
H	-0.682047	-1.801646	1.657387
H	-1.439987	-5.032533	-1.297855
H	-2.175671	-7.198273	-0.351008
H	1.559210	-7.563044	1.744793
H	2.304400	-5.389992	0.794676
H	-0.710230	-9.652289	0.548246
H	-1.855799	-8.848502	1.622544

H	-0.201866	-9.176036	2.172775
H	2.404551	-1.670228	1.467099
H	0.203869	0.100887	2.673418
H	3.883952	-0.802483	2.938247
H	5.542822	0.642461	4.049193
H	4.885462	2.889018	4.895747
H	2.543918	3.681459	4.592988
H	0.881412	2.233037	3.467083

----

total energy -3484.92254982 a.u.

----

number of imaginary frequencies 0

----

molecule No.15a

----

optimized geometry

	x	y	z
C	-2.201562	-0.535183	0.181536
C	-1.186017	-0.069159	-0.618187
C	-1.463703	1.317978	-0.877875
C	-2.638984	1.642598	-0.224839
N	-3.078195	0.510184	0.409878
S	-4.543299	0.428691	1.400941
O	-4.846786	1.832937	1.649244
O	-4.207854	-0.529346	2.443125
C	-5.763536	-0.271265	0.315032
C	-5.957379	-1.656463	0.312793
C	-6.932841	-2.192048	-0.521574
C	-7.716840	-1.370449	-1.348379
C	-7.503297	0.015547	-1.312183
C	-6.532911	0.576141	-0.485535
C	-8.763160	-1.972449	-2.252722
C	-0.603132	2.235772	-1.648659
S	0.669343	3.052753	-0.510302
C	1.800242	3.939283	-1.586291

C	2.078323	5.260929	-1.214003
C	2.944130	6.019173	-2.002894
C	3.512936	5.463740	-3.149910
C	3.220595	4.146812	-3.517559
C	2.361562	3.373664	-2.738694
C	-0.006539	-0.856585	-1.113058
N	1.193935	-0.710474	-0.264373
C	2.074771	0.427423	-0.468126
S	1.903557	-2.166352	0.322211
O	0.766855	-3.026672	0.642183
O	2.879435	-1.723767	1.321341
C	2.811777	-2.910242	-1.028247
C	2.158157	-3.790749	-1.895870
C	2.870030	-4.351675	-2.953029
C	4.227240	-4.057746	-3.155135
C	4.859267	-3.180323	-2.261334
C	4.166007	-2.607131	-1.197786
C	4.998214	-4.705855	-4.278488
C	1.718102	1.718962	0.322373

C	1.144447	1.470707	1.725442
O	2.264217	1.020112	2.499439
C	2.009052	0.066038	3.427881
O	0.931961	-0.385085	3.697489
C	3.312196	-0.295645	4.172854
F	3.510318	0.617413	5.145172
F	4.375987	-0.277843	3.358600
F	3.200132	-1.499254	4.726722
H	-2.359072	-1.505069	0.629743
H	-3.187821	2.569243	-0.144245
H	-5.375947	-2.293058	0.971092
H	-7.099014	-3.265983	-0.521385
H	-8.114989	0.667180	-1.929828
H	-6.393401	1.650931	-0.441097
H	-9.371369	-2.710611	-1.718955
H	-9.432181	-1.208931	-2.659066
H	-8.295483	-2.491347	-3.099141
H	-1.128577	3.104627	-2.053475
H	-0.045514	1.748724	-2.450856

H	1.626845	5.690517	-0.324880
H	3.167592	7.043079	-1.720589
H	4.183299	6.058104	-3.763060
H	3.660900	3.719840	-4.413229
H	2.138656	2.354554	-3.036405
H	0.243696	-0.575361	-2.147736
H	-0.274386	-1.912835	-1.123732
H	2.198767	0.654160	-1.536551
H	3.060323	0.153069	-0.084264
H	1.120861	-4.058258	-1.725108
H	2.367107	-5.043281	-3.623851
H	5.914902	-2.955558	-2.389367
H	4.674798	-1.964245	-0.487380
H	5.837344	-4.082766	-4.602712
H	4.358698	-4.900544	-5.145132
H	5.412815	-5.669979	-3.956894
H	2.641897	2.296562	0.438958
H	0.358969	0.714131	1.721008
H	0.763039	2.394587	2.171792

----

total energy   -3253.90585342   a.u.

----

number of imaginary frequencies   0

----

molecule No.15b

----

optimized geometry

	x	y	z
C	-1.833045	-0.424486	-0.464056
C	-0.694870	0.096785	-1.028049
C	-0.877960	1.519776	-1.105487
C	-2.124633	1.814102	-0.584594
N	-2.695693	0.625747	-0.203575
S	-4.231409	0.496065	0.663191
O	-4.529495	1.881006	1.008659

O	-3.995512	-0.550903	1.646749
C	-5.375950	-0.085520	-0.564476
C	-5.607505	-1.458803	-0.681828
C	-6.518170	-1.902719	-1.636541
C	-7.202006	-1.000720	-2.466122
C	-6.949002	0.372388	-2.317045
C	-6.043466	0.841190	-1.370489
C	-8.214485	-1.492361	-3.470022
C	0.133410	2.475039	-1.591880
S	1.310012	3.037837	-0.205756
C	2.682018	3.790584	-1.083756
C	3.327741	3.176251	-2.165808
C	4.363666	3.852816	-2.807612
C	4.748074	5.125854	-2.376491
C	4.095541	5.732846	-1.302362
C	3.055910	5.069563	-0.650063
C	0.562061	-0.651989	-1.367390
N	1.482096	-0.746326	-0.217282
C	2.464435	0.291122	0.034808

C	2.008842	1.548369	0.842080
C	3.231625	2.181728	1.523704
C	0.870580	1.275134	1.855040
S	1.732721	-2.297648	0.468417
O	0.469963	-3.002513	0.245153
O	2.278205	-2.039396	1.801374
C	3.003759	-3.111401	-0.492957
C	2.648229	-3.836768	-1.634220
C	3.647843	-4.451097	-2.383906
C	4.997663	-4.363266	-2.009894
C	5.322720	-3.638956	-0.853478
C	4.338544	-3.015957	-0.089271
C	6.066848	-5.069208	-2.806767
O	1.484789	0.629739	2.976699
C	0.832367	-0.170378	3.860094
O	1.398387	-0.610624	4.815601
C	-0.670211	-0.461759	3.602637
F	-0.834278	-1.168368	2.464650
F	-1.350783	0.702120	3.468557

F	-1.184226	-1.144508	4.612116
H	-2.075171	-1.436622	-0.177240
H	-2.646133	2.748502	-0.439518
H	-5.101675	-2.159030	-0.025732
H	-6.708762	-2.968129	-1.732111
H	-7.477023	1.084776	-2.945058
H	-5.871279	1.904651	-1.243727
H	-9.214749	-1.533972	-3.019661
H	-8.275522	-0.828133	-4.337541
H	-7.973992	-2.499778	-3.822888
H	-0.281743	3.433560	-1.914018
H	0.765341	2.076432	-2.386744
H	3.035366	2.191534	-2.514595
H	4.869104	3.385047	-3.646765
H	5.554793	5.646158	-2.883575
H	4.389399	6.723994	-0.971615
H	2.540192	5.540955	0.180783
H	1.092779	-0.179091	-2.206177
H	0.308311	-1.663587	-1.683657

H	2.949406	0.613198	-0.897469
H	3.248895	-0.150022	0.654881
H	3.566053	1.494228	2.304072
H	2.993042	3.134523	2.007335
H	4.049988	2.339056	0.816223
H	0.108020	0.648558	1.398710
H	0.424162	2.215027	2.194786
H	1.604937	-3.946389	-1.910402
H	3.373924	-5.022762	-3.266791
H	6.360362	-3.575177	-0.536464
H	4.595406	-2.494165	0.826498
H	7.034949	-4.567488	-2.714106
H	5.807230	-5.123172	-3.868680
H	6.197851	-6.099322	-2.450961

----

total energy -3293.20889726 a.u.

----

number of imaginary frequencies 0

----

molecule No.15c

----

optimized geometry

	x	y	z
C	-2.744599	-0.892851	0.147492
C	-1.502216	-0.405187	-0.160534
C	-1.510260	0.999684	0.153129
C	-2.760764	1.307833	0.650708
N	-3.507938	0.157106	0.630477
S	-5.184650	0.041381	1.145595
O	-5.347327	1.181231	2.039348
O	-5.328418	-1.347699	1.558940
C	-6.121695	0.316171	-0.342447
C	-6.476749	-0.776921	-1.137218
C	-7.222763	-0.551679	-2.290616
C	-7.624433	0.742113	-2.657355

C	-7.256000	1.816873	-1.833147
C	-6.509971	1.616629	-0.675291
C	-8.466061	0.968339	-3.888503
C	-0.418245	1.967839	-0.042082
S	1.118235	1.675392	1.094112
C	1.655483	3.371613	1.377393
C	1.685838	3.774354	2.718043
C	2.035057	5.091481	3.022789
C	2.348739	5.986741	1.999689
C	2.316249	5.573236	0.663888
C	1.965428	4.263531	0.340684
C	-0.307709	-1.178966	-0.629410
N	0.865734	-0.986798	0.264430
C	2.133549	-0.526560	-0.298243
S	0.958303	-1.989195	1.665107
O	-0.380987	-1.968047	2.242764
O	2.134588	-1.484175	2.381507
C	1.301427	-3.647616	1.097189
C	0.236437	-4.523304	0.863786

C	0.507327	-5.809415	0.401392
C	1.822699	-6.240325	0.175901
C	2.872126	-5.343316	0.431975
C	2.624679	-4.054149	0.894022
C	2.108398	-7.646621	-0.289021
C	2.360201	1.011539	-0.136786
C	3.817796	1.386437	0.233712
O	4.644079	0.778088	-0.806394
C	4.348028	0.897142	1.571048
C	4.707096	1.427095	-1.979754
O	4.133261	2.448700	-2.264248
C	5.584803	0.627227	-2.968704
F	5.784703	1.331756	-4.078127
F	4.937161	-0.514000	-3.281915
F	6.764004	0.313754	-2.419949
H	-3.144283	-1.895049	0.124439
H	-3.179217	2.234605	1.014300
H	-6.193280	-1.782336	-0.845109
H	-7.507953	-1.396520	-2.911657

H	-7.567651	2.824079	-2.096351
H	-6.251855	2.448196	-0.028408
H	-9.534195	0.915620	-3.640776
H	-8.281839	1.954516	-4.325659
H	-8.271419	0.210378	-4.653369
H	-0.714233	2.986503	0.214785
H	0.010631	1.964446	-1.049013
H	1.439407	3.073109	3.509988
H	2.061174	5.412088	4.059479
H	2.621660	7.009563	2.240724
H	2.566799	6.269964	-0.130034
H	1.959357	3.949732	-0.698801
H	0.011607	-0.849012	-1.625999
H	-0.549412	-2.244354	-0.726157
H	2.187880	-0.770808	-1.364064
H	2.956354	-1.046614	0.188883
H	-0.780780	-4.211067	1.074689
H	-0.317465	-6.494785	0.225692
H	3.899079	-5.664902	0.280096

H	3.446706	-3.385510	1.126040
H	2.987408	-7.683175	-0.940248
H	1.259198	-8.070711	-0.832998
H	2.310305	-8.303220	0.567148
H	2.125849	1.528639	-1.071065
H	3.939201	2.467202	0.148231
H	5.415455	1.125928	1.633898
H	4.203491	-0.173566	1.723570
H	3.843502	1.421483	2.388587

----

total energy -3293.22803404 a.u.

----

number of imaginary frequencies 0

----

molecule No.15d

----

optimized geometry

	x	y	z
C	3.002657	-0.116918	-0.471115
C	1.904447	-0.018176	0.347669
C	1.744819	1.377587	0.668141
C	2.759597	2.072643	0.036797
N	3.518002	1.155951	-0.644545
S	4.928756	1.567476	-1.625884
O	4.815459	3.013466	-1.775246
O	4.875976	0.628616	-2.736321
C	6.311564	1.175153	-0.579255
C	6.902337	-0.087339	-0.678074
C	8.000724	-0.375627	0.127335
C	8.516350	0.573225	1.023427
C	7.906125	1.836151	1.087459
C	6.807213	2.148312	0.293065
C	9.694145	0.241739	1.905551
C	0.677946	1.965391	1.496113
S	-0.962805	2.258678	0.539957

C	0.974801	-1.112932	0.786725
N	-0.154238	-1.387308	-0.138022
C	-1.696373	0.539386	0.241272
C	-0.796049	-0.232278	-0.763770
C	-1.996961	2.987854	1.812329
C	-2.254419	2.341112	3.029255
C	-3.031596	2.990306	3.986604
C	-3.538087	4.268987	3.732737
C	-3.272963	4.904931	2.519333
C	-2.499647	4.267269	1.548226
C	-3.155887	0.726562	-0.241522
S	0.117352	-2.731463	-1.196238
O	1.533695	-2.773641	-1.581847
O	-0.936688	-2.641196	-2.210134
C	-0.194162	-4.120340	-0.115598
C	0.842982	-5.013284	0.155149
C	0.584906	-6.124846	0.957473
C	-0.691980	-6.360445	1.482584
C	-1.716131	-5.444256	1.186837

C	-1.481382	-4.330056	0.389224
C	-0.973831	-7.583205	2.320562
O	-3.032585	1.290946	-1.566077
C	-3.909304	-0.593342	-0.235813
C	-3.994979	1.990495	-2.222736
O	-3.899674	2.212244	-3.393442
C	-5.167200	2.572105	-1.390286
F	-4.668484	3.391449	-0.426845
F	-5.870692	1.603302	-0.777734
F	-5.978160	3.273918	-2.164563
C	-4.014751	-1.395060	-1.377560
C	-4.719955	-2.599475	-1.320014
C	-5.317074	-3.010654	-0.127788
C	-5.211859	-2.212248	1.014825
C	-4.514432	-1.007291	0.958900
H	3.426221	-0.969561	-0.979517
H	3.000299	3.124740	0.000909
H	6.526805	-0.814490	-1.390044
H	8.474680	-1.350146	0.048280

H	8.306956	2.590437	1.759146
H	6.358769	3.135454	0.326210
H	9.355923	-0.153100	2.872634
H	10.302995	1.127601	2.110668
H	10.335929	-0.517718	1.449160
H	0.900950	2.980951	1.832370
H	0.392316	1.359227	2.359208
H	0.540858	-0.860714	1.763978
H	1.516234	-2.048267	0.937991
H	-1.718099	0.034795	1.209993
H	-1.421840	-0.606532	-1.571790
H	-0.067410	0.443453	-1.230874
H	-1.862015	1.350031	3.236973
H	-3.238748	2.498850	4.932178
H	-4.141404	4.768490	4.484503
H	-3.668800	5.896107	2.322177
H	-2.297960	4.753750	0.599168
H	-3.663466	1.430222	0.422038
H	1.828199	-4.841887	-0.264548

H	1.389378	-6.823409	1.171294
H	-2.714214	-5.612993	1.583131
H	-2.284354	-3.638160	0.157707
H	-1.675916	-7.361413	3.131135
H	-0.058447	-7.988842	2.761302
H	-1.424005	-8.376613	1.710036
H	-3.562667	-1.085408	-2.313655
H	-4.800142	-3.212524	-2.212359
H	-5.872070	-3.943520	-0.090394
H	-5.688114	-2.518109	1.941802
H	-4.459031	-0.376372	1.844242

---

total energy -3484.95217939 a.u.

---

number of imaginary frequencies 0

---

molecule No.21a

----

optimized geometry

	x	y	z
C	0.648543	-0.534888	-0.364794
C	0.015760	0.680710	-0.557583
C	-1.388945	0.434888	-0.671886
C	-1.570115	-0.927522	-0.528965
N	-0.335170	-1.517809	-0.347812
S	0.003256	-3.184932	0.116631
O	0.475561	-3.105074	1.500256
O	0.874174	-3.714506	-0.931423
C	-1.589262	-3.954434	0.045164
C	-2.008420	-4.535633	-1.156260
C	-3.249243	-5.164167	-1.193095
C	-4.067993	-5.228008	-0.053654
C	-3.612312	-4.638344	1.136834
C	-2.376133	-4.002756	1.200589
C	-5.393926	-5.944335	-0.098739

C	2.108983	-0.768405	-0.148481
C	2.621007	-0.123733	1.175193
C	1.840057	1.148730	1.577060
N	1.788413	2.092122	0.457523
C	0.699373	2.023880	-0.564643
C	-2.478550	1.440760	-0.861262
S	-2.779196	2.333670	0.749714
C	-3.995605	3.555006	0.243956
C	-5.356963	3.314946	0.473455
C	-6.304563	4.272238	0.108404
C	-5.899178	5.468181	-0.487162
C	-4.542367	5.711542	-0.712955
C	-3.590278	4.761086	-0.344426
S	3.243910	2.834796	0.154375
O	3.715534	3.482345	1.374258
O	3.174987	3.516761	-1.131619
C	4.283305	1.373916	-0.026760
C	5.104495	1.239107	-1.120551
C	5.896724	0.083960	-1.246488

C	5.865830	-0.994455	-0.322742
C	5.027348	-0.868019	0.762301
C	4.163650	0.304849	0.995738
C	6.738705	-2.204449	-0.541090
H	-2.474656	-1.514770	-0.555897
H	-1.370677	-4.504301	-2.033215
H	-3.585745	-5.620536	-2.119836
H	-4.232057	-4.684232	2.027975
H	-2.020272	-3.562289	2.125800
H	-6.103822	-5.521621	0.618494
H	-5.265994	-7.004888	0.154214
H	-5.841463	-5.899135	-1.096144
H	2.649925	-0.326139	-0.995344
H	2.352470	-1.831485	-0.159876
H	2.562496	-0.849854	1.993823
H	2.299728	1.638018	2.440222
H	0.818004	0.890629	1.854347
H	-0.030781	2.805134	-0.328349
H	1.143711	2.270542	-1.534730

H	-3.407807	0.954624	-1.169263
H	-2.218722	2.185041	-1.619680
H	-5.666598	2.385205	0.941141
H	-7.358790	4.083867	0.290884
H	-6.638381	6.212419	-0.769372
H	-4.224101	6.645546	-1.167435
H	-2.533300	4.956281	-0.501194
H	5.150373	2.024357	-1.868849
H	6.571182	0.014546	-2.097776
H	4.977564	-1.663439	1.503070
H	4.421855	0.728358	1.984318
H	6.623271	-2.930789	0.266928
H	7.795342	-1.919183	-0.594747
H	6.485412	-2.702748	-1.483713

---

total energy -2727.08525279 a.u.

---

number of imaginary frequencies 0

----

molecule No.21b

----

optimized geometry

	x	y	z
C	-1.293990	-0.981205	-0.886332
C	-1.143702	0.375289	-0.765213
C	-2.442440	0.988848	-0.756558
C	-3.345450	-0.037346	-0.885610
N	-2.658906	-1.244956	-0.934893
S	-3.392625	-2.767526	-1.426741
O	-2.461266	-3.781415	-0.933727
O	-3.716437	-2.653044	-2.843562
C	-4.890951	-2.787508	-0.473683
C	-6.099755	-2.483225	-1.104810
C	-7.274722	-2.532295	-0.357544
C	-7.259861	-2.886763	0.999224

C	-6.027097	-3.194087	1.600141
C	-4.841329	-3.152746	0.875755
C	-8.540141	-2.969494	1.791921
C	-2.757637	2.439194	-0.581813
S	-2.353485	2.953569	1.165785
C	-0.156500	-1.945640	-0.882633
C	1.048836	-1.428079	-0.093077
C	0.866109	-0.663052	1.165367
N	1.232695	0.114992	-0.058237
C	0.193775	1.028303	-0.675741
C	-2.488498	4.741473	1.041555
C	-3.734984	5.362823	1.197335
C	-3.835685	6.752374	1.121224
C	-2.694803	7.526873	0.900314
C	-1.450109	6.910871	0.756059
C	-1.344268	5.521022	0.825632
S	2.927257	1.023127	0.020653
O	3.644322	0.304816	1.063516
O	2.500441	2.407394	0.163337

C	3.667303	0.756572	-1.563172
C	3.314750	1.599077	-2.625967
C	3.937783	1.413190	-3.854814
C	4.915259	0.419941	-4.034544
C	5.260790	-0.388641	-2.938882
C	4.650243	-0.231661	-1.698986
C	5.608832	0.254881	-5.361688
C	2.276746	-2.294854	-0.267516
H	-4.424139	-0.013503	-0.924597
H	-6.116059	-2.228536	-2.159163
H	-8.219924	-2.299075	-0.839773
H	-5.999771	-3.476948	2.648964
H	-3.896085	-3.409846	1.341988
H	-8.395852	-2.629207	2.822368
H	-8.898105	-4.006083	1.839305
H	-9.333761	-2.368612	1.338658
H	-3.815131	2.633582	-0.777517
H	-2.168084	3.067584	-1.257064
H	0.174535	-2.189361	-1.902095

H	-0.441554	-2.898255	-0.427070
H	1.659457	-0.721280	1.906183
H	-0.129915	-0.440017	1.535359
H	0.160855	1.931366	-0.062642
H	0.589217	1.287353	-1.664538
H	-4.617216	4.758162	1.385415
H	-4.803883	7.230003	1.242278
H	-2.774817	8.608910	0.847416
H	-0.559939	7.512033	0.592896
H	-0.376293	5.038965	0.723006
H	2.594689	2.398029	-2.486685
H	3.671773	2.059723	-4.686124
H	6.029737	-1.147003	-3.055457
H	4.946271	-0.841339	-0.853038
H	6.501042	0.892953	-5.407638
H	4.959036	0.542403	-6.193421
H	5.937305	-0.776754	-5.517908
H	3.091346	-2.023317	0.400364
H	2.617689	-2.292460	-1.306801

H	1.981170	-3.320511	-0.020632
F	5.357262	-2.340948	1.206254
C	5.789242	-1.849252	2.384653
F	6.762742	-2.642867	2.839984
F	6.286256	-0.623593	2.172329
C	4.614985	-1.781719	3.389054
O	3.463250	-1.982589	3.090100
O	5.073955	-1.461921	4.597698
H	4.314683	-1.414190	5.210808

----

total energy   -3293.23088908   a.u.

----

number of imaginary frequencies   0

----

molecule No.21c

----

optimized geometry

	x	y	z
C	0.252470	-1.496150	-0.486708
C	-0.357179	-0.245979	-0.476966
C	0.493142	0.672216	0.236733
C	1.599941	-0.030034	0.626635
N	1.478352	-1.333524	0.186209
S	2.781601	-2.519239	0.386127
O	2.768022	-3.297042	-0.852029
O	2.585824	-3.155302	1.685866
C	4.238103	-1.504408	0.440978
C	4.843646	-1.255559	1.675582
C	6.016033	-0.503661	1.703633
C	6.591387	-0.007875	0.524150
C	5.958810	-0.283688	-0.700201
C	4.788964	-1.032703	-0.755325
C	7.878920	0.776081	0.561356
C	0.264839	2.133798	0.457860
C	-0.146769	-2.854576	-1.043314

C	-1.381029	-2.847694	-1.944917
C	-1.602179	-1.602586	-2.827622
N	-2.382938	-0.647584	-2.002483
C	-1.599283	0.282958	-1.154885
C	-0.309785	-3.958709	0.030567
S	-3.541575	-1.628207	-1.292612
O	-2.630754	-2.939620	-1.110229
O	-4.689449	-1.830966	-2.157742
C	-4.020578	-1.126901	0.321588
C	-3.151924	-1.248039	1.417123
C	-3.621751	-0.873268	2.668069
C	-4.930693	-0.386513	2.846791
C	-5.770341	-0.285460	1.726598
C	-5.332921	-0.653852	0.458827
C	-5.409575	0.023484	4.214200
S	0.677266	3.083747	-1.096426
C	0.099218	4.727265	-0.654178
C	0.924572	5.585124	0.085833
C	0.484722	6.868810	0.410199

C	-0.772858	7.306399	-0.011213
C	-1.592332	6.458362	-0.758595
C	-1.160152	5.170582	-1.079331
H	2.474782	0.299963	1.164864
H	4.411146	-1.654544	2.586817
H	6.496892	-0.306779	2.657752
H	6.395336	0.087890	-1.623205
H	4.317457	-1.257889	-1.705964
H	7.884630	1.572581	-0.189541
H	8.734077	0.122008	0.347362
H	8.048082	1.226487	1.543639
H	0.895860	2.502751	1.270471
H	-0.775895	2.354483	0.716872
H	0.664562	-3.178491	-1.708519
H	-1.407981	-3.766190	-2.531992
H	-2.176798	-1.845374	-3.724876
H	-0.664092	-1.127929	-3.118115
H	-1.311833	1.102039	-1.824681
H	-2.293375	0.727322	-0.434185

H	-0.554856	-4.911898	-0.450515
H	-1.114856	-3.717522	0.729320
H	0.603509	-4.105422	0.604946
H	-2.141887	-1.621710	1.295931
H	-2.964615	-0.969944	3.527550
H	-6.786517	0.077197	1.848123
H	-5.992914	-0.594824	-0.399524
H	-6.496424	0.134192	4.245344
H	-4.967033	0.985458	4.503222
H	-5.114086	-0.708261	4.973287
H	1.909952	5.248838	0.394792
H	1.128354	7.530459	0.983060
H	-1.109852	8.309154	0.235525
H	-2.566739	6.800218	-1.096385
H	-1.791107	4.511598	-1.668616

----

total energy -2766.42701572 a.u.

----

number of imaginary frequencies 0

----

molecule No.21d

----

optimized geometry

	x	y	z
C	-0.060996	0.209517	-1.021328
C	1.177243	0.017652	-1.740249
C	1.620030	-1.281110	-1.547895
C	0.682820	-1.874462	-0.671284
N	-0.282015	-1.013097	-0.321382
S	-1.465442	-1.379535	1.019824
O	-2.393839	-2.353375	0.472056
O	-1.873476	-0.051665	1.457723
C	-0.394458	-2.142729	2.206672
C	-0.640948	-3.471466	2.563272
C	0.165979	-4.056064	3.537091

C	1.204952	-3.339276	4.147309
C	1.424898	-2.005724	3.756539
C	0.631866	-1.391976	2.795613
C	2.064762	-3.972556	5.211131
C	2.875735	-1.919851	-2.063748
S	3.598110	-3.060848	-0.792580
C	-1.248909	1.022938	-1.829965
C	-0.531306	1.615106	-0.682540
C	0.510014	2.740642	-0.931851
N	1.859372	2.292718	-1.290915
C	1.868170	1.219473	-2.312185
C	5.322499	-3.069222	-1.295375
C	5.866804	-4.218490	-1.880134
C	7.219237	-4.244491	-2.226845
C	8.021816	-3.125210	-2.001737
C	7.475471	-1.978086	-1.419768
C	6.129825	-1.948278	-1.054409
S	2.891470	1.965474	0.064311
O	4.015462	1.210253	-0.501814

O	2.095015	1.401314	1.167667
C	3.416276	3.601525	0.533303
C	4.250542	4.326188	-0.325670
C	4.683700	5.586281	0.067203
C	4.305433	6.135144	1.305720
C	3.474329	5.381745	2.144440
C	3.026988	4.114313	1.771051
C	4.804717	7.496466	1.721264
C	-2.655295	0.559551	-1.863713
C	-3.610115	0.983005	-0.925950
C	-4.937398	0.573377	-1.041051
C	-5.322278	-0.265238	-2.089251
C	-4.380607	-0.686277	-3.032657
C	-3.057414	-0.271745	-2.924643
H	0.705305	-2.875059	-0.257545
H	-1.446120	-4.026663	2.094471
H	-0.016534	-5.087076	3.825941
H	2.233460	-1.440927	4.211965
H	0.824720	-0.366529	2.495508

H	3.124712	-3.749098	5.049754
H	1.942106	-5.058698	5.235622
H	1.799828	-3.584715	6.202991
H	3.617352	-1.146566	-2.276603
H	2.687697	-2.476260	-2.988509
H	-0.850515	1.274246	-2.811483
H	-1.080942	1.677283	0.250525
H	0.579172	3.366549	-0.040240
H	0.151541	3.377977	-1.748154
H	1.358165	1.621857	-3.197343
H	2.897145	0.997883	-2.592950
H	5.235406	-5.083192	-2.059627
H	7.641865	-5.137796	-2.677870
H	9.072830	-3.147038	-2.275448
H	8.101427	-1.109171	-1.236623
H	5.704873	-1.064877	-0.585569
H	4.555340	3.908628	-1.279495
H	5.330398	6.156227	-0.594683
H	3.174913	5.787721	3.106687

H	2.395074	3.527674	2.428825
H	4.272942	7.869563	2.600887
H	4.685948	8.227376	0.913891
H	5.873216	7.460058	1.968252
H	-3.324169	1.640967	-0.112844
H	-5.673352	0.914244	-0.319899
H	-6.356897	-0.581752	-2.173046
H	-4.679718	-1.331928	-3.852978
H	-2.326490	-0.593114	-3.663289
F	-7.714512	-0.812355	0.264606
C	-8.869405	-0.267841	-0.141528
F	-8.904352	-0.315940	-1.490035
F	-9.883403	-0.989897	0.339277
C	-8.933318	1.208429	0.310050
O	-7.947252	1.875099	0.500217
O	-10.198894	1.621217	0.406825
H	-10.184097	2.566833	0.652782

----

total energy -3484.96161361 a.u.

----

number of imaginary frequencies 0

----

molecule No.21ba

----

optimized geometry

	x	y	z
C	-0.628375	-1.303212	0.201563
C	0.074326	-0.115131	0.112924
C	-0.852853	0.941042	-0.177197
C	-2.096863	0.363763	-0.255847
N	-1.967159	-0.998445	-0.046796
S	-3.302517	-2.123961	0.196191
O	-3.474027	-2.250995	1.643290
O	-2.970992	-3.290392	-0.621732
C	-4.683886	-1.261268	-0.505005

C	-4.929023	-1.370835	-1.877663
C	-6.040138	-0.724041	-2.408961
C	-6.910284	0.018513	-1.593654
C	-6.636971	0.102100	-0.219175
C	-5.531639	-0.535483	0.337352
C	-8.131781	0.682336	-2.177723
C	-0.552494	2.398024	-0.331989
S	-0.198755	3.147582	1.340725
C	-0.103733	-2.678415	0.522620
C	0.563807	-2.785083	1.921506
C	1.399074	-1.537453	2.239547
N	2.131245	-1.096696	1.019121
C	1.556489	0.079271	0.300100
C	0.298104	4.809697	0.870227
C	-0.672350	5.804613	0.689107
C	-0.287447	7.101206	0.346364
C	1.064940	7.413456	0.192307
C	2.034686	6.427132	0.382587
C	1.654487	5.127546	0.720454

S	3.763345	-1.330091	1.032036
O	3.870014	-2.864194	1.373473
O	4.557093	-0.516411	1.940282
C	4.321159	-1.155010	-0.631960
C	5.265210	-0.158855	-0.898895
C	5.741373	-0.029890	-2.201041
C	5.297702	-0.876795	-3.227420
C	4.349151	-1.869728	-2.919245
C	3.858277	-2.023149	-1.629555
C	5.844646	-0.750872	-4.625578
C	-0.422514	-3.081050	3.046477
H	-3.057405	0.809506	-0.460898
H	-4.270932	-1.959750	-2.507617
H	-6.242218	-0.803251	-3.473587
H	-7.304029	0.667205	0.425878
H	-5.332295	-0.484368	1.402402
H	-8.426500	1.562814	-1.598995
H	-8.983389	-0.010378	-2.175853
H	-7.965006	0.989955	-3.214551

H	-1.401454	2.922284	-0.778093
H	0.321020	2.571595	-0.968745
H	0.654843	-2.943372	-0.223253
H	-0.892849	-3.428169	0.437260
H	2.090847	-1.751139	3.060074
H	0.759282	-0.716392	2.564471
H	1.739302	1.000197	0.869174
H	2.074603	0.164301	-0.661005
H	-1.721916	5.562242	0.827149
H	-1.043358	7.869187	0.208447
H	1.362716	8.425205	-0.067866
H	3.087974	6.670298	0.273127
H	2.406474	4.360216	0.879930
H	5.621115	0.488675	-0.105008
H	6.475376	0.739825	-2.420538
H	3.999048	-2.536295	-3.702256
H	3.139660	-2.803890	-1.403488
H	5.074169	-0.951086	-5.376697
H	6.651967	-1.477047	-4.786532

H	6.256951	0.245134	-4.808693
H	-1.225859	-2.338943	3.061148
H	0.082895	-3.087993	4.017420
H	-0.890217	-4.059731	2.895416
O	1.569771	-3.852250	1.874323
H	2.955694	-3.341347	1.506365
H	1.188358	-4.666605	1.505466

----

total energy -2842.87569632 a.u.

----

number of imaginary frequencies 0

----

molecule No.22b

----

optimized geometry

x y z

C	-0.986078	-0.062856	-0.020549
C	-0.493531	-0.328986	-1.256570
C	-1.628637	-0.554206	-2.151038
C	-2.813721	-0.450665	-1.324591
N	-2.431158	-0.166900	-0.103429
S	-3.563281	0.041196	1.362602
O	-3.496237	1.467927	1.631071
O	-3.074455	-0.973439	2.279404
C	-5.130498	-0.407804	0.688127
C	-5.530761	-1.749883	0.738565
C	-6.788584	-2.082322	0.248206
C	-7.649299	-1.103692	-0.279368
C	-7.214150	0.232444	-0.310286
C	-5.961466	0.595785	0.173019
C	-9.025096	-1.475558	-0.767757
C	-1.655986	-0.743807	-3.488442
S	0.395461	2.715293	0.183980
C	-0.294845	0.292402	1.264158
C	0.979258	1.164588	1.117991

C	2.166966	0.445996	0.403884
N	1.856970	-0.637924	-0.528220
C	0.960208	-0.386426	-1.647990
S	1.976640	-2.242735	0.034913
O	1.368755	-3.055119	-1.025209
O	1.478062	-2.314112	1.417053
C	3.736922	-2.542661	0.089621
C	4.364342	-2.718191	1.322737
C	5.734760	-2.979289	1.352419
C	6.481763	-3.075715	0.172154
C	5.818922	-2.900883	-1.055365
C	4.455216	-2.638969	-1.107040
C	7.958195	-3.384985	0.206843
C	1.443070	1.573715	2.524269
C	1.851815	3.757553	0.054059
C	2.601021	3.766647	-1.132979
C	3.699359	4.618704	-1.259993
C	4.053225	5.467023	-0.209429
C	3.299204	5.473597	0.966279

C	2.197066	4.628557	1.098401
H	-3.851722	-0.570550	-1.604471
H	-4.879195	-2.504894	1.166009
H	-7.114694	-3.117787	0.285619
H	-7.871938	1.000386	-0.707134
H	-5.638785	1.631675	0.170518
H	-9.046156	-2.489878	-1.177213
H	-9.381623	-0.782465	-1.535193
H	-9.743853	-1.443830	0.061475
H	-2.592610	-0.869346	-4.024307
H	-0.744714	-0.761314	-4.077215
H	-0.012795	-0.631510	1.785597
H	-0.986873	0.832556	1.914668
H	2.814906	0.020031	1.174663
H	2.771143	1.192596	-0.122811
H	1.253054	0.556749	-2.121989
H	1.100192	-1.178317	-2.387462
H	3.785212	-2.662662	2.237894
H	6.227757	-3.116933	2.310954

H	6.381739	-2.977557	-1.982152
H	3.952602	-2.520248	-2.061431
H	8.145780	-4.423977	-0.092683
H	8.374140	-3.249837	1.209305
H	8.516816	-2.744622	-0.484502
H	0.676731	2.158036	3.041648
H	2.357652	2.172456	2.478040
H	1.656335	0.675728	3.117119
H	2.307958	3.124592	-1.958872
H	4.274774	4.621978	-2.181441
H	4.907305	6.130341	-0.310570
H	3.562070	6.145023	1.778643
H	1.595845	4.651266	2.001553
O	0.023790	2.185081	-3.208013
H	-0.027873	2.480834	-2.279309
H	0.137729	3.001786	-3.717772

----

total energy -2842.85305662 a.u.

----

number of imaginary frequencies 0

----

molecule No.23b

----

optimized geometry

	x	y	z
C	1.644253	-0.656925	-0.188763
C	1.001445	-1.809715	-0.579725
C	1.574566	-2.917106	0.130865
C	2.578832	-2.402729	0.919727
N	2.620780	-1.033588	0.731339
S	3.816247	-0.028804	1.559223
O	4.391160	-0.944362	2.536515
O	3.108800	1.189347	1.937586
C	5.014619	0.346806	0.299585
C	5.940951	-0.632665	-0.074224

C	6.890675	-0.320621	-1.040230
C	6.940045	0.954131	-1.629714
C	6.003827	1.915652	-1.223977
C	5.040057	1.625902	-0.260193
C	8.000397	1.286631	-2.649335
C	1.105373	-4.330581	0.122003
C	1.262746	0.741086	-0.586346
C	-0.178879	1.133704	-0.133832
C	-1.305576	0.409530	-0.928957
N	-1.360806	-1.086857	-0.936487
C	-0.171238	-1.787707	-1.506212
S	-2.295403	-1.885352	0.157823
O	-2.017127	-1.691487	1.580100
O	-2.137607	-3.380129	-0.286455
C	-3.964451	-1.458743	-0.221674
C	-4.412555	-1.471039	-1.548704
C	-5.749443	-1.187801	-1.796723
C	-6.641349	-0.894649	-0.749111
C	-6.153747	-0.885570	0.566310

C	-4.820215	-1.170855	0.845916
C	-8.094455	-0.618706	-1.037220
S	-0.281609	2.937692	-0.686692
C	-0.327905	1.012113	1.383054
C	-1.833741	3.545834	-0.017670
C	-2.985688	3.567407	-0.818705
C	-4.171172	4.115859	-0.326538
C	-4.213841	4.649484	0.962949
C	-3.066306	4.642470	1.759010
C	-1.877132	4.099905	1.270852
H	3.263848	-2.878398	1.605139
H	5.927796	-1.612106	0.392240
H	7.614480	-1.075163	-1.336170
H	6.033598	2.910066	-1.660602
H	4.335989	2.382726	0.067883
H	8.205504	0.435200	-3.306227
H	8.943971	1.548307	-2.153323
H	7.708744	2.138487	-3.270365
H	1.821362	-5.001468	0.603396

H	0.901410	-4.713562	-0.880547
H	1.339325	0.842576	-1.678104
H	1.951407	1.466430	-0.152927
H	-1.222463	0.680665	-1.985980
H	-2.275919	0.775831	-0.582260
H	-0.479293	-2.783879	-1.823668
H	0.052708	-1.223040	-2.418279
H	-3.732161	-1.693074	-2.363739
H	-6.110687	-1.193054	-2.821213
H	-6.826899	-0.652653	1.386124
H	-4.447135	-1.165307	1.864078
H	-8.565381	-0.052988	-0.228570
H	-8.647871	-1.560399	-1.146802
H	-8.219042	-0.059730	-1.969932
H	-1.302420	1.380269	1.716252
H	0.453476	1.587131	1.886239
H	-0.239714	-0.029369	1.704079
H	-2.942757	3.176354	-1.830948
H	-5.056616	4.138234	-0.955857

H	-5.134833	5.083578	1.341658
H	-3.091871	5.071722	2.756547
H	-0.978027	4.115252	1.878473
O	-0.183880	-4.493133	0.821073
H	-1.316497	-3.861548	0.217810
H	-0.082958	-4.159052	1.730916

----

total energy -2842.87912061 a.u.

----

number of imaginary frequencies 0

----

molecule No.14aa

----

optimized geometry

	x	y	z
C	-7.455061	-1.695813	-1.697723

C	-8.439767	-2.367584	-2.621759
C	-6.860515	-2.407054	-0.643847
C	-5.977468	-1.789491	0.237117
C	-5.681308	-0.436235	0.052235
C	-6.259794	0.303185	-0.983037
C	-7.140013	-0.336440	-1.850720
S	-4.568814	0.370609	1.180401
O	-4.475439	-0.404199	2.410459
O	-4.789033	1.811452	1.140707
N	-2.982289	0.181237	0.443012
C	-2.147638	-0.909819	0.603962
C	-0.984953	-0.671560	-0.086913
C	-1.128377	0.628239	-0.700688
C	-2.373753	1.117378	-0.355250
C	-0.121401	1.312162	-1.554955
S	0.445528	2.920789	-0.780104
C	1.812980	3.496173	-1.734723
C	1.819388	4.882393	-1.972945
C	2.845165	5.420588	-2.744671

C	3.848414	4.587247	-3.247035
C	3.836924	3.212087	-2.985050
C	2.817665	2.645095	-2.225801
O	1.126487	2.231452	0.627456
C	1.378562	3.156548	1.615151
O	1.243623	4.337615	1.497086
C	1.850818	2.427505	2.898945
F	0.847821	1.661516	3.359184
F	2.176256	3.327786	3.816964
F	2.905457	1.653182	2.629116
C	0.157611	-1.645031	-0.218261
N	1.373902	-1.189962	0.484813
S	2.822296	-1.096933	-0.357885
O	3.739565	-0.322270	0.480305
O	2.444480	-0.600862	-1.702096
C	3.521518	-2.728063	-0.584621
C	1.398105	-1.359625	1.957133
C	1.244630	-2.785692	2.420907
C	0.260544	-3.202326	3.219082

C	3.125939	-3.514407	-1.671988
C	3.657722	-4.792695	-1.809918
C	4.580046	-5.303467	-0.881830
C	4.966121	-4.487674	0.190587
C	4.446389	-3.203620	0.347821
C	5.143336	-6.693317	-1.048449
H	-9.460040	-2.282338	-2.225726
H	-8.436553	-1.908567	-3.615069
H	-8.222257	-3.434136	-2.733915
H	-7.100745	-3.457579	-0.504699
H	-5.542623	-2.338300	1.065501
H	-6.041344	1.360141	-1.091087
H	-7.599302	0.231739	-2.655053
H	-2.450288	-1.753819	1.205332
H	-2.868848	2.051503	-0.578042
H	-0.514768	1.682850	-2.509359
H	0.770958	0.705833	-1.735013
H	1.045163	5.520111	-1.557481
H	2.866000	6.487079	-2.943867

H	4.650484	5.012827	-3.842696
H	4.627407	2.576677	-3.371365
H	2.819061	1.579669	-2.014570
H	0.406585	-1.788568	-1.271613
H	-0.151461	-2.617570	0.181930
H	0.588862	-0.741399	2.359813
H	2.335572	-0.922938	2.309616
H	2.004987	-3.489654	2.084032
H	0.197738	-4.231242	3.561886
H	-0.508389	-2.522454	3.582201
H	2.438563	-3.119809	-2.412478
H	3.360403	-5.402549	-2.659278
H	5.693242	-4.856793	0.908670
H	4.771735	-2.569405	1.165572
H	5.613051	-6.814242	-2.031487
H	4.352900	-7.450152	-0.974535
H	5.894234	-6.917239	-0.285843

----

total energy -3253.87273581 a.u.

----

number of imaginary frequencies 0

----

molecule No.14ab

----

optimized geometry

	x	y	z
C	-2.963445	-0.070114	-1.167951
C	-1.804242	0.217345	-0.495012
C	-1.569223	-0.872972	0.421707
C	-2.590898	-1.782822	0.253790
N	-3.439738	-1.289755	-0.710415
C	-0.414403	-1.098445	1.319368
C	-0.995325	1.466213	-0.701821
N	0.345740	1.226812	-1.298282
S	1.016734	-1.812895	0.326954

C	2.087928	-0.421355	-0.345790
C	1.427828	0.969763	-0.339429
S	0.753264	2.254526	-2.604797
S	-4.966707	-1.995838	-1.143700
O	-4.840379	-3.398548	-0.764110
O	-5.212296	-1.545934	-2.508132
C	-6.135463	-1.200804	-0.053400
C	-6.781015	-0.036924	-0.475695
C	-7.687357	0.578101	0.384364
C	-7.960970	0.047284	1.653451
C	-7.300228	-1.127025	2.044826
C	-6.390726	-1.758808	1.201264
C	-8.968494	0.704672	2.564741
O	-0.418348	2.277898	-3.481036
O	2.064533	1.783940	-3.063499
C	0.971969	3.897417	-1.920539
C	2.212996	4.271418	-1.393084
C	2.346696	5.517177	-0.787793
C	1.265109	6.407125	-0.709285

C	0.041857	6.020695	-1.273416
C	-0.115617	4.773038	-1.874332
C	1.420609	7.742616	-0.023736
C	2.020605	-2.726050	1.503645
C	2.607869	-0.871239	-1.702043
C	2.377070	-4.025468	1.135814
C	3.152537	-4.767689	2.024890
C	3.546217	-4.211708	3.244354
C	3.172820	-2.909525	3.585931
C	2.402097	-2.139669	2.714057
O	3.458597	-2.043578	-1.498976
C	3.455142	-2.922133	-2.453097
O	0.525009	-2.703991	-0.749483
C	4.373851	-4.066361	-1.974916
F	5.271700	-4.333759	-2.937121
F	3.581661	-5.155064	-1.807462
F	5.050352	-3.886270	-0.819971
H	-3.477113	0.464759	-1.952364
H	-2.773174	-2.742525	0.713205

H	-0.603738	-1.895937	2.043160
H	0.009589	-0.234673	1.848796
H	-0.873718	2.009926	0.245560
H	-1.551386	2.111367	-1.382621
H	2.887377	-0.344380	0.408246
H	1.078786	1.183372	0.671941
H	2.266325	1.653463	-0.496741
H	-6.587473	0.366552	-1.463786
H	-8.195045	1.483371	0.061889
H	-7.504941	-1.555964	3.022289
H	-5.897750	-2.677314	1.501175
H	-8.712299	0.556706	3.618626
H	-9.969100	0.280276	2.409429
H	-9.037619	1.780584	2.376590
H	3.066893	3.608731	-1.479139
H	3.308647	5.802556	-0.370684
H	-0.800465	6.707243	-1.245454
H	-1.059421	4.490723	-2.328370
H	0.585249	8.411623	-0.251433

H	2.348670	8.240688	-0.325339
H	1.460889	7.617635	1.065849
H	3.232574	-0.078556	-2.116104
H	1.828468	-1.132567	-2.417275
H	2.068916	-4.435543	0.180967
H	3.452742	-5.775817	1.756768
H	4.152610	-4.795680	3.930961
H	3.487447	-2.480191	4.532186
H	2.116280	-1.117500	2.959218
O	1.465138	0.891685	2.815941
C	2.601007	1.392346	2.580812
O	3.547241	0.933413	1.912083
C	2.799031	2.842661	3.110033
F	4.081454	3.117869	3.411322
F	2.058281	3.114334	4.201327
F	2.417235	3.722150	2.138070

----

total energy -3780.14046264 a.u.

----

number of imaginary frequencies 0

----

molecule No.14ac

----

optimized geometry

	x	y	z
C	-0.732669	-1.551781	-2.007363
C	0.295673	-0.651593	-1.873843
C	-0.228372	0.638322	-2.240175
C	-1.558856	0.469145	-2.577298
N	-1.851753	-0.862604	-2.435849
S	-3.423501	-1.592099	-2.791120
O	-3.088079	-2.954789	-3.178156
O	-4.051617	-0.621364	-3.678221
C	-4.256684	-1.598708	-1.221281
C	-4.069658	-2.677882	-0.353032

C	-4.738827	-2.676578	0.867236
C	-5.590920	-1.621840	1.230113
C	-5.770119	-0.563000	0.325867
C	-5.109531	-0.539280	-0.898617
C	-6.283592	-1.615090	2.569357
C	1.700394	-0.969825	-1.451220
C	0.520151	1.914192	-2.257881
S	0.389774	2.787806	-0.587572
N	1.996658	-0.588705	-0.054664
C	2.406920	0.770355	0.251308
C	1.273944	1.724604	0.713781
C	0.201197	1.028760	1.546716
O	-0.600117	2.087676	2.112960
C	-1.821358	1.915358	2.667451
O	-2.439786	2.845386	3.099058
C	-2.403737	0.478267	2.698325
F	-1.543070	-0.383521	3.268650
F	-3.545562	0.462048	3.377080
F	-2.645394	0.057452	1.434343

C	1.399502	4.263894	-0.726221
C	0.806197	5.448215	-0.271320
C	1.528788	6.639417	-0.350333
C	2.818702	6.642040	-0.883072
C	3.398502	5.454119	-1.339161
C	2.693337	4.254478	-1.263411
S	2.381671	-1.831719	1.055174
O	1.459042	-2.919375	0.734595
O	2.389560	-1.155445	2.358613
C	4.044827	-2.384195	0.709464
C	4.246848	-3.435575	-0.190080
C	5.548318	-3.842903	-0.471831
C	6.652779	-3.226196	0.135313
C	6.417771	-2.182439	1.043487
C	5.125023	-1.758030	1.339242
C	8.055536	-3.703435	-0.147755
H	-0.759843	-2.621110	-1.861561
H	-2.301819	1.163344	-2.941770
H	-3.435461	-3.510700	-0.637429

H	-4.606743	-3.515529	1.544877
H	-6.445505	0.249304	0.580500
H	-5.270712	0.268430	-1.604442
H	-7.260708	-1.125489	2.513986
H	-5.681908	-1.064685	3.303523
H	-6.427597	-2.629129	2.953618
H	2.431021	-0.476944	-2.109266
H	1.863932	-2.043359	-1.550069
H	0.091935	2.661099	-2.931781
H	1.581194	1.798921	-2.488199
H	2.958660	1.201960	-0.593224
H	3.102292	0.742356	1.094572
H	1.717853	2.521794	1.318355
H	0.693789	0.457365	2.337699
H	-0.398967	0.348468	0.942104
H	-0.198173	5.439020	0.140982
H	1.079276	7.562275	0.002199
H	3.375531	7.571871	-0.946317
H	4.400872	5.460901	-1.755877

H	3.151242	3.338616	-1.623000
H	3.399458	-3.946098	-0.634990
H	5.708443	-4.664157	-1.165281
H	7.259410	-1.704806	1.538095
H	4.953976	-0.978069	2.073539
H	8.787394	-2.900111	-0.019420
H	8.148886	-4.095165	-1.165325
H	8.334527	-4.512432	0.539718

----

total energy -3253.90394330 a.u.

----

number of imaginary frequencies 0

----

molecule No.15aa

----

optimized geometry

	x	y	z
C	3.156932	0.995701	-0.101517
C	1.952851	0.340924	-0.087539
C	2.061229	-0.733743	0.868198
C	3.329334	-0.689898	1.401076
N	3.994413	0.368716	0.810707
S	5.681532	0.702516	1.011760
O	6.012348	0.142810	2.318839
O	5.829560	2.115076	0.682091
C	6.472697	-0.274811	-0.255559
C	6.686581	0.285651	-1.516518
C	7.297874	-0.487324	-2.500222
C	7.696611	-1.807713	-2.244768
C	7.480691	-2.335986	-0.963254
C	6.871149	-1.581197	0.035356
C	8.327798	-2.647005	-3.328626
C	1.043801	-1.768530	1.163233
C	0.708376	0.725108	-0.837868
N	-0.229489	1.479683	0.044506

S	-0.518614	-1.214620	2.067066
C	-1.614872	-0.551461	0.707557
C	-1.559074	0.940468	0.372747
C	-3.045484	-0.980891	1.065453
O	-3.795936	-0.868323	-0.167104
C	-4.976440	-1.503380	-0.187999
O	-5.500062	-2.074198	0.730480
C	-5.630866	-1.331368	-1.576064
F	-6.665316	-2.162648	-1.704050
F	-4.762771	-1.554194	-2.569816
F	-6.084770	-0.062042	-1.691315
C	0.039317	0.238609	3.082714
C	-1.023928	0.650732	4.072595
C	-1.559525	1.945193	4.022814
C	-2.520648	2.343057	4.955226
C	-2.958497	1.455217	5.937804
C	-2.427174	0.163963	5.993098
C	-1.464135	-0.234884	5.068506
S	-0.160167	3.172999	-0.060216

O	1.233032	3.504622	-0.362114
O	-0.832313	3.673743	1.145020
C	-1.155533	3.651666	-1.472751
C	-2.530683	3.852176	-1.319954
C	-3.297716	4.179612	-2.435511
C	-2.715919	4.312087	-3.704792
C	-1.331476	4.125626	-3.825807
C	-0.547112	3.796307	-2.722664
C	-3.562562	4.633988	-4.912109
H	3.471492	1.889150	-0.617480
H	3.822055	-1.313084	2.132056
H	6.398460	1.312778	-1.712885
H	7.474527	-0.055306	-3.481759
H	7.800136	-3.350969	-0.741840
H	6.724985	-1.987579	1.030335
H	7.562040	-3.202377	-3.885998
H	9.024517	-3.381599	-2.912627
H	8.871694	-2.028562	-4.049517
H	1.424858	-2.556962	1.816981

H	0.614202	-2.232263	0.264403
H	0.192576	-0.164065	-1.212710
H	0.964829	1.328078	-1.712290
H	-1.294265	-1.161737	-0.144109
H	-2.262273	1.054593	-0.466972
H	-1.945714	1.528246	1.207831
H	-3.503781	-0.335323	1.822510
H	-3.059784	-2.019987	1.393465
H	0.334673	1.047845	2.414296
H	0.938346	-0.145541	3.577812
H	-1.217254	2.645372	3.265213
H	-2.923973	3.350813	4.910356
H	-3.708054	1.766208	6.660168
H	-2.760716	-0.531813	6.757757
H	-1.053262	-1.240564	5.120668
H	-2.984615	3.782963	-0.337234
H	-4.365840	4.342480	-2.316673
H	-0.858244	4.248004	-4.796718
H	0.527364	3.684183	-2.818587

H	-2.982227	5.150262	-5.683241
H	-4.416687	5.265070	-4.646257
H	-3.963225	3.716438	-5.362668
O	-1.439719	-3.515431	0.664871
C	-1.349639	-3.471505	-0.585954
O	-0.645670	-2.712370	-1.297872
C	-2.321259	-4.418224	-1.347018
F	-3.566535	-3.855175	-1.373697
F	-2.443398	-5.617138	-0.748447
F	-1.958480	-4.632610	-2.622844

----

total energy -3819.62022273 a.u.

----

number of imaginary frequencies 0

----

molecule No.16a

----

optimized geometry

	x	y	z
C	-0.881912	-1.830166	-1.533379
C	-0.956832	-0.515135	-1.159138
C	0.353145	0.059073	-1.365963
C	1.172055	-0.935676	-1.856296
N	0.414723	-2.080496	-1.968027
S	1.087164	-3.664438	-2.197276
O	2.229059	-3.468381	-3.084746
O	-0.052860	-4.507967	-2.535003
C	1.682917	-4.078714	-0.566197
C	3.043983	-3.958146	-0.286148
C	3.488595	-4.219525	1.008948
C	2.594126	-4.585592	2.022859
C	1.234613	-4.722826	1.700872
C	0.769138	-4.473622	0.414665
C	3.063934	-4.786607	3.442333
C	0.775937	1.457793	-1.095341

C	-2.198840	0.226654	-0.727668
N	-3.314038	-0.663019	-0.379986
S	-4.714611	-0.604362	-1.308376
O	-5.456141	-1.827132	-0.988809
O	-4.294014	-0.274794	-2.672456
C	-5.700432	0.773754	-0.706123
C	-5.493084	2.053109	-1.229579
C	-6.234799	3.121943	-0.732507
C	-7.189227	2.937171	0.278754
C	-7.386406	1.642925	0.778498
C	-6.654573	0.560762	0.291916
C	-8.012020	4.097369	0.786050
C	-3.438171	-1.139581	1.019704
C	-3.217140	-2.622298	1.179117
C	-2.353908	-3.147695	2.049981
S	1.970768	1.468808	0.373945
C	2.085542	3.157627	1.028472
C	3.347020	3.590695	1.447449
C	3.471540	4.859890	2.011968

C	2.352529	5.681431	2.152518
C	1.098796	5.231460	1.735083
C	0.954621	3.962405	1.175926
O	0.369418	1.192408	1.462843
C	0.222857	0.213169	2.328856
O	-0.815246	-0.011257	2.915199
C	1.452433	-0.696504	2.603721
F	1.174774	-1.602316	3.546669
F	2.509849	0.035446	3.022969
F	1.822543	-1.356567	1.486491
H	-1.636332	-2.599067	-1.566090
H	2.215794	-0.928062	-2.133832
H	3.736447	-3.669388	-1.069422
H	4.547384	-4.126489	1.235996
H	0.529317	-5.020298	2.472453
H	-0.284486	-4.574854	0.178613
H	2.594744	-5.663316	3.901935
H	2.795103	-3.915340	4.053581
H	4.149605	-4.910959	3.495971

H	-0.051639	2.100092	-0.797077
H	1.330461	1.940583	-1.897076
H	-2.524040	0.877194	-1.545674
H	-1.972545	0.870301	0.132344
H	-4.780862	2.199912	-2.034699
H	-6.077898	4.116155	-1.144219
H	-8.130201	1.476226	1.553859
H	-6.836208	-0.443673	0.658684
H	-8.336927	3.937304	1.819130
H	-8.915481	4.234065	0.177133
H	-7.449415	5.035974	0.746678
H	-4.436693	-0.878404	1.395019
H	-2.720150	-0.580858	1.625823
H	-3.849256	-3.257953	0.562115
H	-2.266130	-4.224526	2.176228
H	-1.717564	-2.524746	2.674974
H	4.221012	2.963526	1.313578
H	4.448744	5.203539	2.337505
H	2.456266	6.670446	2.589115

H	0.224549	5.865790	1.846771
H	-0.026240	3.604591	0.889355
O	3.548122	1.679369	-0.736961
C	3.743652	2.659441	-1.596858
O	2.947627	3.473044	-2.013429
C	5.233788	2.689472	-2.027356
F	5.439167	3.600329	-2.981558
F	5.633278	1.490939	-2.484035
F	5.999695	3.009953	-0.960459

----

total energy -3780.26271065 a.u.

----

number of imaginary frequencies 0

----

molecule No.17a

----

optimized geometry

	x	y	z
C	1.722982	0.073959	-0.445728
C	0.873811	-0.973764	-0.187561
C	1.554253	-2.188161	-0.571113
C	2.788393	-1.835222	-1.048041
N	2.889494	-0.447343	-0.986867
S	4.313680	0.457117	-1.293540
O	3.849169	1.822210	-1.534042
O	5.057462	-0.314202	-2.284667
C	5.214407	0.423880	0.249949
C	5.026804	1.451588	1.175409
C	5.729895	1.412190	2.377421
C	6.613103	0.362945	2.668703
C	6.788294	-0.648972	1.712789
C	6.096707	-0.628343	0.504807
C	7.347289	0.312237	3.986728
C	1.020721	-3.587989	-0.499588
O	2.093216	-4.486983	-0.769317

C	-0.509042	-0.837619	0.389576
N	-1.558391	-1.363411	-0.504208
S	-2.671633	-2.471793	0.085476
C	-1.598973	-0.937010	-1.903446
O	-1.978978	-3.241631	1.128212
O	-3.262208	-3.127121	-1.086642
C	-3.990933	-1.578525	0.915286
C	-3.839219	-1.207558	2.252356
C	-4.865177	-0.506301	2.884936
C	-6.045812	-0.175918	2.204922
C	-6.178651	-0.579077	0.868269
C	-5.161403	-1.273147	0.218554
C	-7.145703	0.601462	2.887853
C	-1.583861	0.588042	-2.136765
S	-3.080116	1.398873	-1.397872
C	-1.455680	0.878527	-3.644064
O	-1.113475	2.225031	-3.939105
C	-2.382563	2.731813	-0.418432
C	-2.635397	2.760180	0.960378

C	-2.155365	3.816779	1.737607
C	-1.409087	4.839419	1.150266
C	-1.155479	4.810819	-0.223424
C	-1.650121	3.770971	-1.011361
H	1.605103	1.138600	-0.310083
H	3.587251	-2.437091	-1.448886
H	4.358881	2.274792	0.945872
H	5.595153	2.214236	3.098619
H	7.481503	-1.461692	1.914111
H	6.250705	-1.403064	-0.238535
H	8.344807	-0.124697	3.873144
H	6.803000	-0.304976	4.713650
H	7.458694	1.309787	4.422972
H	0.205169	-3.718523	-1.229687
H	0.577405	-3.773099	0.490008
H	1.720845	-5.380568	-0.797563
H	-0.709010	0.216946	0.620389
H	-0.580880	-1.398775	1.324069
H	-2.478491	-1.390114	-2.364740

H	-0.721444	-1.354614	-2.417330
H	-2.945163	-1.490413	2.797762
H	-4.752908	-0.225880	3.929508
H	-7.093593	-0.348564	0.327998
H	-5.275948	-1.593678	-0.810786
H	-7.117069	1.660272	2.598825
H	-7.053533	0.555267	3.977489
H	-8.135063	0.219385	2.613679
H	-0.715248	1.020305	-1.631670
H	-2.411372	0.693685	-4.143612
H	-0.714146	0.190315	-4.080020
H	-0.179963	2.348442	-3.704798
H	-3.203534	1.955181	1.417373
H	-2.357306	3.833046	2.805633
H	-1.030491	5.656997	1.757932
H	-0.584051	5.610368	-0.688052
H	-1.484553	3.754570	-2.083974

----

total energy -2879.59237401 a.u.

----

number of imaginary frequencies 0

----

molecule No.18ba

----

optimized geometry

	x	y	z
C	0.211952	0.064306	0.098588
C	0.137897	-1.055994	-0.663204
C	1.460332	-1.696024	-0.600315
C	2.270985	-0.865524	0.231075
N	1.518348	0.161115	0.618423
C	1.775915	-2.891490	-1.228646
S	3.146180	-3.896918	-1.296454
C	4.411383	-3.157724	-0.257804
C	4.454690	-3.480597	1.104541

C	5.472096	-2.944574	1.894418
C	6.438447	-2.111370	1.324554
C	6.398687	-1.814326	-0.040146
C	5.385361	-2.339637	-0.843372
C	-1.046779	-1.593314	-1.418237
N	-2.252015	-0.768858	-1.262188
S	-3.730282	-1.709945	-1.292760
C	-2.281972	0.450273	-2.101177
C	-2.613788	1.752305	-1.396718
C	-2.638990	2.941593	-2.325859
C	-2.873849	1.856930	-0.089469
S	2.084865	1.450800	1.757260
O	3.400028	0.960940	2.146159
O	0.964267	1.596874	2.670208
C	2.226153	2.870198	0.711507
C	3.452659	3.141265	0.094667
C	3.557947	4.273816	-0.705060
C	2.465800	5.137184	-0.894694
C	1.250936	4.833994	-0.258872

C	1.117855	3.708900	0.548575
C	2.606775	6.381263	-1.733721
O	-3.315420	-3.085979	-0.991017
O	-4.451977	-1.387136	-2.527642
C	-4.693198	-1.112393	0.085680
C	-5.787364	-0.281624	-0.154735
C	-6.555321	0.149665	0.925611
C	-6.248284	-0.237341	2.237146
C	-5.142174	-1.077998	2.445085
C	-4.366067	-1.524902	1.380472
C	-7.104777	0.206916	3.397300
H	-0.534297	0.808848	0.329771
H	3.295106	-0.961830	0.554815
H	0.985722	-3.341846	-1.830318
H	3.708451	-4.138453	1.539494
H	5.512807	-3.184647	2.952316
H	7.230548	-1.701847	1.943918
H	7.161156	-1.181322	-0.483853
H	5.355598	-2.122009	-1.906741

H	-1.285065	-2.596568	-1.054815
H	-0.779656	-1.703069	-2.483479
H	-2.979932	0.306811	-2.936653
H	-1.289738	0.551143	-2.561743
H	-2.871831	3.864914	-1.788389
H	-3.391913	2.810057	-3.114141
H	-1.674313	3.074568	-2.835487
H	-3.124956	2.816141	0.355699
H	-2.890373	0.992207	0.564932
H	4.305563	2.490921	0.256004
H	4.506882	4.499154	-1.183598
H	0.399269	5.495495	-0.389599
H	0.181942	3.494065	1.053000
H	1.650461	6.682277	-2.171342
H	3.329395	6.240585	-2.543190
H	2.963642	7.218375	-1.119675
H	-6.035524	0.009568	-1.169209
H	-7.411641	0.793700	0.744210
H	-4.894904	-1.396209	3.454563

H	-3.530612	-2.196501	1.547924
H	-6.506392	0.367029	4.300065
H	-7.857671	-0.554889	3.637751
H	-7.639294	1.134278	3.170561

---

total energy -2766.42365036 a.u.

---

number of imaginary frequencies 0

---

molecule No.19b

---

optimized geometry

	x	y	z
N	-2.085033	-1.089614	-1.362659
C	-1.316673	0.072492	-1.188622
C	-0.036097	-0.290614	-0.905654

C	-0.011388	-1.755803	-0.925033
C	-1.337188	-2.182425	-1.221013
S	-3.847434	-1.120047	-1.809243
O	-4.085246	-2.545602	-1.986751
O	-3.932216	-0.138886	-2.877020
C	-4.626305	-0.519573	-0.341863
C	-5.024544	-1.432809	0.639794
C	-5.647950	-0.945700	1.784287
C	-5.874799	0.428304	1.962637
C	-5.469829	1.315171	0.950344
C	-4.848028	0.856655	-0.204819
C	-6.532947	0.950127	3.214052
C	1.087893	-2.571330	-0.699495
S	1.108080	-4.269775	-0.803533
C	2.759583	-4.695669	-0.246064
C	3.257377	-4.218481	0.972474
C	4.531089	-4.614054	1.380729
C	5.281740	-5.492338	0.595407
C	4.764759	-5.977048	-0.607851

C	3.498811	-5.580070	-1.039056
C	1.127753	0.644860	-0.663383
N	1.084427	1.348663	0.634280
C	2.219937	1.135977	1.542532
S	0.403447	2.915606	0.619498
O	0.208977	3.275348	2.022164
O	-0.714994	2.824616	-0.328100
C	1.612107	4.030063	-0.087532
C	2.554223	4.644418	0.744118
C	3.507270	5.488377	0.180618
C	3.535076	5.738103	-1.200325
C	2.571274	5.118181	-2.008642
C	1.608116	4.270693	-1.465110
C	4.550304	6.684312	-1.792369
C	2.183167	-0.171276	2.315310
C	1.073001	-0.901427	2.458781
C	3.492693	-0.527056	2.974314
H	-1.758147	1.053618	-1.260985
H	-1.760951	-3.171647	-1.327644

H	-4.871137	-2.497250	0.498336
H	-5.972900	-1.645081	2.549153
H	-5.651508	2.379906	1.066042
H	-4.560235	1.544954	-0.992121
H	-5.798060	1.452373	3.855939
H	-6.989957	0.145242	3.795902
H	-7.308670	1.685810	2.976412
H	2.032203	-2.109916	-0.422454
H	2.660300	-3.558686	1.594867
H	4.928557	-4.248637	2.322798
H	6.267776	-5.804782	0.925345
H	5.347748	-6.659941	-1.218007
H	3.098694	-5.946036	-1.979731
H	1.179789	1.364546	-1.487542
H	2.067578	0.085746	-0.697298
H	2.219319	1.950225	2.274477
H	3.171395	1.212694	0.990409
H	2.519479	4.488866	1.817146
H	4.235212	5.972911	0.826129

H	2.565154	5.312340	-3.077985
H	0.841078	3.830261	-2.092932
H	4.741306	6.461296	-2.846422
H	5.502127	6.640158	-1.253598
H	4.193019	7.720736	-1.736430
H	1.064119	-1.795455	3.077523
H	0.133002	-0.595332	2.012148
H	3.400947	-1.426104	3.590521
H	3.839813	0.287472	3.624145
H	4.286960	-0.692317	2.233195

----

total energy -2766.43168618 a.u.

----

number of imaginary frequencies 0

----

molecule No.20b

----

optimized geometry

	x	y	z
N	-0.029881	0.187788	3.352596
C	-0.986404	-0.012058	2.254747
C	-0.287637	-0.882015	1.251315
C	0.969481	-1.180699	1.773165
C	1.029935	-0.477393	3.074473
C	-1.553820	1.341223	1.712198
C	-1.181544	1.663158	0.253338
C	-1.955906	0.771332	-0.745943
N	-2.145994	-0.606497	-0.272630
C	-0.923212	-1.364577	0.000970
S	1.135090	1.445692	-1.256212
O	1.827298	0.112358	-1.285525
O	0.260218	1.415783	0.227041
C	2.351914	2.661189	-0.753972
C	2.179868	3.975280	-1.193100
C	3.121337	4.939644	-0.835626
C	4.237012	4.606541	-0.055489

C	4.389237	3.272257	0.361596
C	3.460973	2.297759	0.013786
C	5.270590	5.644532	0.304973
C	1.950929	-1.899640	1.091125
S	3.492172	-2.231558	1.696354
C	4.350376	-2.937168	0.290641
C	4.266683	-2.350532	-0.979422
C	4.983650	-2.929252	-2.026747
C	5.784172	-4.052058	-1.802469
C	5.873381	-4.610739	-0.525201
C	5.155135	-4.056737	0.533519
S	-3.313506	-1.505236	-1.195462
O	-3.152079	-2.885568	-0.725486
O	-3.171494	-1.161446	-2.613151
C	-4.857948	-0.842608	-0.599020
C	-5.322220	-1.214715	0.667140
C	-6.543702	-0.717732	1.107370
C	-7.316052	0.137488	0.301986
C	-6.827036	0.484902	-0.964356

C	-5.604506	-0.002210	-1.426444
C	-8.655940	0.638135	0.781377
C	-1.455625	3.137703	-0.061079
H	-1.831691	-0.591745	2.657976
H	1.884127	-0.495061	3.748290
H	-1.159481	2.124833	2.364151
H	-2.643463	1.348391	1.800166
H	-1.475745	0.803008	-1.734040
H	-2.952527	1.206931	-0.864036
H	-1.215271	-2.416832	0.131931
H	-0.200625	-1.333662	-0.829882
H	1.326955	4.248414	-1.809460
H	2.991553	5.963764	-1.174545
H	5.252163	2.998604	0.963545
H	3.591789	1.265047	0.320374
H	6.147742	5.563796	-0.349939
H	5.622832	5.517186	1.333967
H	4.875367	6.659079	0.201594
H	1.751069	-2.239850	0.078884

H	3.666895	-1.460549	-1.150483
H	4.925321	-2.489169	-3.017552
H	6.344726	-4.488944	-2.623275
H	6.495205	-5.483416	-0.351453
H	5.210774	-4.496119	1.525059
H	-4.745637	-1.896262	1.283978
H	-6.913349	-1.005717	2.088060
H	-7.414416	1.137313	-1.604683
H	-5.240087	0.247640	-2.417202
H	-8.973708	1.528343	0.230939
H	-8.634258	0.882839	1.848455
H	-9.428113	-0.129139	0.640968
H	-2.494865	3.395992	0.167241
H	-1.283881	3.352793	-1.121751
H	-0.798634	3.776016	0.535968

----

total energy -2766.44631352 a.u.

----

number of imaginary frequencies 0

----

9. ^{1}H and ^{13}C NMR chart

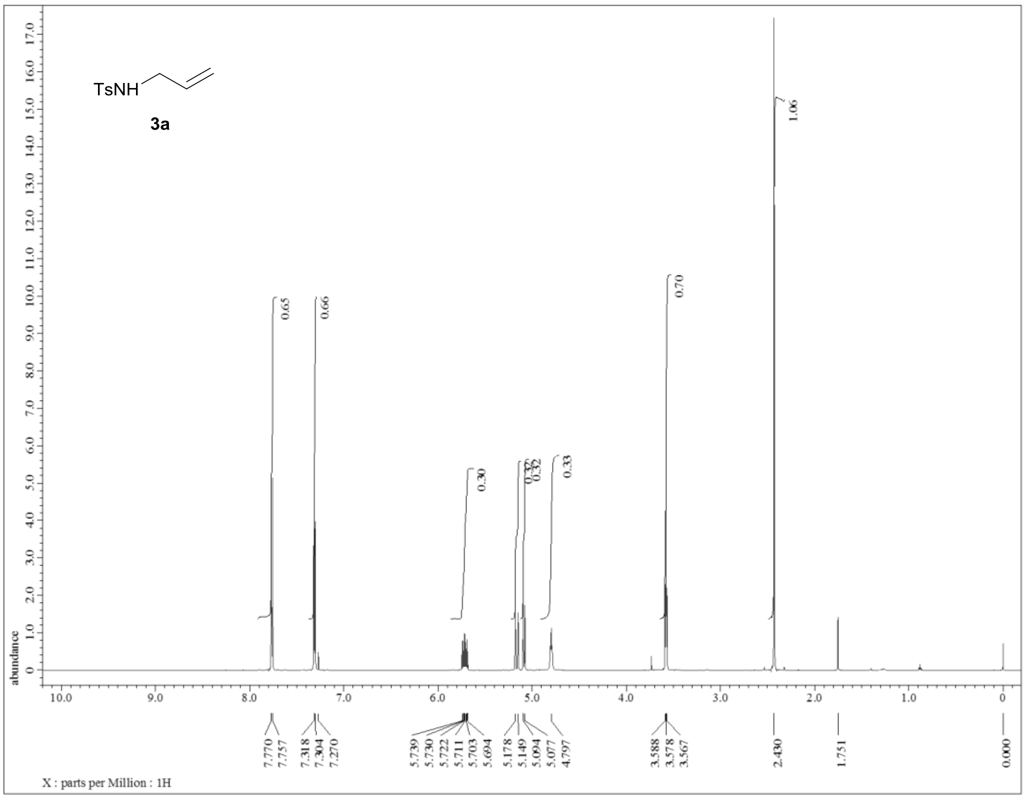

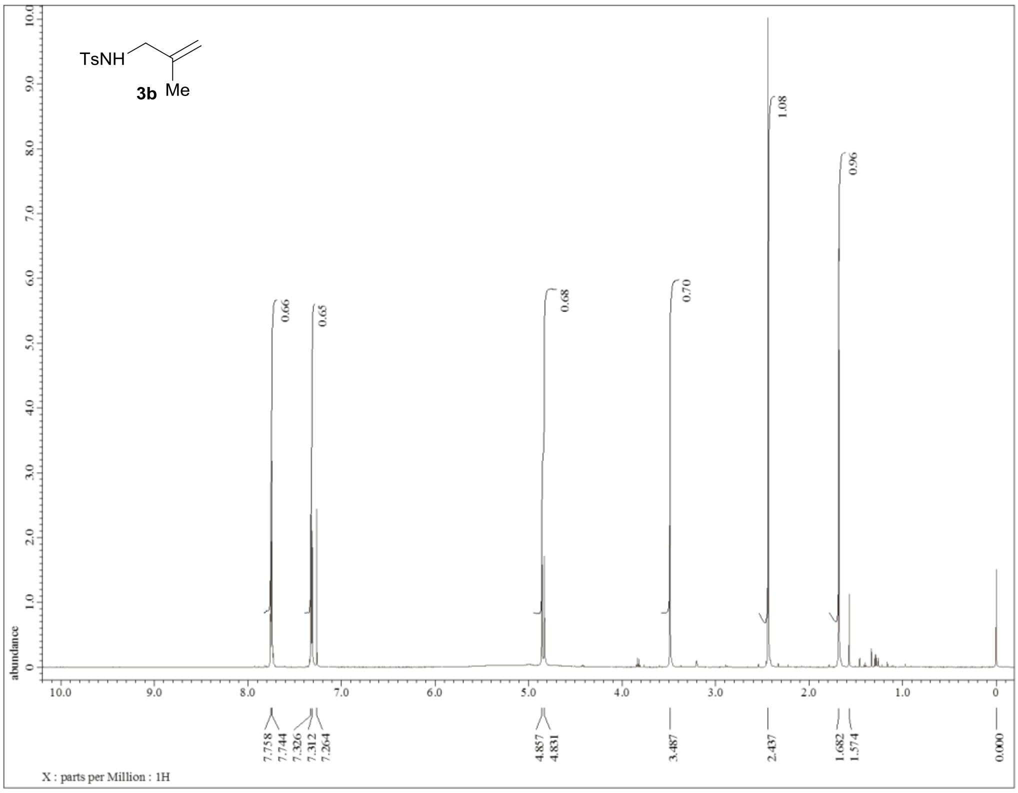

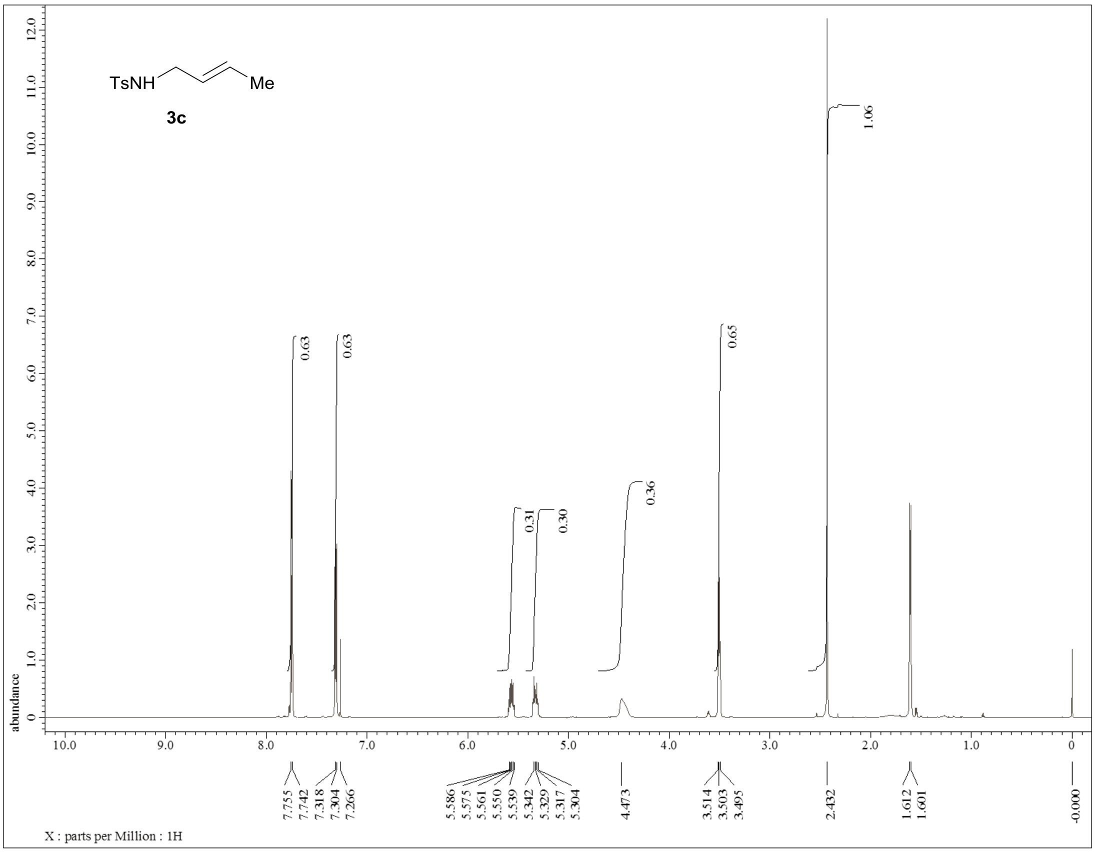

TsNH
Me
3c
abundance
X : parts per Million : 1H

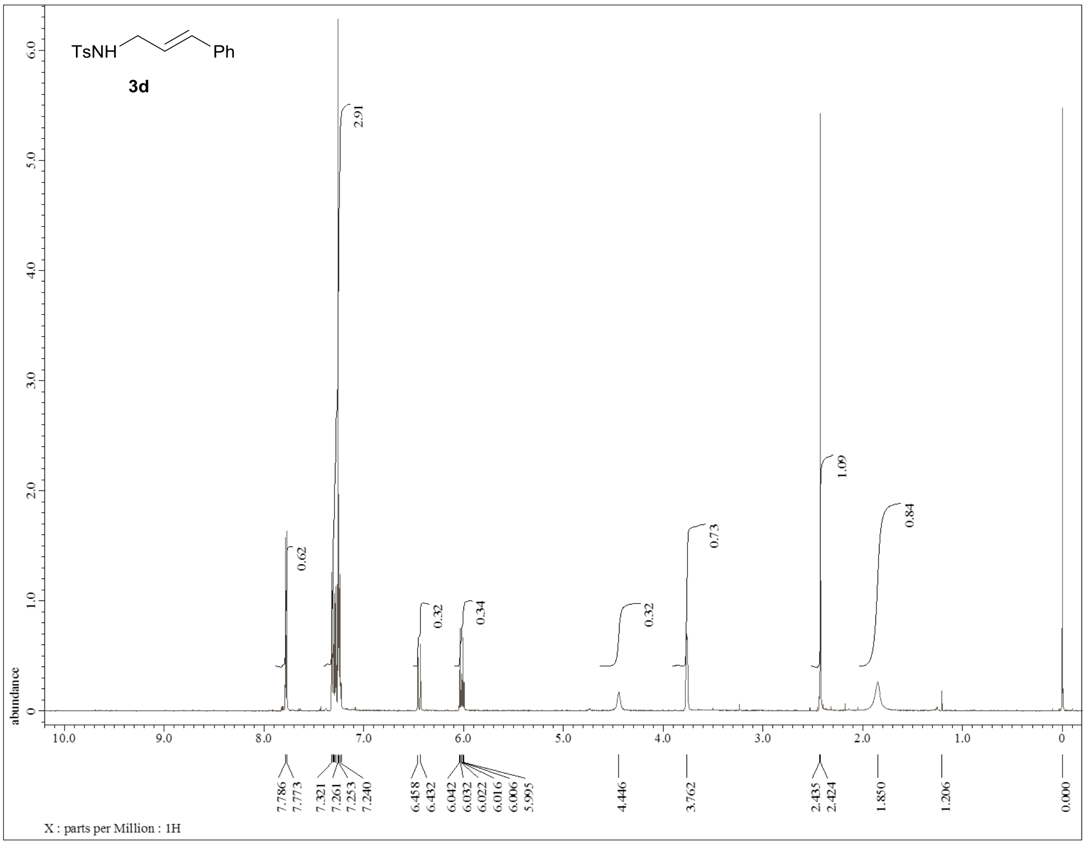

TsNH
Ph
3d
abundance
X : parts per Million : 1H

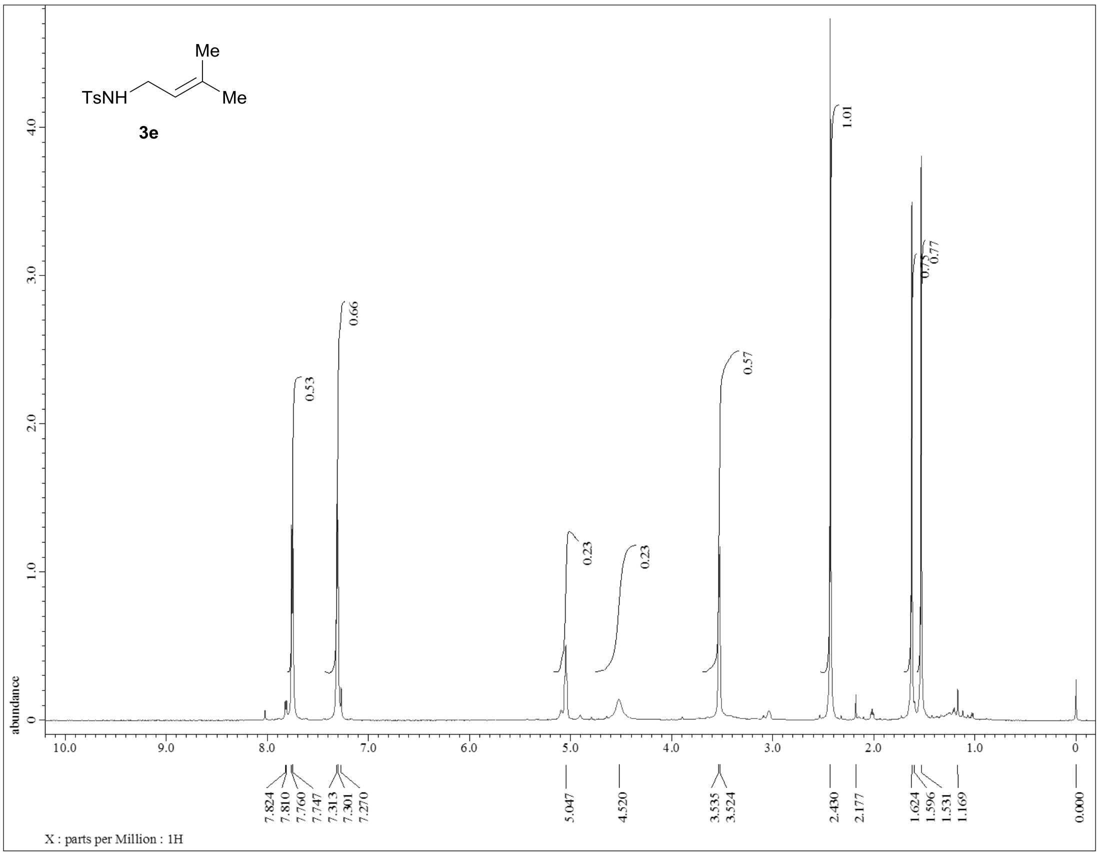

TsNH
Me
Me
3e
abundance
X : parts per Million : 1H

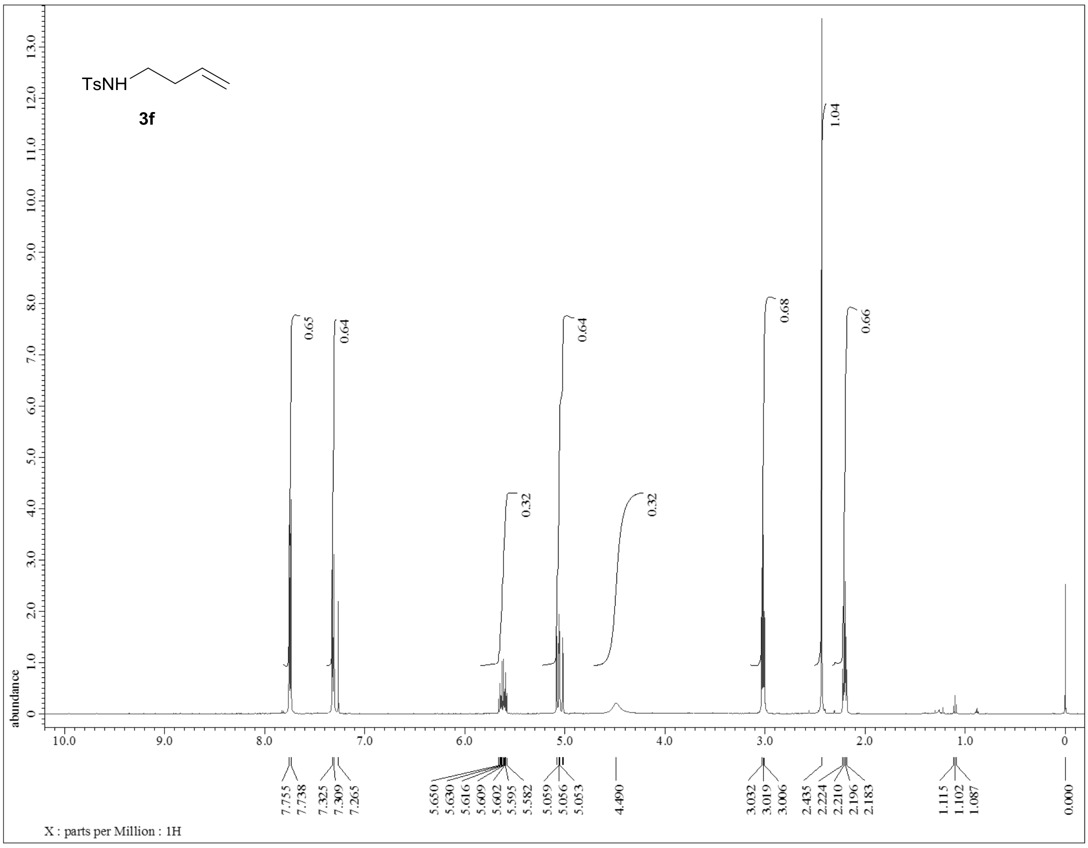

TsNH
3f
abundance
X : parts per Million : 1H

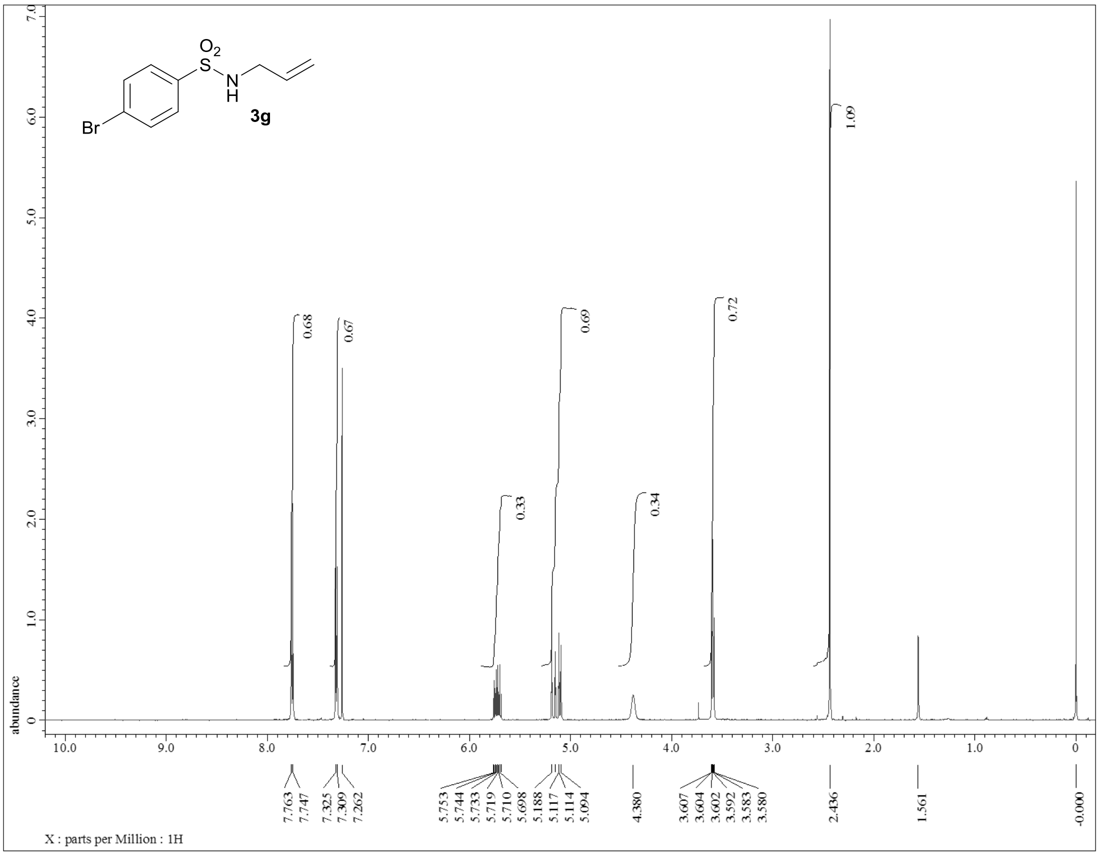

Br
O2
S
N
H
3g
abundance
X : parts per Million : 1H
0.68
0.67
0.33
0.69
0.34
0.72
1.09
7.763
7.747
7.325
7.309
7.262
5.753
5.744
5.733
5.719
5.710
5.698
5.188
5.117
5.114
5.094
4.380
3.607
3.604
3.602
3.592
3.583
3.580
2.436
1.561
-0.000

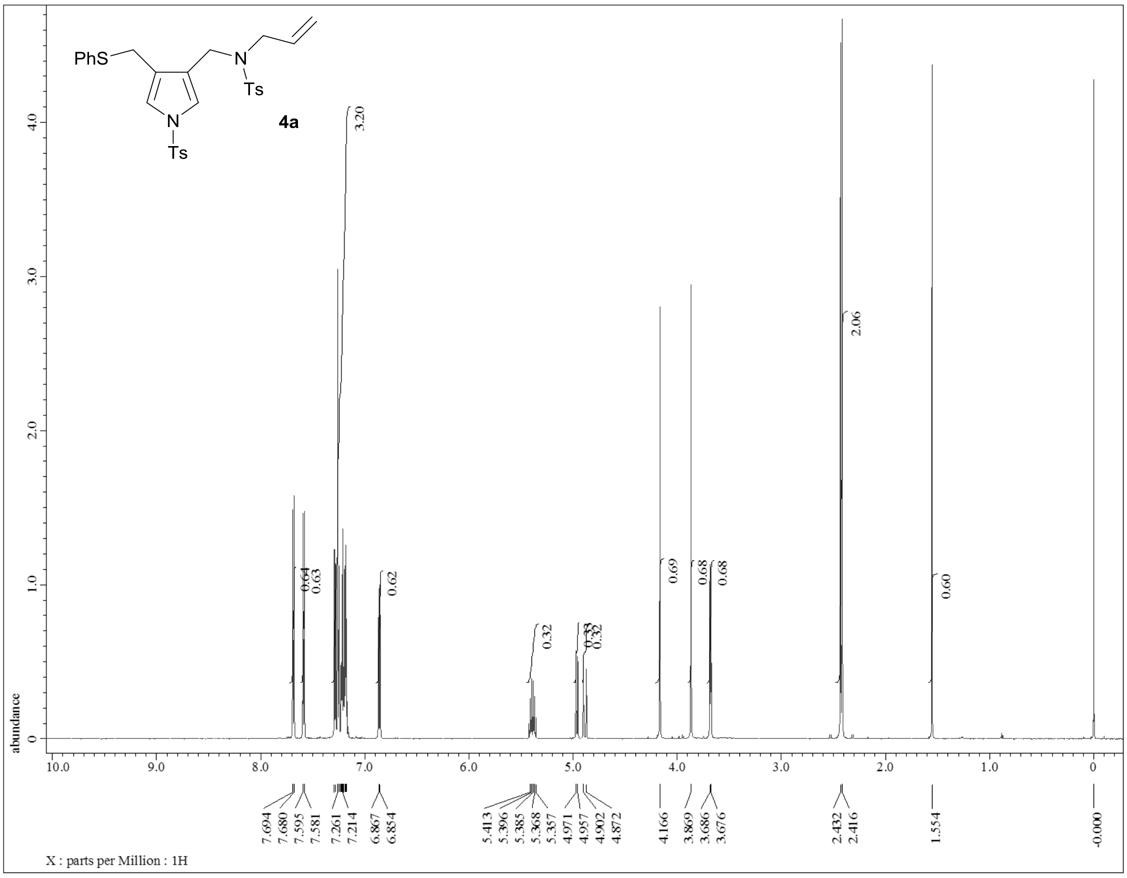
PhS
N
Ts
4a
abundance
3.20
0.64
0.63
0.62
0.32
0.33
0.32
0.69
0.68
0.68
2.06
0.60
7.694 7.680 7.595 7.581 7.261 7.214 6.867 6.854
5.413 5.396 5.385 5.368 5.357 4.971 4.957 4.902 4.872
4.166 3.869 3.686 3.676
2.432 2.416
1.554
-0.000
X : parts per Million : 1H

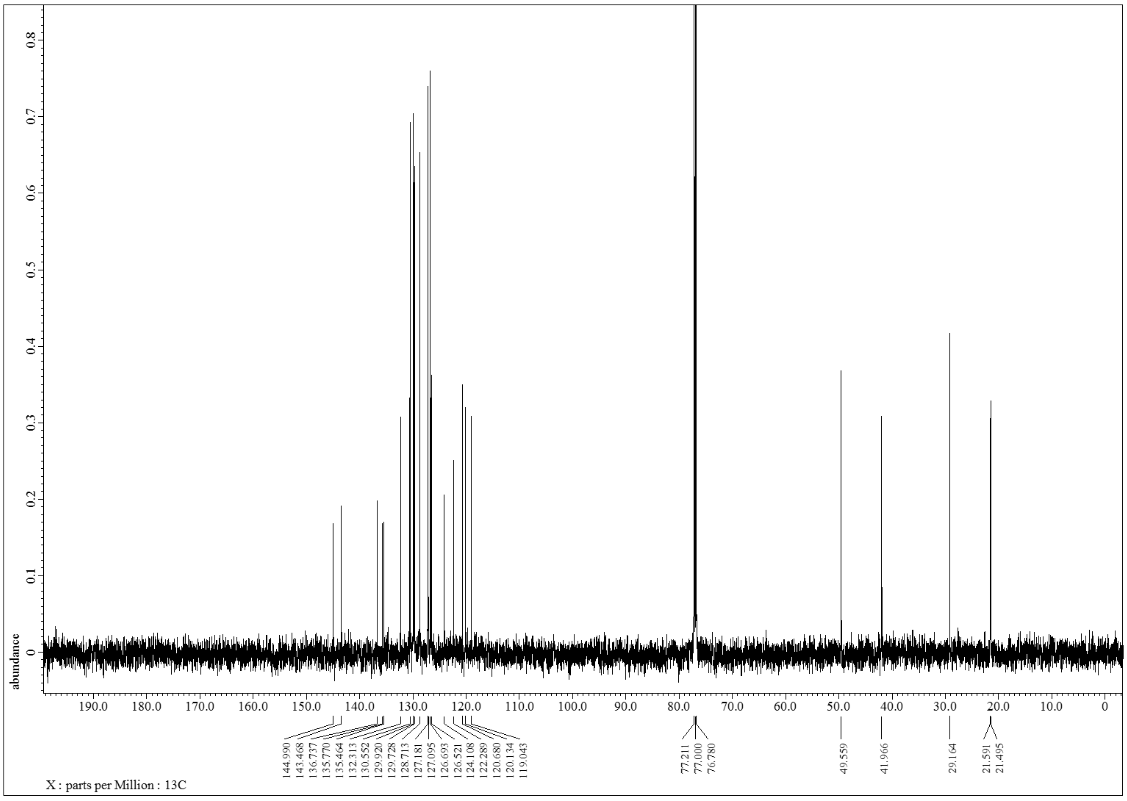
abundance
144.990 143.468 136.737 135.770 135.464 132.313 130.552 129.920 129.728 128.713 127.181 127.095 126.693 126.521 124.108 122.289 120.680 120.134 119.043
77.211 77.000 76.780
49.559
41.966
29.164
21.591 21.495
X : parts per Million : 13C

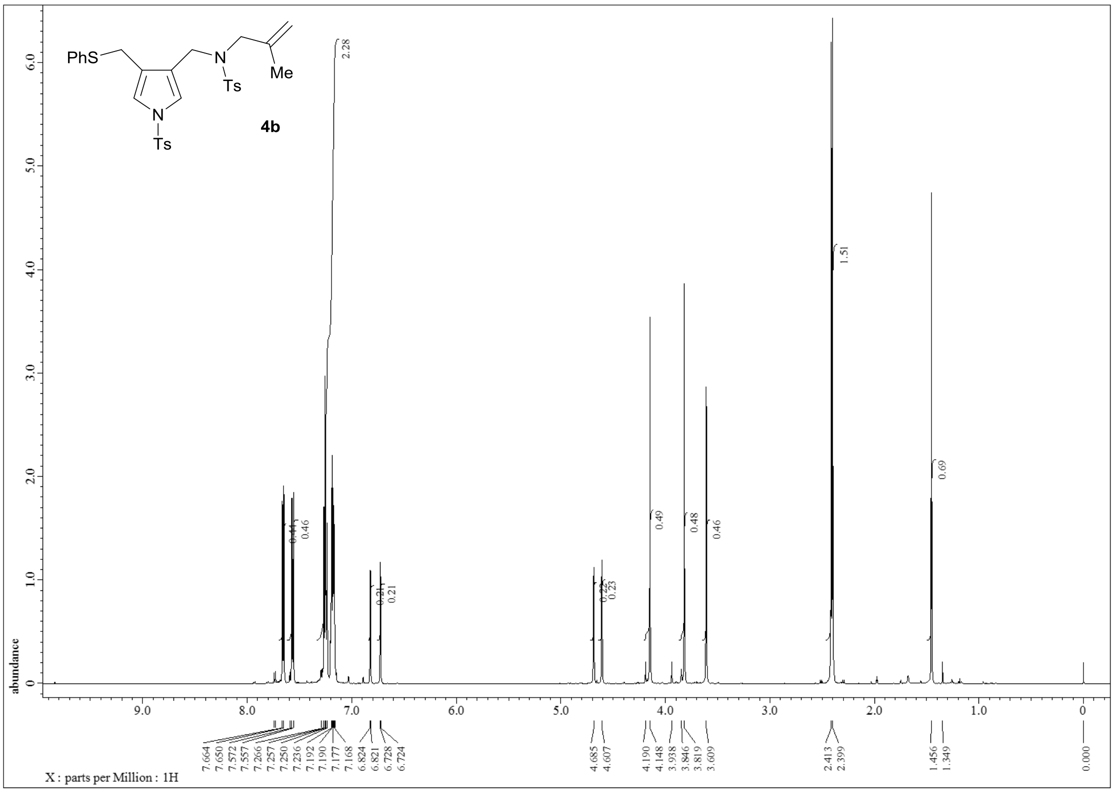
PhS
N
Me
Ts
N
Ts
4b
abundance
X : parts per Million : 1H
2.28
1.51
0.69
0.49
0.48
0.46
0.41
0.46
0.21
0.21
0.22
0.23
7.664
7.650
7.572
7.557
7.266
7.257
7.250
7.236
7.192
7.190
7.177
7.168
6.824
6.821
6.728
6.724
4.685
4.607
4.190
4.148
3.938
3.846
3.819
3.609
2.413
2.399
1.456
1.349
0.000

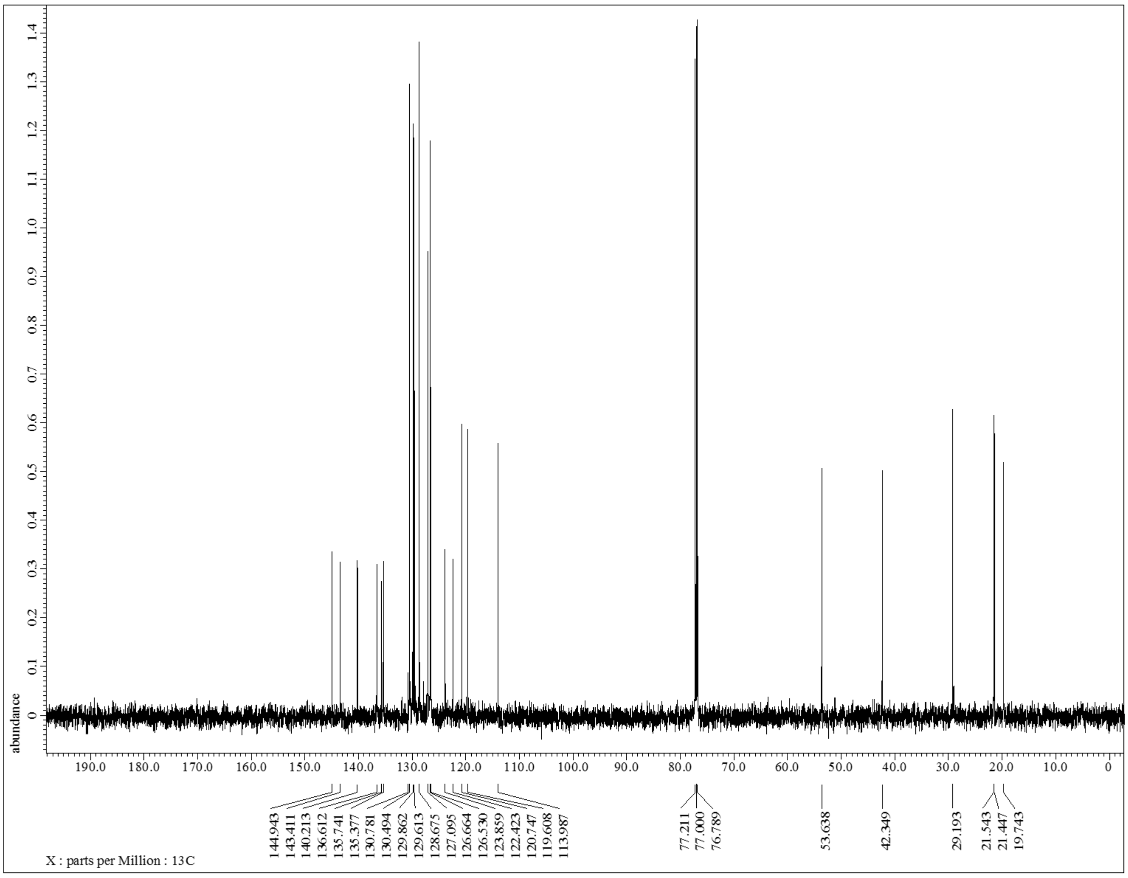
abundance
X : parts per Million : 13C
144.943
143.411
140.213
136.612
135.741
135.377
130.781
130.494
129.862
129.613
128.675
127.095
126.664
126.530
123.859
122.423
120.747
119.608
113.987
77.211
77.000
76.789
53.638
42.349
29.193
21.543
21.447
19.743

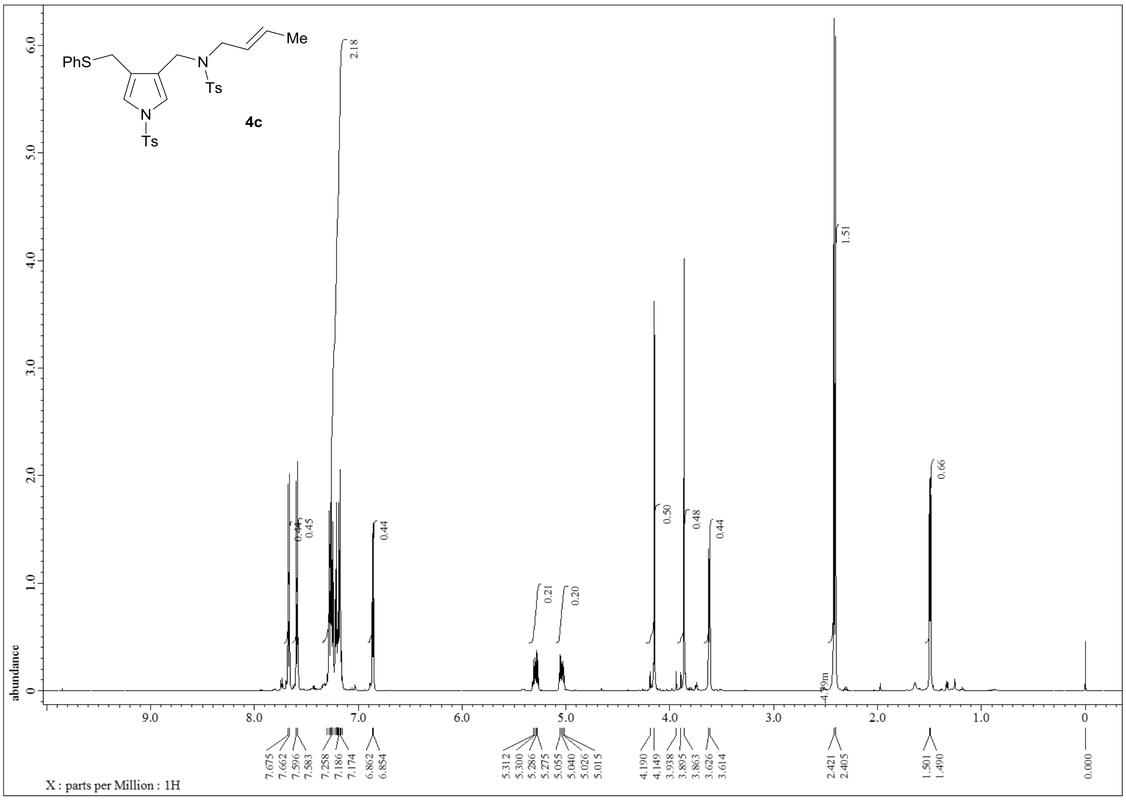
PhS
N
Ts
N
Ts
Me
4c
abundance
X : parts per Million : 1H

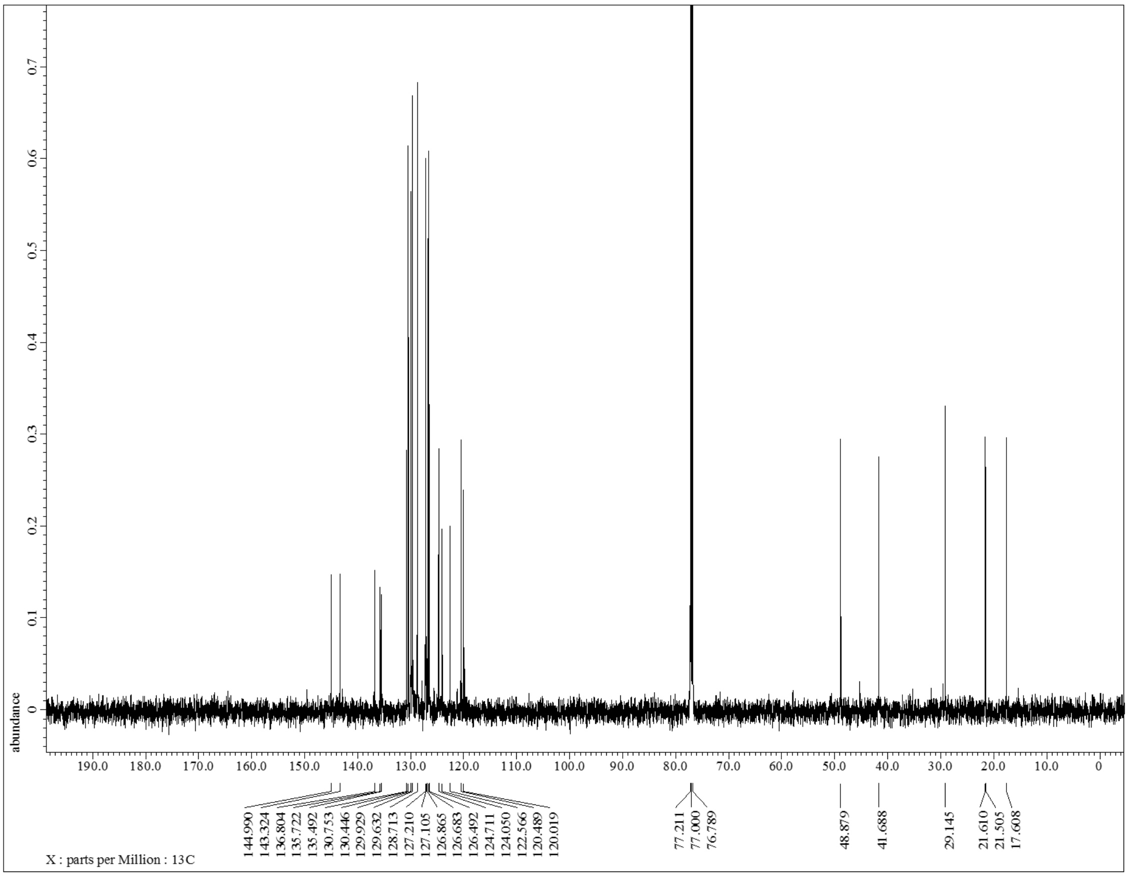
abundance
X : parts per Million : 13C

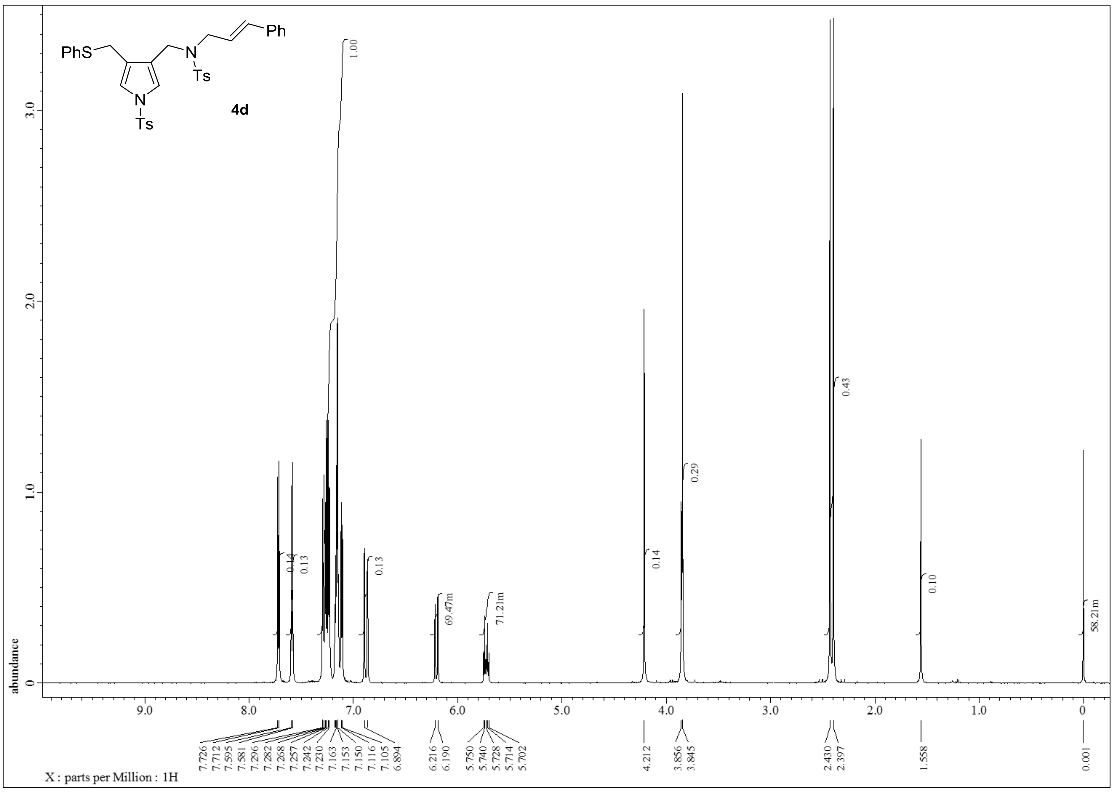
PhS
N
Ts
N
Ts
Ph
4d
abundance
1.00
0.14
0.13
0.13
69.47m
71.21m
0.14
0.29
0.43
0.10
58.21m
7.726 7.712 7.595 7.581 7.296 7.282 7.268 7.257 7.242 7.230 7.163 7.153 7.150 7.116 7.105 6.894
6.216 6.190
5.750 5.740 5.728 5.714 5.702
4.212
3.856 3.845
2.430 2.397
1.558
0.001
X : parts per Million : 1H

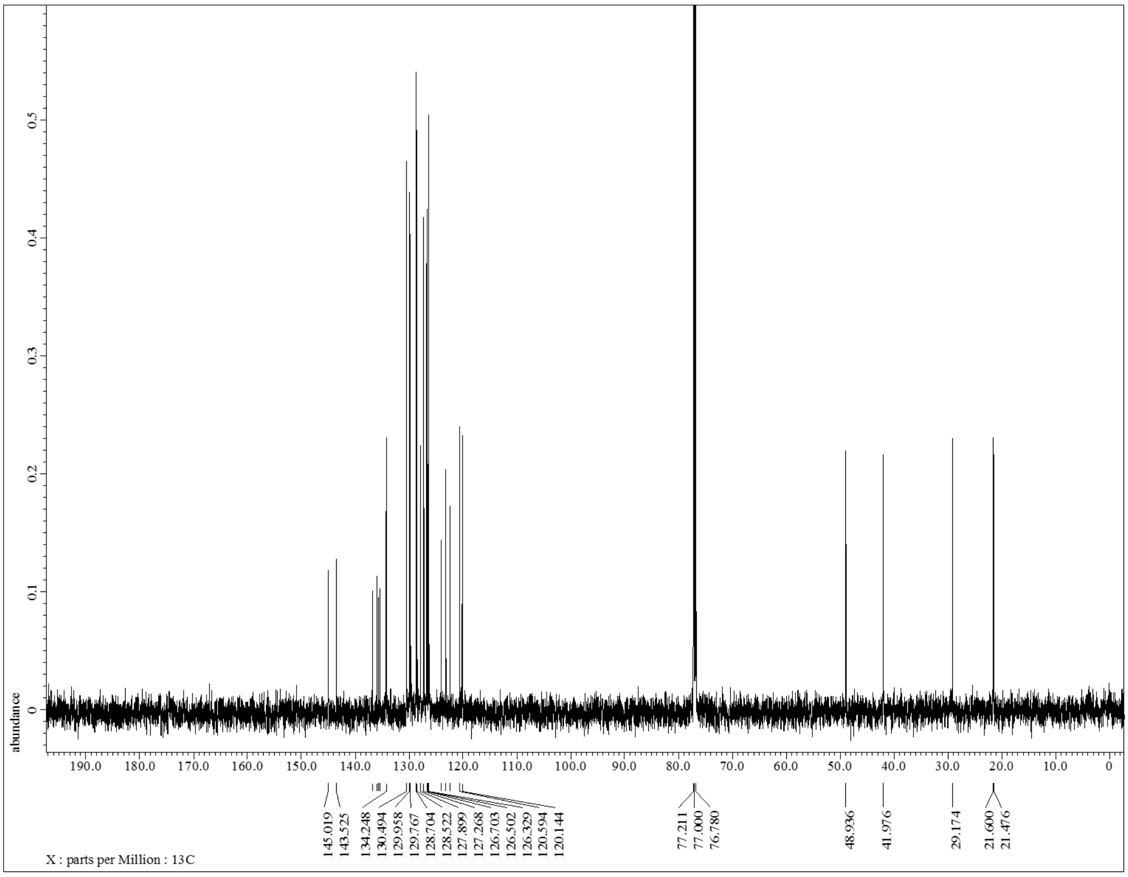
abundance
145.019 143.525 134.248 130.494 129.958 129.767 128.704 128.522 127.899 127.268 126.703 126.502 126.329 120.594 120.144
77.211 77.000 76.780
48.936
41.976
29.174
21.600 21.476
X : parts per Million : 13C

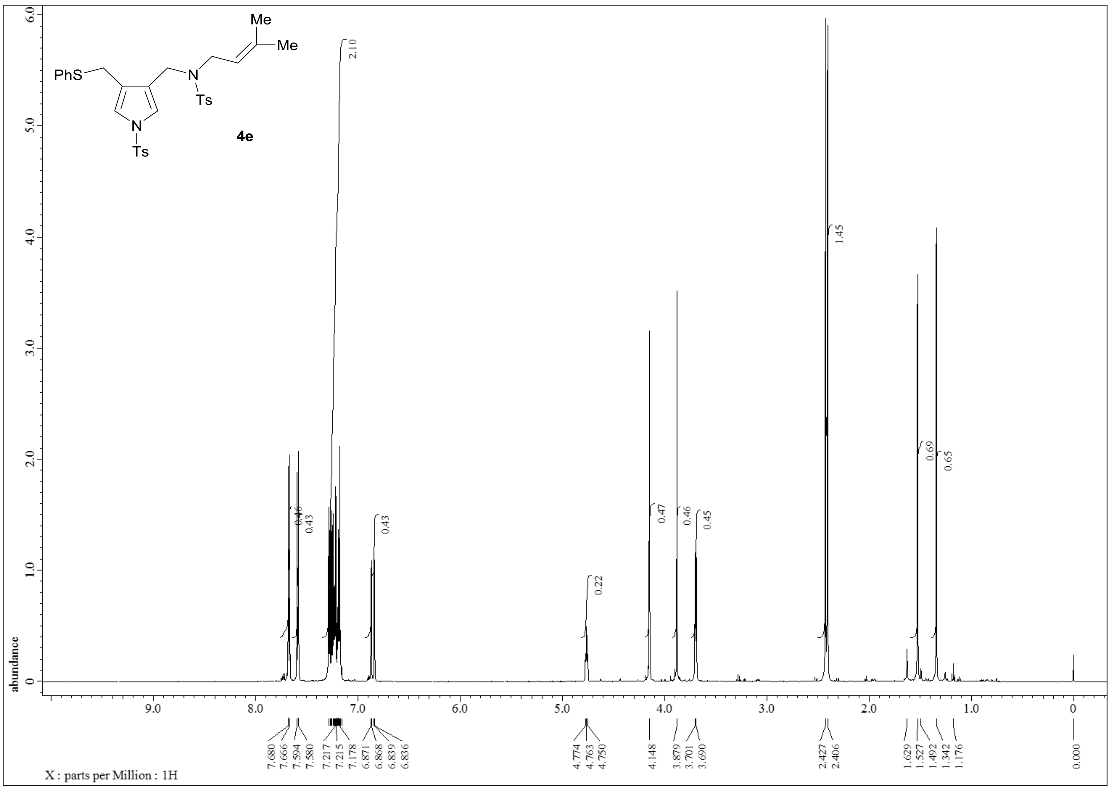
Me
Me
PhS
N
Ts
N
Ts
4e
abundance
X : parts per Million : 1H
2.10
0.46
0.43
0.43
0.22
0.47
0.46
0.45
1.45
0.69
0.65
7.680
7.666
7.594
7.580
7.217
7.215
7.178
6.871
6.868
6.839
6.836
4.774
4.763
4.750
4.148
3.879
3.701
3.690
2.427
2.406
1.629
1.527
1.492
1.342
1.176
0.000

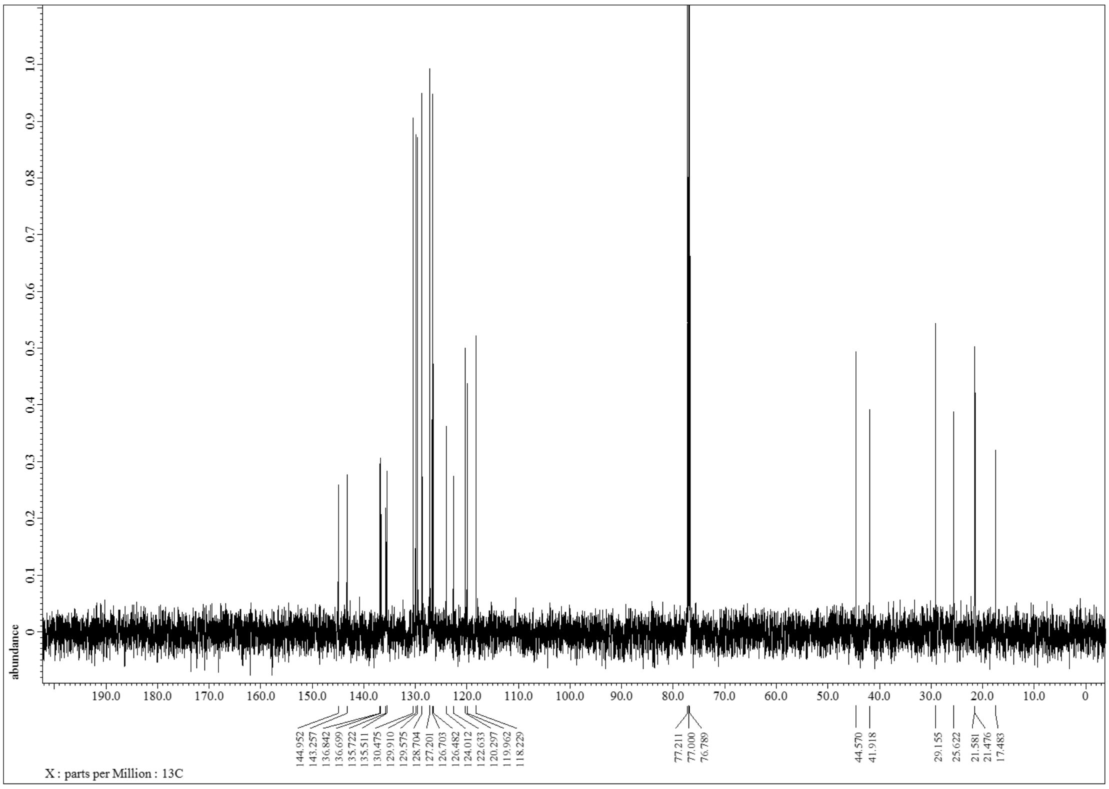
abundance
X : parts per Million : 13C
144.952
143.257
136.842
136.699
135.722
135.511
130.475
129.910
129.575
128.704
127.201
126.703
126.482
124.012
122.633
120.297
119.962
118.229
77.211
77.000
76.789
44.570
41.918
29.155
25.622
21.581
21.476
17.483

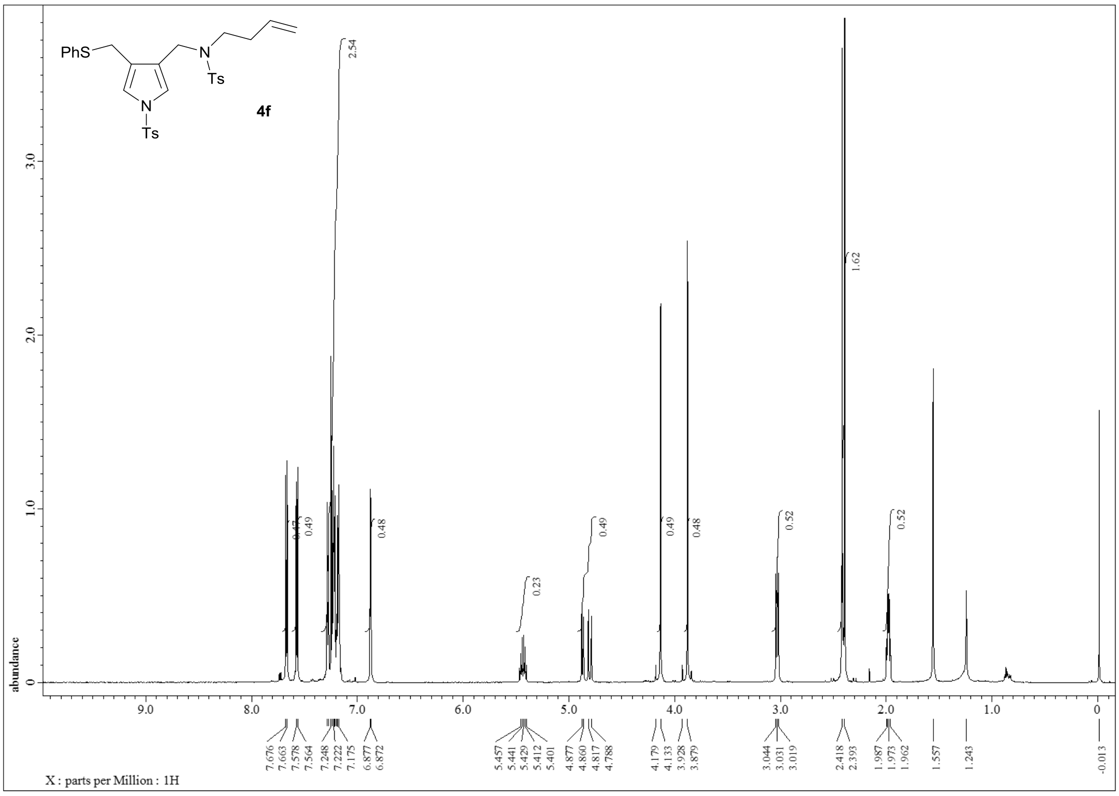
PhS
N
Ts
N
Ts
4f
X : parts per Million : 1H
abundance

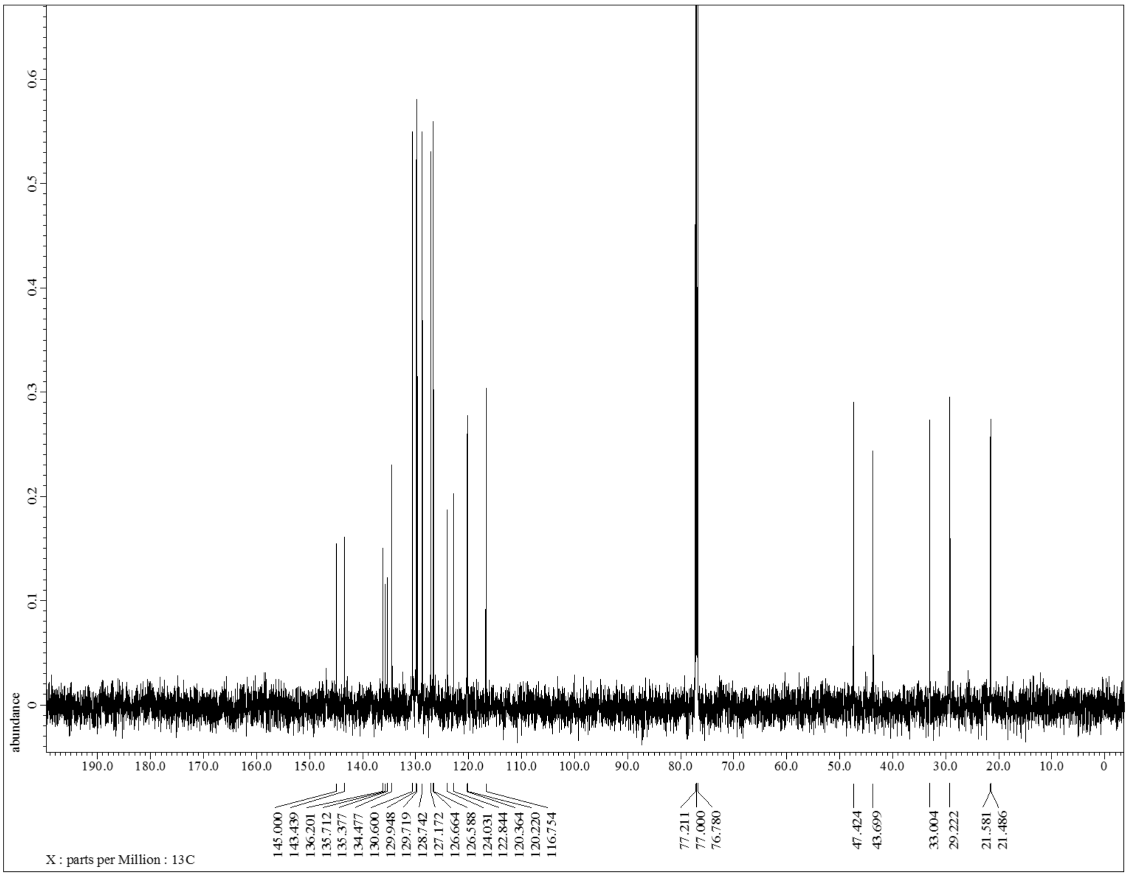
X : parts per Million : 13C
abundance

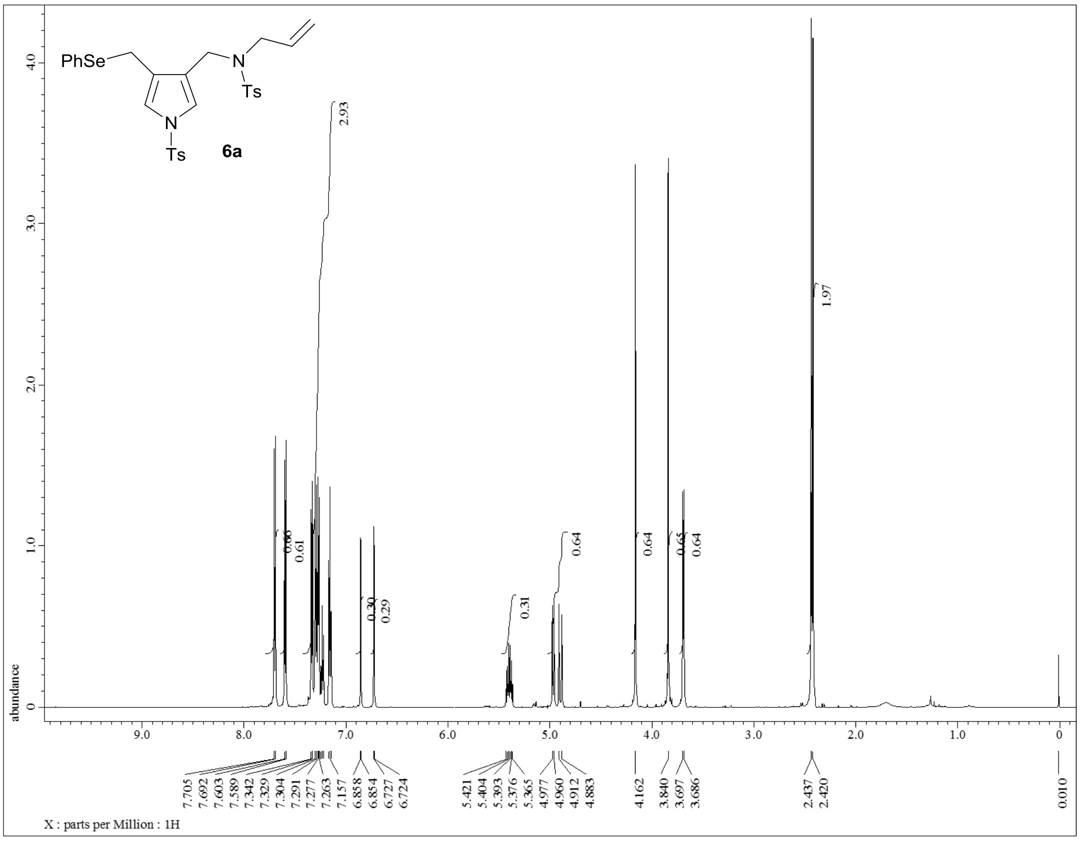
PhSe
N
Ts
N
Ts
6a
X : parts per Million : 1H
abundance

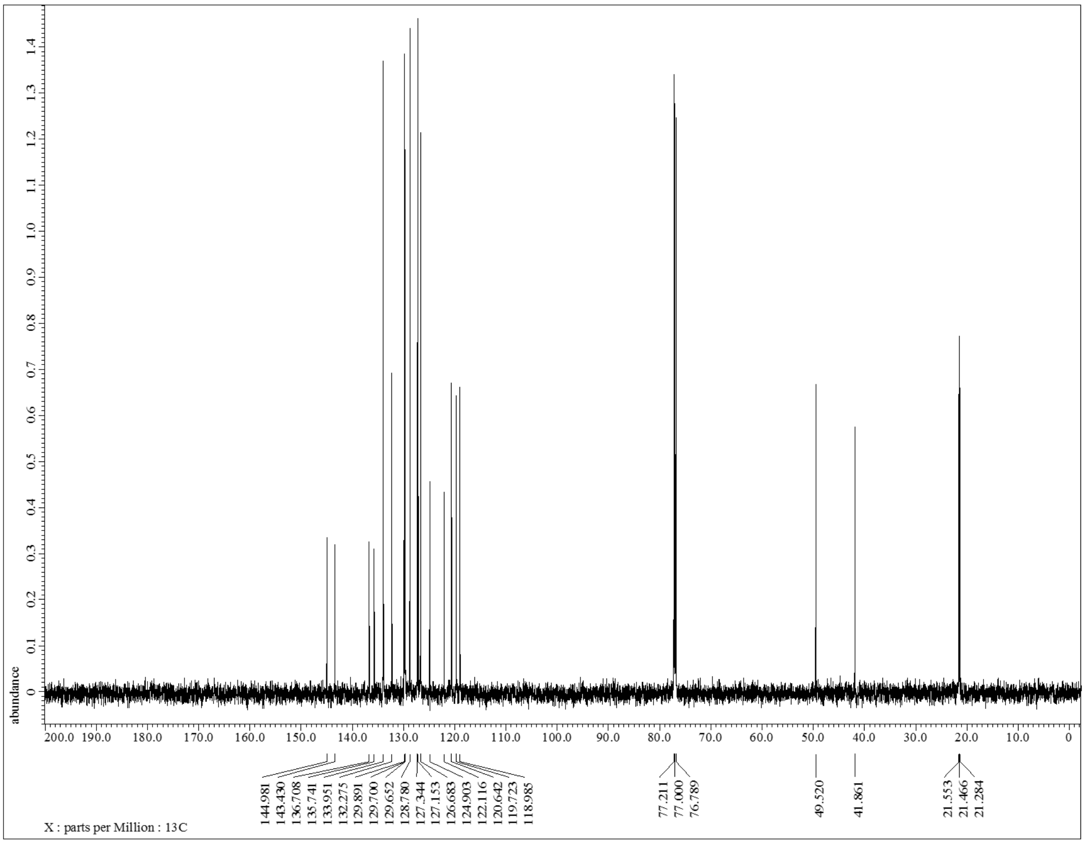
X : parts per Million : 13C
abundance

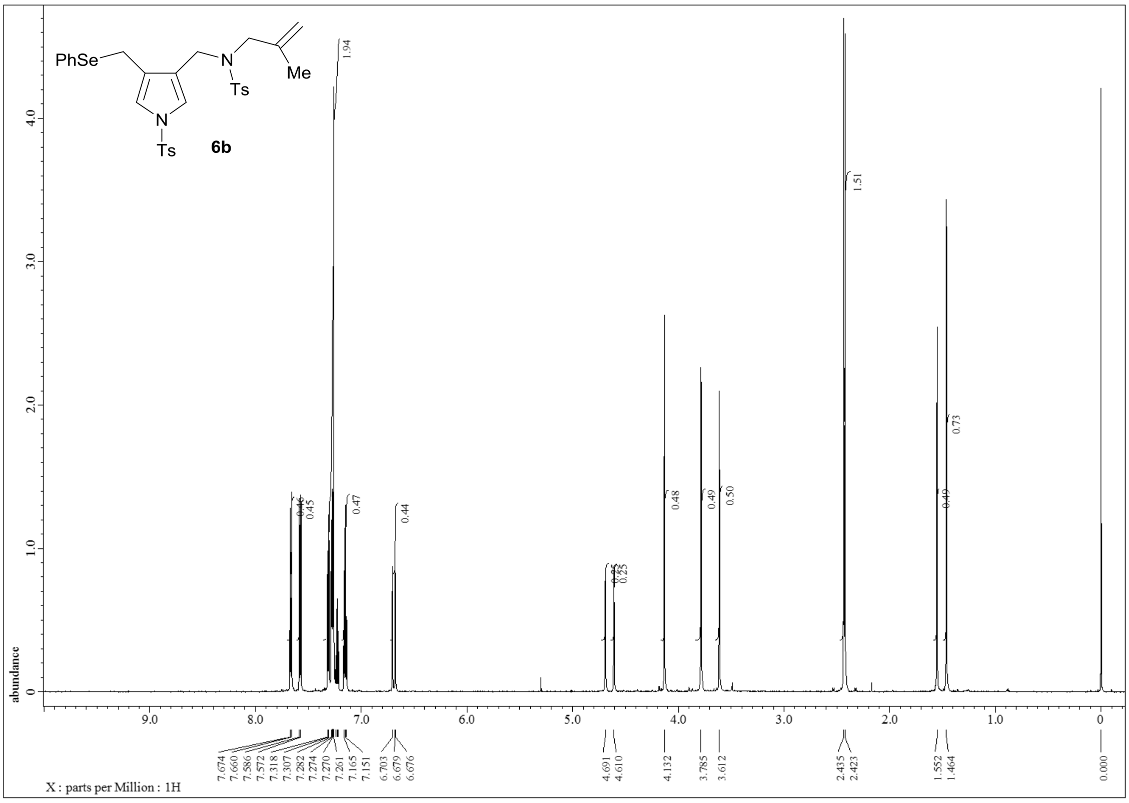
PhSe
N
Ts
Me
N
Ts
6b
abundance
X : parts per Million : 1H

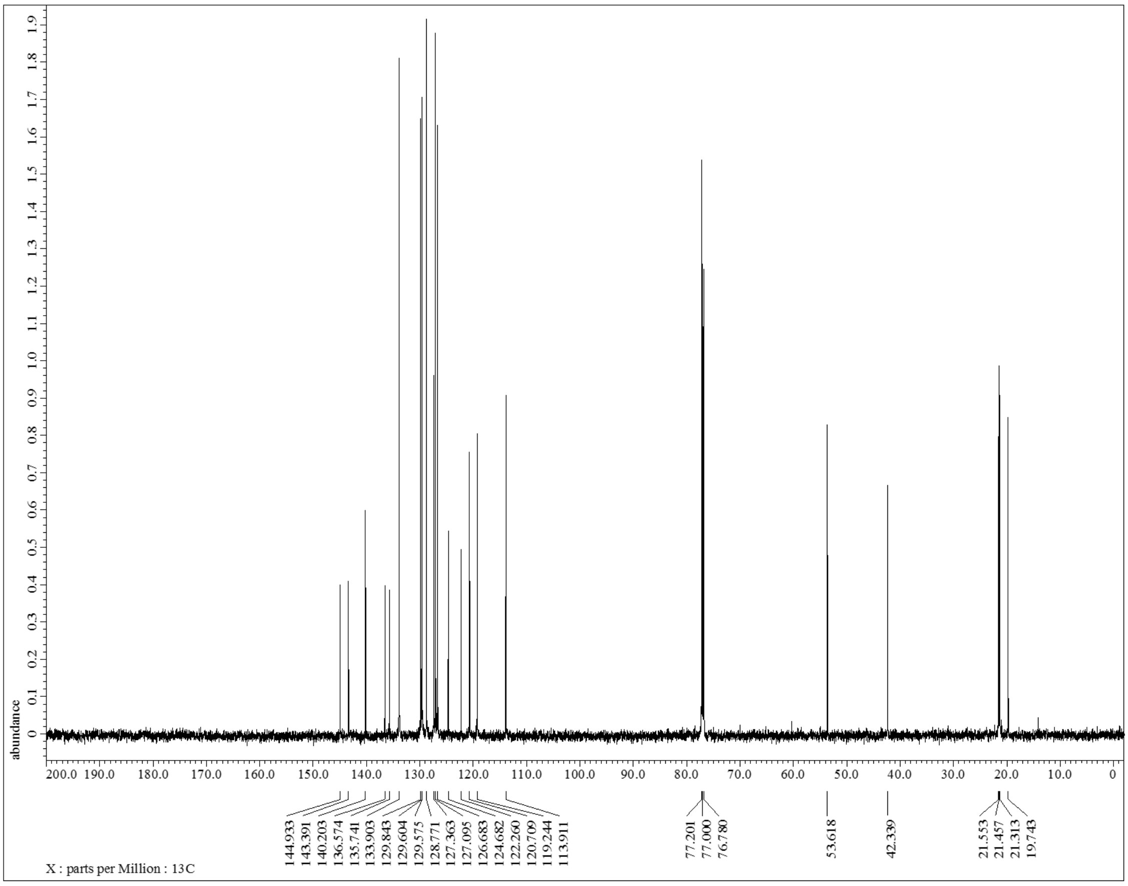
abundance
X : parts per Million : 13C

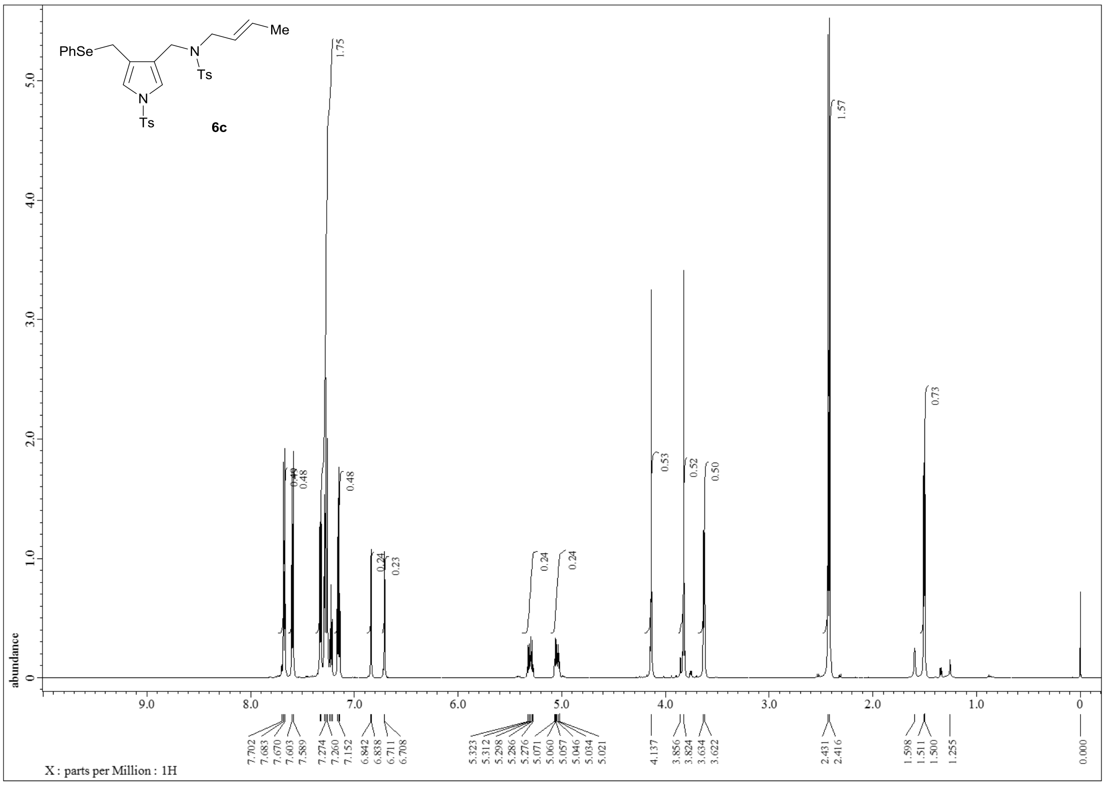
PhSe
N
Ts
Me
6c
abundance
X : parts per Million : 1H
7.702
7.683
7.670
7.603
7.589
7.274
7.260
7.152
6.842
6.838
6.711
6.708
5.323
5.312
5.298
5.286
5.276
5.071
5.060
5.057
5.046
5.034
5.021
4.137
3.856
3.824
3.634
3.622
2.431
2.416
1.598
1.511
1.500
1.255
0.000

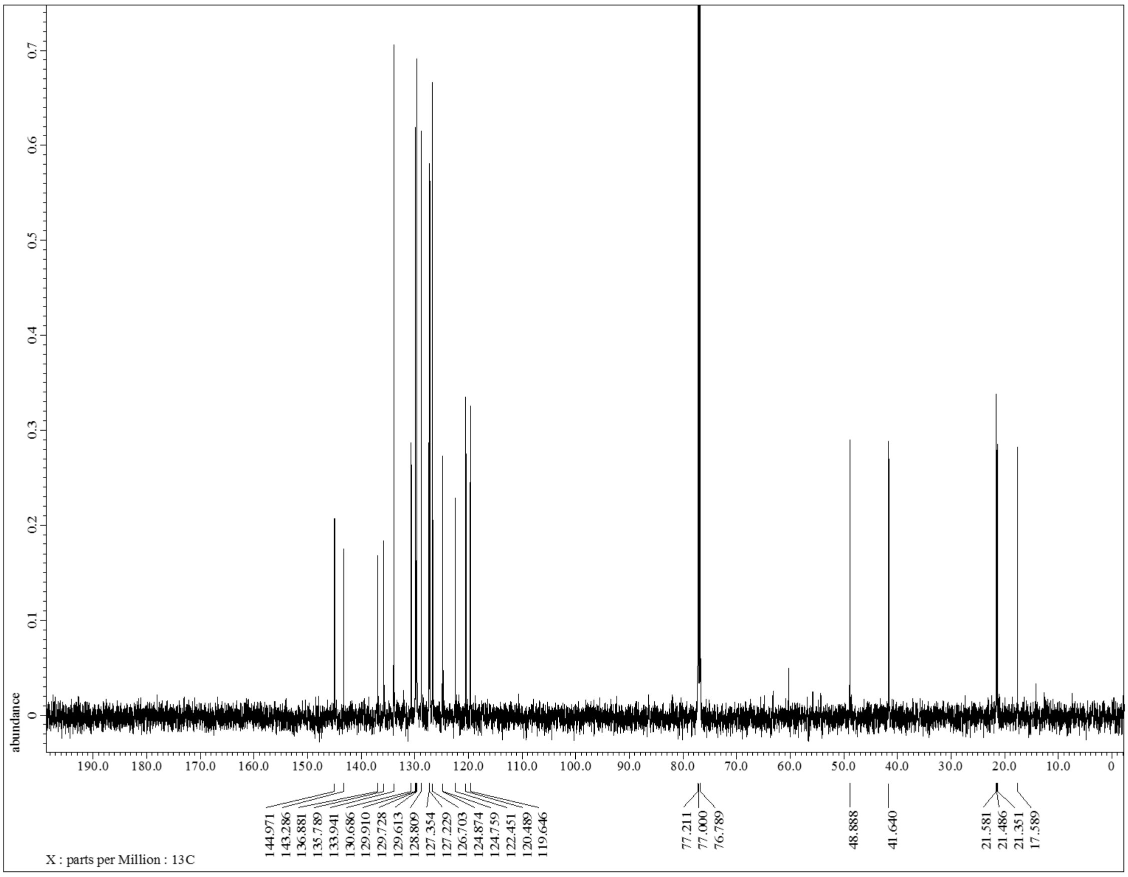
abundance
X : parts per Million : 13C
144.971
143.286
136.881
135.789
133.941
130.686
129.910
129.728
129.613
128.809
127.354
127.229
126.703
124.874
124.759
122.451
120.489
119.646
77.211
77.000
76.789
48.888
41.640
21.581
21.486
21.351
17.589

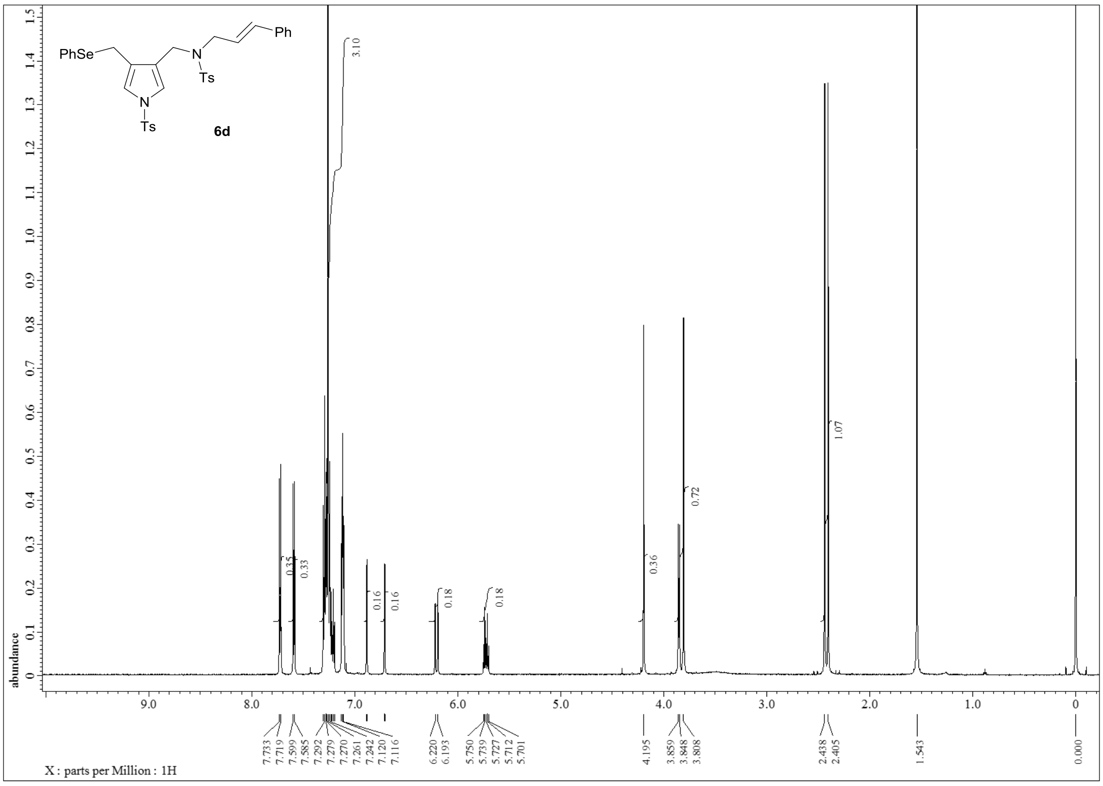
PhSe
N
Ts
N
Ts
Ph
6d
X : parts per Million : 1H
abundance

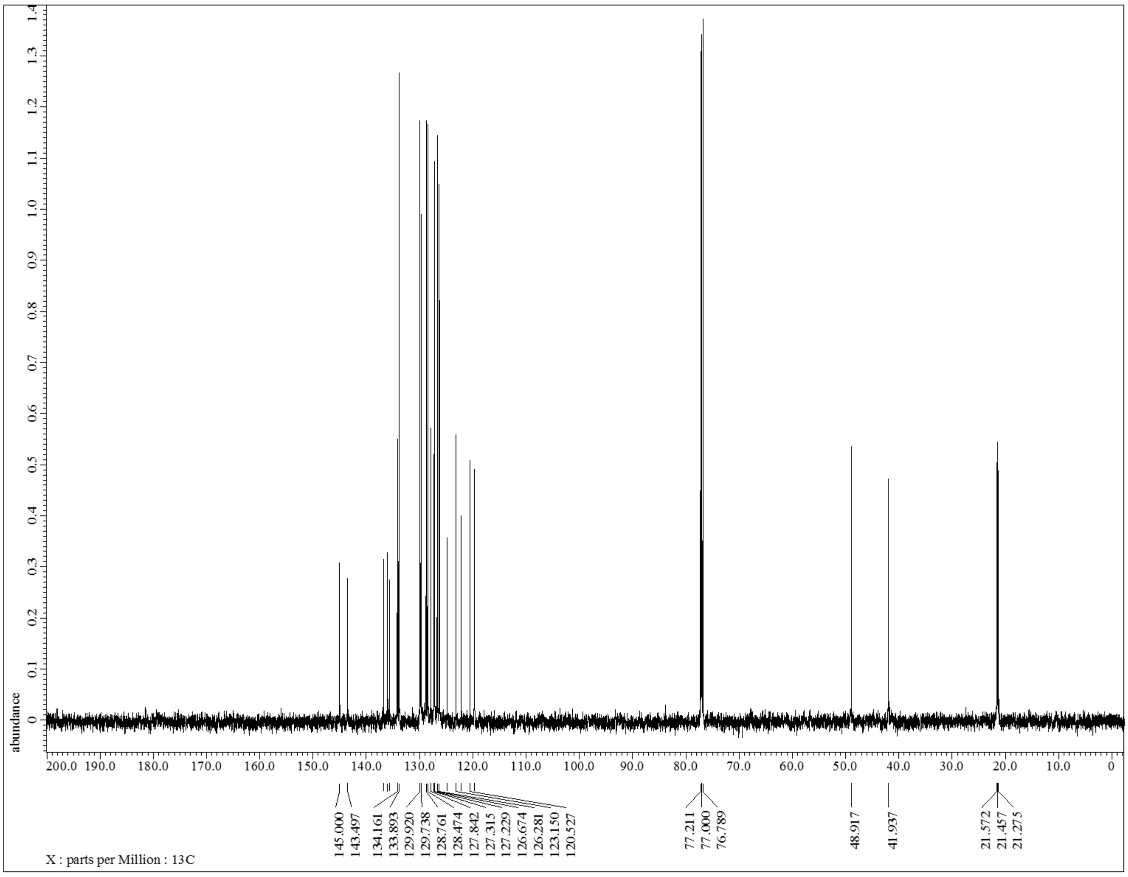
X : parts per Million : 13C
abundance

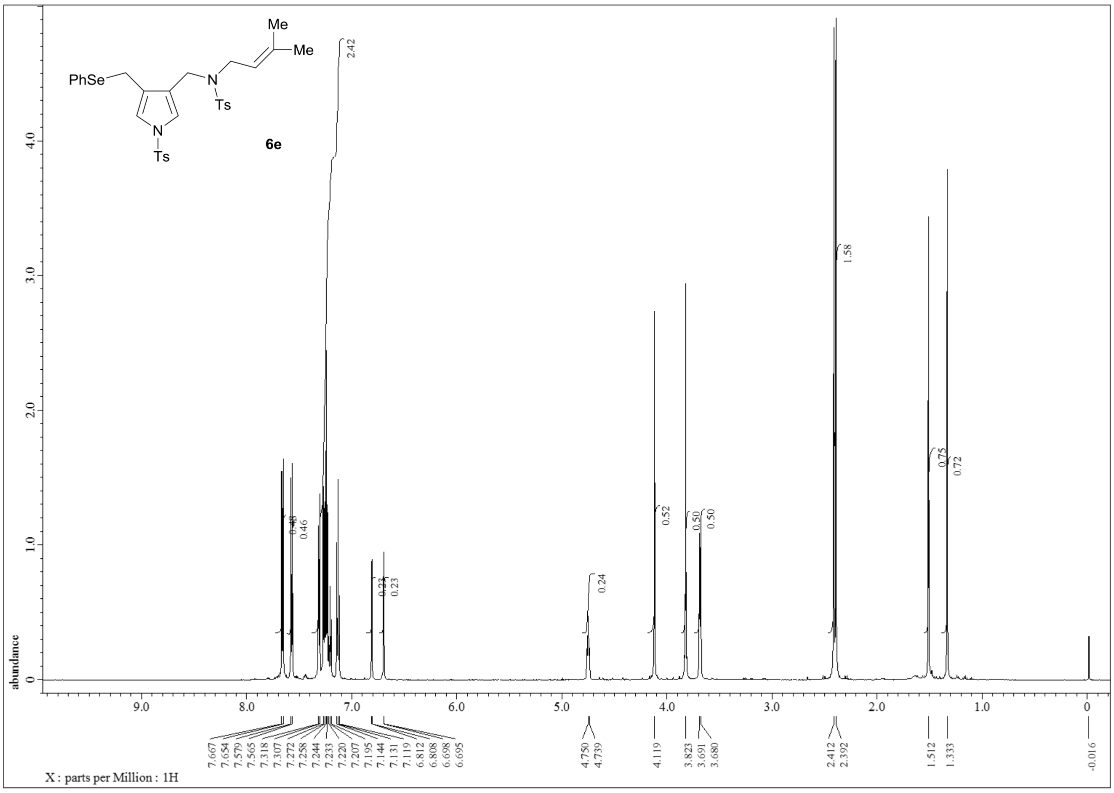
Me
Me
PhSe
N
Ts
N
Ts
6e
2.42
1.58
0.75
0.72
0.46
0.46
0.23
0.23
0.24
0.52
0.50
0.50
abundance
X : parts per Million : 1H
7.667
7.654
7.579
7.565
7.318
7.307
7.272
7.258
7.244
7.233
7.220
7.207
7.195
7.144
7.131
7.119
6.812
6.808
6.698
6.695
4.750
4.739
4.119
3.823
3.691
3.680
2.412
2.392
1.512
1.333
-0.016

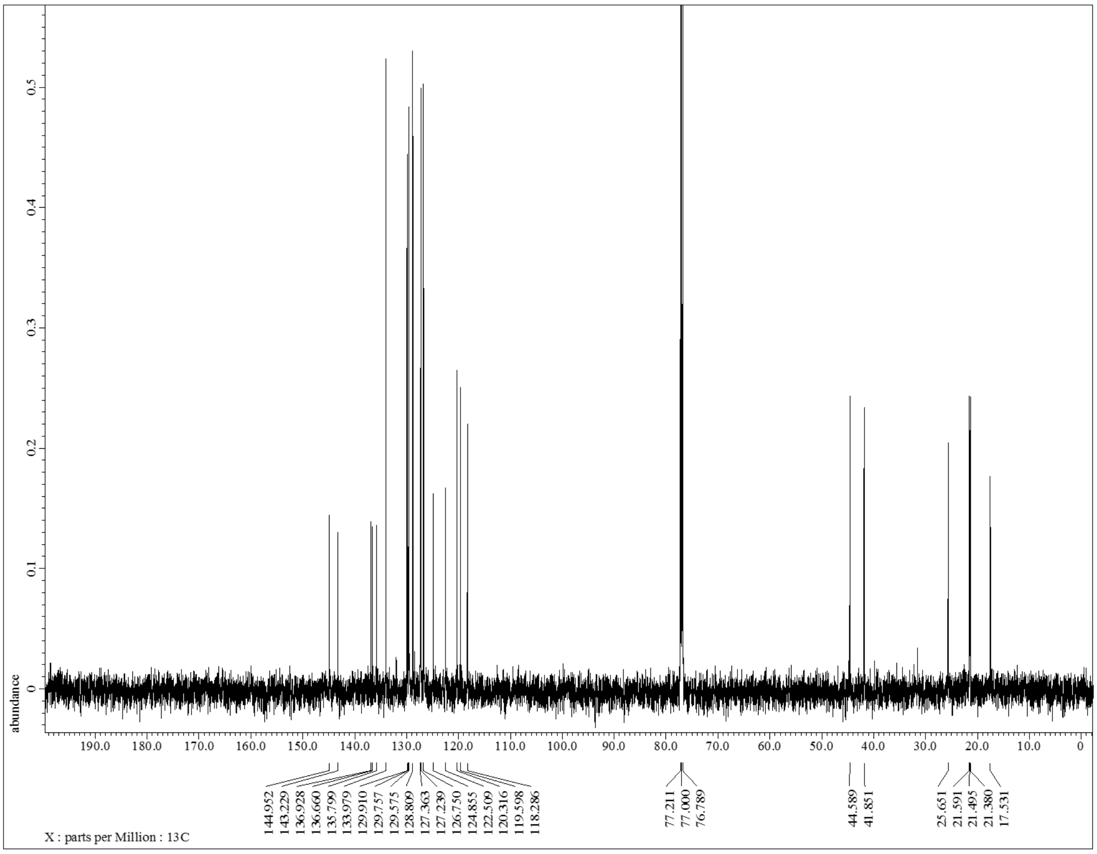
abundance
X : parts per Million : 13C
144.952
143.229
136.928
136.660
135.799
133.979
129.910
129.757
129.575
128.809
127.363
127.239
126.750
124.855
122.509
120.316
119.598
118.286
77.211
77.000
76.789
44.589
41.851
25.651
21.591
21.495
21.380
17.531

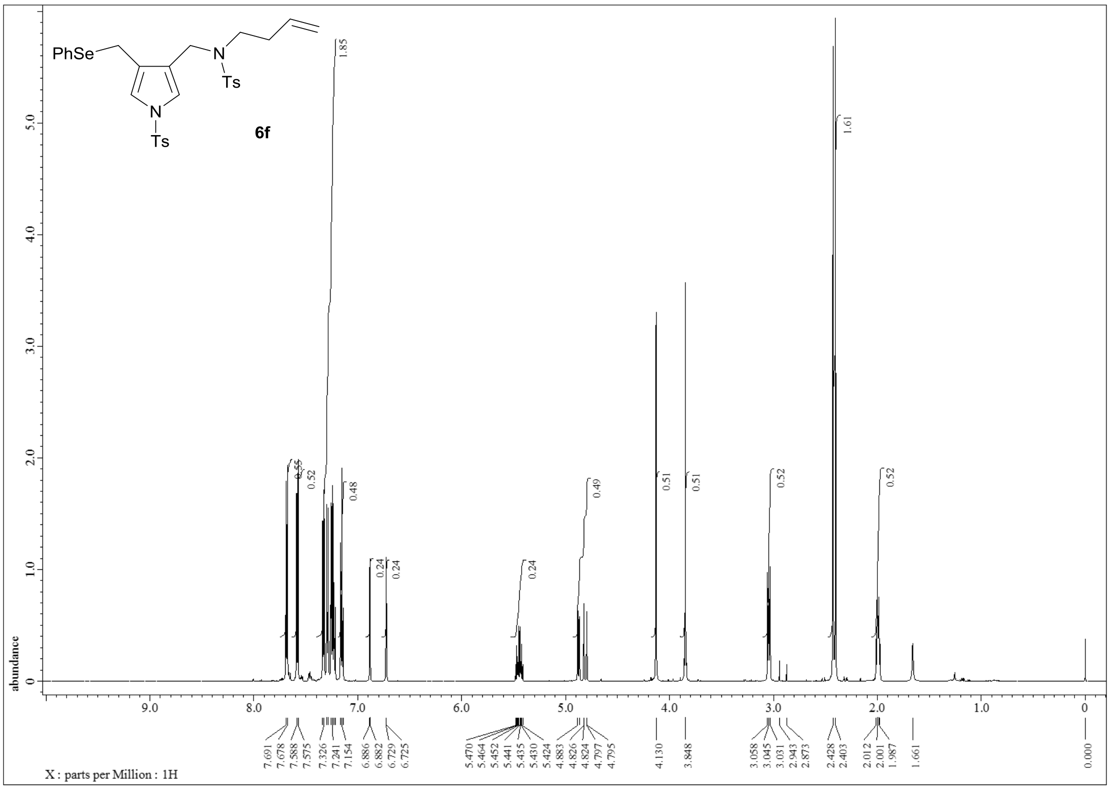
PhSe
N
Ts
N
Ts
6f
abundance
X : parts per Million : 1H

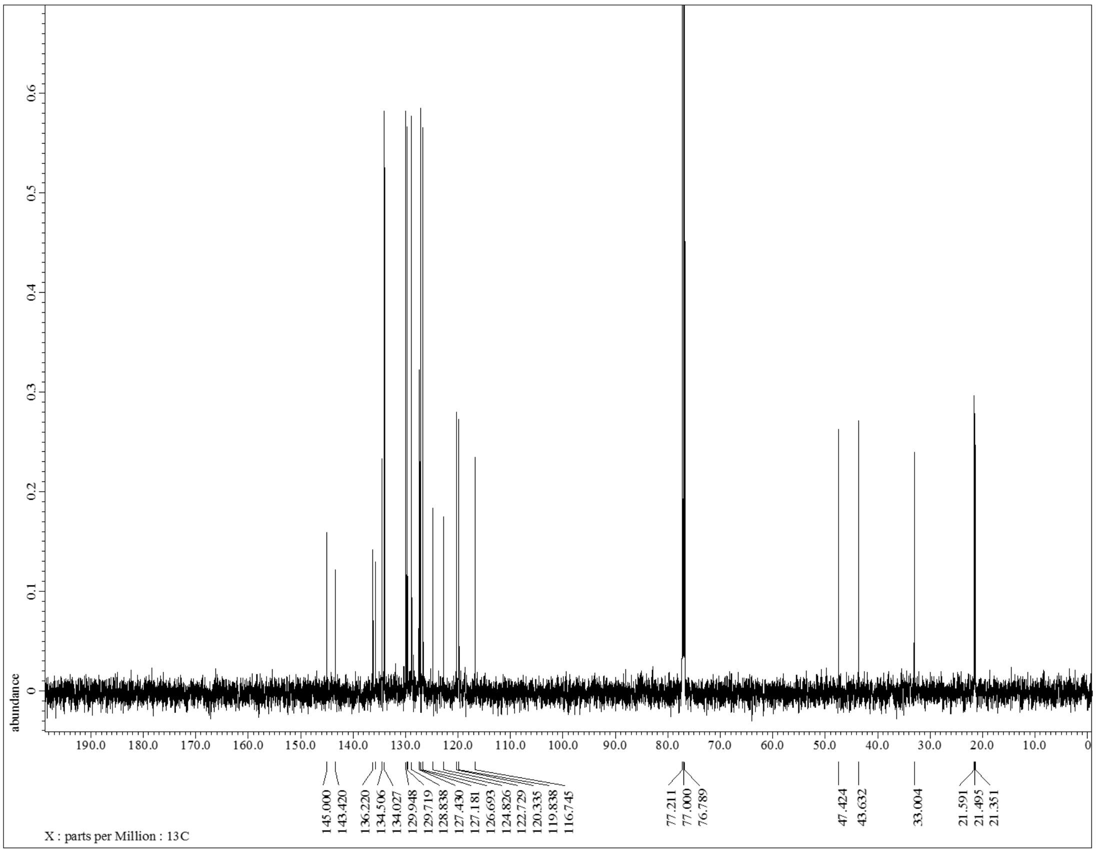
abundance
X : parts per Million : 13C

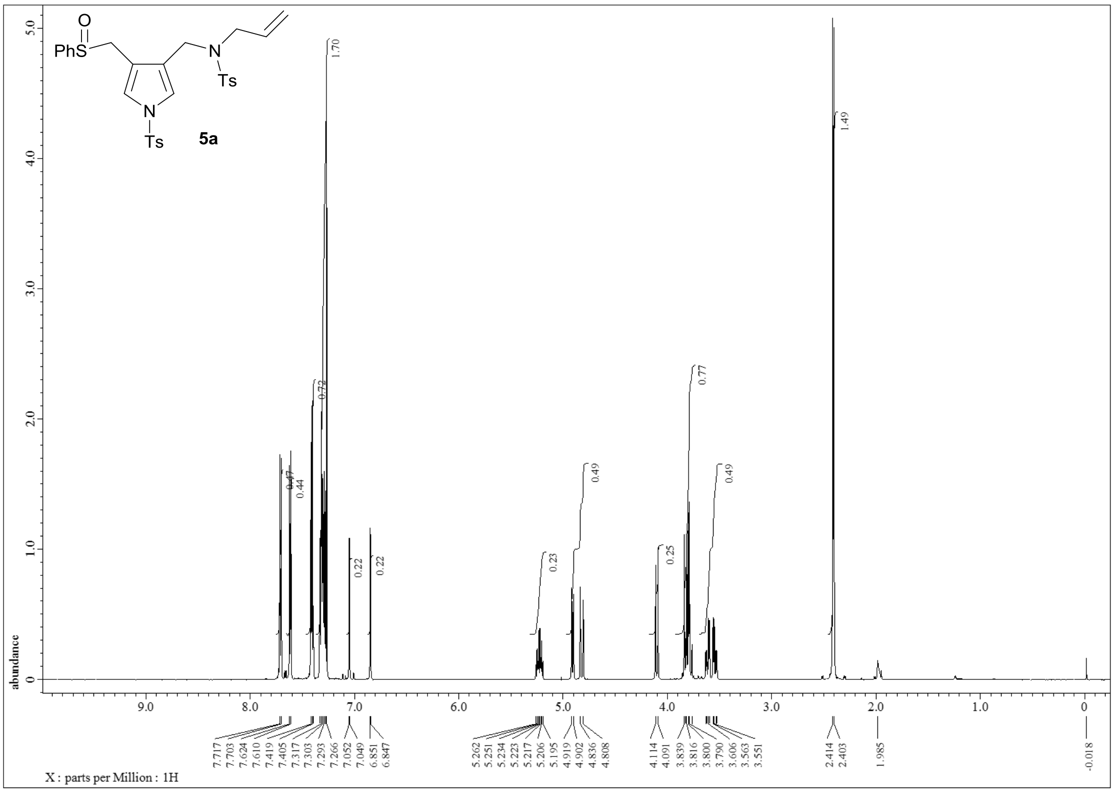
PhS
O
N
Ts
N
Ts
5a
X : parts per Million : 1H
abundance

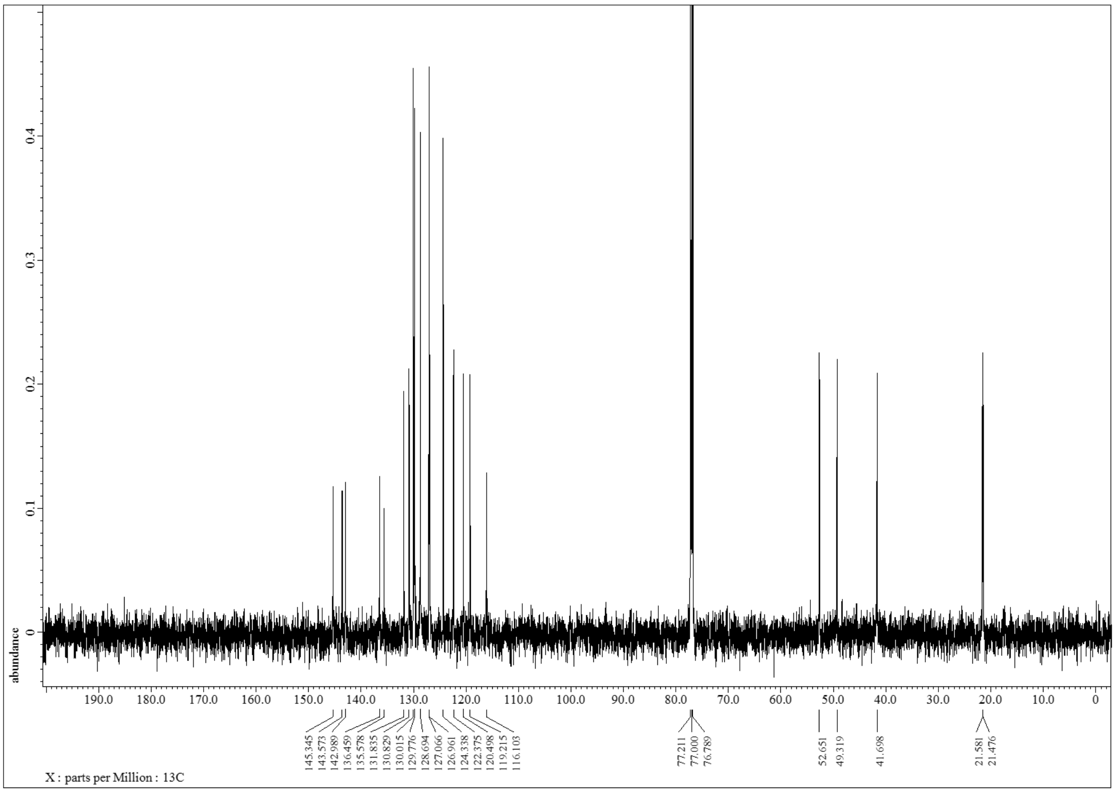
X : parts per Million : 13C
abundance

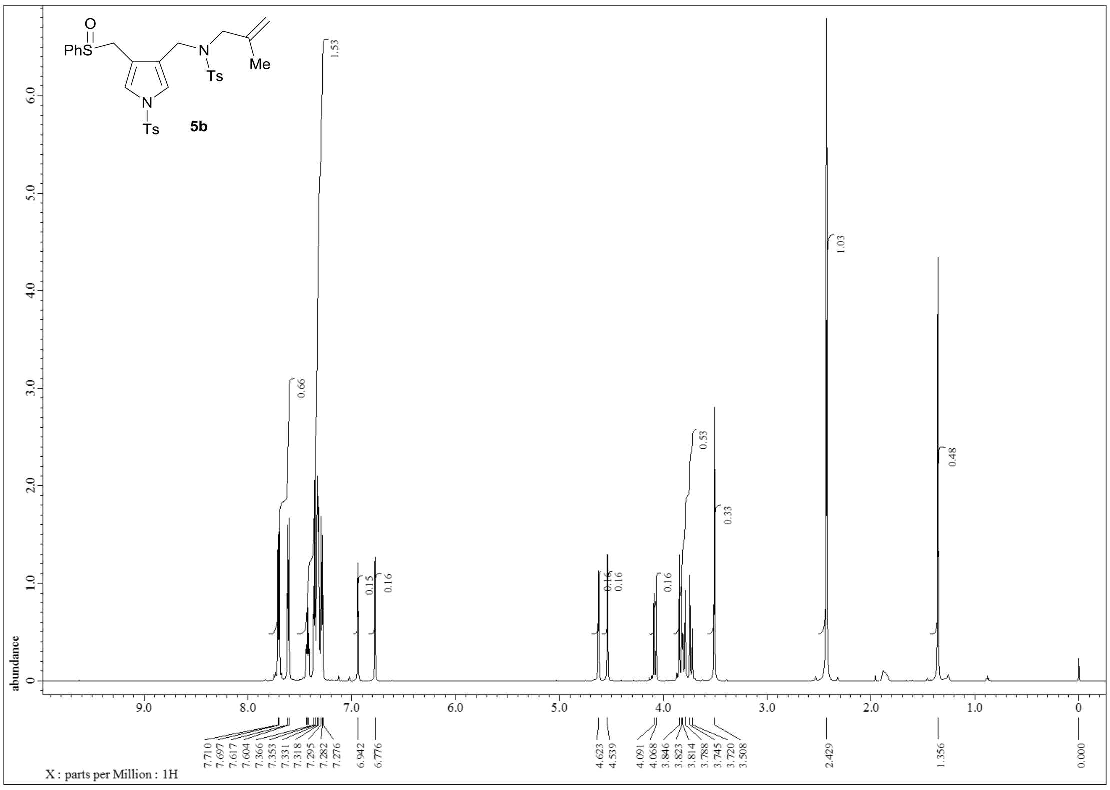
PhS
O
N
Ts
Me
N
Ts
5b
abundance
X : parts per Million : 1H

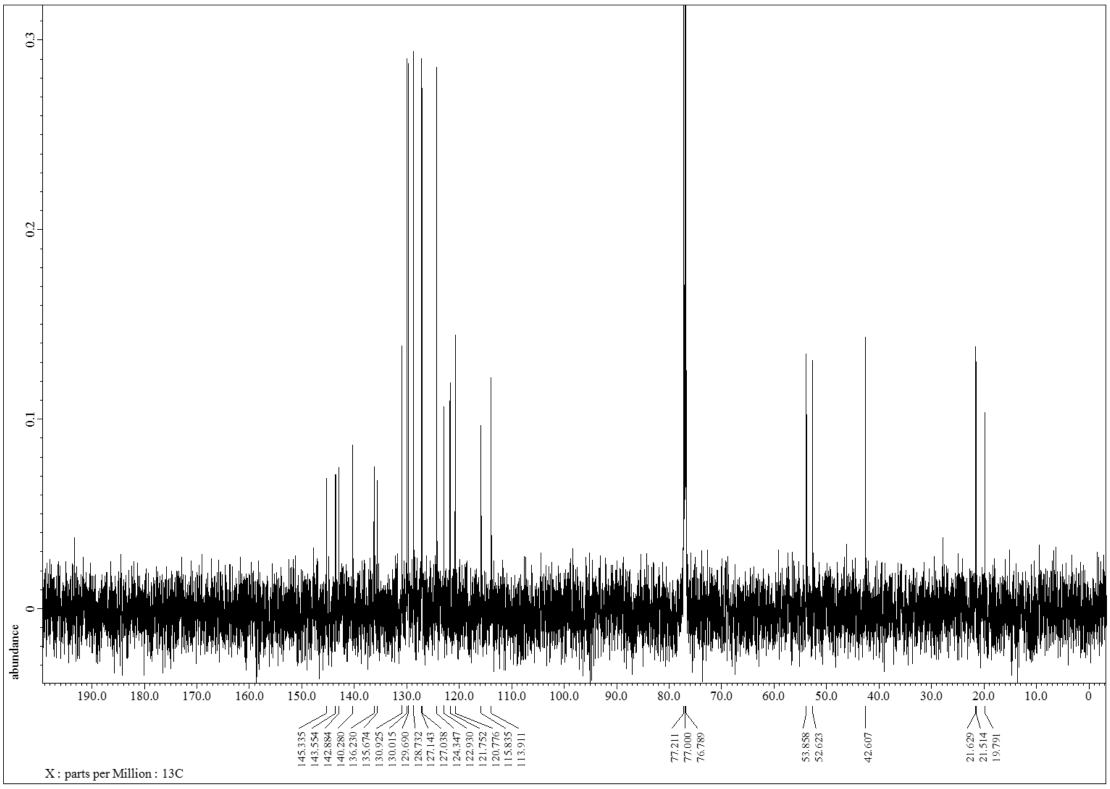
abundance
X : parts per Million : 13C

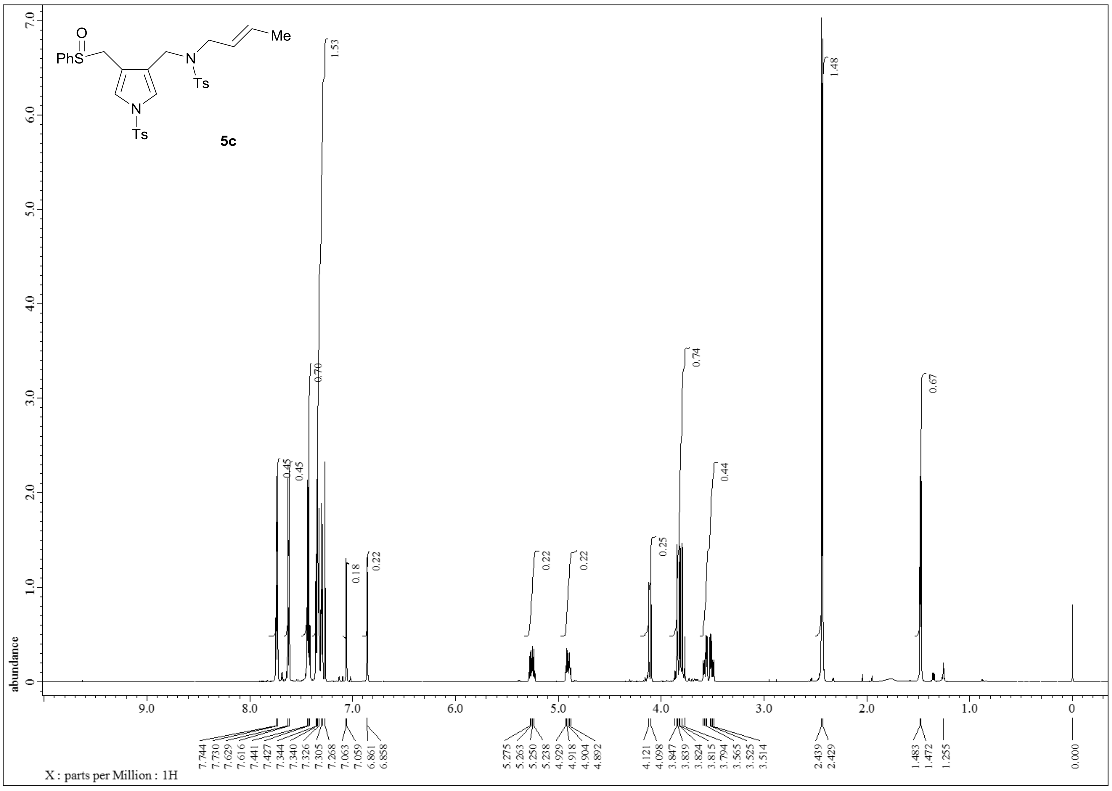
5c
X : parts per Million : 1H
abundance

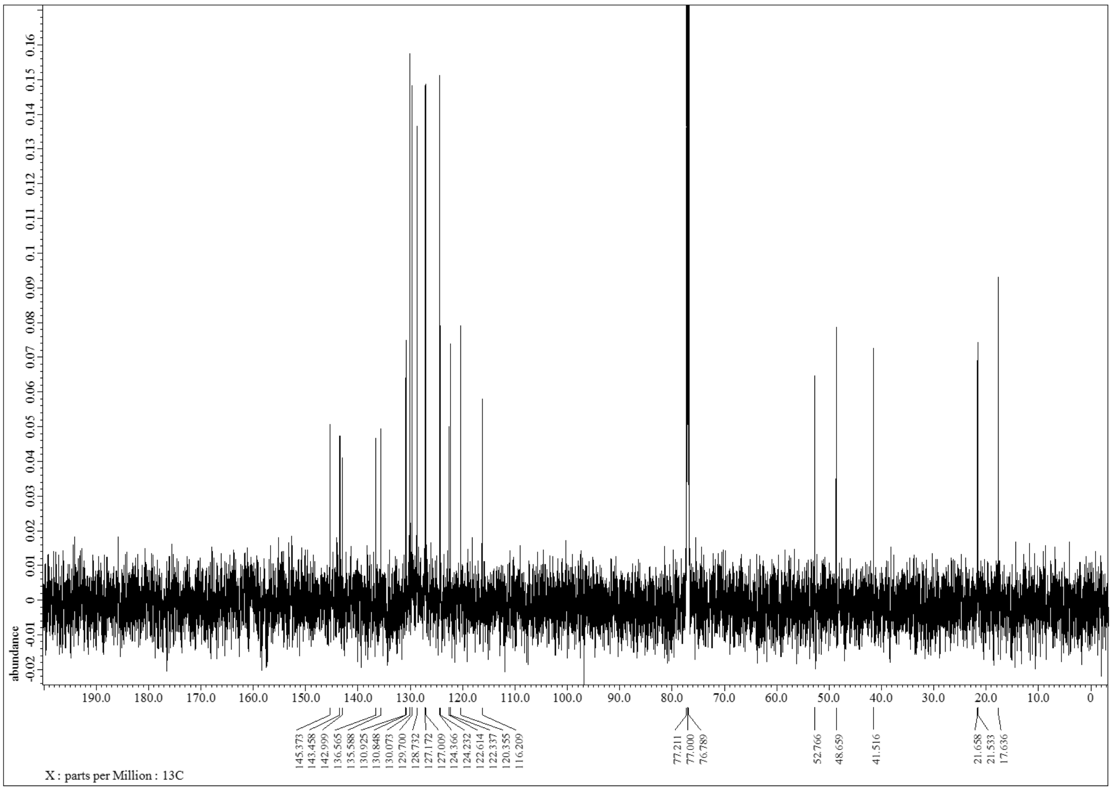
X : parts per Million : 13C
abundance

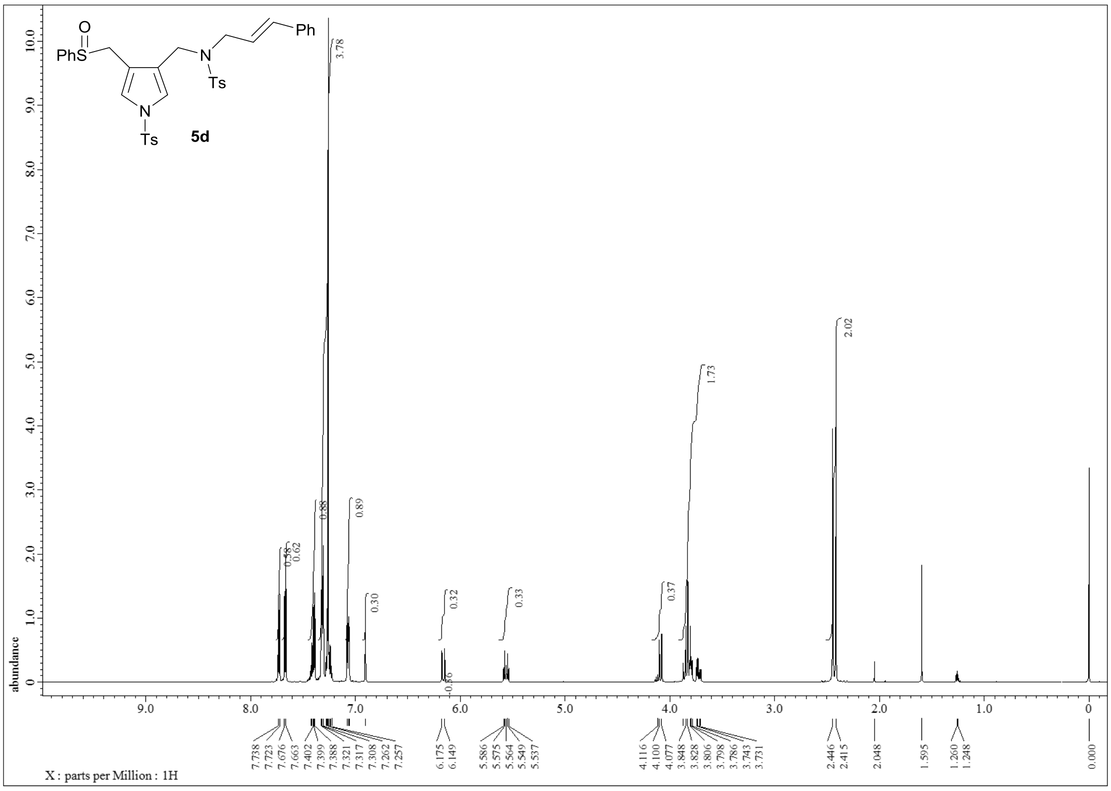
PhS
O
N
Ts
Ph
N
Ts
5d
abundance
X : parts per Million : 1H

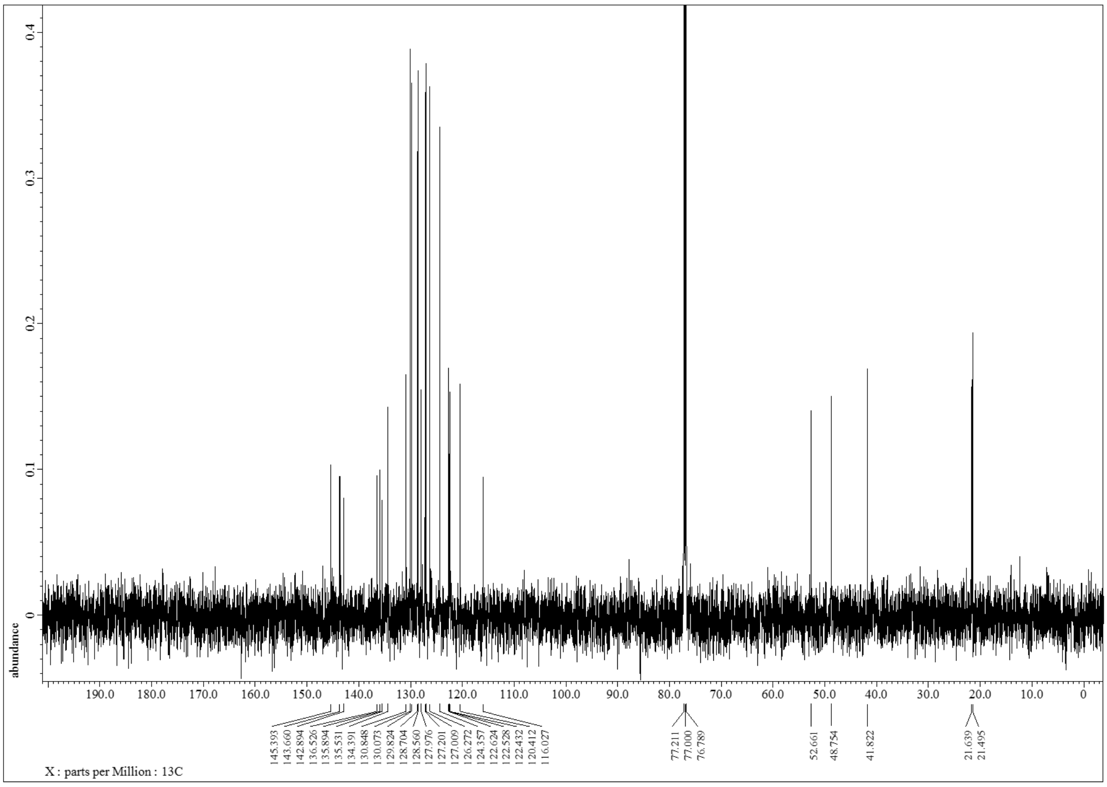
abundance
X : parts per Million : 13C

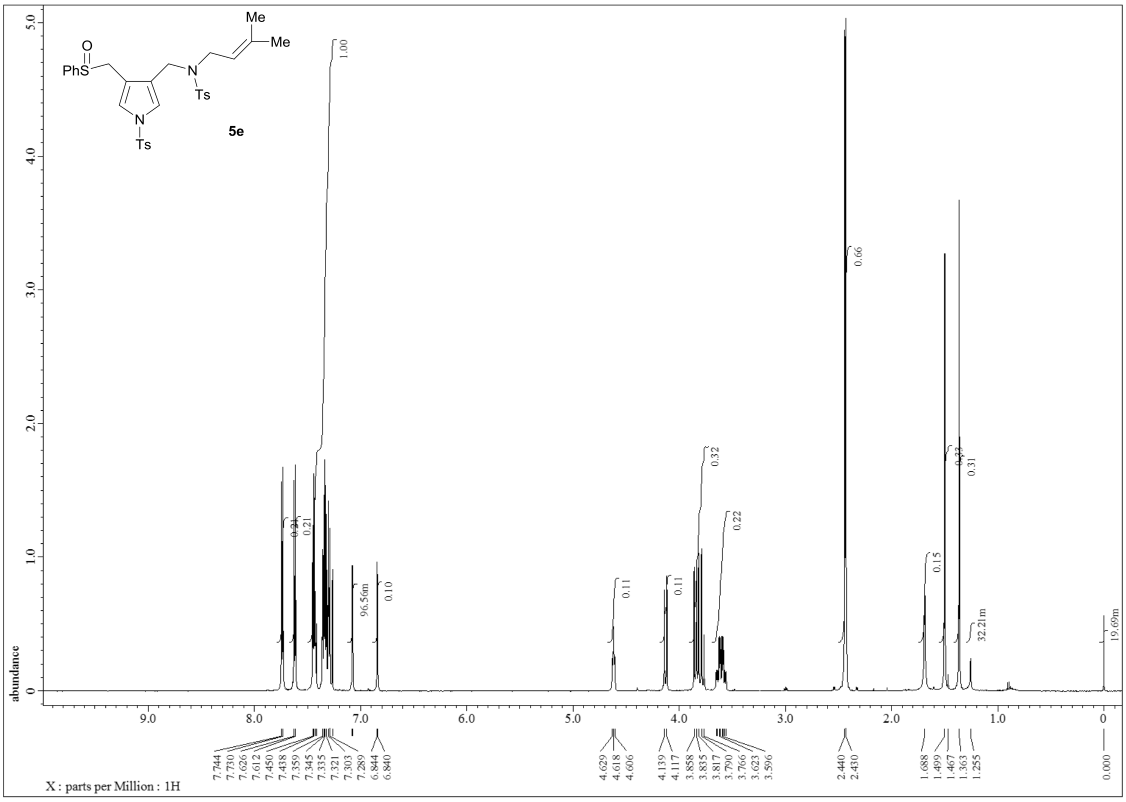
Me
Me
O
PhS
N
Ts
N
Ts
5e
abundance
X : parts per Million : 1H
7.744 7.730 7.626 7.612 7.450 7.438 7.359 7.345 7.335 7.321 7.303 7.289 6.844 6.840
4.629 4.618 4.606
4.139 4.117 3.858 3.835 3.817 3.790 3.766 3.623 3.596
2.440 2.430
1.688 1.499 1.467 1.363 1.255
0.000

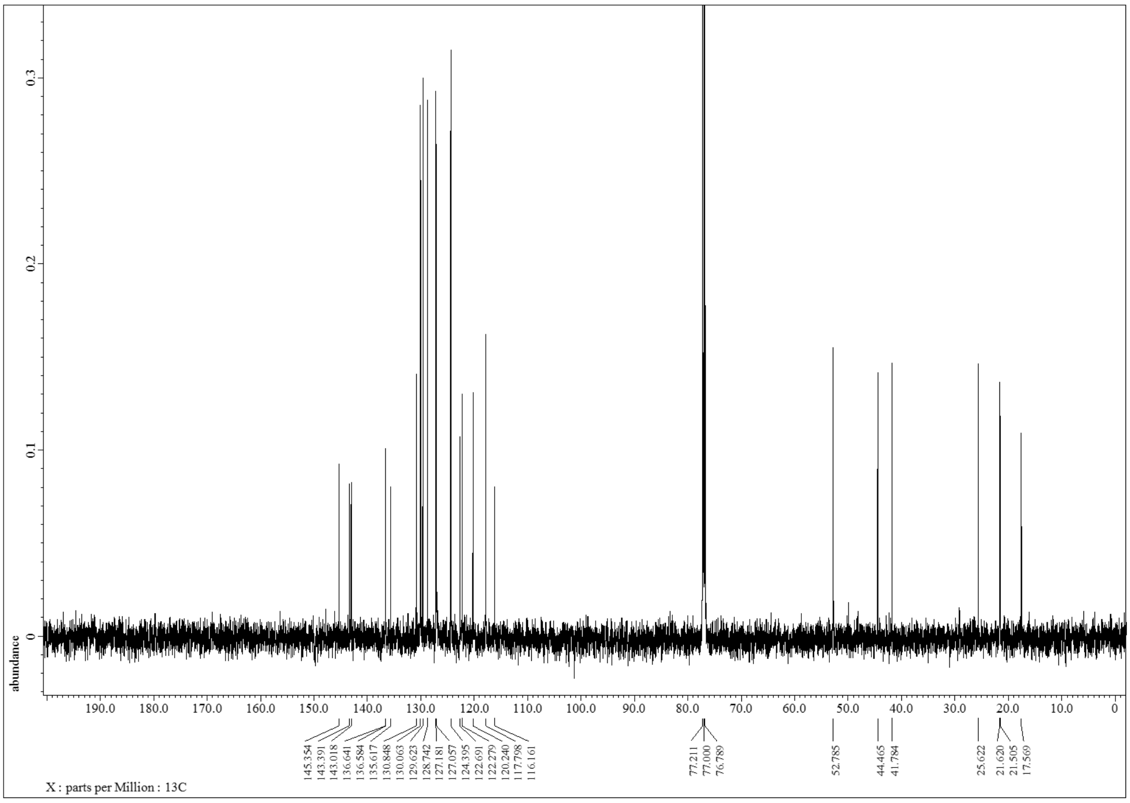
abundance
X : parts per Million : 13C
145.354 143.391 143.018 136.641 136.584 135.617 130.848 130.063 129.623 128.742 127.181 127.057 124.395 122.691 122.279 120.240 117.798 116.161
77.211 77.000 76.789
52.785
44.465 41.784
25.622 21.620 21.505 17.569

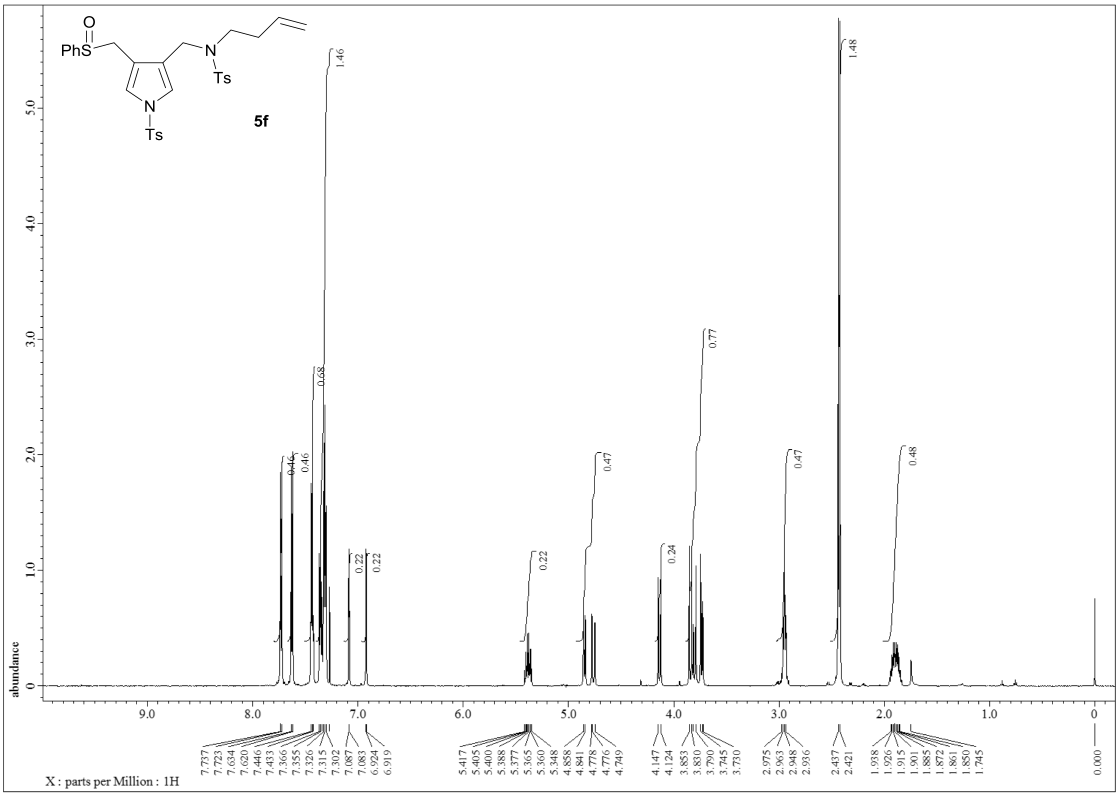
PhS
O
N
Ts
N
Ts
5f
abundance
X : parts per Million : 1H
1.46
0.68
0.46
0.46
0.22
0.22
0.22
0.47
0.24
0.77
0.47
1.48
0.48
7.737 7.723 7.634 7.620 7.446 7.433 7.366 7.355 7.326 7.315 7.302 7.087 7.083 6.924 6.919
5.417 5.405 5.400 5.388 5.377 5.365 5.360 5.348 4.858 4.841 4.778 4.776 4.749
4.147 4.124 3.853 3.830 3.790 3.745 3.730
2.975 2.963 2.948 2.936
2.437 2.421
1.938 1.926 1.915 1.901 1.885 1.872 1.861 1.850 1.745
0.000

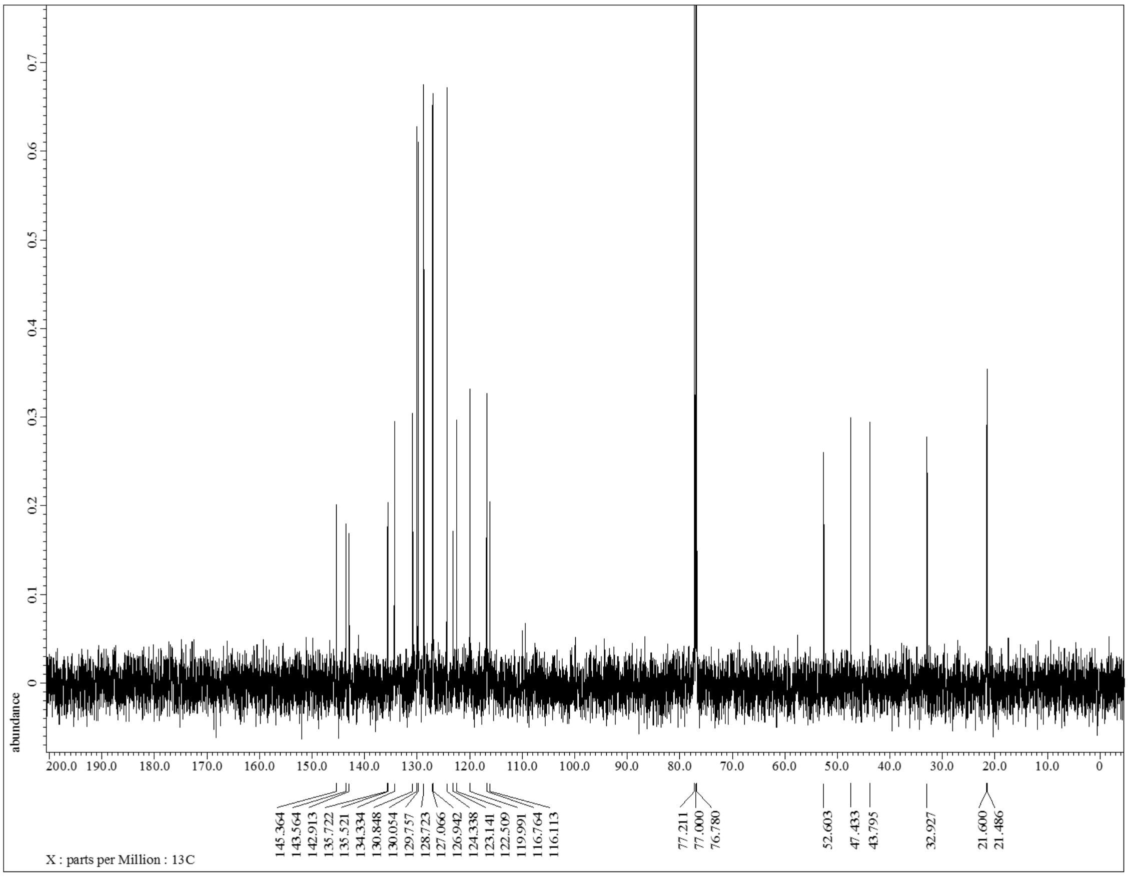
abundance
X : parts per Million : 13C
145.364 143.564 142.913 135.722 135.521 134.334 130.848 130.054 129.757 128.723 127.066 126.942 124.338 123.141 122.509 119.991 116.764 116.113
77.211 77.000 76.780
52.603
47.433
43.795
32.927
21.600 21.486

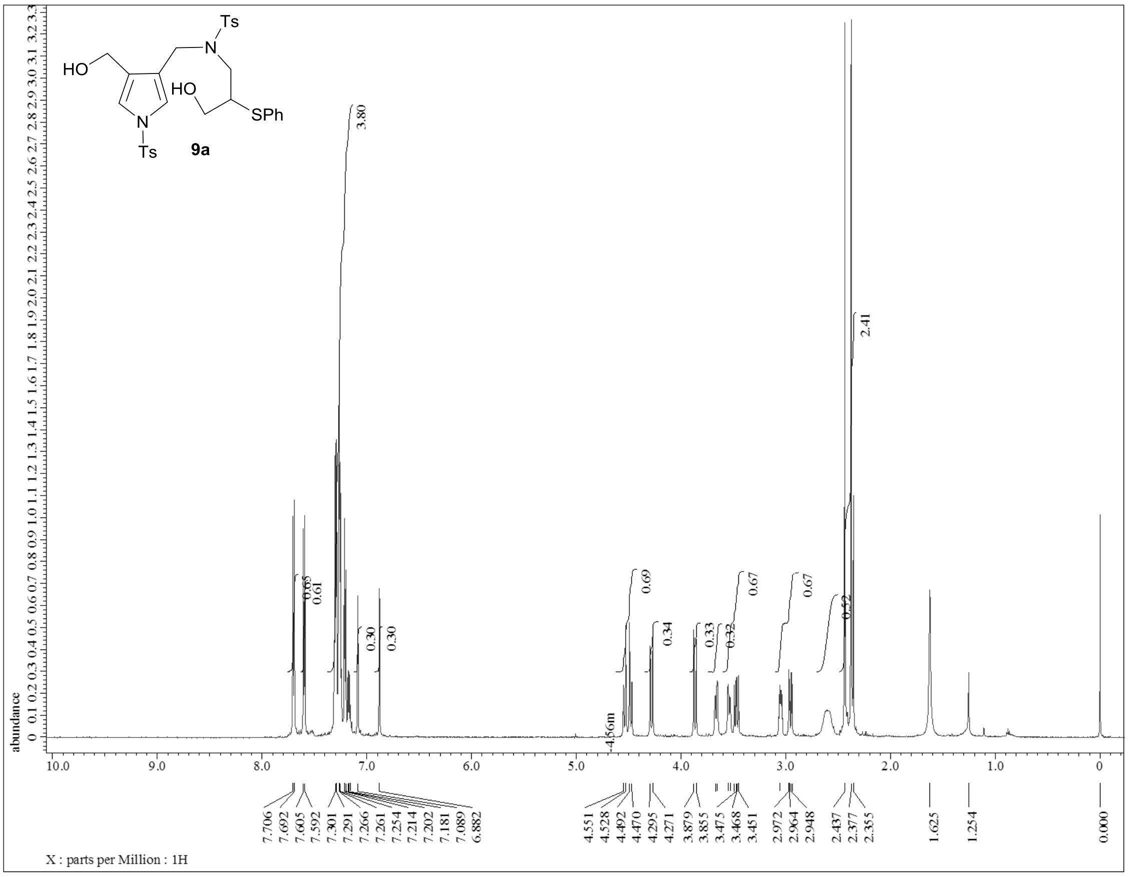
HO
Ts
N
HO
SPh
N
Ts
9a
abundance
3.80
0.65
0.61
0.30
0.30
0.69
0.34
0.33
0.32
0.67
0.67
0.52
2.41
4.56m
7.706
7.692
7.605
7.592
7.301
7.291
7.266
7.261
7.254
7.214
7.202
7.181
7.089
6.882
4.551
4.528
4.492
4.470
4.295
4.271
3.879
3.855
3.475
3.468
3.451
2.972
2.964
2.948
2.437
2.377
2.355
1.625
1.254
0.000
X : parts per Million : 1H

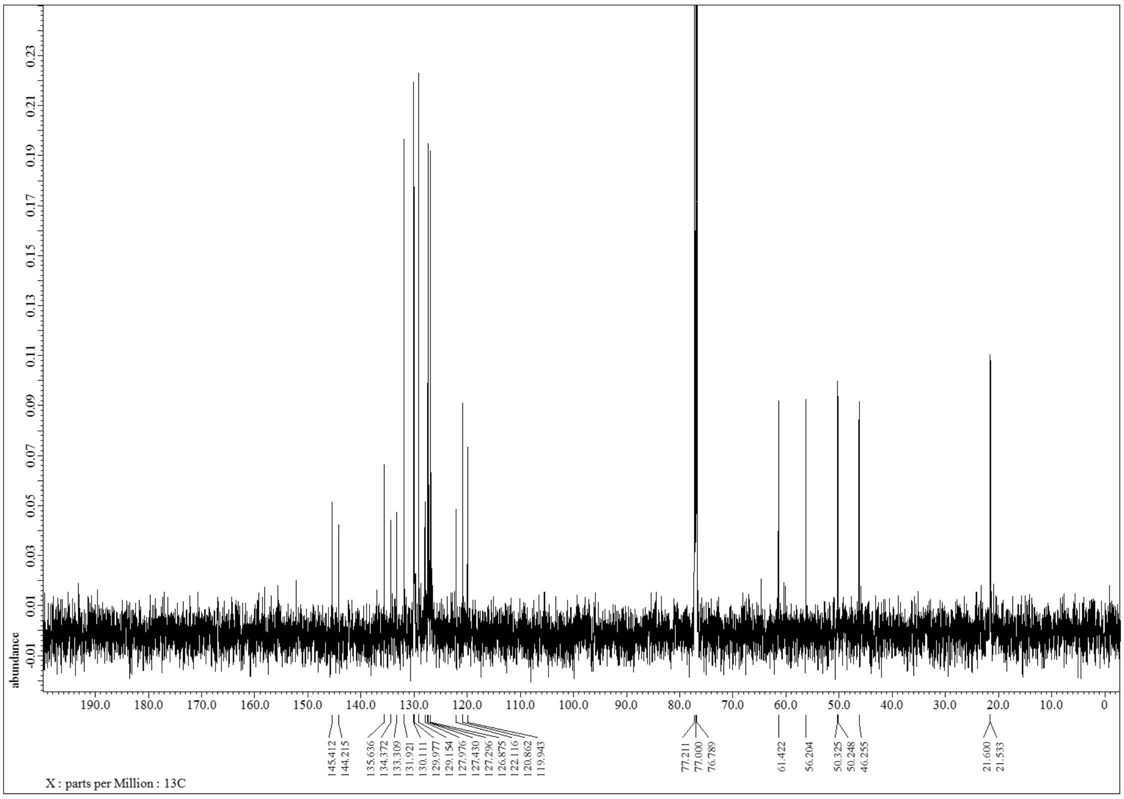
abundance
145.412
144.215
135.636
134.372
133.309
131.921
130.111
129.977
129.154
127.976
127.430
127.296
126.875
122.116
120.862
119.943
77.211
77.000
76.789
61.422
56.204
50.325
50.248
46.255
21.600
21.533
X : parts per Million : 13C

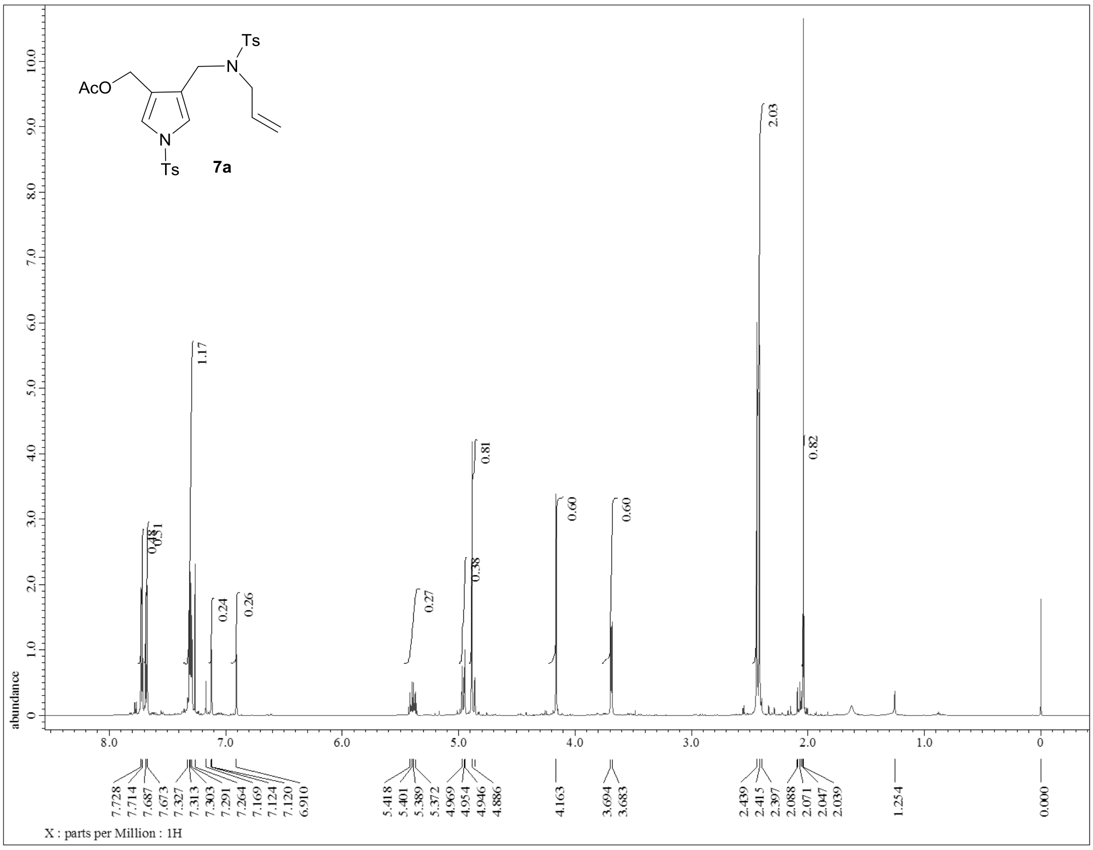
AcO
N
Ts
Ts
N
7a
1.17
0.78
0.51
0.24
0.26
0.27
0.38
0.81
0.60
0.60
2.03
0.82
abundance
7.728
7.714
7.687
7.673
7.327
7.313
7.303
7.291
7.264
7.169
7.124
7.120
6.910
5.418
5.401
5.389
5.372
4.969
4.954
4.946
4.886
4.163
3.694
3.683
2.439
2.415
2.397
2.088
2.071
2.047
2.039
1.254
0.000
X : parts per Million : 1H

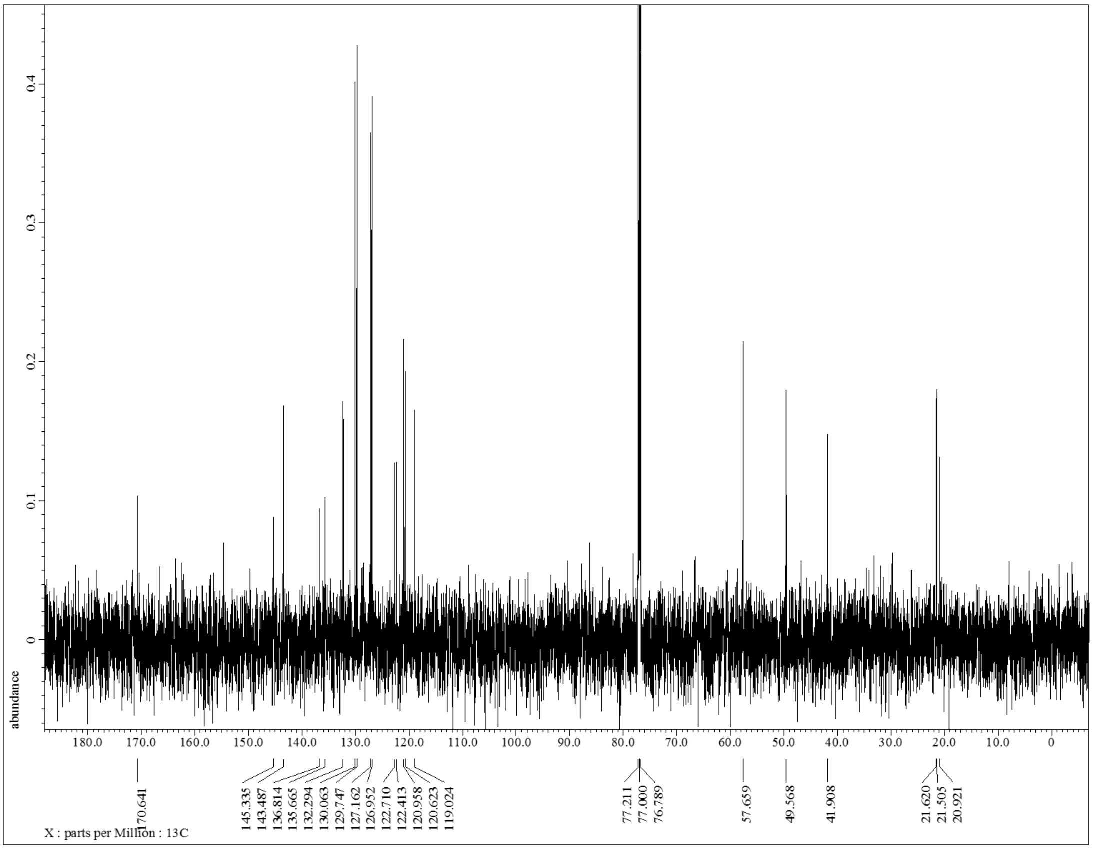
abundance
170.641
145.335
143.487
136.814
135.665
132.294
130.063
129.747
127.162
126.952
122.710
122.413
120.958
120.623
119.024
77.211
77.000
76.789
57.659
49.568
41.908
21.620
21.505
20.921
X : parts per Million : 13C

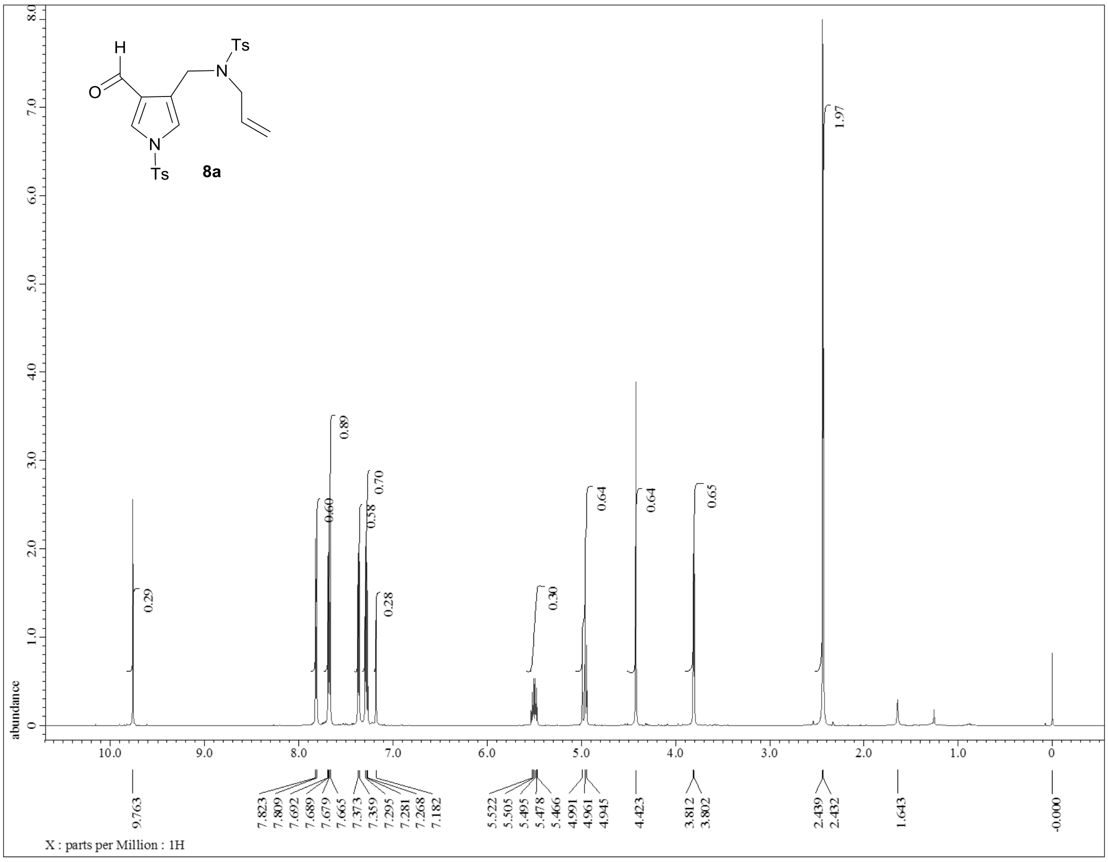
Ts
H
N
O
N
Ts
8a
0.29
0.60
0.89
0.58
0.70
0.28
0.30
0.64
0.64
0.65
1.97
abundance
9.763
7.823
7.809
7.692
7.689
7.679
7.665
7.373
7.359
7.295
7.281
7.268
7.182
5.522
5.505
5.495
5.478
5.466
4.991
4.961
4.945
4.423
3.812
3.802
2.439
2.432
1.643
-0.000
X : parts per Million : 1H

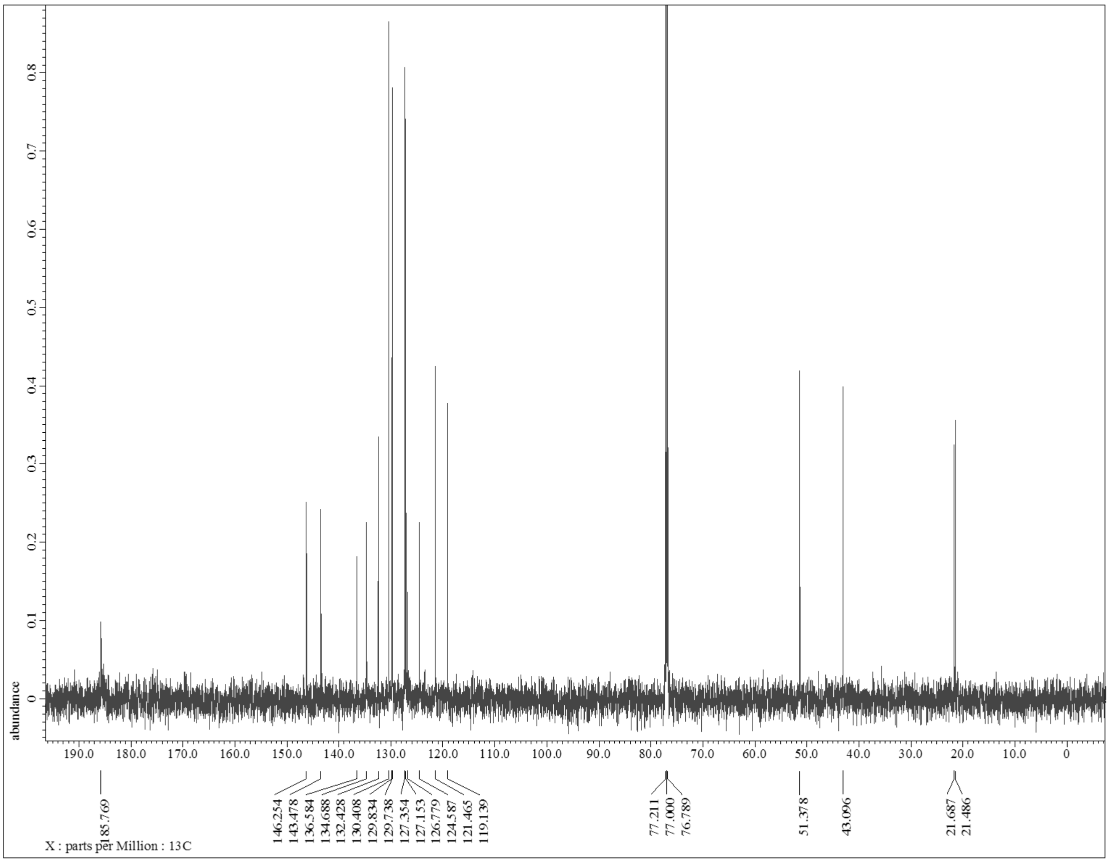
abundance
185.769
146.254
143.478
136.584
134.688
132.428
130.408
129.834
129.738
127.354
127.153
126.779
124.587
121.465
119.139
77.211
77.000
76.789
51.378
43.096
21.687
21.486
X : parts per Million : 13C

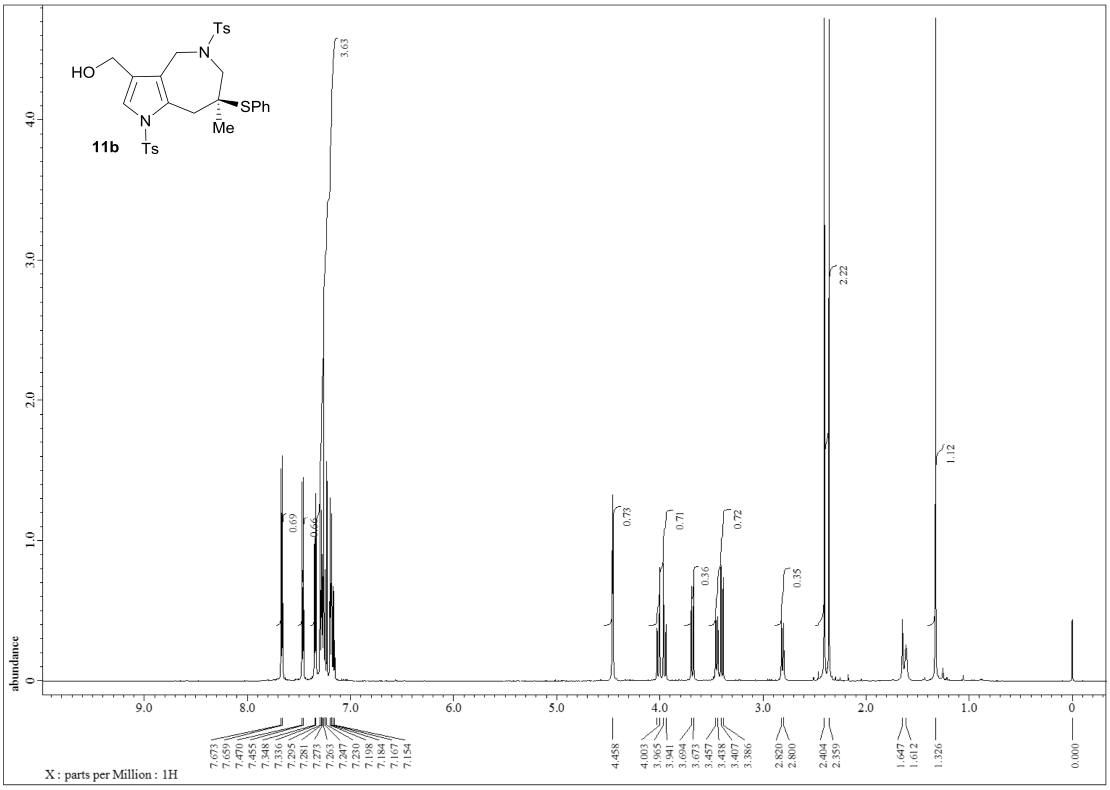
HO
Ts
N
SPh
Me
N
Ts
11b
abundance
X : parts per Million : 1H
3.63
0.69
0.66
0.73
0.71
0.36
0.72
0.35
2.22
1.12
7.673 7.659 7.470 7.455 7.348 7.336 7.295 7.281 7.273 7.263 7.247 7.230 7.198 7.184 7.167 7.154
4.458
4.003 3.965 3.941 3.694 3.673 3.457 3.438 3.407 3.386
2.820 2.800
2.404 2.359
1.647 1.612
1.326
0.000

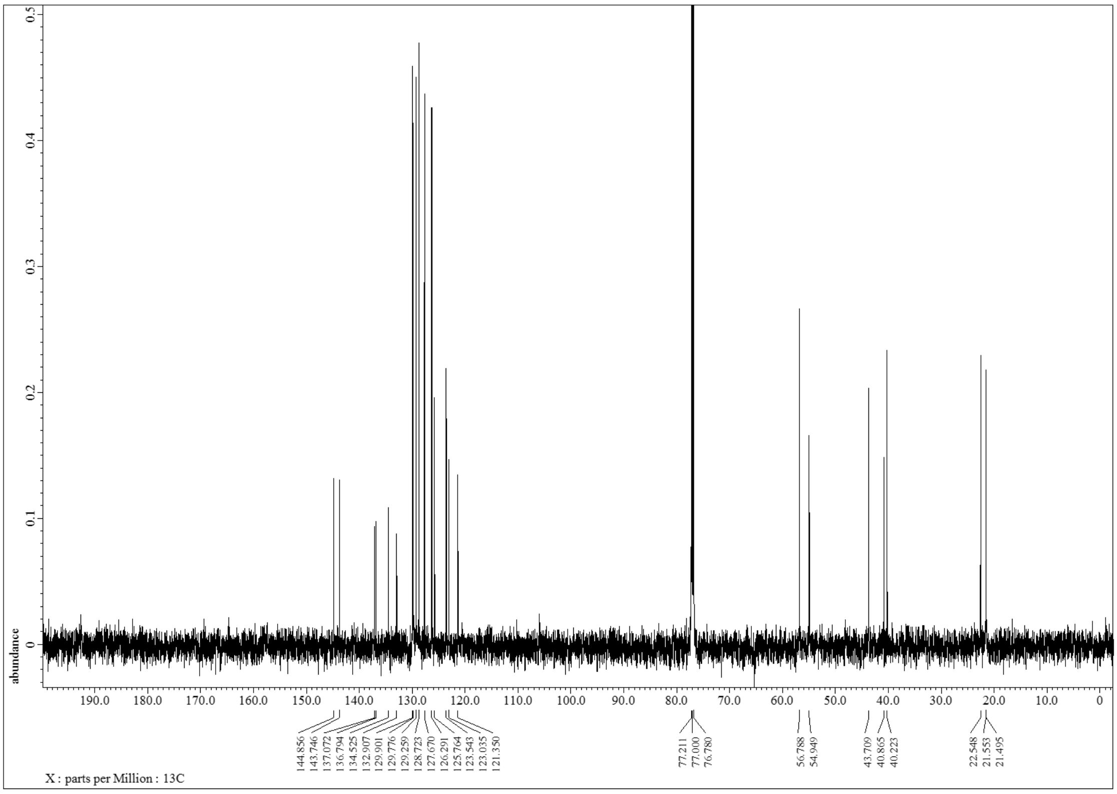
abundance
X : parts per Million : 13C
144.856 143.746 137.072 136.794 134.525 132.907 129.901 129.776 129.259 128.723 127.670 126.291 125.764 123.543 123.035 121.350
77.211 77.000 76.780
56.788 54.949
43.709 40.865 40.223
22.548 21.553 21.495

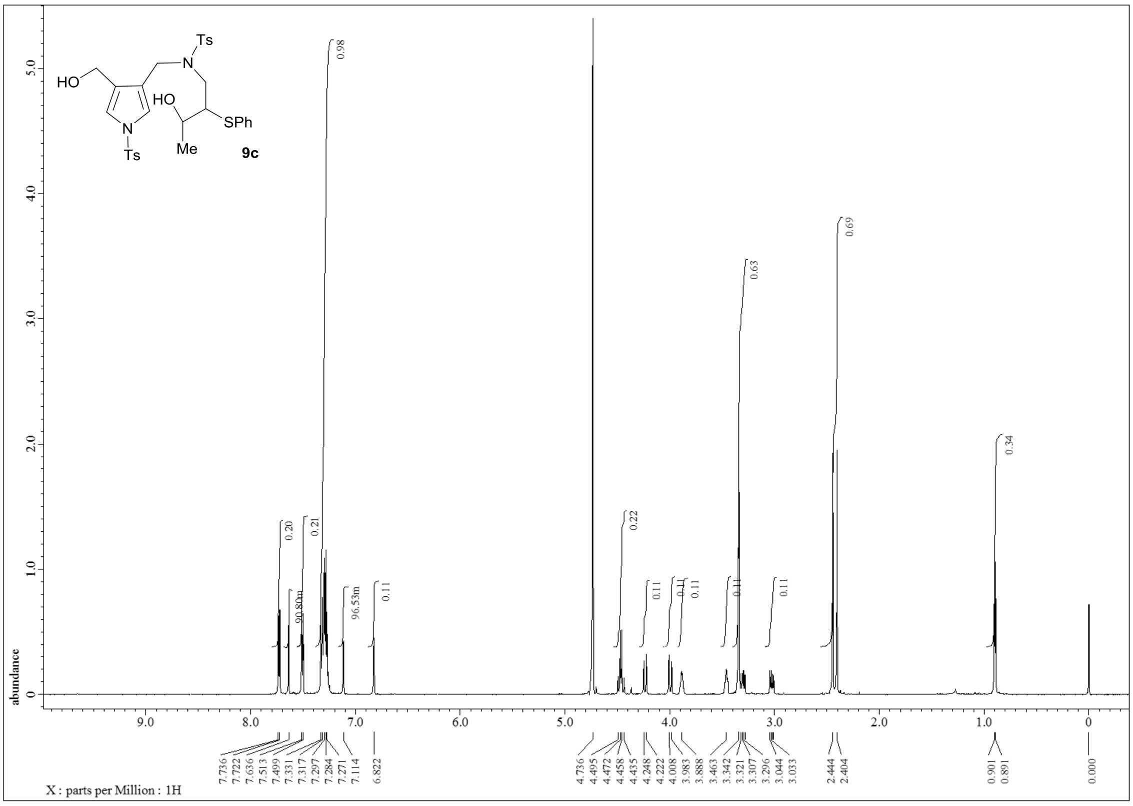
HO
Ts
N
HO
SPh
N
Ts
Me
9c
X : parts per Million : 1H
abundance

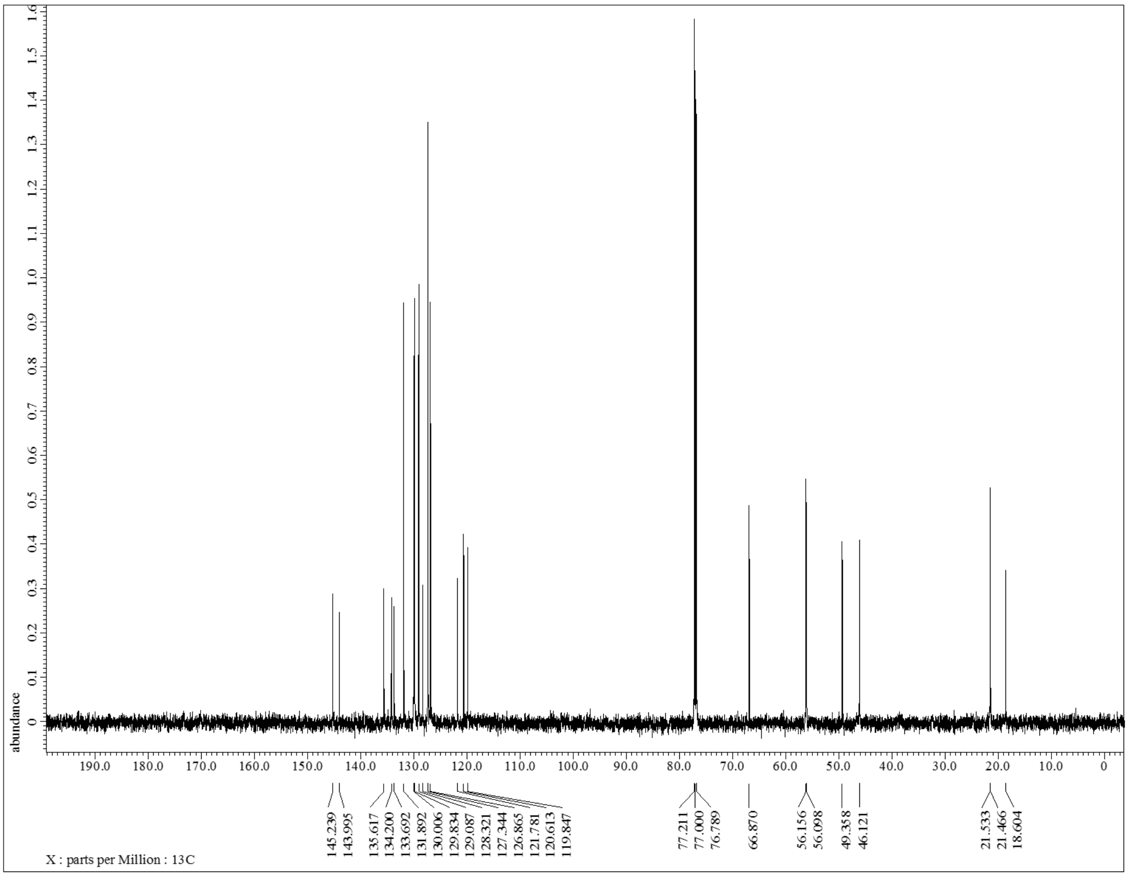
X : parts per Million : 13C
abundance

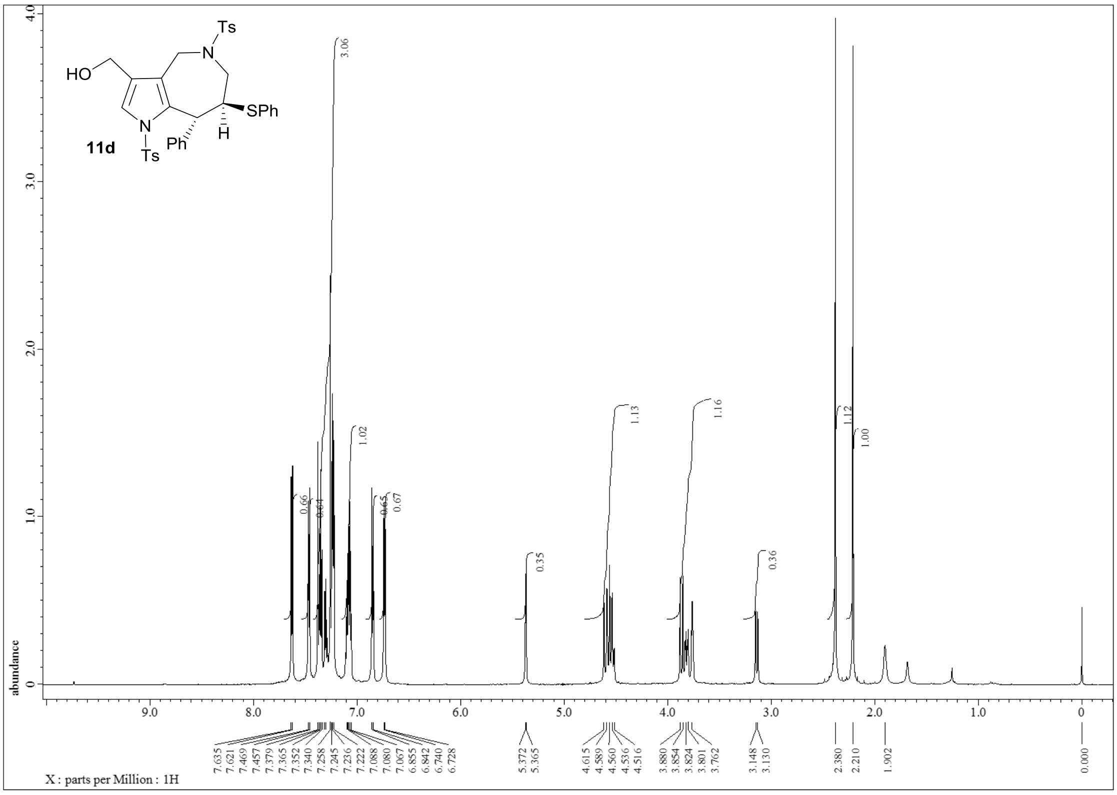
11d
X : parts per Million : 1H
abundance

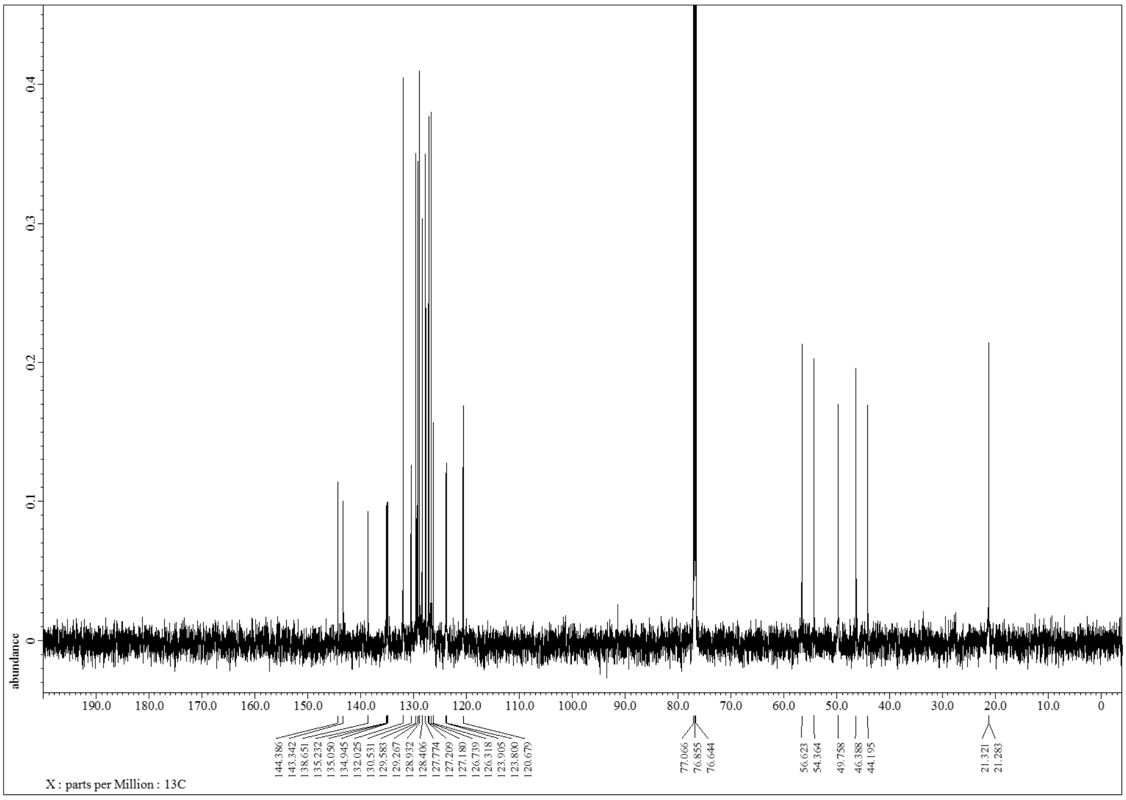
X : parts per Million : 13C
abundance

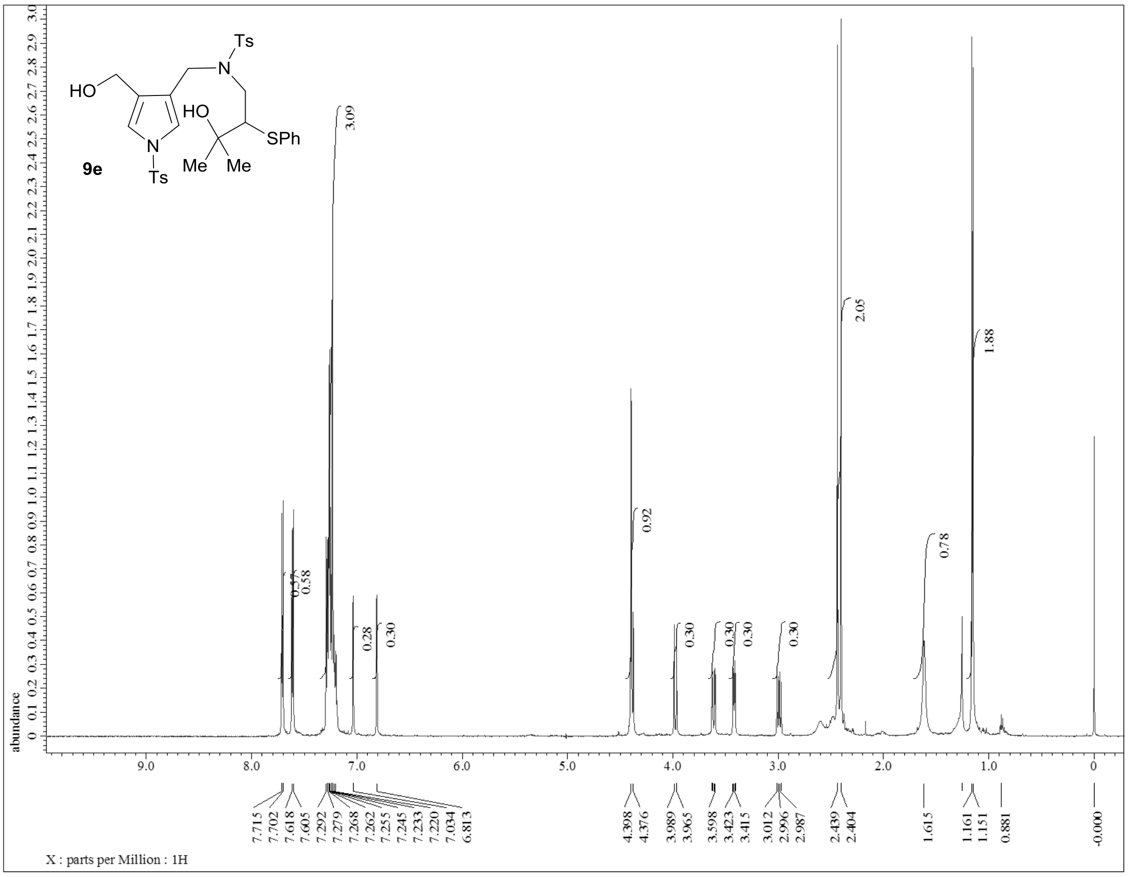

9e
HO
Ts
N
HO
SPh
Me
Me
abundance
3.09
0.58
0.28
0.30
0.92
0.30
0.30
0.30
0.30
2.05
0.78
1.88
7.715
7.702
7.618
7.605
7.292
7.279
7.268
7.262
7.255
7.245
7.233
7.220
7.034
6.813
4.398
4.376
3.989
3.965
3.598
3.423
3.415
3.012
2.996
2.987
2.439
2.404
1.615
1.161
1.151
0.881
-0.000
X : parts per Million : 1H

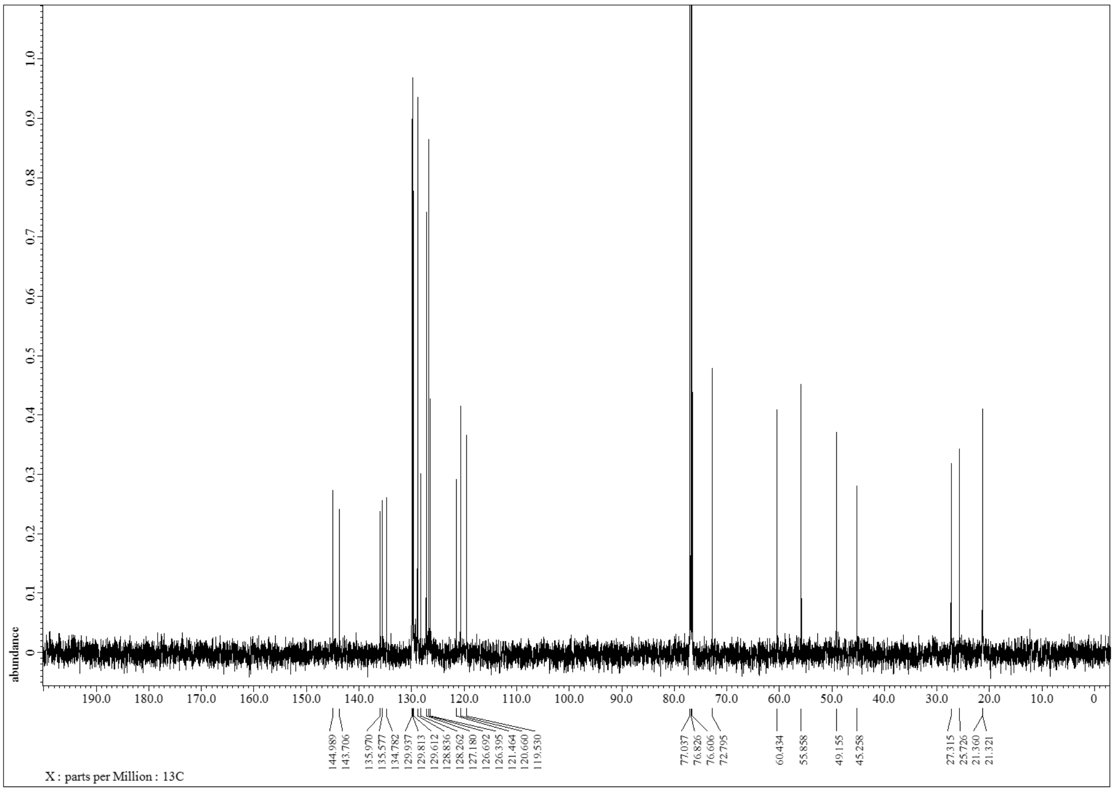

abundance
144.989
143.706
135.970
135.577
134.782
129.937
129.813
129.612
128.836
128.262
127.180
126.692
126.395
121.464
120.660
119.530
77.037
76.826
76.606
72.795
60.434
55.858
49.155
45.258
27.315
25.726
21.360
21.321
X : parts per Million : 13C

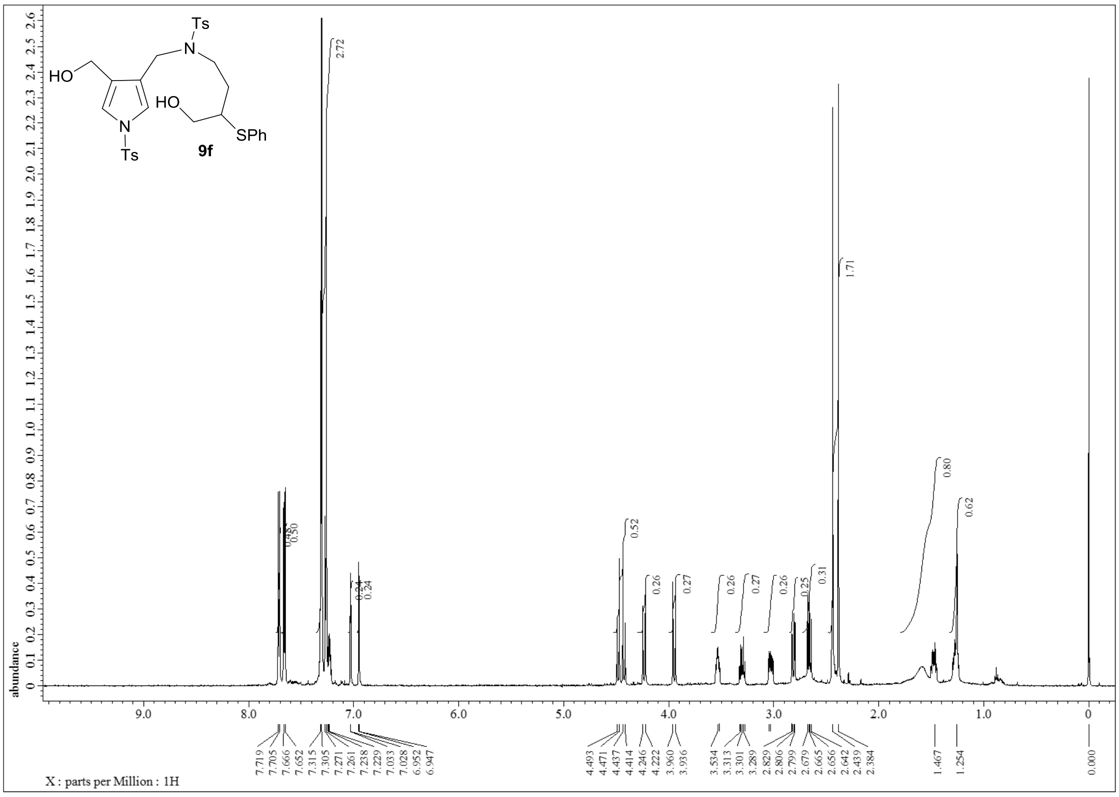
Ts
N
HO
HO
SPh
N
Ts
9f
abundance
X : parts per Million : 1H

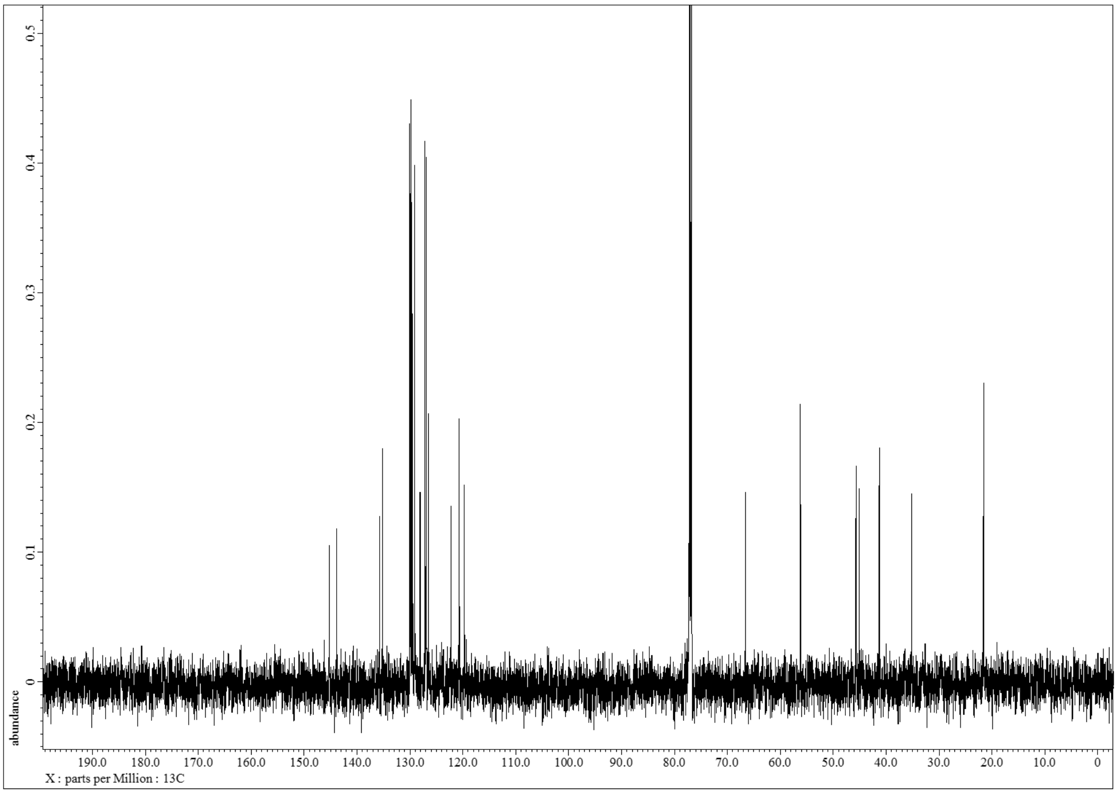
abundance
X : parts per Million : 13C

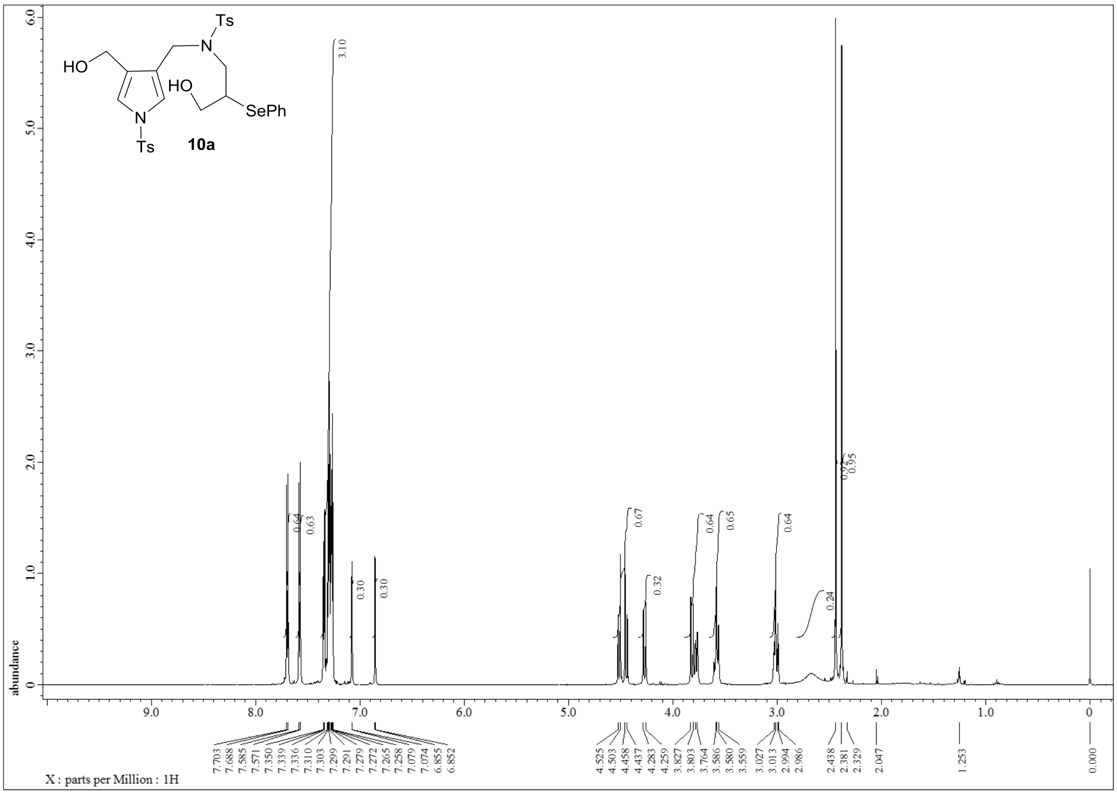

HO
Ts
N
HO
SePh
N
Ts
10a
X : parts per Million : 1H
abundance

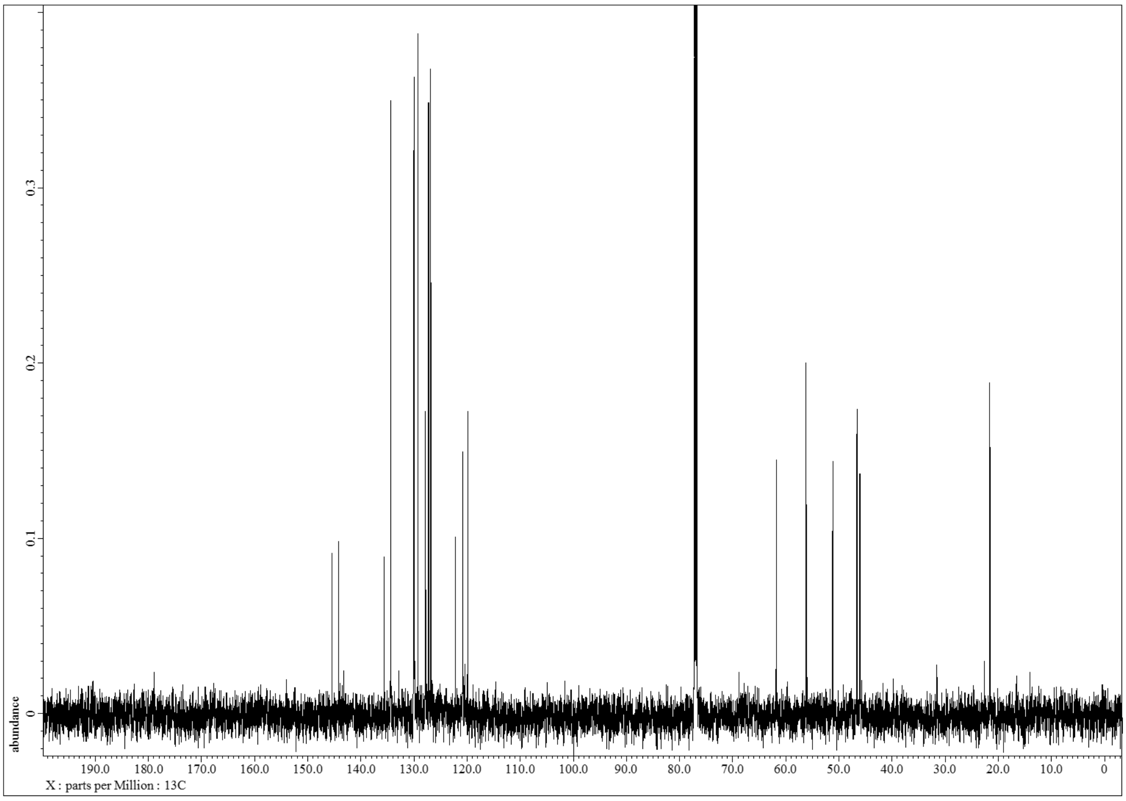

X : parts per Million : 13C
abundance

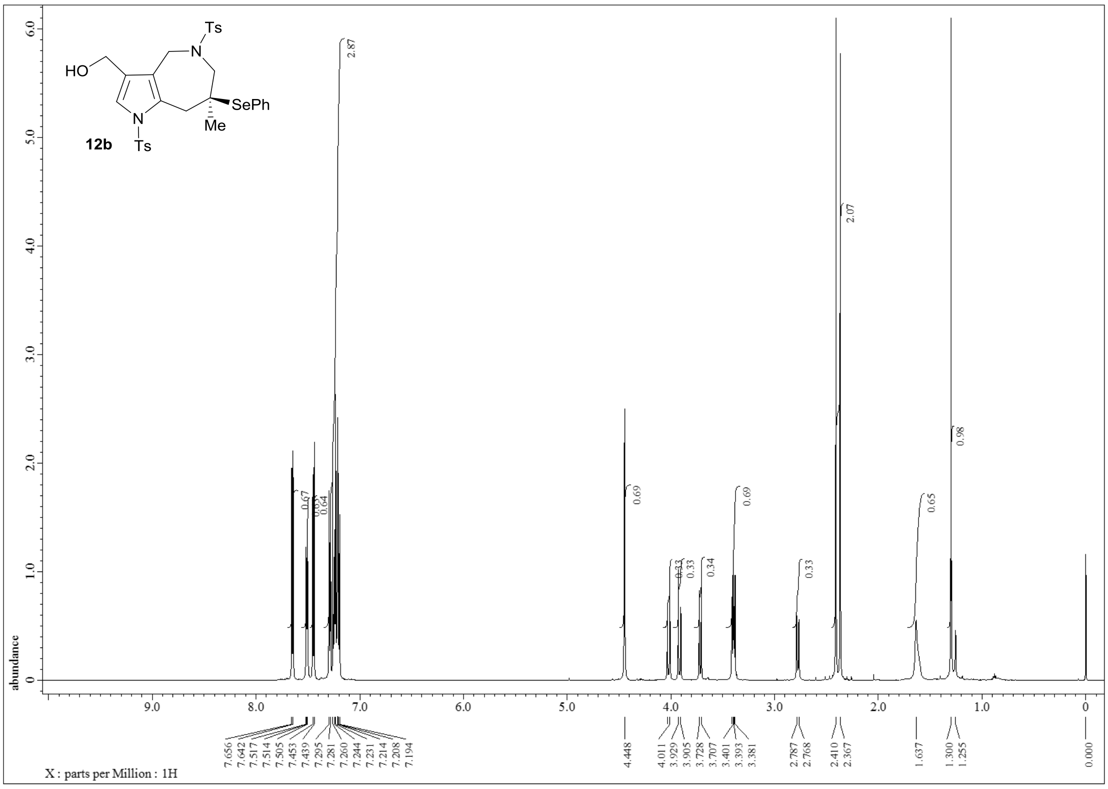
HO
Ts
N
SePh
Me
N
Ts
12b
abundance
7.656
7.642
7.517
7.514
7.505
7.453
7.439
7.295
7.281
7.260
7.244
7.231
7.214
7.208
7.194
4.448
4.011
3.929
3.905
3.728
3.707
3.401
3.393
3.381
2.787
2.768
2.410
2.367
1.637
1.300
1.255
0.000
X : parts per Million : 1H

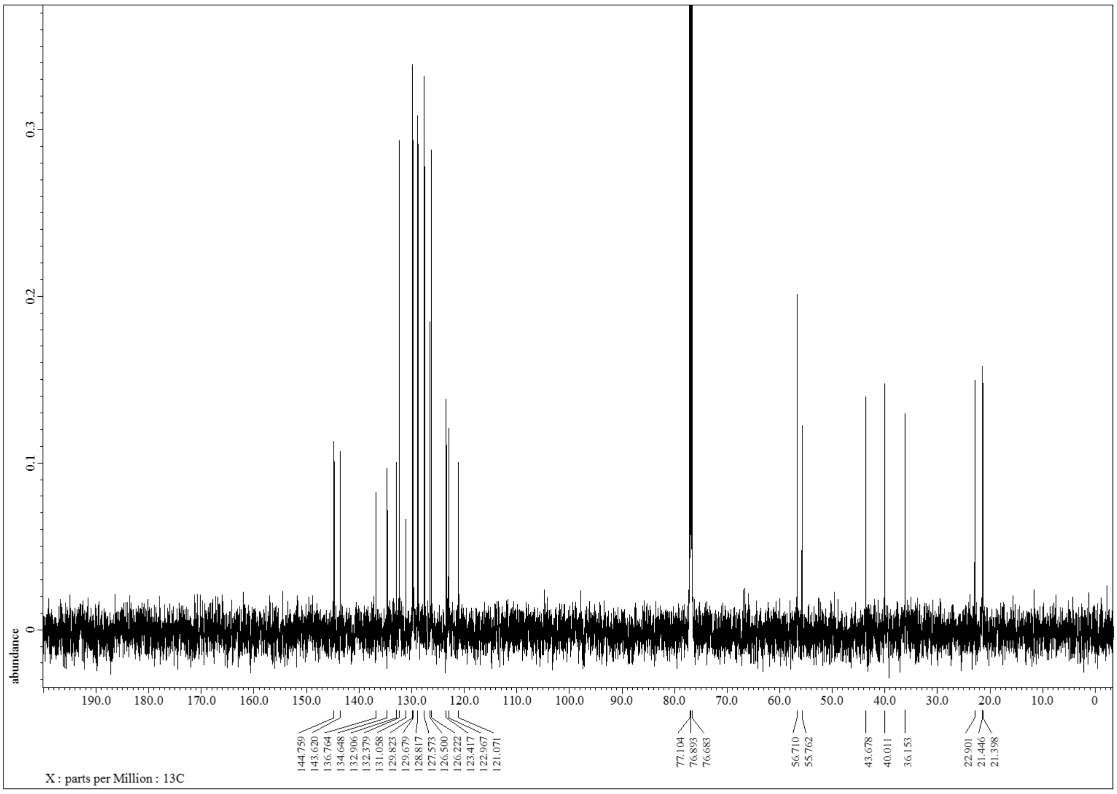
abundance
144.759
143.620
136.764
134.648
132.906
132.379
131.058
129.823
129.679
128.817
127.573
126.500
126.222
123.417
122.967
121.071
77.104
76.893
76.683
56.710
55.762
43.678
40.011
36.153
22.901
21.446
21.398
X : parts per Million : 13C

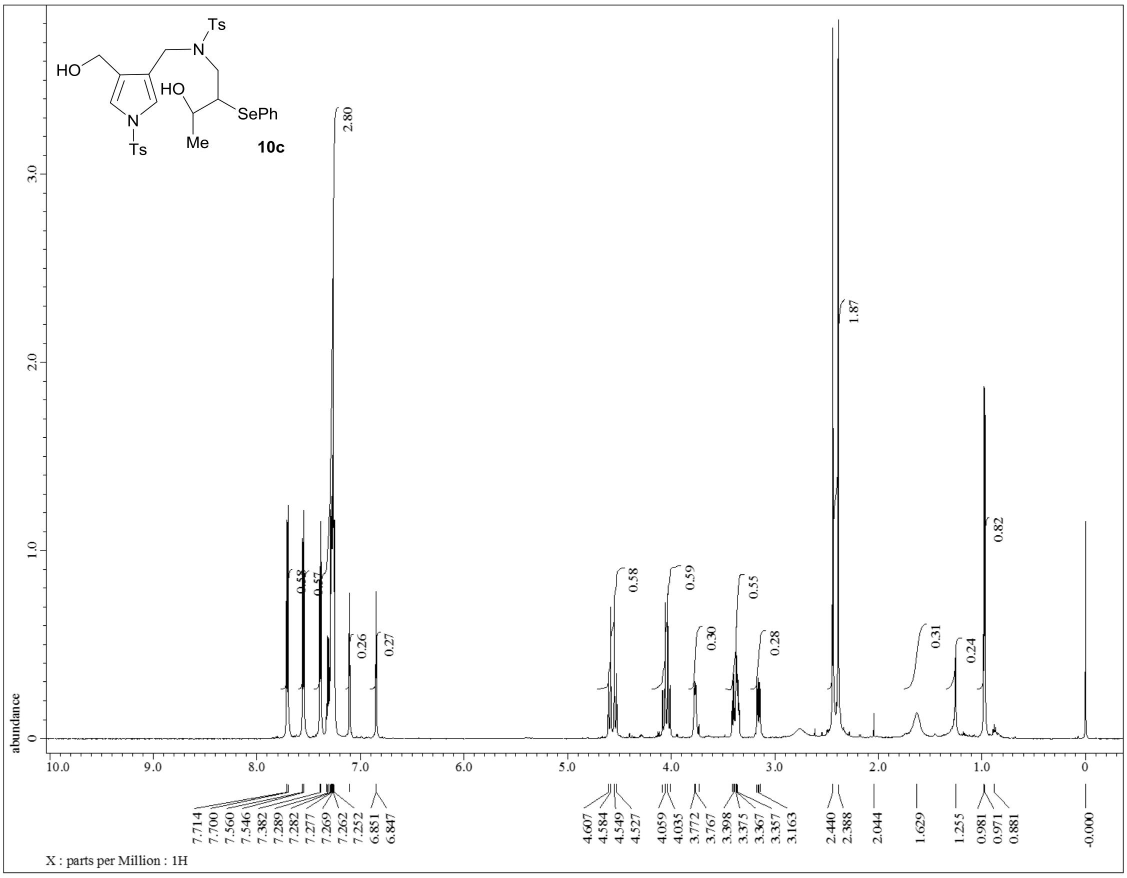
HO
N
Ts
HO
SePh
Me
Ts
10c
abundance
X : parts per Million : 1H
7.714
7.700
7.560
7.546
7.382
7.289
7.282
7.277
7.269
7.262
7.252
6.851
6.847
4.607
4.584
4.549
4.527
4.059
4.035
3.772
3.767
3.398
3.375
3.367
3.357
3.163
2.440
2.388
2.044
1.629
1.255
0.981
0.971
0.881
-0.000

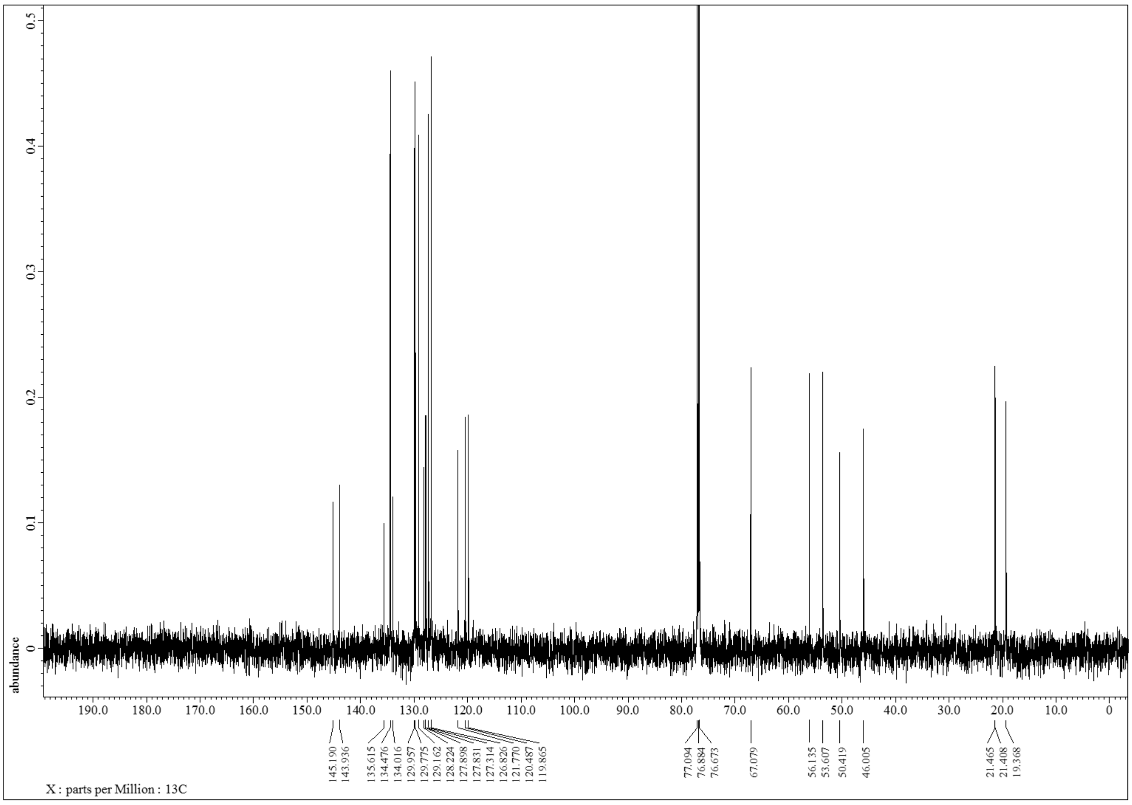
abundance
X : parts per Million : 13C
145.190
143.936
135.615
134.476
134.016
129.957
129.775
129.162
128.224
127.898
127.831
127.314
126.826
121.770
120.487
119.865
77.094
76.884
76.673
67.079
56.135
53.607
50.419
46.005
21.465
21.408
19.368

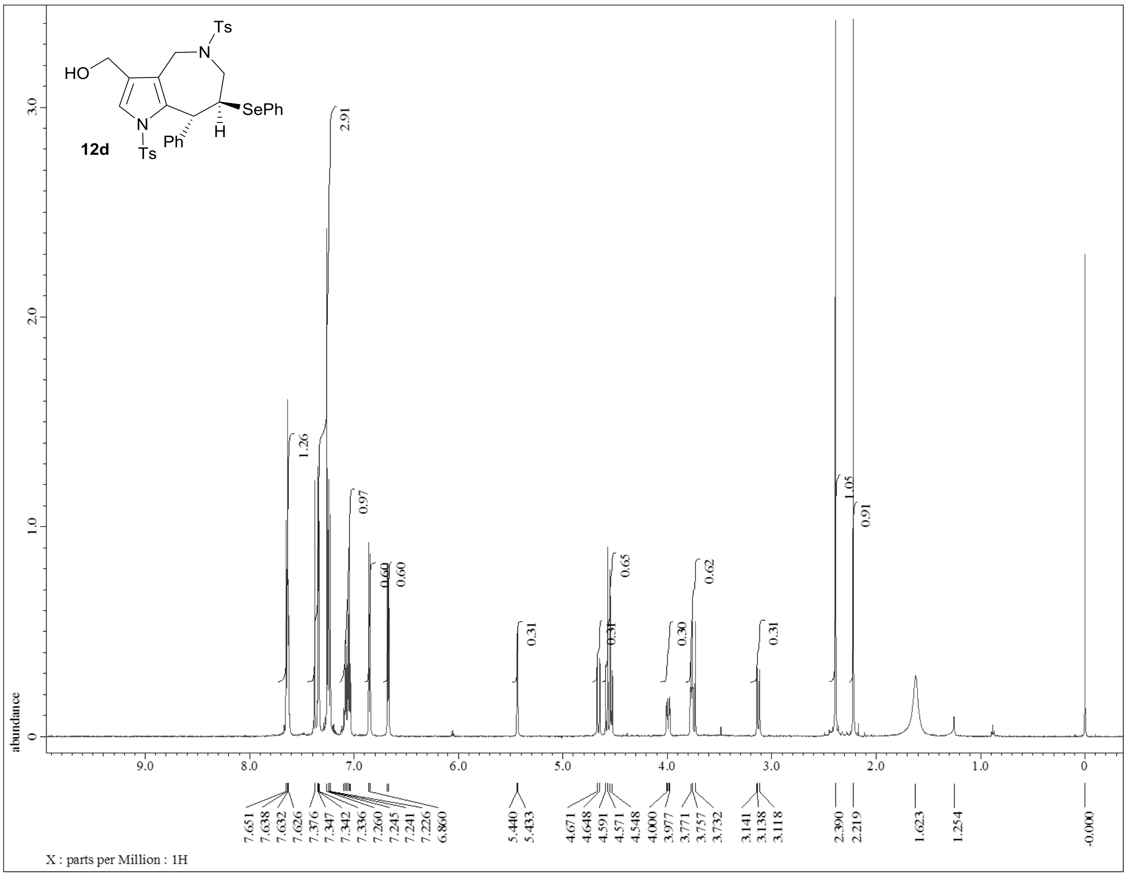
HO
N
Ts
SePh
H
Ph
12d
abundance
X : parts per Million : 1H
1.26
2.91
0.97
0.60
0.60
0.31
0.31
0.65
0.30
0.62
0.31
1.05
0.91
7.651
7.638
7.632
7.626
7.376
7.347
7.342
7.336
7.260
7.245
7.241
7.226
6.860
5.440
5.433
4.671
4.648
4.591
4.571
4.548
4.000
3.977
3.771
3.757
3.732
3.141
3.138
3.118
2.390
2.219
1.623
1.254
-0.000

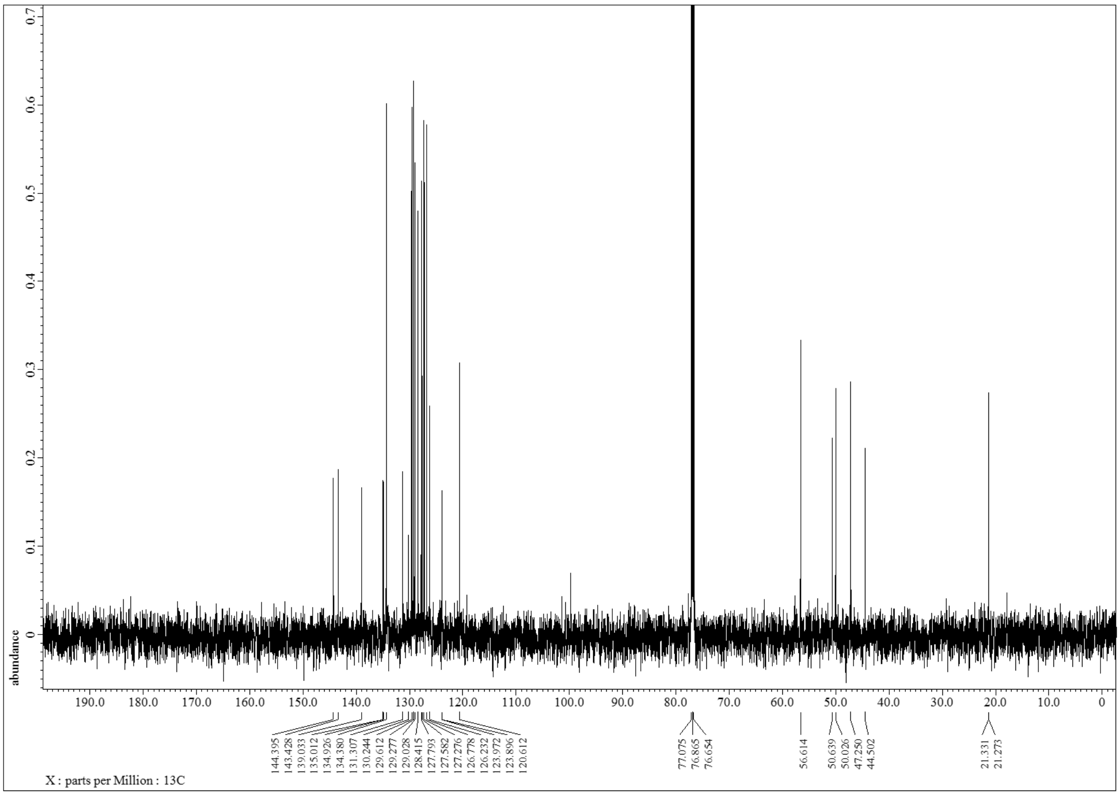
abundance
X : parts per Million : 13C
144.395
143.428
139.033
135.012
134.926
134.380
131.307
130.244
129.612
129.277
129.028
128.415
127.793
127.582
127.276
126.778
126.232
123.972
123.896
120.612
77.075
76.865
76.654
56.614
50.639
50.026
47.250
44.502
21.331
21.273

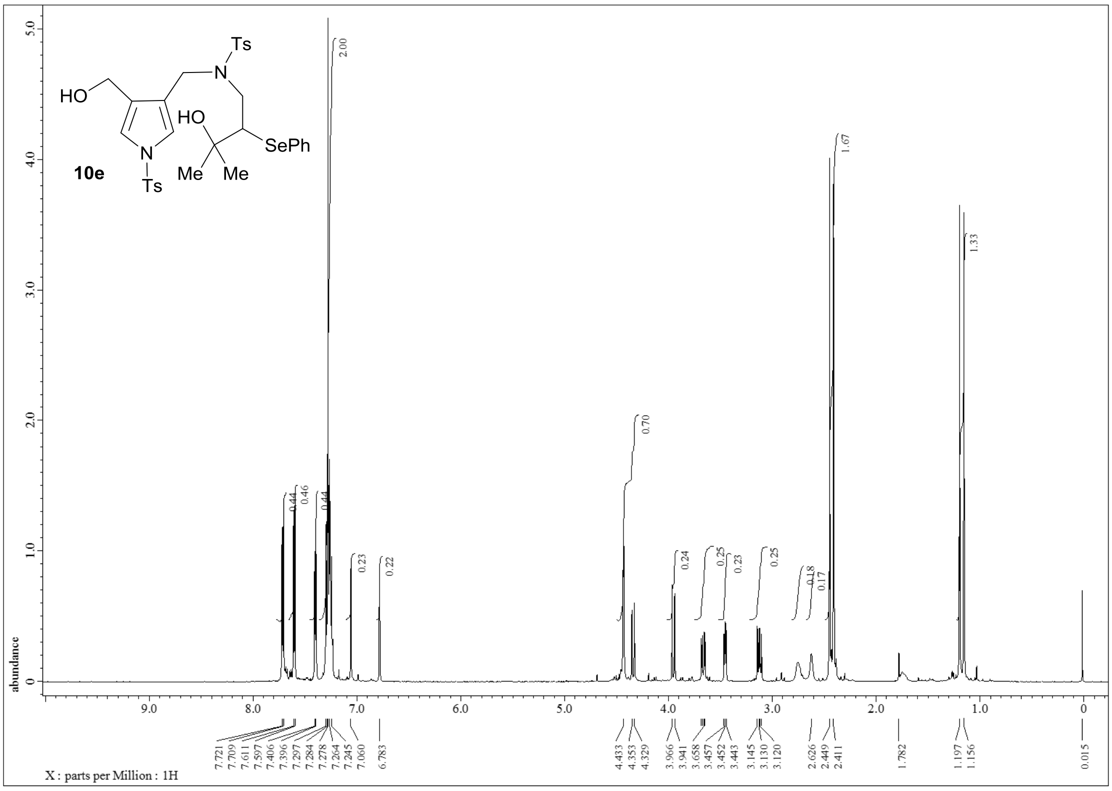

Ts
N
HO
HO
SePh
N
10e
Ts
Me
Me
abundance
X : parts per Million : 1H
7.721
7.709
7.611
7.597
7.406
7.396
7.297
7.284
7.278
7.264
7.245
7.060
6.783
4.433
4.353
4.329
3.966
3.941
3.658
3.457
3.452
3.443
3.145
3.130
3.120
2.626
2.449
2.411
1.782
1.197
1.156
0.015

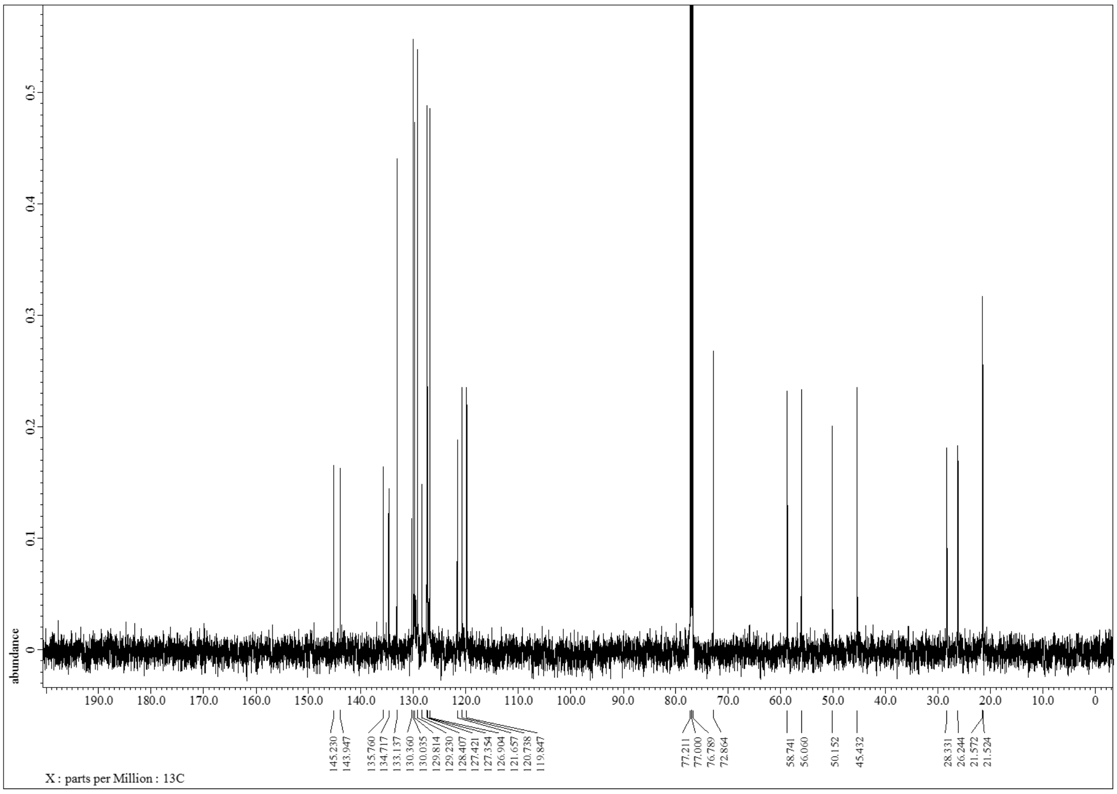

abundance
X : parts per Million : 13C
145.230
143.947
135.760
134.717
133.137
130.360
130.035
129.814
129.230
128.407
127.421
127.354
126.904
121.657
120.738
119.847
77.211
77.000
76.789
72.864
58.741
56.060
50.152
45.432
28.331
26.244
21.572
21.524

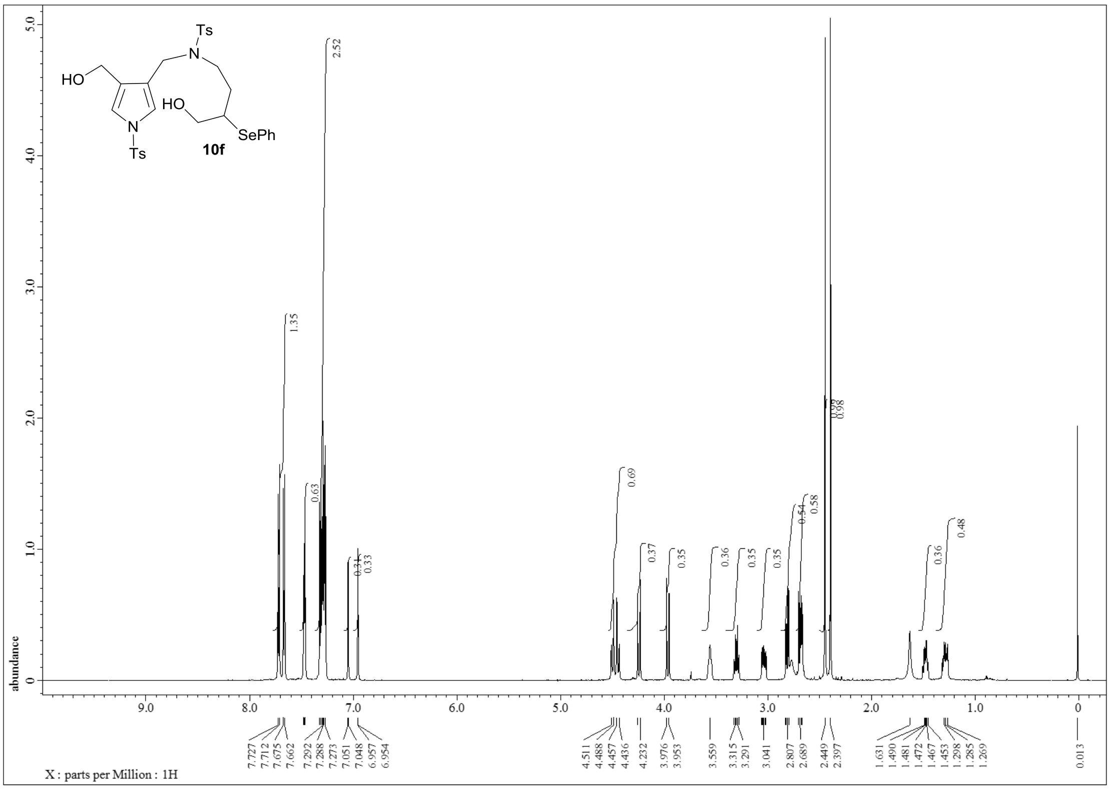
HO
Ts
N
HO
SePh
N
Ts
10f
abundance
X : parts per Million : 1H
2.52
1.35
0.63
0.31
0.33
0.69
0.37
0.35
0.36
0.35
0.35
0.54
0.58
0.98
0.36
0.48
7.727
7.712
7.675
7.662
7.292
7.288
7.273
7.051
7.048
6.957
6.954
4.511
4.488
4.457
4.436
4.232
3.976
3.953
3.559
3.315
3.291
3.041
2.807
2.689
2.449
2.397
1.631
1.490
1.481
1.472
1.467
1.453
1.298
1.285
1.269
0.013

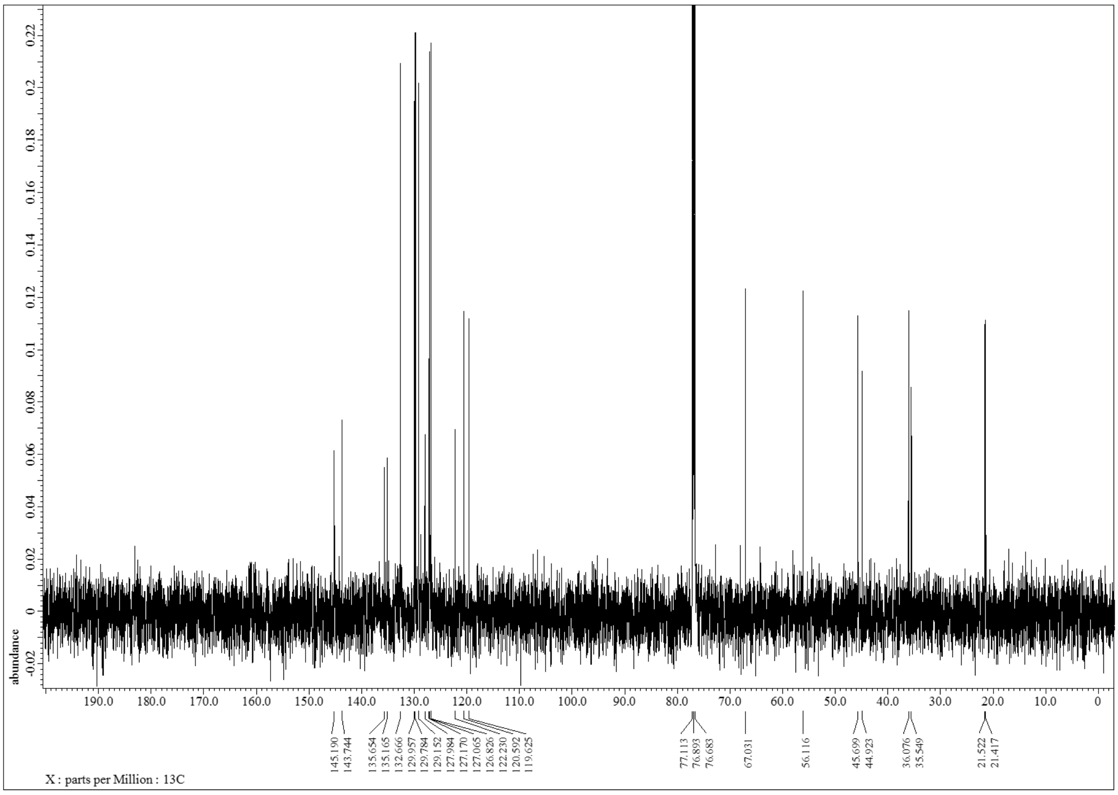
abundance
X : parts per Million : 13C
145.190
143.744
135.654
135.165
132.666
129.957
129.784
129.152
127.984
127.170
127.065
126.826
122.230
120.592
119.625
77.113
76.893
76.683
67.031
56.116
45.699
44.923
36.076
35.549
21.522
21.417

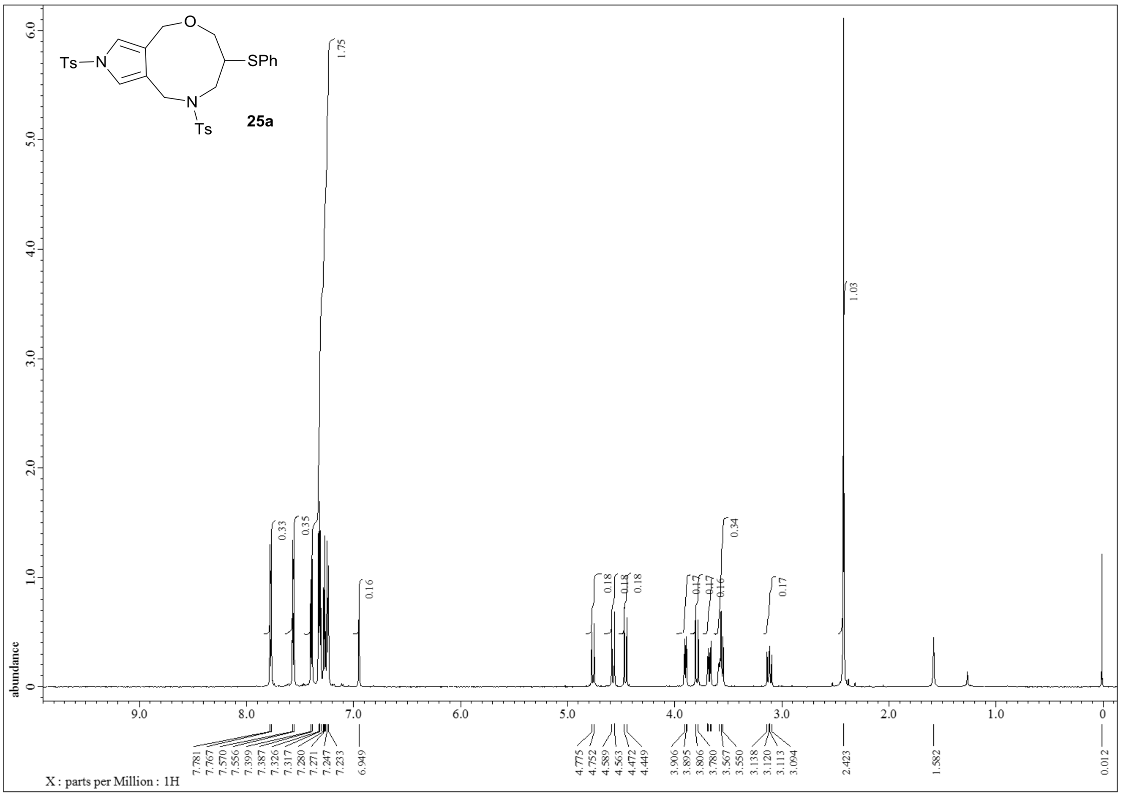
Ts
SPh
25a
abundance
X : parts per Million : 1H
7.781
7.767
7.570
7.556
7.399
7.387
7.326
7.317
7.280
7.271
7.247
7.233
6.949
4.775
4.752
4.589
4.563
4.472
4.449
3.906
3.895
3.806
3.780
3.567
3.550
3.138
3.120
3.113
3.094
2.423
1.582
0.012
0.33
0.35
1.75
0.16
0.18
0.18
0.18
0.17
0.17
0.16
0.34
0.17
1.03

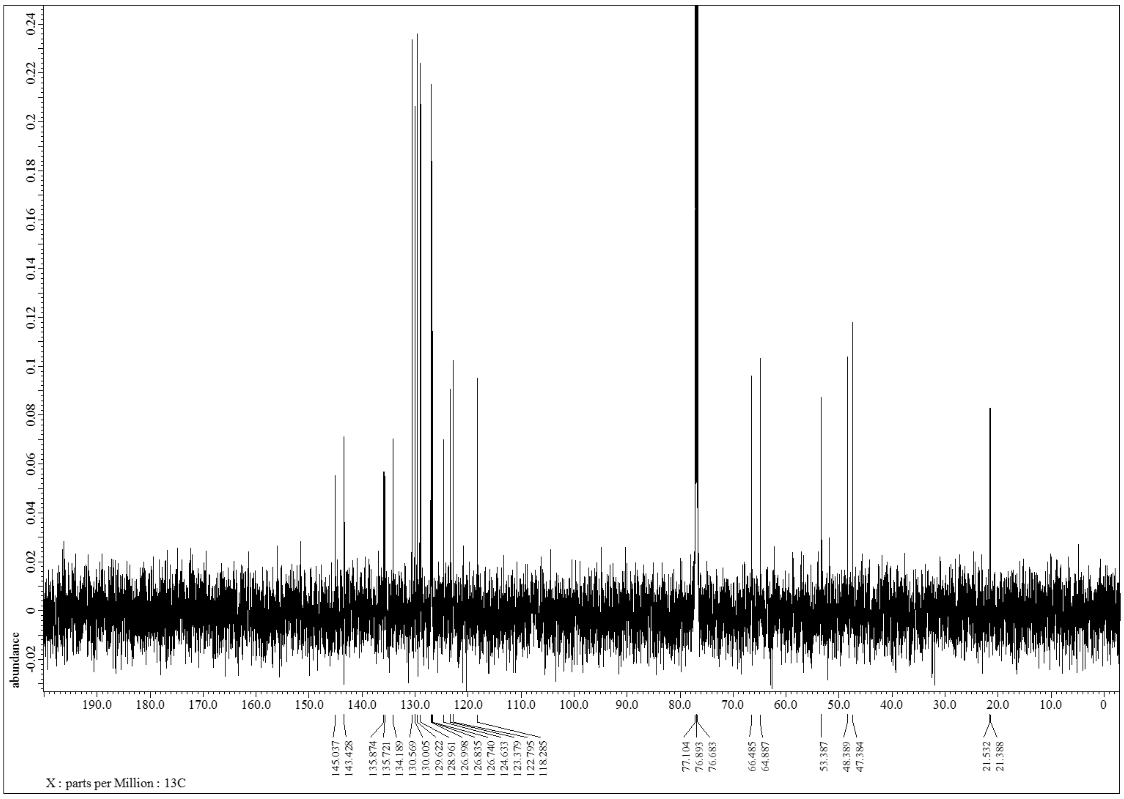
abundance
X : parts per Million : 13C
145.037
143.428
135.874
135.721
134.180
130.569
130.005
129.622
128.961
126.998
126.835
126.740
124.633
123.379
122.795
118.285
77.104
76.893
76.683
66.485
64.887
53.387
48.389
47.384
21.532
21.388

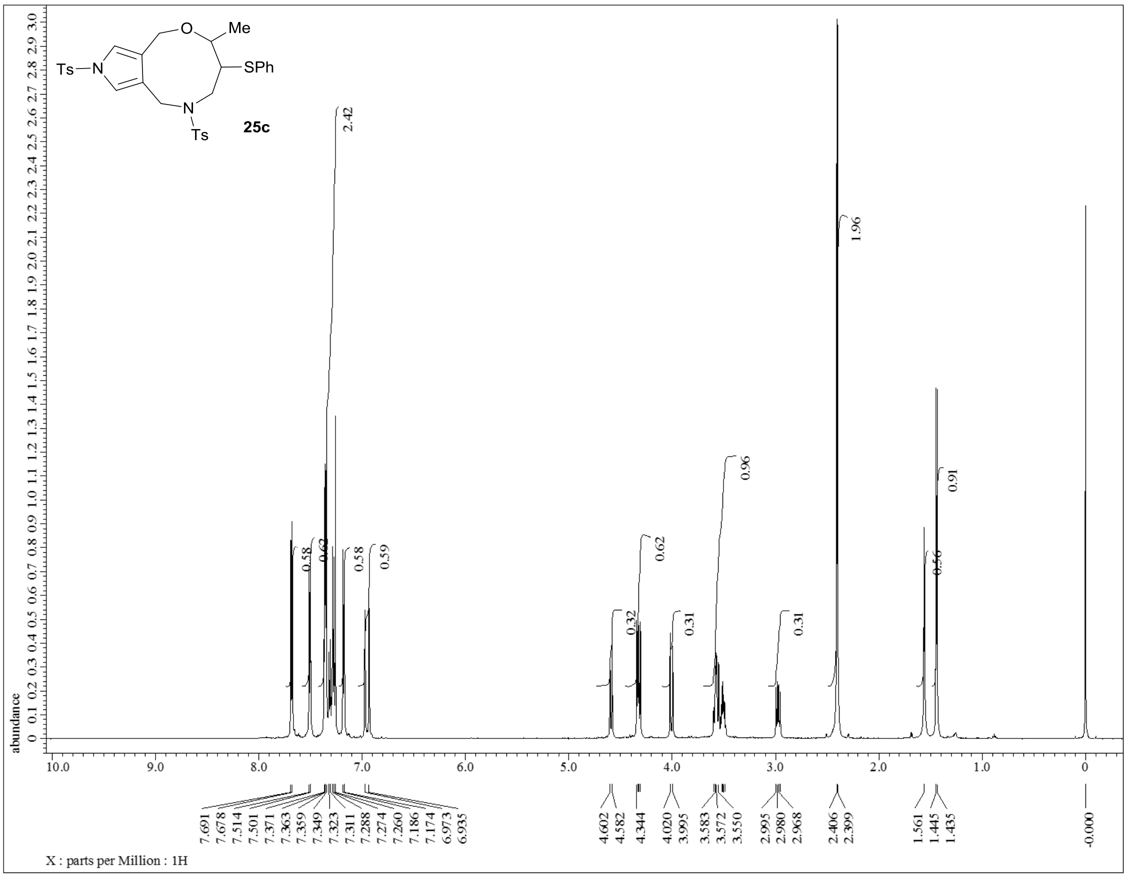
Ts
N
O
Me
SPh
N
Ts
25c
abundance
X : parts per Million : 1H
7.691
7.678
7.514
7.501
7.371
7.363
7.359
7.349
7.323
7.311
7.288
7.274
7.260
7.186
7.174
6.973
6.935
4.602
4.582
4.344
4.020
3.995
3.583
3.572
3.550
2.995
2.980
2.968
2.406
2.399
1.561
1.445
1.435
-0.000

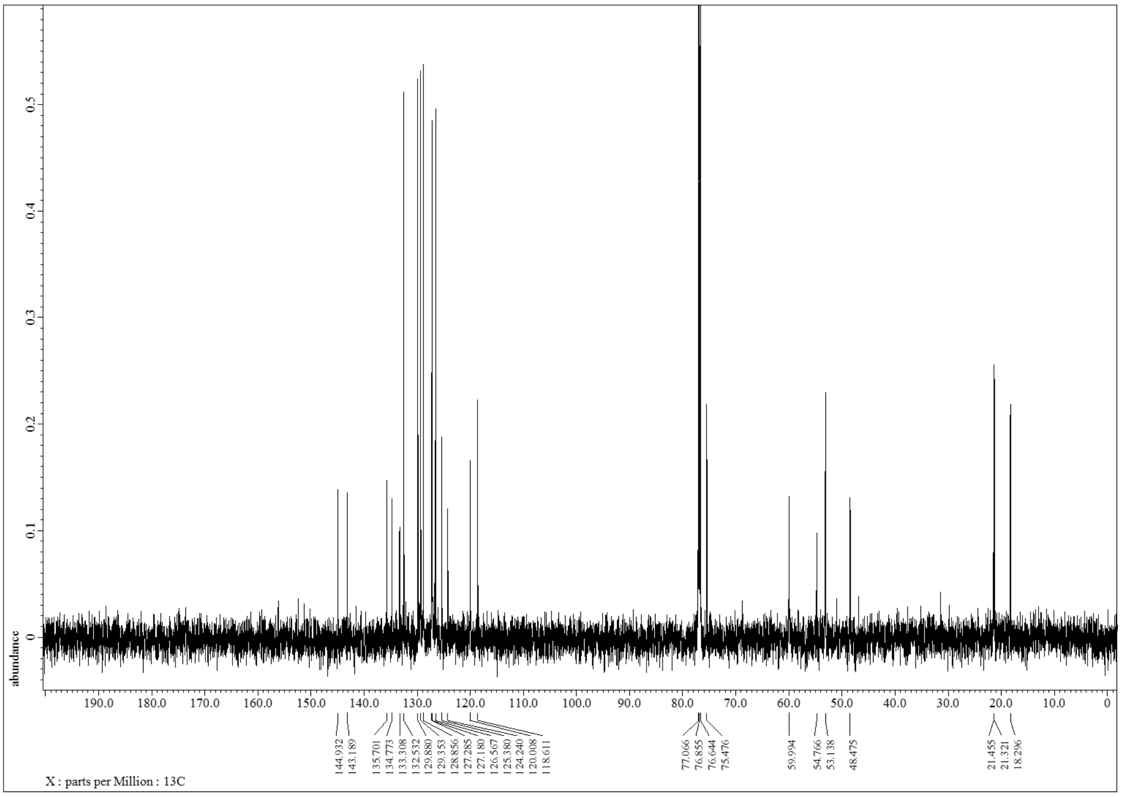
abundance
X : parts per Million : 13C
144.932
143.189
135.701
134.773
133.308
132.532
129.880
129.353
128.856
127.285
127.180
126.567
125.380
124.240
120.008
118.611
77.066
76.855
76.644
75.476
59.994
54.766
53.138
48.475
21.455
21.321
18.296

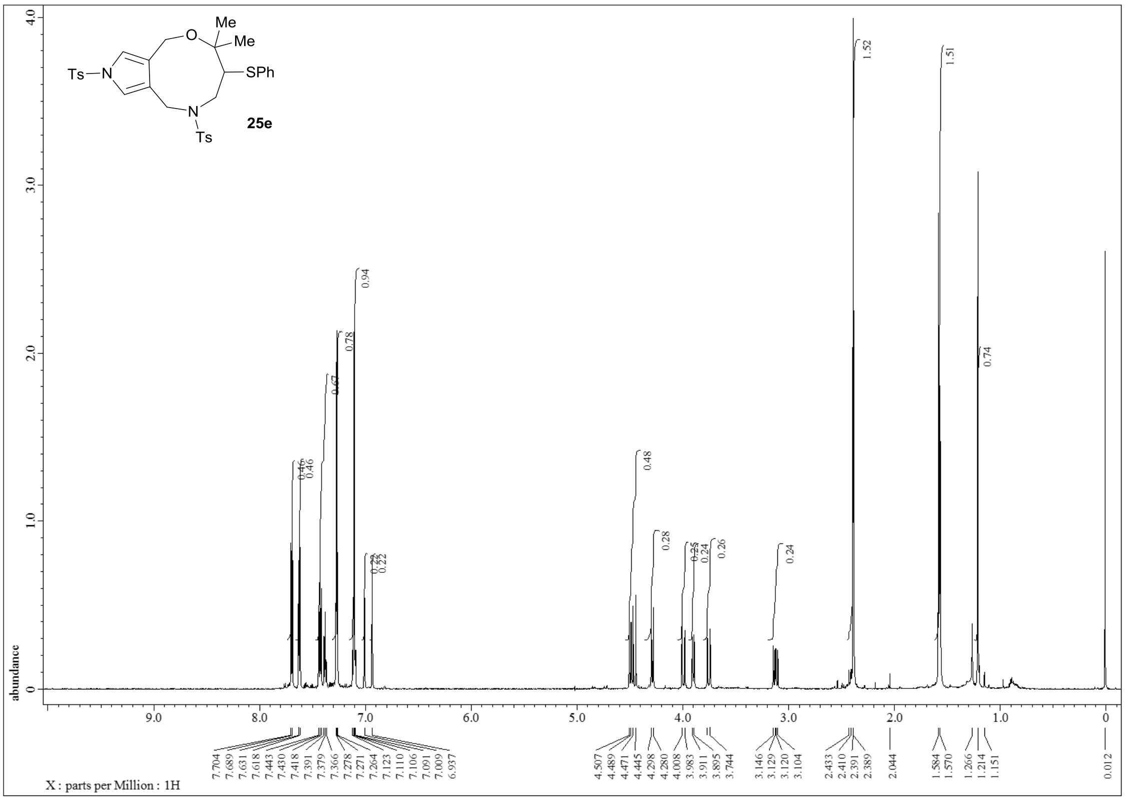
Me
O
Me
Ts–N
SPh
N
Ts
25e
abundance
X : parts per Million : 1H
7.704 7.689 7.631 7.618 7.443 7.430 7.418 7.391 7.379 7.366 7.278 7.271 7.264 7.123 7.110 7.106 7.091 7.009 6.937
4.507 4.489 4.471 4.445 4.298 4.280 4.008 3.983 3.911 3.895 3.744
3.146 3.129 3.120 3.104
2.433 2.410 2.391 2.389
2.044
1.584 1.570
1.266 1.214 1.151
0.012

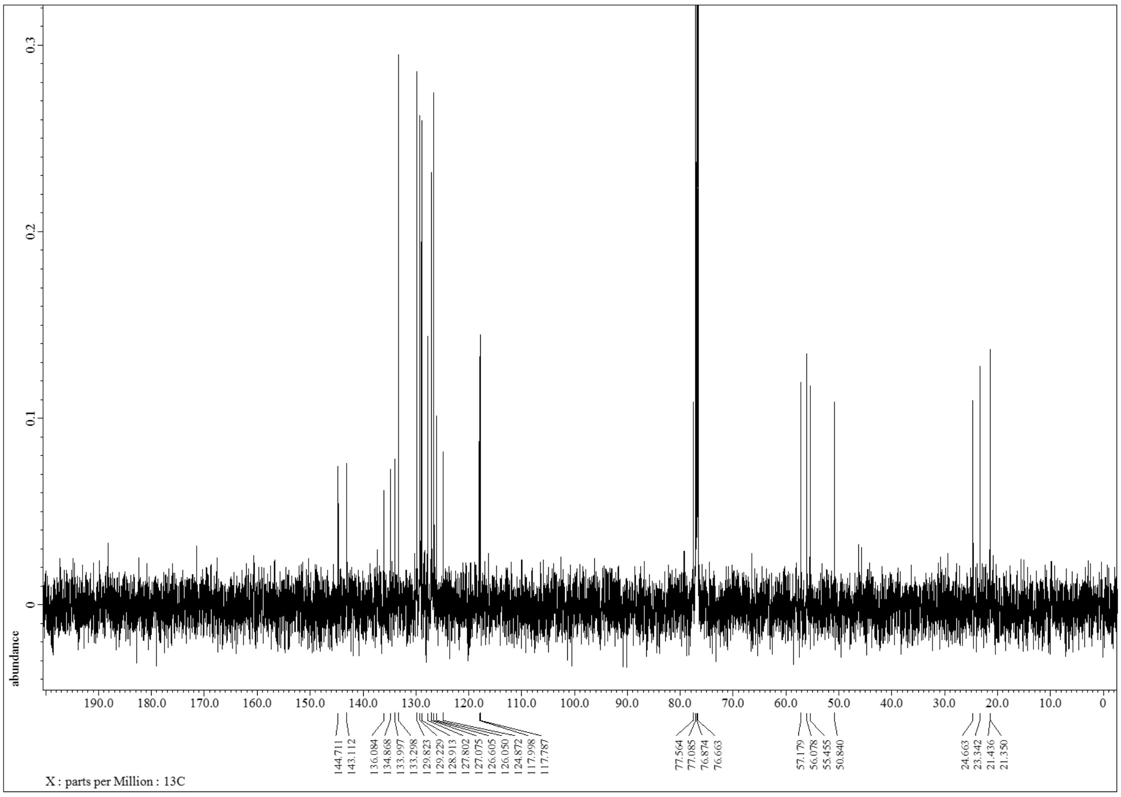
abundance
X : parts per Million : 13C
144.711 143.112 136.084 134.868 133.997 133.298 129.823 129.229 128.913 127.802 127.075 126.605 126.050 124.872 117.998 117.787
77.564 77.085 76.874 76.663
57.179 56.078 55.455 50.840
24.663 23.342 21.436 21.350

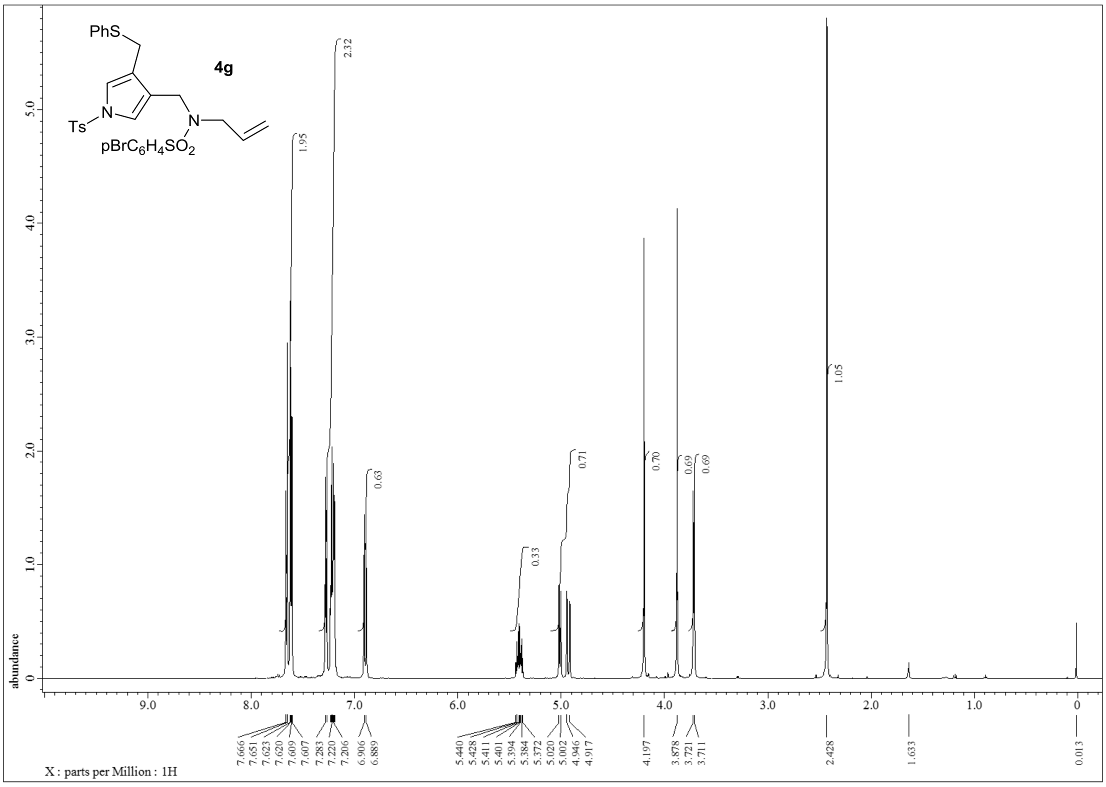
PhS
4g
Ts
N
N
pBrC$_6$H$_4$SO$_2$
1.95
2.32
0.63
0.33
0.71
0.70
0.69
0.69
1.05
abundance
9.0 8.0 7.0 6.0 5.0 4.0 3.0 2.0 1.0 0
7.666 7.651 7.623 7.620 7.609 7.607 7.283 7.220 7.206 6.906 6.889
5.440 5.428 5.411 5.401 5.394 5.384 5.372 5.020 5.002 4.946 4.917
4.197 3.878 3.721 3.711
2.428
1.633
0.013
X : parts per Million : 1H

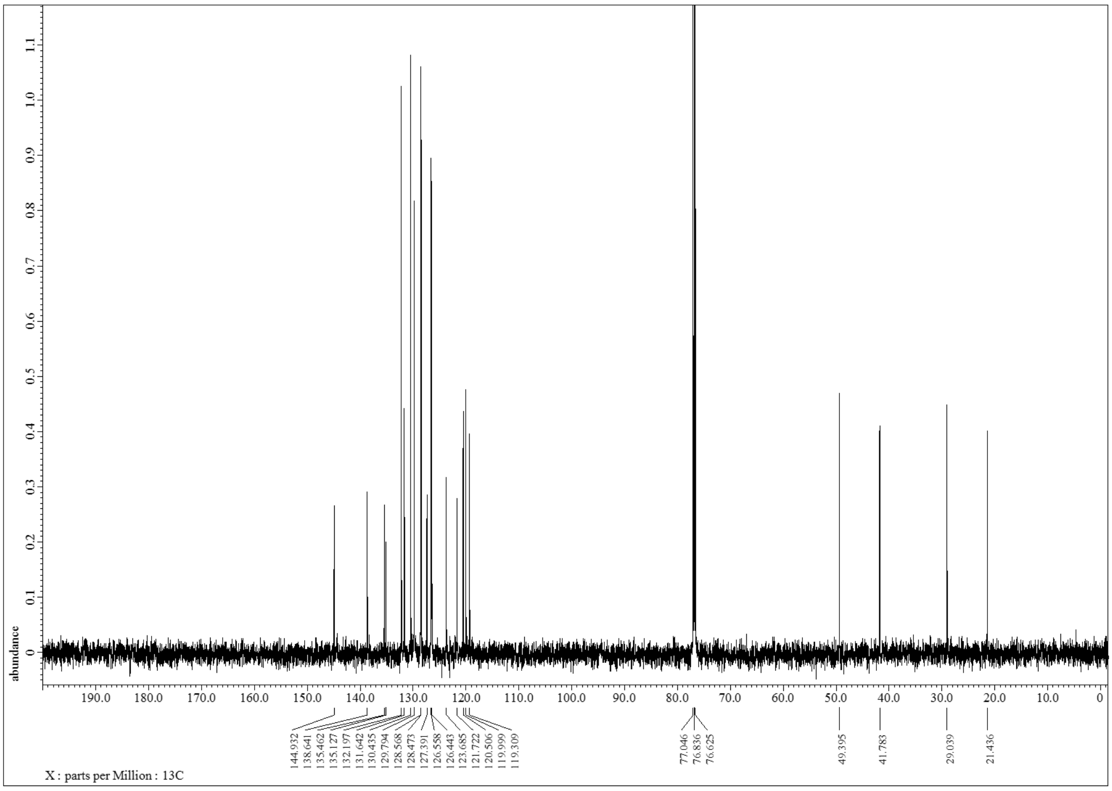
abundance
190.0 180.0 170.0 160.0 150.0 140.0 130.0 120.0 110.0 100.0 90.0 80.0 70.0 60.0 50.0 40.0 30.0 20.0 10.0 0
144.932 138.641 135.462 135.127 132.197 131.642 130.435 129.794 128.568 128.473 127.391 126.558 126.443 123.685 121.722 120.506 119.999 119.309
77.046 76.836 76.625
49.395
41.783
29.039
21.436
X : parts per Million : 13C

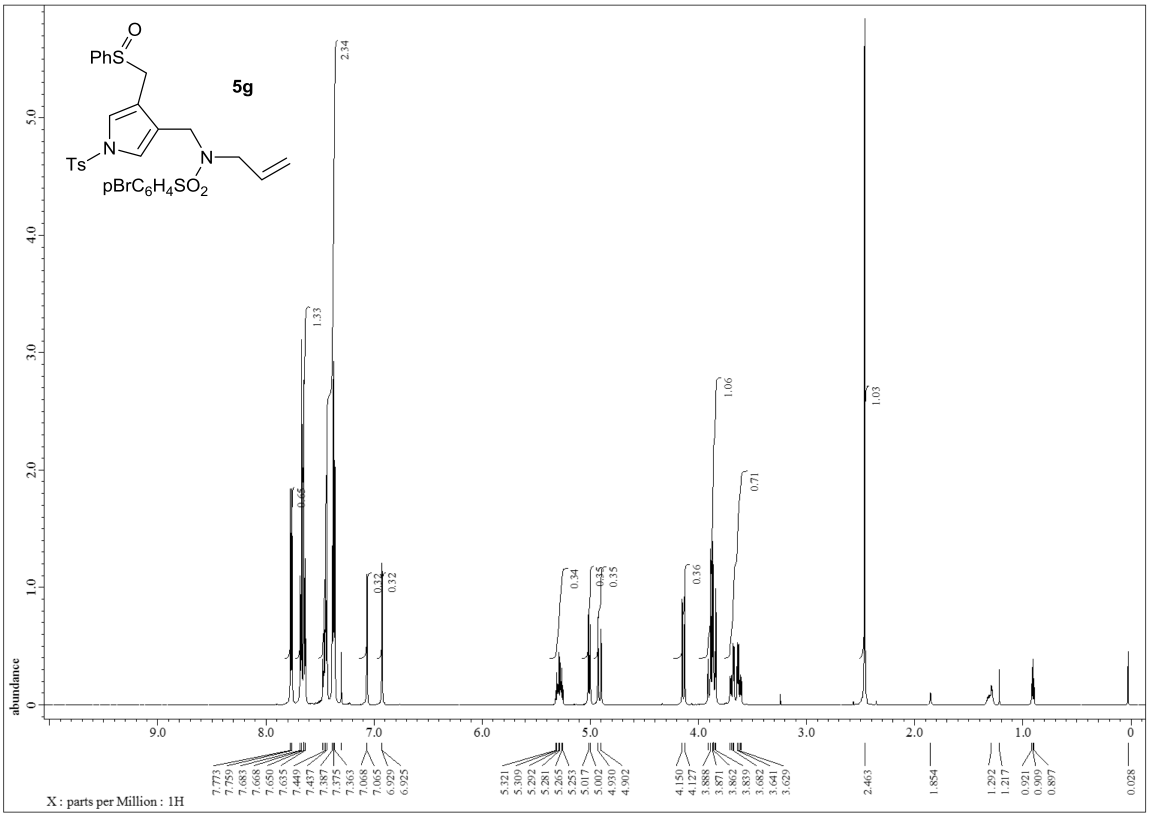
PhS
O
5g
Ts
N
N
pBrC6H4SO2
abundance
X : parts per Million : 1H
2.34
1.33
1.06
1.03
0.71
0.65
0.36
0.35
0.35
0.34
0.32
0.32
7.773 7.759 7.683 7.668 7.650 7.635 7.449 7.437 7.387 7.375 7.363 7.068 7.065 6.929 6.925
5.321 5.309 5.292 5.281 5.265 5.253 5.017 5.002 4.930 4.902
4.150 4.127 3.888 3.871 3.862 3.839 3.682 3.641 3.629
2.463
1.854
1.292 1.217
0.921 0.909 0.897
0.028

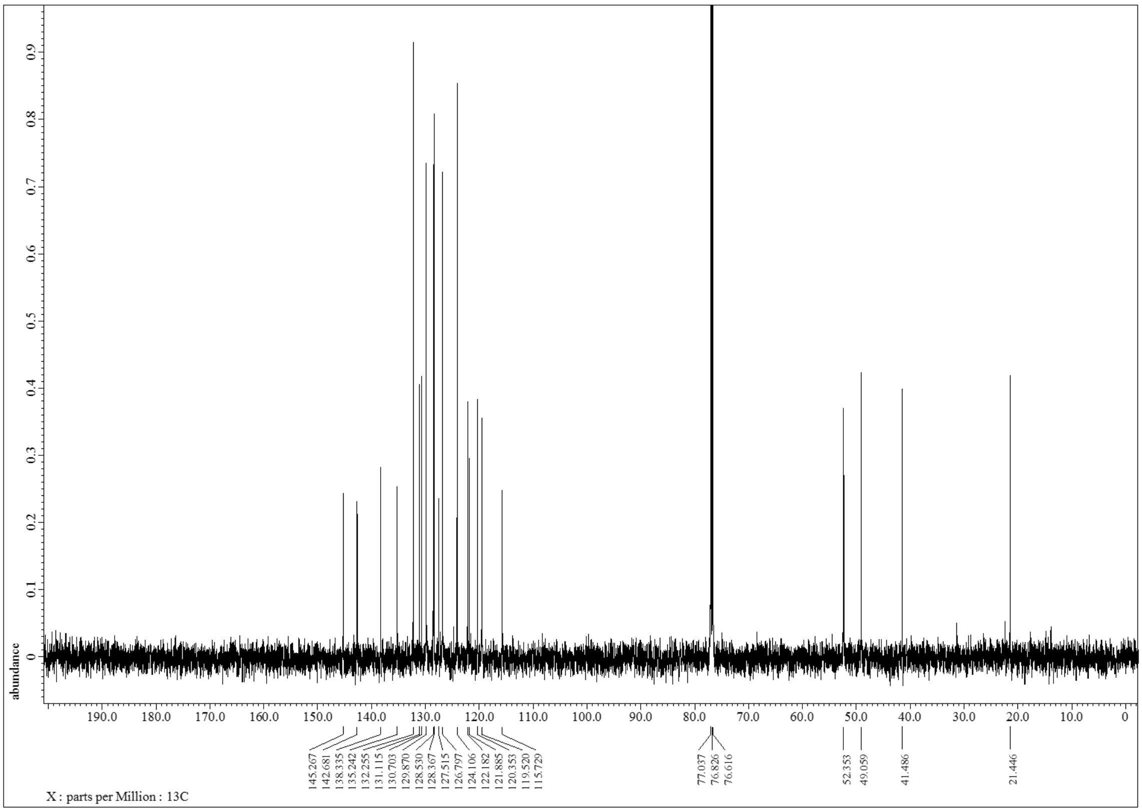
abundance
X : parts per Million : 13C
145.267 142.681 138.335 135.242 132.255 131.115 130.703 129.870 128.530 128.367 127.515 126.797 124.106 122.182 121.885 120.353 119.520 115.729
77.037 76.826 76.616
52.353
49.059
41.486
21.446

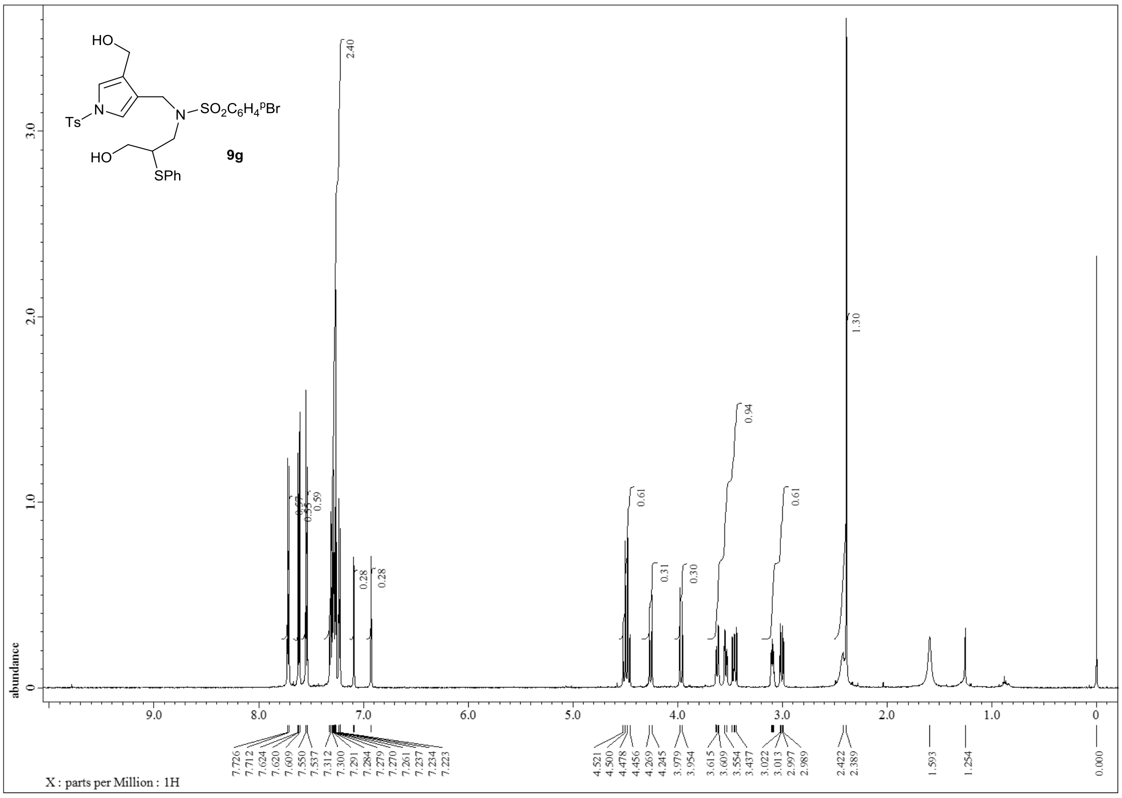
HO
Ts
N
N–SO2C6H4pBr
HO
SPh
9g
abundance
X : parts per Million : 1H

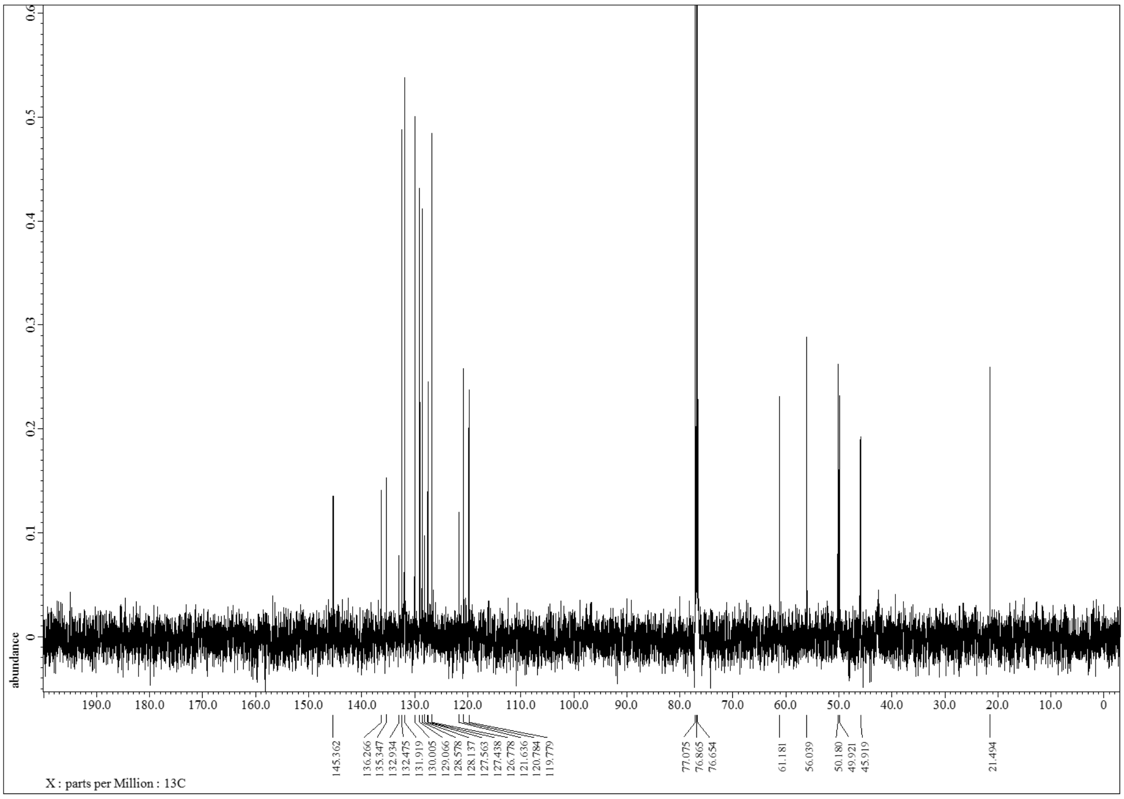
abundance
X : parts per Million : 13C

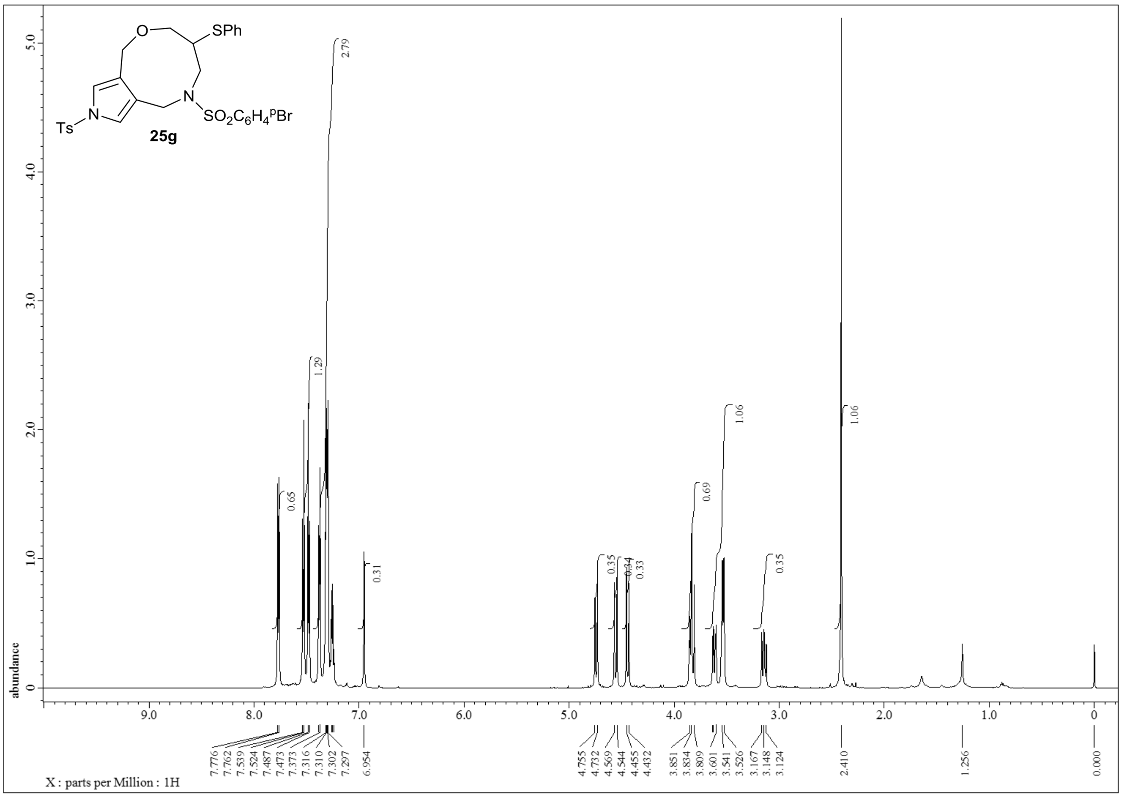

25g
X : parts per Million : 1H
abundance

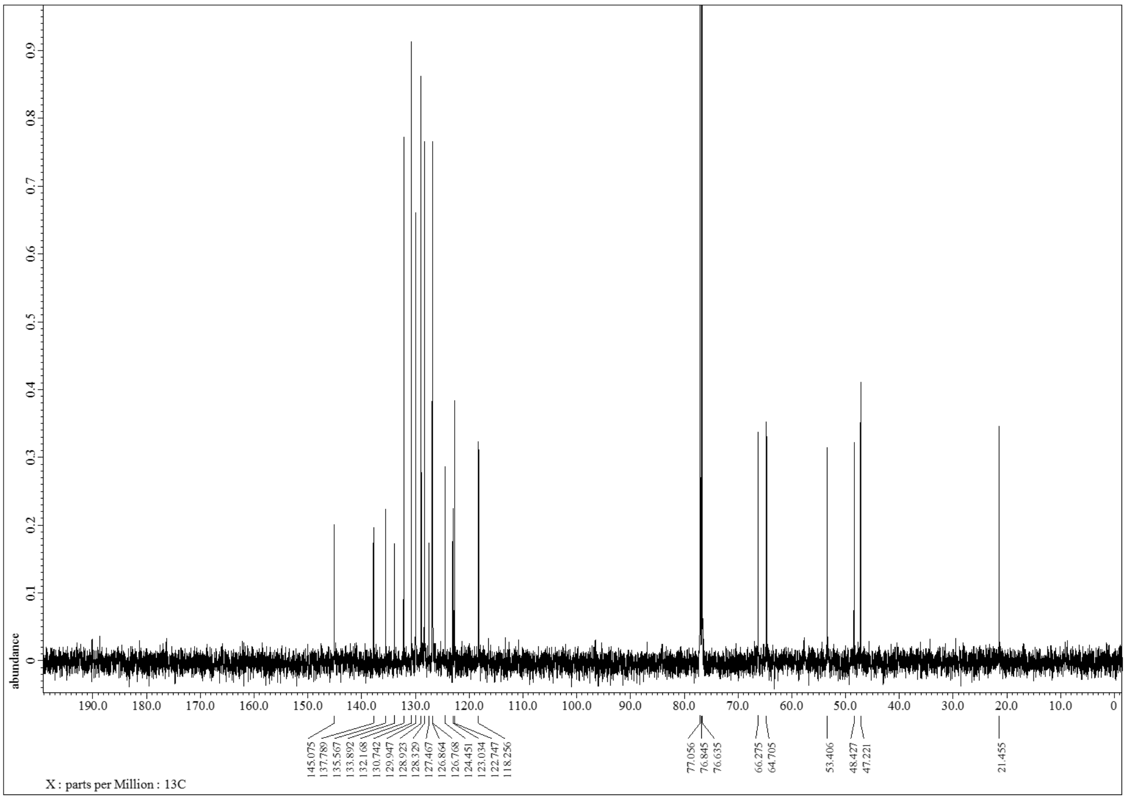

X : parts per Million : 13C
abundance

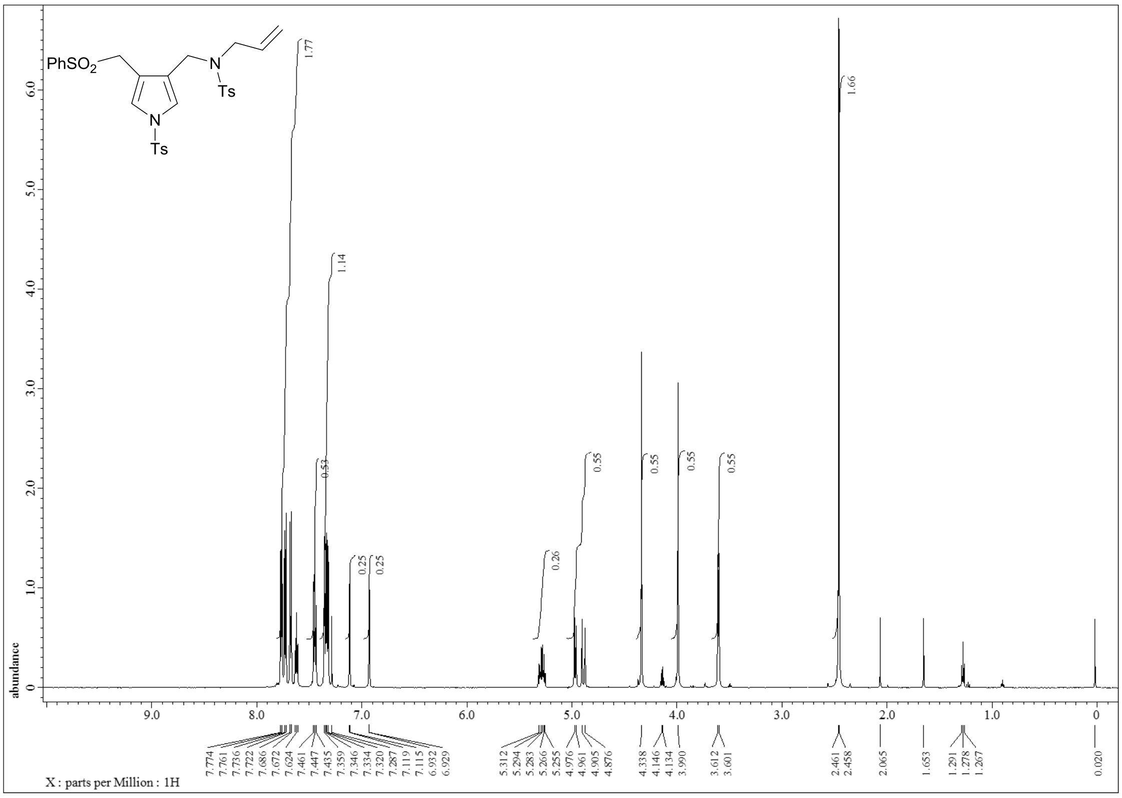
PhSO2
N
Ts
N
Ts
X : parts per Million : 1H
abundance

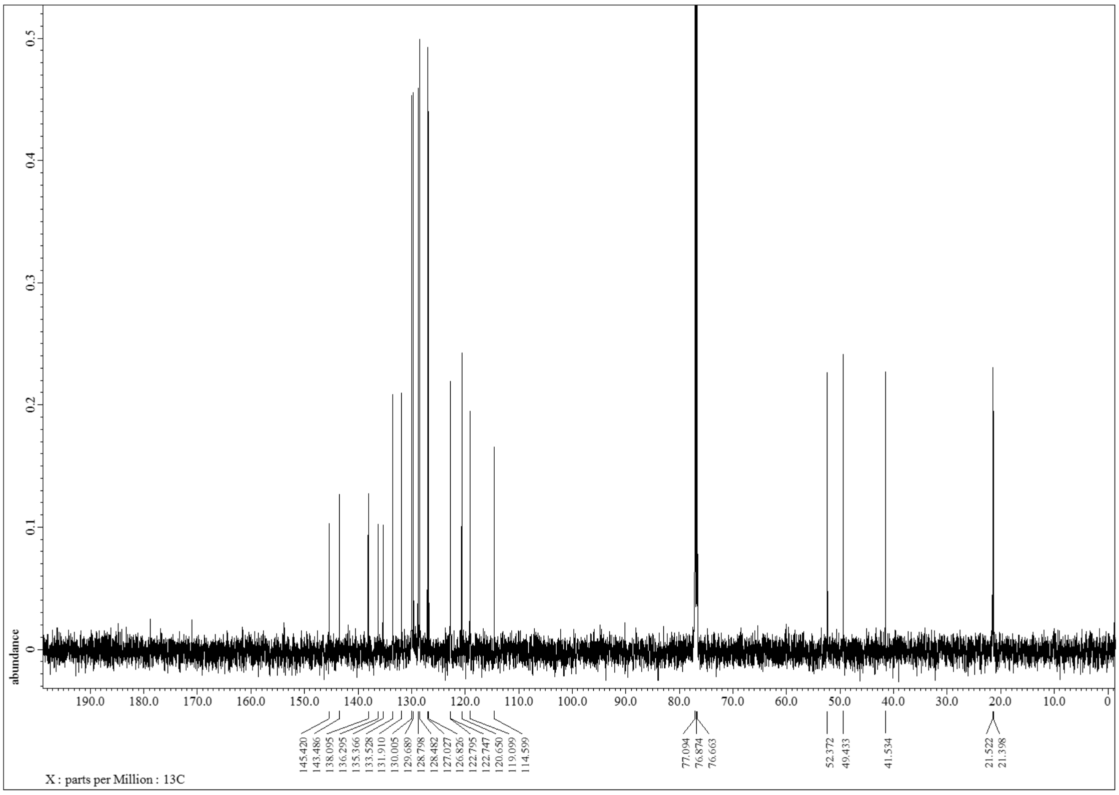
X : parts per Million : 13C
abundance
